# Synthesis of unsymmetrically and symmetrically functionalized disiloxanes via subsequent hydrosilylation of C≡C bonds

**DOI:** 10.1038/s41598-023-37375-8

**Published:** 2023-06-23

**Authors:** Jakub Szyling, Jędrzej Walkowiak, Agnieszka Czapik, Adrian Franczyk

**Affiliations:** 1 Center for Advanced Technology, Adam Mickiewicz University, Uniwersytetu Poznanskiego 10, 61-614 Poznan, Poland; 2 Faculty of Chemistry, Adam Mickiewicz University, Uniwersytetu Poznanskiego 8, 61-614 Poznan, Poland

**Keywords:** Chemistry, Catalysis, Inorganic chemistry, Organic chemistry

## Abstract

A selective synthesis of unsymmetrically functionalized disiloxanes via the subsequent hydrosilylation of internal alkynes in the first step, and alkynes (terminal or internal) or 1,3-diynes in the second, with 1,1,3,3-tetramethyldisiloxane (1) is presented for the first time. Using developed approaches performed in a stepwise or one-pot manner a new family of disubstituted disiloxanes was obtained which had previously been inaccessible by other synthetic methods. Moreover, symmetrically functionalized disiloxanes were obtained by direct hydrosilylation of 2 equivalents of terminal or internal alkynes with 1, showing the unique versatility of the hydrosilylation process. Three examples of symmetric disiloxanes were characterized by single crystal X-ray diffraction for the first time. As a result, a wide group of new compounds which can find potential applications as building blocks or coupling agents was obtained and characterized.

## Introduction

The organosilicon compounds are one of the most important classes of molecules used in modern organic and materials chemistry in academia and industry^1–4^. Due to the almost unlimited possibilities of designing their structure, which influences their properties, such compounds are widely used as e.g., hybrid materials^5^, anti-corrosive coatings^6^, silicon rubbers^7^, and active pharmaceutical indigents (API)^8^. Among several methods for the preparation of organosilicon compounds, transition metal-catalyzed (TM) hydrosilylation of unsaturated bonds is one of the most important because of its 100% atom economy, high selectivity, and good functional group tolerance^9^.

The bifunctional organosilicon compounds, such as 1,1,3,3-tetramethyldisiloxane (**1**), are attractive building blocks that can be applied as polymeric electrolytes^10^, coupling agents^11^, or conjugated polymers^12^, depending on the groups attached to the silicon atom. The simplest dihydride siloxane **1** is produced as a by-product of the silicon industry. Because of its low-boiling point, relatively low price, and good stability in air and moisture, it is used as a versatile scaffold for the synthesis of advanced molecules^13^.

The mono- or bifunctionalization of **1** via the hydrosilylation of alkenes or allyl compounds with various substituents (e.g., epoxy^14^, aminopropyl^15^, *γ*-methacryloxypropyl^16^, boryl/germyl^17^, silyl^18^) is well-established. The synthesis of the symmetrically functionalized disiloxanes by hydrosilylation of alkynes^19–25^ or other methods^26–28^ was described in a few works in which disiloxanes mainly with monosubstituted alkenyl groups^20–26,28–31^ were obtained that were subsequently used in Hiyama coupling^19–22,24,25,30,31^. Reports on the synthesis of disiloxanes with disubstituted alkenyl groups are almost neglected and are limited to four compounds with no functional groups attached^19,29,32^. On the other hand, there are no examples of unsymmetrical functionalized derivatives bearing two different alkenyl groups.

Bearing in mind the above-described information, based on our experience in the utilization of the carbon-carbon triple (C≡C) bonds in hydrosilylation reactions^33–43^, we decided to develop a simple and straightforward synthetic protocol for obtaining new unsymmetrically functionalized disiloxanes via subsequent hydrosilylation of alkynes in the first step, and alkynes or 1,3-diynes in the second, using stepwise or one-pot approaches. The stepwise method gives an opportunity for the detailed characterization of both hydrosilylation processes and products of monofunctionalization (RSiMe_2_OSiMe_2_H). Isolation of monofunctionalization products allows the type of catalyst to be changed after the first hydrosilylation which opens new possibilities for the modification of the Si-H group. On the other hand, the one-pot method allows for avoiding the isolation of products (RSiMe_2_OSiMe_2_H) and the addition of a second portion of catalyst if the same systems can be used for both steps. Moreover, we planned to study the synthesis of the symmetrically functionalized disiloxanes obtained by the direct hydrosilylation of two equivalents of alkynes (terminal or internal) with disiloxane **1**, in the presence of different catalysts. This will show the unique utility of the hydrosilylation process to obtain all possible products using one method. It cannot be done by the use of any other reported method.

## Results and discussion

**Figure 1:**
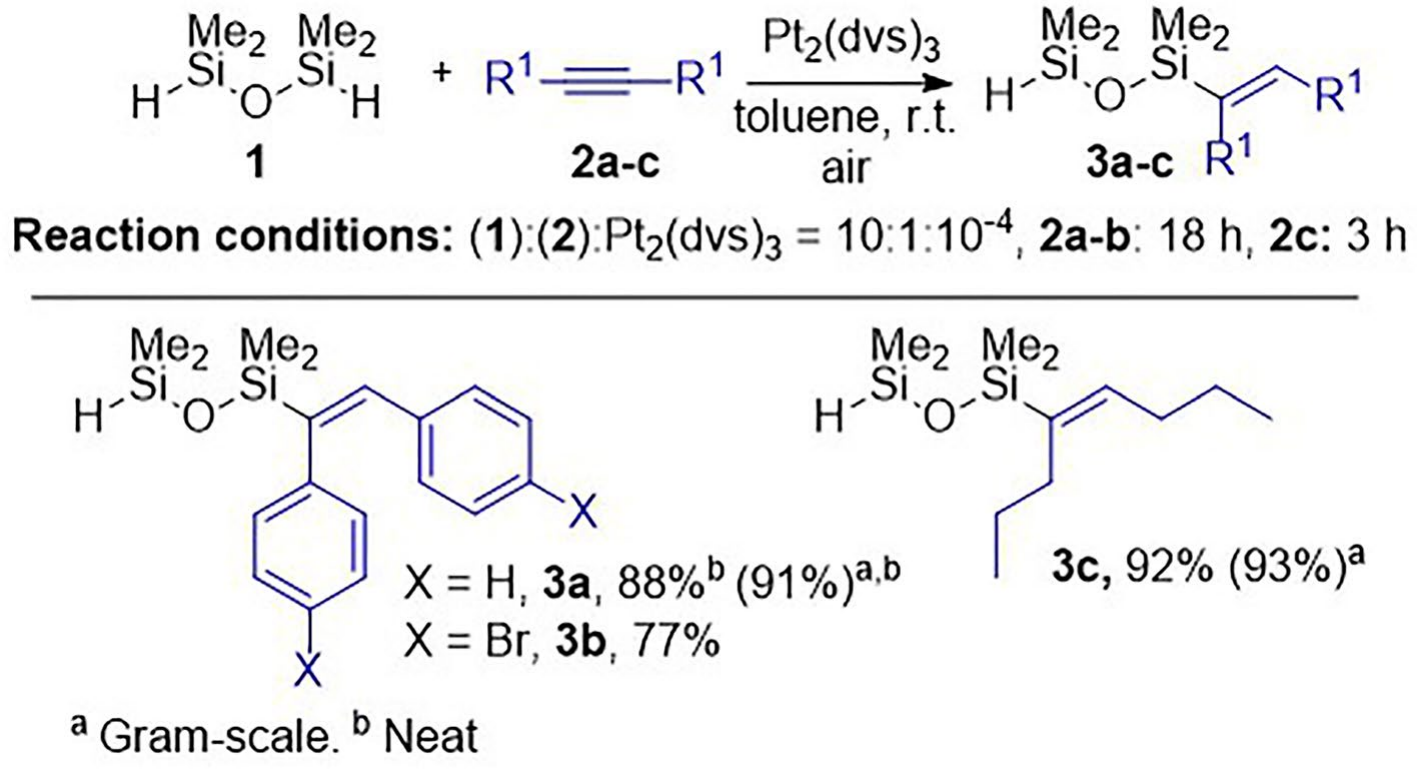
Hydrosilylation of internal alkynes **2a‒c** with **1** towards the monofunctionalized disiloxane derivatives **3a‒c**.

In the first stage of the research, the monofunctionalization of 1,1,3,3-tetramethyldisiloxane (**1**) was optimized. A series of terminal and internal alkynes (**2**) were tested in the hydrosilylation with **1** in the presence of various catalysts and under different conditions. It was found that for diphenylethyne **2a**, a high yield of monofunctionalization product **3a** was observed if the reaction was performed at room temperature. The requirement for selective activation of only one Si-H bond was the use of a 10-fold excess of disiloxane **1** in the presence of Karstedt’s catalyst (Pt_2_(dvs)_3_) (Fig. 1). If a lower amount of **1** was added, both Si-H groups reacted and symmetrical disiloxanes with two of the same alkenyl groups were formed. After the full conversion of **2a**, the excess of **1** was evaporated from the post-reaction mixture, and the targeted product was isolated by filtration through silica pad to remove the catalyst and some by-products obtained from the decomposition of disiloxane **1**.

With the optimized reaction conditions in hand, we tested two other internal alkynes. The hydrosilylation of 1,2-bis(4-bromophenyl)ethyne (**2b**) and aliphatic 4-octyne (**2c**) gave hydrosilylation products **3b** and **3c** with excellent selectivity and high isolation yields. For both, the formation of side hydrosilylation products was not observed. Moreover, the synthesis of **3a** and **3c** was performed on a gram-scale, giving monoalkenyl-functionalized disiloxane derivatives **3a**‒**c** with very high isolation yields. These compounds have been reported for the first time and are perfect reagents for further transformations typical of the Si-H group. The attempts to obtain analogue products with terminal alkynes were unsuccessful despite using different catalysts, the stoichiometry of reagents, and reaction conditions.

**Figure 2:**
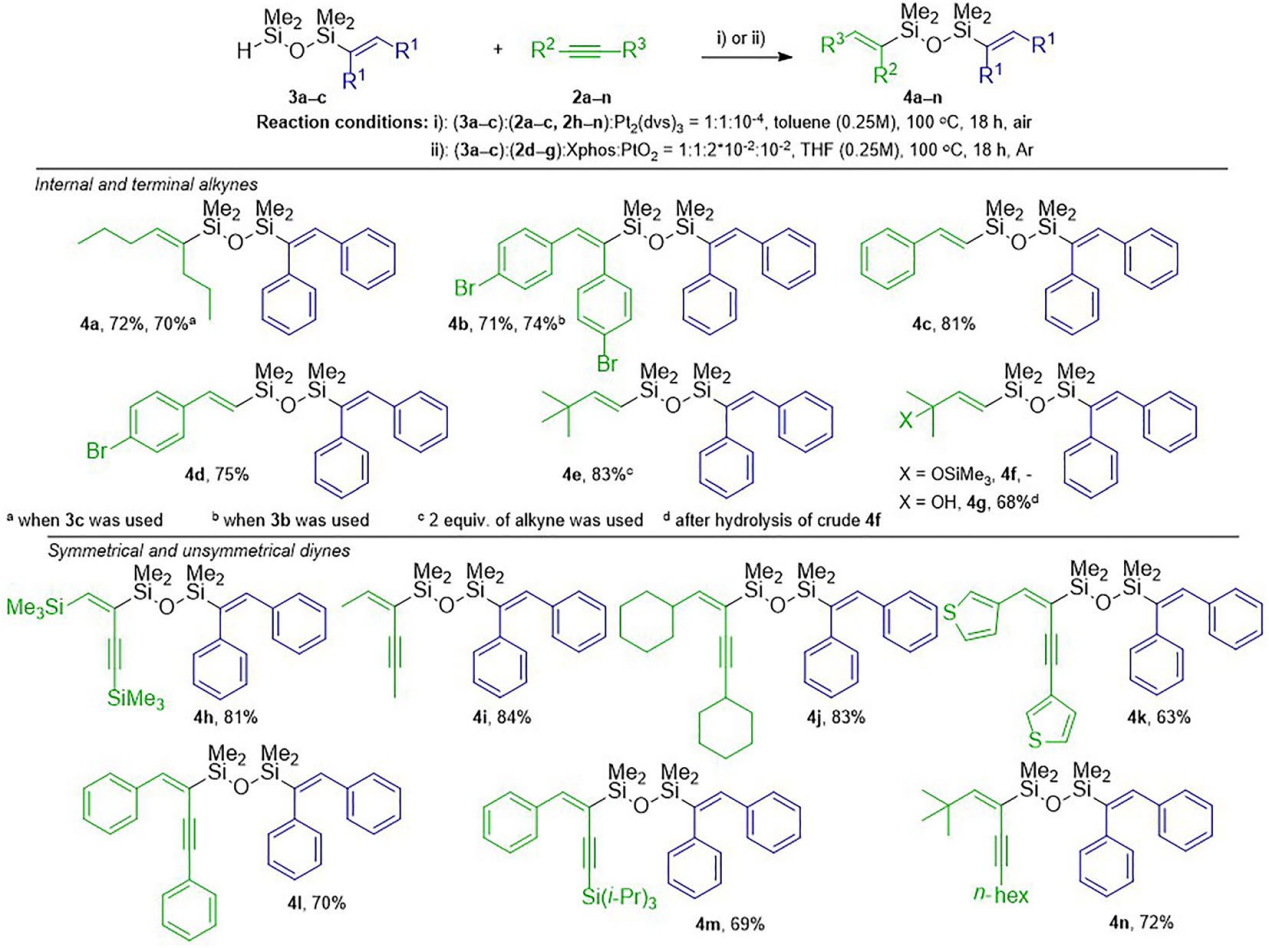
The synthesis of unsymmetrical disiloxane derivatives **4a‒n** via the hydrosilylation of alkynes and 1,3-diynes with **3a‒c**.

In the next stage of the study, we used **3a**‒**c** in the subsequent hydrosilylation of internal and terminal alkynes, as well as 1,3-diynes, to obtain unsymmetrically functionalized disiloxanes **4a**‒**n** (Fig. 2). The hydrosilylation of 4-octyne (**2c**) with **3a** yielded **4a** with a good isolation yield in 18 h at 100 °C. The same product was obtained through the hydrosilylation of diphenylethyne (**2a**) with **3c**, with a similar yield and selectivity. The application of bis(4-bromophenyl)ethyne (**2b**) and **3a** gave **4b** in a good isolation yield. Similarly, **4b** could also be obtained via the hydrosilylation of **2a** with **3b**, without the loss of selectivity. In contrast to internal alkynes, hydrosilylation of terminal alkynes with monofunctionalized disiloxanes **3** was performed in the presence of the PtO_2_/Xphos catalytic system under inert gas, to ensure the highest possible selectivity of the process.

The hydrosilylation of phenylacetylene (**2d**) with **3a** provided **4c** with excellent selectivity (*trans*/*gem* = 99/1) and a very good isolation yield. Very similar results were observed when 4-bromophenylacetylene (**2e**) was used as the reagent. The applied catalytic system was also efficient for the hydrosilylation of sterically hindered 3,3-dimethylbut-1-yne (**2f**) and trimethyl((2-methylbut-3-yn-2-yl)oxy)silane (**2g**) to give β-(*E*)-isomers **4e** and **4f** exclusively. Compound **4f** was further easily converted through hydrolysis to **4g** with a good isolation yield (68%), without any changes in selectivity. It is worth underlining that the possibility of the use of a different catalyst in the second step of modification is undoubtedly an advantage of the stepwise method since Karstedt’s catalyst is not selective in the reaction with terminal alkynes and thus the above-described products cannot be obtained.

Similar to the internal and terminal alkynes, the reactivity of more challenging reagents 1,3-diynes (symmetrical and unsymmetrical) was tested. The presence of two C≡C bonds makes this class of compounds attractive building blocks and at the same time, it is a great challenge to modify them in a selective manner. The hydrosilylation of 1,4-bis(trimethylsilyl)buta-1,3-diyne (**2h**) with **3a** in the presence of Karstedt’s catalyst yielded product **4h** with alkenyl and 1,3-enynyl moieties attached to the disiloxane core. The silicon atom was bonded with the internal carbon adjacent to the second C≡C bond. This was confirmed by 1D nOe NMR (see ESI, Fig. S38, page S35). The same regio- and stereoselectivity was observed for aliphatic 1,3-diynes substituted with methyl (**2i**) and cyclohexyl groups (**2j**). The hydrosilylation of symmetrical 1,4-substituted 1,3-diynes with thienyl (**2k**) and phenyl (**2l**) groups led to the stereoselective formation of expected addition products **4k** and **4l** with high isolation yields. We also tested the hydrosilylation of two unsymmetrical 1,3-diynes (**2m** and **2n**) with **3a**. The functionalization of **2m** substituted with phenyl and tri*iso*propyl groups occurred in a stereo- and regioselective manner, giving a pure product **4m**. The silicon atom was attached to the internal carbon of the 1,3-diyne scaffold, whereas the alkenyl proton was bonded to the Cα connected to the phenyl ring. The hydrosilylation of **2n** diyne with aliphatic *t*-butyl and *n*-hexyl substituents led to the product of monoaddition **4n** with a 72% yield. The structures of products **4m** and **4n** and the regioselectivity of the processes were confirmed by 1D nOe NMR (see ESI, Figs. S57 and S59, pages S45 and S46).

**Figure 3:**
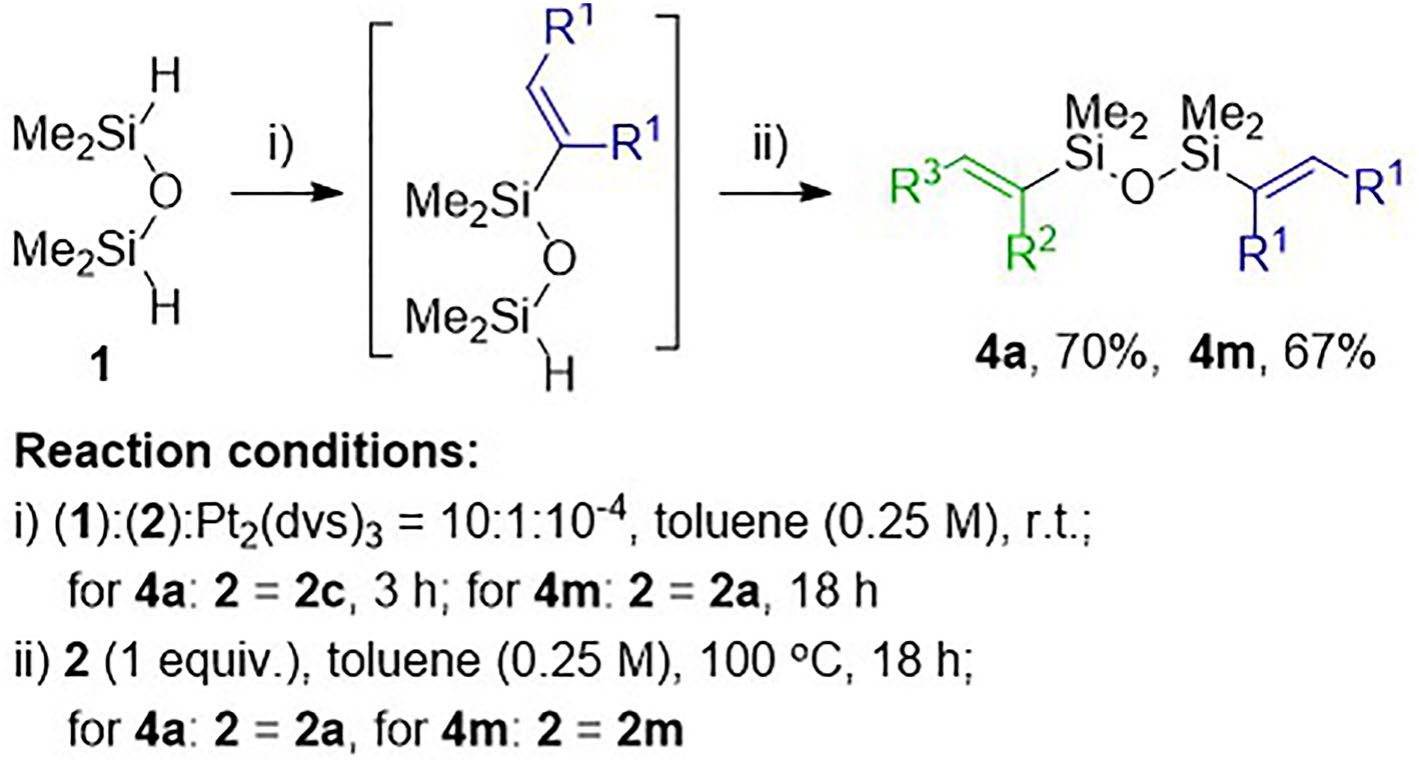
Synthesis of unsymmetrical disiloxanes **4a** and **4 m** via one-pot, subsequent hydrosilylation reactions.

The facts that in the stepwise, subsequent hydrosilylations of internal alkynes **2a**‒**c** with disiloxane **1** in the first step, and internal alkynes (**2a**‒**c**) or 1,3-diynes (**2h**‒**n**) with monofunctionalized siloxanes **3a**‒**c** in the second, Karstedt’s catalysts were used, encouraged us to perform the synthesis of **4a** and **4m** in a one-pot manner (Fig. 3). Moreover, we could check if one portion of the catalyst was going to be efficient for both transformations. Moreover, the work-up concerning the isolation of monofunctionalization products **3a**‒**c** would be unnecessary. Therefore, we reacted alkyne 4-octyne (**2c)** with the 10-fold excess of disiloxane **1** in the presence of Karstedt’s catalyst (10^−4^ mol) at room temperature. The excess of **1** was evaporated after 3 hours when the complete conversion of alkyne **2c** was confirmed. Subsequently, 1,4-diphenyletyne (**2a**) and toluene were added, and the reaction mixture was heated up to 100 °C. After 18 hours, the reaction had finished and product **4a** was obtained. The selectivity of the one-pot process and the isolated yield of **4a** were found to be similar to those obtained using the stepwise method. By the same method product **4m** was synthesized. However, the synthesis of **3a** was performed at room temperature for 18 hours as it was optimized for the stepwise approach.

All synthesized unsymmetrically substituted disiloxanes **4a**‒**n** were obtained and characterized for the first time. They constitute a novel class of organosilicon compounds possessing the Si-O-Si core suited to further functionalization due to the presence of reactive alkenyl, 1,3-enynyl moieties and other functional groups like Br or OH. It is worth emphasizing that, although studies on the synthesis of molecules based on a disiloxane core have been broadly carried out, there is no other method which allows the synthesis of analogues to **4a**‒**n** systems possessing both di- or di- and monosubstituted alkenyl groups in the structure. Subsequent hydrosilylations of the C≡C of internal and terminal alkynes and/or 1,3-diynes gives such a unique opportunity and has herein been described for the first time.

**Figure 4:**
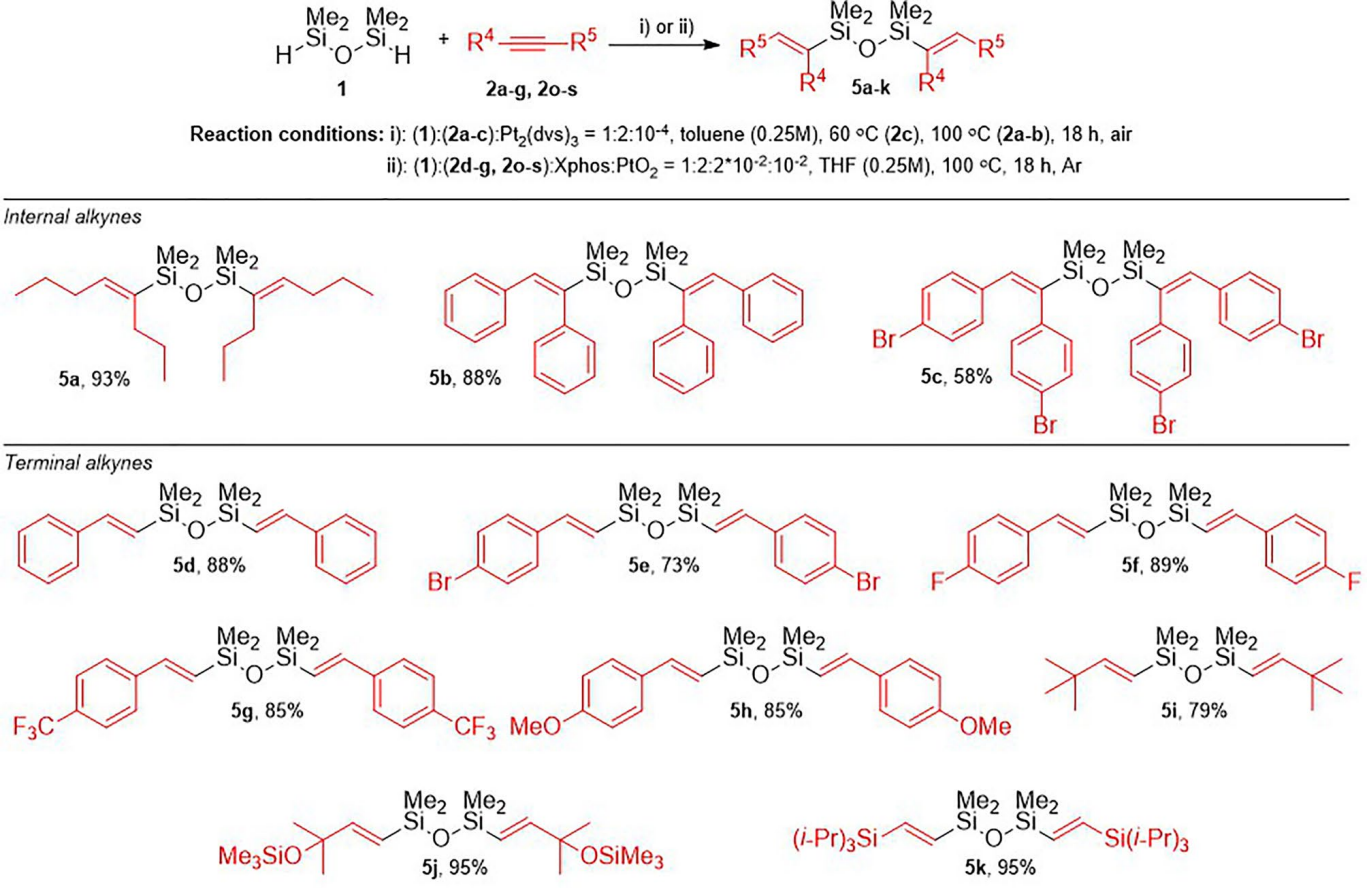
The synthesis of symmetrical disiloxanes **5a‒k** via the hydrosilylation of internal and terminal alkynes with **1**.

In the last stage of our study, we performed the synthesis of symmetrically monoalkenyl-substituted disiloxanes (Fig. 4). This class of compounds can easily be prepared through the reaction of monofunctionalized siloxane **3a**‒**c** with the corresponding alkyne **2a**‒**c** in the presence of Karstedt’s catalyst or directly from the modification of both Si-H bonds with the application of 2 equiv. of alkyne **2** over disiloxane **1**. The hydrosilylation of internal alkynes **2a**‒**c** with disiloxane **1** selectively gave the *cis*-addition products **5a**‒**c** with isolation yields from good to excellent. Products **5a** and **5c** were obtained selectively, isolated, and characterized for the first time. Product **5b** was also synthesized with a high yield using the heterogenous system PtO/PtO_2_-Fe_3_O_4_^31^.

**Figure 5:**
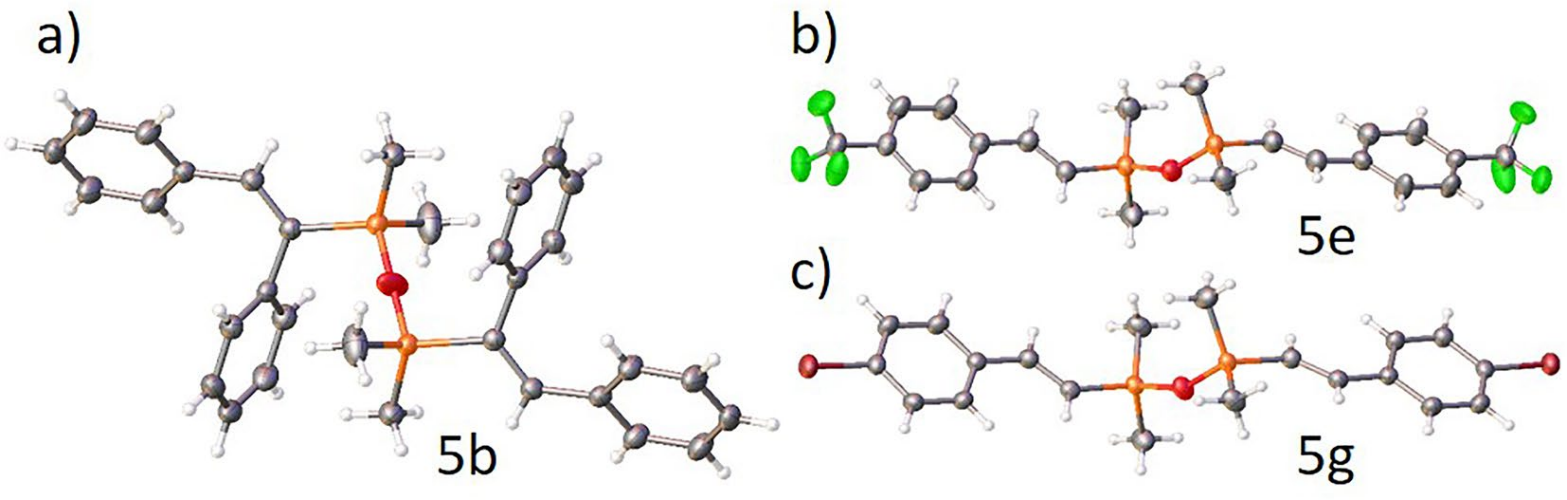
The molecular structure of compounds **5b** (**a**), **5e** (**b**), and **5g** (**c**). Displacement ellipsoids are shown at the 50% probability level. Selected geometrical parameters are summarized in Table S2 in ESI.

Moreover, for compound **5b**, the crystal structure was confirmed by single-crystal X-ray diffraction (Fig. 5a). To the best of our knowledge, this is the first example of the crystal structure of disiloxane **5** with two disubstituted alkenyl moieties.

Subsequently, we investigated the functionalization of terminal alkynes with **1**. Similar to the hydrosilylation of terminal alkynes with **3a**, the reactions were performed in the presence of the in situ generated PtO_2_/Xphos catalytic system, which selectively led to the formation of *trans*-isomers in the *cis*-addition of the SiH bond to the C≡C bond. The addition of Si–H bonds to 2 equiv. of phenylacetylene **2d** gave product **5d**, as expected. The same regio- and stereoselectivity was observed for the hydrosilylation of phenylacetylene derivatives substituted with electron-withdrawing (–Br (**2d**), –F (**2o**), -CF_3_ (**2p**)) or electron-donating (–OMe (**2r**)) groups. Products **5e**‒**h** were easily isolated with high yields, and the process occurred with excellent selectivity (97% or greater) of β-(*E*)-isomers. The synthesis of symmetrically alkenyl-substituted disiloxanes was also applicable for sterically hindered terminal alkynes such as 3,3-dimethylbut-1-yne (**2f**), trimethyl((2-methylbut-3-yn-2-yl)oxy)silane (**2g**), and ethynyltri*iso*propylsilane (**2s**). Products **5j** and **5k** could be obtained in almost quantitative yields. Disiloxanes **5a, c, g**, **j,** and** k** were obtained and fully characterized for the first time. On the other hand, compounds **5b**, **d**‒**f**, **h**‒**i**, and analogue symmetrical systems were synthesized using various approaches^19,21,22,26–28,31,44^. It is worth emphasizing that compounds **5e** and **5g**, were characterized by single crystal X-ray diffraction (Fig. 5b,c) and their crystal structures are the only representatives of such a family of compounds.

**Figure 6:**
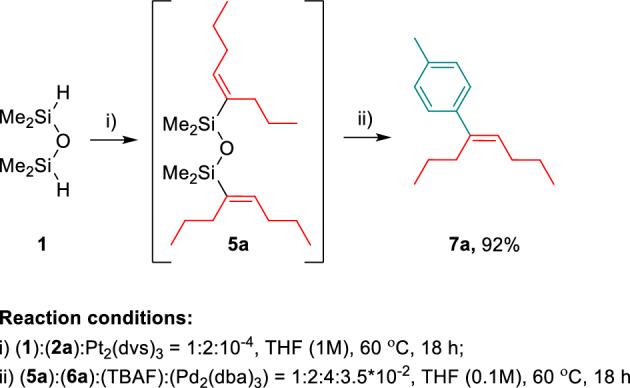
Synthesis of **7a** by the one-pot hydrosilylation and Hiyama reactions, starting from disiloxane **1**.

Finally, to prove that alkenyl-functionalized disiloxanes **4** and **5** constitute potentially useful building blocks in the formation of new carbon-carbon bonds, a Pd-catalyzed Hiyama coupling of **5a** with 4-iodotoluene **6a** was carried out. The cross-coupling product **7a** was obtained with 92% of the isolated yield. This result encouraged us to improve the 2-step, sequential method and perform hydrosilylation and Hiyama reactions one after the other, in the one-pot manner (Fig. 6). Such an approach led directly to product **7a** with excellent selectivity (> 99%) and the same high isolation yield (92%) as was observed for the sequential processes.

## Conclusion

In summary, we synthesized novel bifunctional disiloxanes via the subsequent hydrosilylation of internal alkynes in the first step and alkynes (terminal or internal) or 1,3-diynes in the second. As a result, unsymmetrical bisfunctionalized disiloxanes with two disubstituted (symmetrical (**4a**–**b**) and unsymmetrical (**4h**–**n**)) or one di- and one monosubstituted (**4c**–**g**) alkenyl groups were obtained which became a completely new family of compounds unavailable through other reported methods.

At the same time, hydrosilylation of two equivalents of the same alkyne (internal or terminal) led to a known type of symmetrical siloxanes with two mono- (**5d**–**k**) or disubstituted (**5a**–**c**) alkenyl functionalities. Even though analogues systems were obtained by other methods, hydrosilylation was proved to be the most versatile, straightforward, and tolerant for many functional groups, based on commercially available reagents and catalysts and an easily accessible method. Only with the use of hydrosilylation process all types of disiloxanes (unsymmetrical and symmetrical) with mono- and disubstituted alkenyl groups could be achieved.

Therefore, by the developed method 27 compounds were obtained, 21 of which were described for the first time (**3a‒c, 4a‒n, 5a, c, g, j, k**). Crystal structures for compounds **5b**, **5e**, and **5g** were determined for the first time and represent the only examples of such systems.

Finally, disiloxane **5a** was reacted with 4-iodotoluene in the presence of Pd_2_(dba)_3_ and TBAF, and gave the product of cross-coupling **7a** in high yield. The high efficiency of both processes (hydrosilylation and Hiyama coupling) allowed the development of the one-pot method of **7a** synthesis. This example proved the concept that obtained compounds can find potential applications as building blocks or coupling agents, or reagents in many powerful transformations such as Heck, Suzuki, and Hiyama couplings.

## Methods

### Synthesis of monofunctionalized disiloxanes 3a‒c by the hydrosilylation of internal alkynes 2a‒c with 1,1,3,3-tetramethyldisiloxane (1)

Alkyne (**2a‒c**) (0.5 mmol) and **1** (5 mmol) were placed in a 100 mL round-bottom flask equipped with a stirring bar containing 2 mL of toluene (0.25M). Reaction for **3a** was performed without solvent. Subsequently, the reaction mixture was stirred at room temperature and Karstedt’s catalyst (5 × 10^−5^ mmol of Pt) was added. All volatiles were removed under vacuum from the post-reaction mixture after 18 h for **3a‒b** and **3h** for **3c**. The residue was dissolved in *n*-hexane and filtered through a pad of silica using TLC to control the separation process. Compounds were characterized by GC-MS, FT-IR and ^1^H, ^13^C and ^29^Si NMR and elemental analyses. The gram-scale synthesis of **3a** and **3c** was performed in the same manner with the application of 50 mmol of **1** and 5 mmol of alkynes **2a** and **2c**, respectively.

### Synthesis of unsymmetric disiloxanes (4a‒b, 4h‒n) by the hydrosilylation of internal alkynes (2a‒c) or 1,3-diynes (2h‒n) with monofunctionalized disiloxanes (3a‒c)

Alkyne (**2a‒c**) or 1,3-diyne (**2h‒n)** (0.5 mmol) and monofunctionalized disiloxane (**3a‒c**) (0.5 mmol) were placed in a 50 mL round-bottom flask equipped with stirring bar containing 2 mL of dry toluene. Subsequently, the reaction mixture was heated up to 100 °C and Karstedt’s catalyst (5 × 10^−4^ mmol of Pt_2_(dvs)_3_) was added. After 18 h post-reaction mixture was cooled down and all volatiles were removed under a vacuum. Products were purified by flash chromatography and characterized by GC-MS, FT-IR and ^1^H, ^13^C, ^29^Si NMR and elemental analyzes.

### Synthesis of unsymmetric disiloxanes (4c‒f) by the hydrosilylation of terminal alkynes (2c‒f) with monofunctionalized disiloxane (3a)

Platinum(IV) oxide (0.005 mmol) and Xphos (0.01 mmol) were placed in a 25 mL Schlenk vessel equipped with a stirring bar under an argon atmosphere. 2 mL of dry tetrahydrofuran was added and the reaction mixture was heated up to 60 °C and stirred for 1 hour. Subsequently, terminal alkyne **2** (0.5 mmol) and **3** (0.5 mmol) were added and heated up to 100 °C. After 18 h post-reaction mixture was cooled down, and filtered through a syringe filter and all volatiles were removed under a vacuum. flash chromatography was performed and products were characterized by GC-MS, FT-IR and ^1^H, ^13^C, ^29^Si NMR and elemental analyzes.

### Synthesis of unsymmetric disiloxanes (4a, 4m) by the subsequent, one-pot bishydrosilylation of alkyne 2c and alkyne 2a with 1,1,3,3-tetramethyldisiloxane (1) as well as alkyne 2a and 1,3-diyne (2m) with 1,1,3,3-tetramethyldisiloxane (1)

Alkyne (**2c** for **4a** and **2a** for **4m**) (0.5 mmol) and **1** (5 mmol) were placed in a 100 mL round-bottom flask equipped with a stirring bar containing 2 mL of toluene (0.25M). Reaction with **2a** was performed without solvent Subsequently, the reaction mixture was stirred at room temperature and Karstedt’s catalyst (5 × 10^−4^ mmol of Pt_2_(dvs)_3_) was added. All volatiles were removed under vacuum from the post-reaction mixture after 3 h or 18 h for **2c** and **2a**, respectively. Afterwards, 2 mL of toluene and appropriate alkyne or 1,3-diyne (0.5 mmol) (**2a** for **4a** and **2m** for **4m**) were added and heated over 18 h at 100 °C. Obtained *via* one-pot protocol products were purified and characterized analogously like for the stepwise procedure.

### Synthesis of symmetric disiloxanes (5a‒c) by the hydrosilylation of internal alkynes (2a‒c) with 1,1,3,3-tetramethyldisiloxane (1)

To the 100 mL, round-bottom flask equipped with a stirring bar and 2 mL of toluene, the internal alkynes (**2a‒c**) (1 mmol) and 1,1,3,3-tetramethyldisiloxane (**1**) (0.5 mmol) were added and heated up to 60 °C (**2c**) or 100 °C (**2a-b**).

Subsequently, Karstedt’s catalyst (5 × 10^−4^ mmol of Pt_2_(dvs)_3_) was added. After 18 h, the reaction mixture was cooled down and all volatiles were removed under a vacuum. Products were purified by flash chromatography and characterized by GC–MS, FT–IR and ^1^H, ^13^C, ^29^Si NMR and elemental analyzes.

### Synthesis of symmetric disiloxanes (5d‒k) by the hydrosilylation of terminal alkynes (2c‒f, 2o‒s) with 1,1,3,3-tetramethyldisiloxane (1)

Platinum(IV) oxide (0.005 mmol) and Xphos (0.01 mmol) were placed in a 25 mL Schlenk vessel equipped with a stirring bar under an argon atmosphere. 2 mL of dry tetrahydrofuran was added and the reaction mixture was heated up to 60 °C and stirred for 1 hour. Subsequently, terminal alkyne **2** (0.5 mmol) and **1** (0.25 mmol) were added and heated up to 100 °C. After 18 h, the post-reaction mixture was cooled down, filtered through a syringe filter and all volatiles were removed under a vacuum. flash chromatography was performed and products were characterized by GC–MS, FT-IR and ^1^H, ^13^C, ^29^Si NMR and elemental analyzes.

### One-pot synthesis of 7a via hydrosilylation and Hiyama reactions

To a Schlenk flask with a Rotaflo^®^ stopcock equipped with a magnetic stirring bar the 1,1,3,3-tetramethyldisiloxane (**1**, 1 equiv.) and 4-octyne (**2c**, 2 equiv.) were added and the mixture was purged with argon for 5 min after which THF (1M) and Karstedt’s catalyst (10^−4^ mol of Pt per mol of **1**) were added. The mixture was stirred at 60 °C for 18 h. Subsequently, the 4-iodotoluene (**6a**, 2 equiv.) was added, followed by the addition of TBAF (4 equiv. 1M in THF) and Pd_2_(dba)_3_ (3.5 × 10^−2^ mol) dissolved in THF (0.11M). The obtained dark brown solution was stirred at 60 °C for 18 h. The mixture was diluted with dichloromethane, passed through a pad of Celite and concentrated. The residue was purified by flash column chromatography to afford the product **7a** with 92% of yield.

## Supplementary Information

Supplementary Information.

## Data Availability

All data generated or analyzed during this study are included in this published article and its supplementary information file.
