# Supplementary material for: Synthesis of unsymmetrically and symmetrically functionalized disiloxanes via subsequent hydrosilylation of C≡C bonds
Source: Sci Rep. 2023 Jun 23;13:10244. doi: 10.1038/s41598-023-37375-8 (PMC10290067; doi:10.1038/s41598-023-37375-8)
Supplement: Supplementary file 1 — Supplementary Information. [file 41598_2023_37375_MOESM1_ESM.pdf]

Supporting Information

Synthesis of unsymmetrically and symmetrically functionalized disiloxanes via subsequent hydrosilylation of C≡C bonds

Jakub Szyling[1], Jędrzej Walkowiak[1], Agnieszka Czapik[2], and Adrian Franczyk[1,]*

[1] Center for Advanced Technology, Adam Mickiewicz University, Uniwersytetu Poznańskiego 10, 61-614 Poznan, Poland.

[2] Faculty of Chemistry, Adam Mickiewicz University, Uniwersytetu Poznańskiego 10, 61-614 Poznan

* Correspondence: adrian.franczyk@amu.edu.pl

Outline

1. General information S2

1.1. NMR analyzes S2

1.2. GC-MS analysis S2

1.3. ESI-HRMS analysis S2

1.4. FT-IR analysis S2

1.5. Elemental analysis S2

1.6. Melting point analysis S2

1.7. Single crystal X-ray diffraction S2

1.8. Products purification S3

2. Materials S3

3. General procedures S3

3.1 Synthesis of alkynyl bromides S3

3.2. Synthesis of symmetrical 1,3-diynes S4

3.3. Synthesis of unsymmetrical 1,3-diynes S4

4. Products characterization S5

5 NMR spectra. S17

6. HRMS spectra. S65

7. Single crystal X-ray diffraction data S69

8. References S74

General information:

1.1. NMR analyzes

^{1}H, ^{13}C, and ^{29}Si NMR spectra were recorded at 25 °C on Bruker UltraShield 300, Bruker Ascend 400 or 600 MHz. The 1D selective gradient NOESY experiments were performed on Bruker Ascend 400 MHz and characteristic/isolated signals were selectively irradiated. The 2D HSQC experiments were performed on Bruker Ascend 400 MHz. Chemical shifts were reported in ppm with the reference to the residue portion solvent peak. Chloroform-d_1 and benzene-d_6 were used as solvents and for internal deuterium lock. The multiplicities were reported as follows: singlet (s), doublet (d), doublet of doublets (dd), multiplet (m), triplet (t), pentet (p), doublet of doublets of triplets (ddt).

1.2. GC-MS analysis

The mass spectra of the products were obtained by GC-MS analysis on a Bruker Scion 436-GC with a 30m Varian DB-5 0.25mm capillary column and a Scion SQ-MS mass spectrometry detector. Two temperature programs were used a) 60 °C (3 min), 10°C/min, 250 °C (30 min), b) 100 °C (3 min), 10°C/min, 280 °C (44.5 min).

1.3. ESI-HRMS analysis

High-resolution mass spectra (HRMS) were obtained using an Impact HD mass spectrometer (Q-TOF type instrument equipped with an electrospray ion source; Bruker Daltonics, Germany). The sample solutions (DCM:MeOH) were infused into the ESI source by a syringe pump (direct inlet) at the flow rate of 3 μL/min. The instrument was operated under the following optimized settings: end plate voltage 500 V; capillary voltage 4.2 kV; nebulizer pressure 0.3 bar; dry gas (nitrogen) temperature 200 °C; dry gas flow rate 4 L/min. The spectrometer was previously calibrated with the standard tune mixture.

1.4. FT-IR analysis

FT-IR spectra were measured on a Nicolet iS50 FT-IR spectrometer (Thermo Scientific) equipped with a built-in ATR accessory with an ATR diamond unit. In all experiments, 16 scans at a resolution of 2 cm^{-1} were performed.

1.5. Elemental analysis

Elemental analyses were performed using a Vario EL III instrument.

1.6. Melting point analysis

Melting point were determined by Buchi® Melting Point M-565.

1.7. Single crystal X-ray diffraction

Single crystals of **5b**, **5e** and **5g** suitable for X-ray structural analysis were obtained by slow evaporation of solvent. The diffraction data were collected at 130 K with an Oxford Diffraction SuperNova diffractometer using Cu Kα radiation (λ = 1.54184 Å) equipped with mirror monochromator. The intensity data were collected and processed using CrysAlis PRO software.[1] The structures were solved by direct methods with the program SHELXT 2018/2[2] and refined by full-matrix least-squares method on F^2 with SHELXL 2018/3.[3] The carbon-bound hydrogen atoms were refined as riding on their carriers and their displacement parameters were set equal to 1.5Ueq(C) for the methyl groups and 1.2Ueq(C) for the remaining H atoms. A summary of the crystallographic data is given in Table S1 and selected geometrical data are juxtaposed in Table S2. Molecular graphics were generated with Olex2[4] and Mercury 2022.2.0 software.[5]

1.7. Products purification

The UV-absorbing products were purified on silica by flash chromatography (Biotage IsoleraOne chromatograph) with a UV detector (λ_1 = 255 nm, λ_2= 280 nm). Purification details: cartridge 10 g, flow rate: 12 mL/min, length: 10 CV (CV = column volume), phase: hexane/dichloromethane (step 1: hexane 100% by 4 CV, step 2: gradient from 0% up to 50% of dichloromethane by 4 CV, step 3: hexane 50% by 2 CV). The non-aromatic products were purified on silica using standard column chromatography with hexane/dichloromethane (95/5–70/30) as eluents. Products were characterized by GC-MS, ^{1}H, ^{13}C, ^{29}Si NMR, and FT-IR analyses.

The non-absorbing products were purified based on gravity column chromatography using hexane and dichloromethane as eluents (initial phase: hexane - 100%, final phase: hexane/dichloromethane = 50%/50%.).

2. Materials

1,1,3,3-Tetramethyldisiloxane (97%, acbr), 1,2-diphenylacetylene (98%, Sigma-Aldrich), bis(4-bromophenyl)acetylene (97%, Acros Organics), 4-octyne (99%, Sigma-Aldrich), phenylacetylene (98%, Sigma-Aldrich), 1-bromo-4-ethynylbenzene (97%, Sigma-Aldrich), 1-fluoro-4-ethynylbenzene (97%, Sigma-Aldrich), 1-ethynyl-4-(trifluoromethyl)benzene (97%, Sigma-Aldrich), 1-ethynyl-4-methoxybenzene (97%, Sigma-Aldrich), 1-octyne (97%, Sigma-Aldrich), 3-ethynylthiophene (96%, Sigma-Aldrich), [(1,1-dimethyl-2-propynyl)oxy]trimethylsilane (98%, Sigma-Aldrich), ethynylcyclohexane (98%, Sigma-Aldrich),), 3,3-dimethylbut-1-yne (98%, Sigma-Aldrich), hexa-2,4-diyne (99%, abcr), 1,4-bis(trimethylsilyl)buta-1,3-diyne (98%, Sigma-Aldrich), 1,4--diphenylbuta-1,3-diyne (98%, Sigma-Aldrich), triethylamine (99%, Sigma-Aldrich), (tri*iso*propylsilyl)acetylene (97%, Sigma-Aldrich), N-bromosuccinimide (98%, Sigma-Aldrich), hydroxylamine hydrochloride (98%, abcr), tributylamine (99%, Sigma-Aldrich), piperidine (99%, TCI), ammonium chloride (99%, Avantor Performance Materials Poland), platinum(0)-1,3-divinyl-1,1,3,3-tetramethyldisiloxane (Karstedt's catalyst, solution in xylene, Pt~2%, Sigma-Aldrich), platinum(IV) oxide (99%, Sigma-Aldrich), 2-dicyclohexylphosphino-2′,4′,6′-tri*iso*propylbiphenyl (XPhos; 97%, Sigma-Aldrich) were used as received. Deuterium solvents were obtained from Dr. Glaser AG Basel. The *n*-hexane (99%, POCH Basic) and dichloromethane (99%, POCH Basic) were used as received. Toluene (99%, POCH Basic) and tetrahydrofuran (99%, POCH Basic), used in the reactions, were distilled and stored under argon prior to use.

3. General procedures

3.1. Synthesis of alkynyl bromides

The alkynyl bromides were prepared according to the literature with some modification[6]:

To the one-neck round bottom flask containing a solution of alkyne (5 mmol) in acetone (50 mL), N-bromosuccinimide (6 mmol) and silver nitrate (0.5 mmol) were successively added. The reaction mixture was stirred without light access at room temperature over 18 h before adding water (100 mL). The resulting mixture was extracted with *n*-hexane (3 x 100 mL) and the combined organic layers were washed with brine (100 mL), dried over $MgSO_4$, filtered through a pad of silica, and concentrated to give colourless or slightly yellow liquids.

Caution: All synthesized alkynyl bromides are strong lachrymators. The isolation should be performed under the hood.

3.2. Synthesis of symmetrical 1,3-diynes

The CuCl (0.1 mmol) was placed in a round bottom bulb equipped with a condenser and magnetic stirring bar. Subsequently, toluene (10 mL), piperidine (0.15 mmol), and alkyne (5 mmol) were placed in the reaction vessel. The reaction was performed at 80 °C for 18 hours in an air atmosphere. Afterward, the reaction mixture was cooled and all volatiles were removed under vacuum. The crude residue was dissolved in hexanes (with a small amount of dichloromethane if necessary), purified, and analyzed with NMR and GC-MS analyzes.

3.3. Synthesis of unsymmetrical 1,3-diynes

The unsymmetrical 1,3-diynes were prepared according to the literature[7] with some modifications:

CuCl was dissolved in a 2:3 mixture by volume of *n*-$BuNH_2$:H_2O (5 mL/mmol alkyne) and the solution was cooled to 0 °C in an ice bath. Hydroxylamine hydrochloride was slowly added until trace amounts of copper(II) were reduced and the color of the solution changed from blue to colorless. The alkyne bromide and alkyne were dissolved in dichloromethane (5 mL/mmol alkyne), and this solution was added to the reaction flask at once. The biphasic mixture was vigorously stirred overnight under an argon atmosphere. Subsequently, the organic layer was removed and washed with portions of saturated aq. NH_4Cl until these portions no longer took on a blue color. The organic layer was dried ($MgSO_4$) and concentrated by rotary evaporation. The crude residue was dissolved in *n*-hexane, purified, analyzed with NMR and GC-MS analyzes.

4. Products characterization

1-Bromooct-1-yne

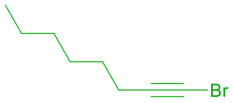

Chemical Formula: $C_8H_{13}Br$
Molecular Weight: 189,10

^{1}H NMR (300 MHz, $CDCl_3$, δ, ppm): 2.20 (t, $J_{H\text{-}H}$ = 7.0 Hz, 2H, $C\underline{H}_2C{\equiv}C$), 1.51 (p, $J_{H\text{-}H}$ = 6.8 Hz, 2H), 1.38 – 1.24 (m, 6H), 0.89 (t, $J_{H\text{-}H}$ = 6.8 Hz, 3H, $CH_2C\underline{H}_3$). **GC-MS** (EI, m/z): 161(M^+-28 ($C_2H_5^\bullet$), 4), 159(M^+-30($C_2H_5^\bullet$), 4), 147(4), 145(4), 132(7), 119(8), 117(9), 109(15), 79(41), 67(100). Colorless liquid. Isolated yield: 95%. Analytical data are in agreement with the literature.[8]

(Bromoethynyl)triisopropylsilane

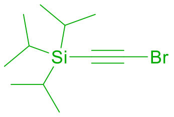

Chemical Formula: $C_{11}H_{21}BrSi$
Molecular Weight: 261,28

^{1}H NMR (300 MHz, $CDCl_3$, δ, ppm): 1.08 (s, 21H, $(Si(C\underline{H}(C\underline{H}_3)_2)_3)$. **GC-MS** (EI, m/z): 262 (M^++2, 5) 260 (M^+, 5), 219(83), 217(83), 191(43), 189(42), 163(74), 161(68), 149(100), 147(93), 137(22), 109(31), 95(23), 69(17), 53(22). Colorless liquid. Isolated yield: 90%. Analytical data are in agreement with the literature.[9]

1,4-Di(thiophen-3-yl)buta-1,3-diyne (**2k**)

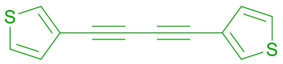

Chemical Formula: $C_{12}H_6S_2$
Molecular Weight: 214,30

^{1}H NMR (300 MHz, $CDCl_3$, δ, ppm): 7.64 – 7.56 (m, 2H), 7.32 – 7.26 (m, 2H), 7.22 – 7.14 (m, 2H). **^{13}C NMR** (75 MHz, $CDCl_3$, δ, ppm): 131.39, 130.31, 125.75, 121.03, 73.64 (C≡C). **GC-MS** (EI, m/z): 214(M^+, 100), 169(24), 144(7), 117(10), 93(9), 69(9). Brown solid. Isolated yield: 78%. Analytical data are in agreement with the literature.[10]

(Phenylbuta-1,3-diyn-1-yl)triisopropylsilane (**2m**)

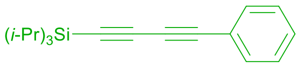

Chemical Formula: $C_{19}H_{26}Si$
Molecular Weight: 282,50

^{1}H NMR (300 MHz, $CDCl_3$, δ, ppm): 7.55 – 7.27 (m, 5H, Ph), 1.12 (s, 21H, $Si(C\underline{H}(C\underline{H}_3)_2)_3$). **^{13}C NMR** (75 MHz, $CDCl_3$, δ, ppm): 132.84, 129.36, 128.54, 121.68, 89.63 (C≡C), 88.03 (C≡C), 75.69 (C≡C), 74.80 (C≡C), 18.72, 11.45. **^{29}Si NMR** (79 MHz, $CDCl_3$ δ, ppm): -0.65. **GC-MS** (EI, m/z): 282(M^+, 8), 239(98), 211(44), 197(40), 183(54), 169(100), 159(21), 153(27), 91(20) 59(10). **FT-IR** (cm^{-1}): 2942, 2890, 2865, 2204, 2101, 1488, 1461, 1070, 1018, 995, 881, 752, 729, 675, 602. Colorless oil. Isolated yield: 75%. Analytical data are in agreement with the literature.[11]

2,2-Dimethyldodeca-3,5-diyne (**2n**)

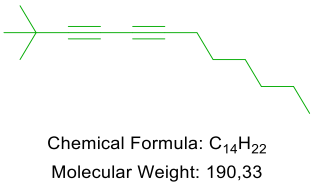

^{1}H NMR (300 MHz, $CDCl_3$, δ, ppm): 2.25 (d, $J_{H\text{-}H}$ = 7.0 Hz, 2H, ≡CCH_2), 1.59 – 1.24 (m, 8H), 1.23 (s, 9H, $C(H_3)_3$), 0.88 (t, $J_{H\text{-}H}$ = 6.8 Hz, 3H, CH_2CH_3). **^{13}C NMR** (75 MHz, $CDCl_3$, δ, ppm): 85.22 (C≡C), 78.99 (C≡C), 65.12 (C≡C), 64.04 (C≡C), 31.46, 30.77, 28.70, 28.47, 28.06, 22.66, 19.39, 14.19. **GC-MS** (EI, m/z): 175(M^+-15, 20), 161(20), 147(19), 133(), 119(70), 105(100), 91(67), 79(38), 67(22), 55(25). **FT-IR** (cm^{-1}): 2966, 2930, 2861, 1706, 1456, 1363, 1281, 1201, 1169. Yellow oil. Isolated yield: 89%.

*(E)-1-(1,2-Diphenylvinyl)-1,1,3,3-tetramethyldisiloxane (**3a**)*

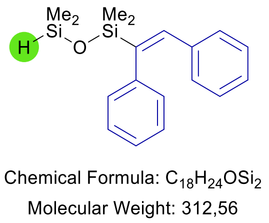

^{1}H NMR (300 MHz, $CDCl_3$, δ, ppm): 7.41 – 7.02 (m, 10H, Ph), 6.99 (s, 1H, C=CH), 4.83 (hept, $J_{H\text{-}H}$ = 2.9 Hz, 1H), 0.29 (s, 6H, $Si(CH_3)_2$), 0.25 (s, 2H, $Si(CH_3)_2$), 0.24 (s, 3H, $Si(CH_3)_2$). **^{13}C NMR** (101 MHz, $CDCl_3$, δ, ppm): 146.12, 141.99, 138.14, 137.30, 129.76, 128.82, 128.77, 128.06, 127.86, 127.83, 127.35, 126.66, 125.94, 0.99 $Si(CH_3)_2$), 0.04 $Si(CH_3)_2$). **^{29}Si NMR** (79 MHz, $CDCl_3$ δ, ppm): -1.91, -5.62. **GC-MS** (EI, m/z): 312(M^+, 43), 297(12), 219(100), 193(18), 117(71), 73(48). **FT-IR** (cm^{-1}): 691, 780, 900, 958, 1049, 1251, 1493, 1600, 2120, 2957, 3022, 3086. **Elemental Anal.** for $C_{18}H_{24}OSi_2$ (%): calcd.: C, 69.17; H, 7.74; found: C, 69.33; H, 7.81. Colorless liquid. Isolated yield: 88% (91% gram-scale).

*(E)-1-(1,2-Bis(4-bromophenyl)vinyl)-1,1,3,3-tetramethyldisiloxane (**3b**)*

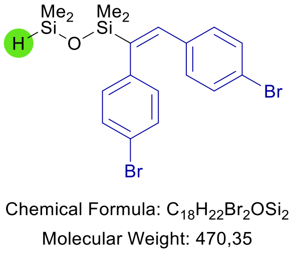

^{1}H NMR (400 MHz, $CDCl_3$, δ, ppm): 7.70 – 7.64 (m, 2H, Ph), 7.53 – 7.46 (m, 2H, Ph), 7.17 – 7.10 (m, 2H, Ph), 7.08 (m, 3H, Ph, C=CH), 4.95 (p, $J_{H\text{-}H}$ = 2.8 Hz, 1H, Si-H), 0.44 (s, 6H, $Si(CH_3)_2$)), 0.41 (s, 3H, $Si(CH_3)_2$)), 0.41 (s, 3H, $Si(CH_3)_2$)). **^{13}C NMR** (101 MHz, $CDCl_3$, δ, ppm): 145.87 (C=C), 140.54 (C=C), 137.40, 135.83, 132.05, 132.02, 131.34, 131.19, 129.56, 129.53, 128.16, 121.55, 120.14, 0.95 $Si(CH_3)_2$), -0.06 $Si(CH_3)_2$). **^{29}Si NMR** (79 MHz, $CDCl_3$ δ, ppm): -1.99, -5.15. **GC-MS** (EI, m/z): 470(M^+, 3), 454(3), 390(7), 388(7), 298(15), 233(15), 207(25), 176(15), 133(100), 117(14), 73(40). **FT-IR** (cm^{-1}): 653, 699, 723, 880, 958, 1061, 1250, 1498, 1615, 2124, 2951, 3023, 3091. **Elemental Anal.** for $C_{18}H_{22}Br_2OSi_2$ (%): calcd.: C, 45.97; H, 4.71; found: C, 46.05; H, 4.75. Colorless oil. Isolated yield: 77%.

*(E)-1,1,3,3-Tetramethyl-1-(oct-4-en-4-yl)disiloxane (**3c**)*

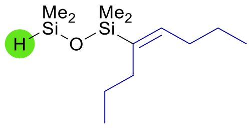

Chemical Formula: $C_{12}H_{28}OSi_2$
Molecular Weight: 244,53

^{1}H NMR (300 MHz, $CDCl_3$, δ, ppm): 5.93 – 5.71 (m, 1H, C=C$\underline{H}$), 4.72 (hept, J_{H-H} = 2.7 Hz, 1H, Si-$\underline{H}$), 2.16 – 2.02 (m, 4H), 1.48 – 1.30 (m, 4H), 0.92 (m, 6H), 0.18 (s, 3H, $Si(CH_3)_2$), 0.17 (s, 3H, $Si(CH_3)_2$), 0.14 (s, 6H, $Si(CH_3)_2$). **^{13}C NMR** (101 MHz, $CDCl_3$, δ, ppm): 141.29 (C=C), 140.87 (C=C), 31.48, 30.54, 23.50, 22.83, 14.62, 14.07, 1.03 Si($\underline{C}H_3)_2$), 0.58 Si($\underline{C}H_3)_2$). **^{29}Si NMR** (79 MHz, $CDCl_3$ δ, ppm): -0.48, -6.90. **GC-MS** (EI, m/z): 244(M^+, 1), 229(3), 201(5), 173(10), 159(6), 133(100), 119(37), 73(17). **FT-IR** (cm^{-1}): 701, 789, 1019, 1257, 1613, 2109, 2872, 2930, 2959. **Elemental Anal.** for $C_{12}H_{28}OSi_2$ (%): calcd.: C, 58.94; H, 11.54; found: C 59.04; H, 11.62. Colorless liquid. Isolated yield: 92% (93% gram-scale). Despite previous study, the compound **3c** was isolated and characterized for the first time.[12]

*1-((E)-1,2-Diphenylvinyl)-1,1,3,3-tetramethyl-3-((E)-oct-4-en-4-yl)disiloxane (**4a**)*

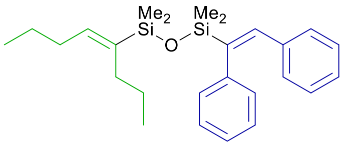

Chemical Formula: $C_{26}H_{38}OSi_2$
Molecular Weight: 422,76

^{1}H NMR (400 MHz, $CDCl_3$, δ, ppm): 7.27 – 7.04 (m, 4H, Ph), 6.99 – 6.83 (m, 6H, Ph), 6.79 (s, 1H, C=C$\underline{H}$Ph), 5.75 – 5.63 (m, 1H, C=C$\underline{H}CH_2$), 2.05 – 1.86 (m, 4H,), 1.32 – 1.08 (m, 4H), 0.86 – 0.70 (m, 5H), 0.05 (s, 6H, Si(C$\underline{H}_3)_2$), 0.00 (s, 6H, Si(C$\underline{H}_3)_2$). **^{13}C NMR** (101 MHz, $CDCl_3$, δ, ppm): 146.62 (C=C), 142.21, 141.25, 140.99, 137.86, 137.47, 131.76, 129.75, 129.71, 128.83, 128.69, 128.49, 128.06, 128.02, 127.85, 127.21, 125.81, 77.48, 77.16, 76.84, 31.50, 30.54, 23.53, 22.83, 14.66, 14.10, 0.91 Si($\underline{C}H_3)_2$), 0.33 Si($\underline{C}H_3)_2$). **^{29}Si NMR** (79 MHz, $CDCl_3$ δ, ppm): -1.42, -4.03. **GC-MS** (EI, m/z): 422(M^+, 10), 311(11), 295(64), 242(31), 193(22), 180(23), 133(100), 73(20). **FT-IR** (cm^{-1}): 699, 783, 958, 1054, 1253, 1493, 1601, 2871, 2930, 2957 3022, 3056. **Elemental Anal.** for $C_{26}H_{38}OSi_2$ (%): calcd.: C, 73.87; H, 9.06; found: C, 74.01; H, 9.15. Colorless liquid. Isolated yield: 70-72%.

*1-((E)-1,2-Bis(4-bromophenyl)vinyl)-3-((E)-1,2-diphenylvinyl)-1,1,3,3-tetramethyldisiloxane (**4b**)*

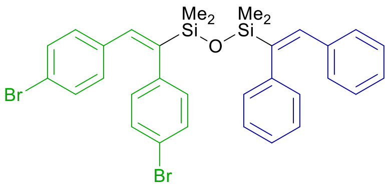

Chemical Formula: $C_{32}H_{32}Br_2OSi_2$
Molecular Weight: 648,59

^{1}H NMR (400 MHz, $CDCl_3$, δ, ppm): 7.73 – 7.67 (m, 2H, Ph), 7.61 – 7.47 (m, 5H, Ph), 7.41 – 7.08 (m, 13H), 0.47 (s, 6H, Si(C$\underline{H}_3)_2$), 0.49 (s, 6H, Si(C$\underline{H}_3)_2$). **^{13}C NMR** (101 MHz, $CDCl_3$, δ, ppm): 146.01, 141.89, 140.59, 138.26, 137.39, 137.16, 135.84, 132.05, 131.32, 131.20, 129.76, 129.74, 129.55, 128.81, 128.76, 128.10, 128.06, 128.04, 127.85, 127.82, 127.43, 126.01, 121.52, 120.13, 0.33 (Si($\underline{C}H_3)_2$), 0.21 (Si($\underline{C}H_3)_2$). **^{29}Si NMR** (79 MHz, $CDCl_3$ δ, ppm): -2.31, -2.89. **ESI-HRMS** (m/z) ($[M+Na]^+$, (%)): 671.0242. **FT-IR** (cm^{-1}): 495, 692, 781, 957,

1009, 1046, 1251, 1483, 1600, 2956, 3021, 3054. **Elemental Anal.** for $C_{32}H_{32}Br_2OSi_2$ (%): calcd.: C, 59.26; H, 4.97; found: C, 59.17; H, 5.03. White viscous oil. Isolated yield: 71-74%.

*1-((E)-1,2-Diphenylvinyl)-1,1,3,3-tetramethyl-3-((E)-styryl)disiloxane (**4c**)*

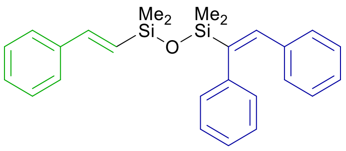

Chemical Formula: $C_{26}H_{30}OSi_2$
Molecular Weight: 414,70

^{1}H NMR (300 MHz, $CDCl_3$, δ, ppm): 7.60 – 7.43 (m, 2H, Ph), 7.37 – 7.24 (m, 6H, Ph), 7.16 – 7.12 (m, 5H, Ph), 7.06 – 6.92 (m, 4H), 6.46 (d, $J_{H\text{-}H}$ = 19.2 Hz, 1H, CH=CH), 0.28 (s, 12H, Si(CH_3)$_2$). **^{13}C NMR** (101 MHz, $CDCl_3$, δ, ppm): 146.38, 144.46, 142.07, 138.27, 138.05, 137.33, 129.75, 128.74, 128.65, 128.58, 128.31, 128.04, 127.86, 127.29, 126.70, 125.90, 0.92 (Si(CH_3)$_2$), 0.42 (Si(CH_3)$_2$). **^{29}Si NMR** (79 MHz, $CDCl_3$ δ, ppm): -2.00, -2.98. **GC-MS** (EI, m/z): 414(M^+, 20), 323(50), 295(37), 234(15), 219(27), 209(65), 193(100), 133(95), 116(21), 73(31). **FT-IR** (cm^{-1}): 690, 782, 957, 1028, 10431 1252, 1493, 1601, 2957, 3957. **Elemental Anal.** for $C_{26}H_{30}OSi_2$ (%): calcd.: C, 75.30; H, 7.29; found: C, 75.43; H, 7.33. Colorless liquid. Isolated yield: 81%.

*1-((E)-4-Bromostyryl)-3-((E)-1,2-diphenylvinyl)-1,1,3,3-tetramethyldisiloxane (**4d**)*

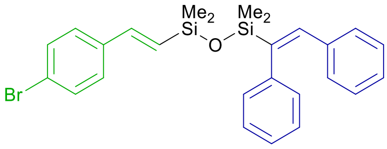

Chemical Formula: $C_{26}H_{29}BrOSi_2$
Molecular Weight: 493,59

^{1}H NMR (300 MHz, $CDCl_3$, δ, ppm): 7.56 – 7.45 (m, 2H, Ph), 7.36 – 7.27 (m, 4H, Ph), 7.27 – 7.01 (m, 8H, Ph) 6.98 (s, 1H, C=CH), 6.90 (d, $J_{H\text{-}H}$ = 19.2 Hz, 1H, CH=CH), 6.43 (d, $J_{H\text{-}H}$ = 19.2 Hz, 1H, CH=CH), 0.28 (s, 12H, Si(CH_3)$_3$). **^{13}C NMR** (101 MHz, $CDCl_3$, δ, ppm): 146.27, 143.11, 142.01, 138.11, 137.27, 137.20, 131.75, 129.73, 128.75, 128.21, 128.06, 127.85, 127.35, 125.93, 122.15, 0.88 (Si(CH_3)$_3$), 0.42 (Si(CH_3)$_3$). **^{29}Si NMR** (79 MHz, $CDCl_3$ δ, ppm): -2.14, -2.78. **GC-MS** (EI, m/z): 495((M+2)$^+$, 8), 493(M^+, 9), 414(18), 403(9), 401(7), 324(14), 322(15), 296(35), 233(24), 193(43), 134(100), 73(30). **FT-IR** (cm^{-1}): 692, 781, 830, 1045, 1251, 1485, 1602, 2965, 3055. **Elemental Anal.** for $C_{26}H_{29}BrOSi_2$ (%): calcd.: C, 63.27; H, 5.92; found: C, 63.45; H, 5.99. Colorless oil. Isolated yield: 75%.

*1-((E)-3,3-Dimethylbut-1-en-1-yl)-3-((E)-1,2-diphenylvinyl)-1,1,3,3-tetramethyldisiloxane (**4e**)*

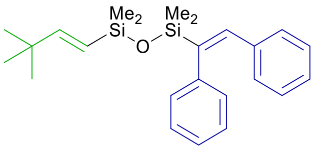

Chemical Formula: $C_{24}H_{34}OSi_2$
Molecular Weight: 394,71

^{1}H NMR (600 MHz, Benzene-d_6) δ 7.19-7.16 (m, 7H, Ph), 7.07 – 7.04 (m, 1H), 6.98 – 6.93 (m, 2H), 6.91 – 6.87 (m, 1H), 6.32 (d, J = 19.0 Hz, 1H, CH=CH), 5.74 (d, J = 19.0 Hz, 1H, CH=CH), 1.00 (s, 9H, C(CH_3)$_3$), 0.30 (s, 6H, Si(CH_3)$_2$)), 0.28 (s, 6H, Si(CH_3)$_2$)). **^{13}C NMR** (151 MHz, C_6D_6, δ, ppm): 158.87, 146.58, 142.53, 138.74, 137.74, 130.13, 129.05, 128.35, 126.24, 122.94, 35.07, 29.11, 1.07 Si(CH_3)$_2$), 0.53 Si(CH_3)$_2$). **^{29}Si NMR**

(79 MHz, $CDCl_3$ δ, ppm): -1.83, -3.42. **GC-MS** (EI, m/z): 394(M^+, 7), 337(14), 295(44), 215(10), 193(26), 147(25), 133(100), 117(18), 73(24). **FT-IR** (cm^{-1}): 699, 799, 1053, 1252, 1612, 2865, 2958, 3056. **Elemental Anal.** for $C_{24}H_{34}OSi_2$ (%): calcd.: C, 73.03; H, 8.68; found: C, 72.91; H, 8.61. Colorless oil. Isolated yield: 83%.

*1-((E)-1,2-Diphenylvinyl)-1,1,3,3-tetramethyl-3-((E)-3-methyl-3-((trimethylsilyl)oxy)but-1-en-1-yl)disiloxane (**4f**)*

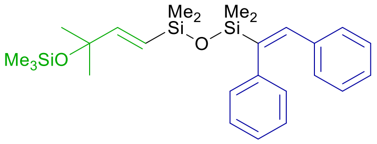

Chemical Formula: $C_{26}H_{40}O_2Si_3$
Molecular Weight: 468,86

GC-MS (EI, m/z): 468(M^+, 2), 454(1), 378(1), 312(29), 295(6), 221(100), 193(17), 148(27), 133(36). 73(50). Not isolated, converted into **4g**.

*(E)-4-(3-((E)-1,2-Diphenylvinyl)-1,1,3,3-tetramethyldisiloxaneyl)-2-methylbut-3-en-2-ol (**4g**)*

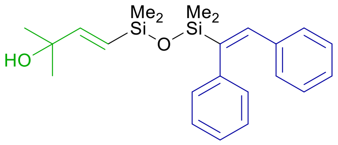

Chemical Formula: $C_{23}H_{32}O_2Si_2$
Molecular Weight: 396,68

^{1}H NMR (400 MHz, $CDCl_3$, δ, ppm): 7.40 – 7.23 (m, 3H, Ph), 7.16 – 6.99 (m, 7H), 6.94 (s, 1H, C=C<u>H</u>), 6.27 (d, J_{H-H} = 19.0 Hz, 1H), 5.80 (d, J_{H-H} = 19.0 Hz, 1H), 1.54 (br s, 1H, O<u>H</u>), 1.33 (s, 6H, C(C<u>H</u>3)2), 0.25 (s, 6H, Si(C<u>H</u>3)2), 0.20 (s, 6H, Si(C<u>H</u>3)2). **^{13}C NMR** (101 MHz, $CDCl_3$, δ, ppm): 154.25, 146.36, 142.09, 138.00, 137.33, 129.71, 128.72, 128.06, 127.88, 127.86, 127.32, 125.88, 124.15, 72.00 (<u>C</u>(CH3)2OH), 29.41 (C(<u>C</u>H3)2OH), 0.85 Si(<u>C</u>H3)2), 0.39 Si(<u>C</u>H3)2). **^{29}Si NMR** (79 MHz, $CDCl_3$ δ, ppm): -1.87, -3.17. **GC-MS** (EI, m/z): 396(M^+, 6), 279(8), 221(3), 193(8), 179(14), 149(70). 133(100), 118(11), 73(10). **FT-IR** (cm^{-1}): 962, 829, 957, 1043, 1251, 1601, 2899, 2691, 3022, 3366 (br). **Elemental Anal.** for $C_{23}H_{32}O_2Si_2$ (%): calcd.: C, 69.64; H, 8.13; found: C, 69.77; H, 8.09. Colorless liquid. Isolated yield: 68%.

*1-((E)-1,4-Bis(trimethylsilyl)but-1-en-3-yn-2-yl)-3-((E)-1,2-diphenylvinyl)-1,1,3,3-tetramethyldisiloxane (**4h**)*

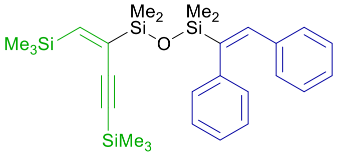

Chemical Formula: $C_{28}H_{42}OSi_4$
Molecular Weight: 506,98

^{1}H NMR (400 MHz, $CDCl_3$, δ, ppm): 7.35 – 7.25 (m, 2H, Ph), 7.24 – 7.16 (m, 1H, Ph), 7.12 – 7.06 (m, 3H), 7.07 – 7.00 (m, 2H, Ph), 7.01 – 6.94 (m, 2H, Ph), 6.90 (s, 1H, C=C<u>H</u>), 6.68 (s, 1H, C=C<u>H</u>), 0.21 (s, 12H, Si(C<u>H</u>3)2), 0.18 (s, 9H, Si(C<u>H</u>3)3), 0.16 (s, 9H, Si(C<u>H</u>3)3). **^{13}C NMR** (101 MHz, $CDCl_3$, δ, ppm): 156.54, 146.22, 144.61, 142.07, 138.00, 137.36, 129.76, 128.72, 128.02, 127.85, 127.27, 125.86, 107.14 (C≡C), 103.90 (C≡C), 0.23, -0.01, -0.40, -0.94. **^{29}Si NMR** (79 MHz, $CDCl_3$ δ, ppm) -2.62, -4.64, -8.39, -18.92. **GC-MS** (EI, m/z): 506(M^+, 3), 432(5), 417(17), 344(9), 310(100), 294(11), 233(30), 221(15), 193(33), 133(42), 73(86). **FT-IR** (cm-

[1]): 699, 791, 832, 1056, 1250, 1493, 1600, 2889, 2957, 3056. **Elemental Anal.** for $C_{28}H_{42}OSi_4$ (%): calcd.: C, 66.34; H, 8.35; found: C, 66.59; H, 8.43. Colorless oil. Isolated yield: 81%.

*1-((E)-1,2-Diphenylvinyl)-3-((E)-hex-2-en-4-yn-3-yl)-1,1,3,3-tetramethyldisiloxane (**4i**)*

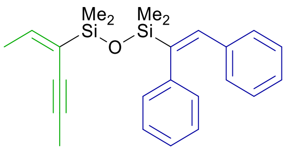

Chemical Formula: $C_{24}H_{30}OSi_2$
Molecular Weight: 390,67

^{1}H NMR (400 MHz, $CDCl_3$, δ, ppm): δ 7.33 – 7.26 (m, 2H, Ph), 7.24 – 7.17 (m, 2H, Ph), 7.13 – 6.96 (m, 7H, Ph), 6.92 (s, 1H, PhC=CHPh), 6.28 – 6.14 (m, 1H,C=CHCH$_3$), 2.01 (s, 3H, C≡CCH$_3$), 1.92 (d, $J_{H\text{-}H}$ = 6.5 Hz, 3H, C=CHCH$_3$), 0.20 (s, 6H, Si(CH$_3$)$_2$), 0.18 (s, 6H, Si(CH$_3$)$_2$)). **^{13}C NMR** (101 MHz, $CDCl_3$, δ, ppm 146.33, 145.33, 142.12, 137.96, 137.44, 129.73, 128.70, 128.03, 127.87, 127.24, 125.97, 125.83, 94.17 (C≡C), 78.29 (C≡C), 18.04, 4.87, 0.22 (Si(CH$_3$)$_2$)., -0.05 (Si(CH$_3$)$_2$). **^{29}Si NMR** (79 MHz, $CDCl_3$ δ, ppm) -2.98, -3.54. **GC-MS** (EI, m/z): 390(M$^+$, 21), 375(16), 311(16), 295(8), 221(7), 211(21), 193(21), 171(14), 148(10), 133(100), 117(10), 73(26). **FT-IR** (cm^{-1}): 663, 790, 858, 1046, 1250, 1448, 2854, 2926. **Elemental Anal.** for $C_{24}H_{30}OSi_2$ (%): calcd.: C, 73.79; H, 7.74; found: C, 74.01; H, 7.82. Yellowish oil. Isolated yield: 84%.

*1-((E)-1,4-Dicyclohexylbut-1-en-3-yn-2-yl)-3-((E)-1,2-diphenylvinyl)-1,1,3,3-tetramethyldisiloxane (**4j**)*

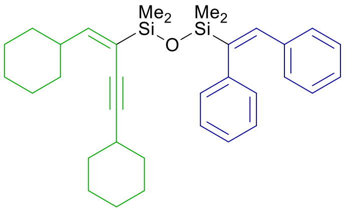

Chemical Formula: $C_{34}H_{46}OSi_2$
Molecular Weight: 526,91

^{1}H NMR (400 MHz, $CDCl_3$, δ, ppm): 7.18 – 6.82 (m, 10H, Ph), 6.77 (s, 1H, PhC=CHPh), 5.84 (d, $J_{H\text{-}H}$ = 8.5 Hz, 1H, C=CHCy), 2.46 (m, 2H), 1.67 – 0.90 (m, 20H), 0.05 (s, 6H, Si(CH$_3$)$_2$), 0.05 (s, 6H, Si(CH$_3$)$_2$). **^{13}C NMR** (101 MHz, $CDCl_3$, δ, ppm): 156.15, 146.35, 142.17, 137.94, 137.45, 129.75, 128.69, 127.99, 127.87, 127.21, 125.81, 122.59, 102.25, 77.48, 77.16, 76.84, 40.99, 33.13, 32.23, 30.19, 26.25, 26.18, 25.98, 24.87, 0.24 (Si(CH$_3$)$_2$), -0.11 (Si(CH$_3$)$_2$). **^{29}Si NMR** (79 MHz, $CDCl_3$ δ, ppm): -3.12, -3.38. **GC-MS** (EI, m/z): 526(M$^+$, 3), 443(12), 311(36), 295(6), 221(10), 193(17), 133(100), 119(9), 73(9). **FT-IR** (cm^{-1}): 662, 788, 856, 1047, 1252, 1447, 2852, 2926, 3055. **Elemental Anal.** for $C_{34}H_{46}OSi_2$ (%): calcd.: C, 77.50; H, 8.80; found: C, 77.39; H, 8.87. Colorless oil. Isolated yield: 83%.

*1-((E)-1,4-Di(thiophen-3-yl)but-1-en-3-yn-2-yl)-3-((E)-1,2-diphenylvinyl)-1,1,3,3-tetramethyldisiloxane (**4k**)*

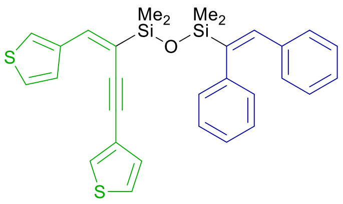

Chemical Formula: $C_{30}H_{30}OS_2Si_2$
Molecular Weight: 526,86

^{1}**H NMR** (400 MHz, $CDCl_3$, δ, ppm): 7.94 – 7.88 (m, 1H), 7.74 (dd, $J_{H\text{-}H}$ = 5.1, 1.2 Hz, 1H), 7.45 – 7.40 (m, 1H), 7.34 – 7.25 (m, 4H), 7.18 – 6.95 (m, 12H), 0.35 (s, 6H, Si(C$\underline{H}_3$)$_2$), 0.28 (s, 6H, Si(C$\underline{H}_3$)$_2$). ^{13}**C NMR** (101 MHz, $CDCl_3$, δ, ppm): 146.07, 141.99, 140.42, 138.46, 138.20, 137.26, 129.76, 128.77, 128.26, 128.03, 127.86, 127.33, 126.05, 125.93, 125.50, 125.14, 123.44, 121.14, 95.74 (C≡C), 90.26 (C≡C), 0.34 (Si($\underline{C}H_3$)$_2$), 0.08 (Si($\underline{C}H_3$)$_2$). ^{29}**Si NMR** (79 MHz, $CDCl_3$ δ, ppm): -2.24. **GC-MS** (EI, m/z): 526(M^+, 15), 435(5), 362(9), 331(25), 311(27), 257(57), 233(24), 193(55), 179(10), 165(17), 149(18), 133(100), 112(24), 73(46), 57(38). **FT-IR** (cm^{-1}): 689, 797, 856, 1046, 1250, 2852, 2926, 3055. **Elemental Anal.** for $C_{30}H_{30}OS_2Si_2$ (%): calcd.: C, 68.39; H, 5.74; found: C, 69.25; H, 5.65. Brown oil. Isolated yield: 63%.

*1-((E)-1,4-Diphenylbut-1-en-3-yn-2-yl)-3-((E)-1,2-diphenylvinyl)-1,1,3,3-tetramethyldisiloxane (**4l**)*

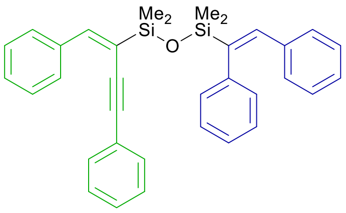

Chemical Formula: $C_{34}H_{34}OSi_2$
Molecular Weight: 514,82

^{1}**H NMR** (400 MHz, $CDCl_3$, δ, ppm): 8.16 – 8.08 (m, 2H, Ph), 7.62 – 7.56 (m, 2H, Ph), 7.53 – 7.46 (m, 2H, Ph), 7.46 – 7.39 (m, 4H, Ph), 7.36 – 7.31 (m, 1H, Ph), 7.23 – 7.17 (m, 6H, Ph), 7.10 (m, 4H, Ph), 0.50 (s, 6H, Si(C$\underline{H}_3$)$_2$), 0.41 (s, 6H, Si(C$\underline{H}_3$)$_2$)). ^{13}**C NMR** (101 MHz, $CDCl_3$, δ, ppm): 146.06, 144.86, 141.98, 138.24, 137.75, 137.26, 131.44, 129.79, 129.11, 128.78, 128.49, 128.40, 128.11, 128.02, 127.86, 127.32, 125.94, 124.44, 123.02, 101.09 ($\underline{C}$≡C), 90.29 (C≡$\underline{C}$) , 0.36 (Si($\underline{C}H_3$)$_2$), 0.02 (Si($\underline{C}H_3$)$_2$). ^{29}**Si NMR** (79 MHz, $CDCl_3$ δ, ppm): -2.11, -2.19. **GC-MS** (EI, m/z): 514(M^+, 13), 423(8), 311(35), 245(73), 233(23), 193(52), 133(100), 129(72), 117(18), 73(40). **FT-IR** (cm^{-1}): 690, 754, 787, 830, 956, 1066, 1253, 1225, 1445, 1489, 1597, 2957, 3021, 3056. **Elemental Anal.** for $C_{34}H_{34}OSi_2$ (%): calcd.: C, 79.32; H, 6.66; found: C, 80.01; H, 6.75. Yellowish oil. Isolated yield: 70%.

*1-((E)-1,2-Diphenylvinyl)-1,1,3,3-tetramethyl-3-((E)-4-phenyl-1-(triisopropylsilyl)but-1-en-3-yn-2-yl)disiloxane (**4m**)*

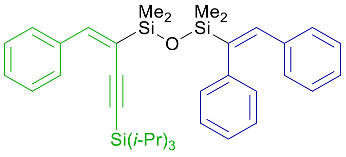

Chemical Formula: $C_{37}H_{50}OSi_3$
Molecular Weight: 595,06

^{1}**H NMR** (400 MHz, $CDCl_3$, δ, ppm): 8.23 – 8.12 (m, 2H, Ph), 7.48 – 7.08 (m, 17H), 1.26 (s, 21H, Si(CH(C$\underline{H}_3$)$_2$)$_3$), 0.45 (s, 6H, Si(C$\underline{H}_3$)$_2$)), 0.40 (s, 6H, Si(C$\underline{H}_3$)$_2$)). ^{13}**C NMR** (101 MHz, $CDCl_3$, δ, ppm): 146.03, 145.74, 141.98, 138.28, 137.67, 137.26, 129.80, 129.23, 128.82, 128.81, 128.24, 128.04, 127.86, 127.35, 125.97, 123.51, 107.34 (C≡C), 104.10 (C≡C), 18.88 (Si(CH($\underline{C}H_3$)$_2$)$_3$), 11.68 (Si($\underline{C}H(CH_3)_2$)$_3$), 0.37 (Si($\underline{C}H_3$)$_2$), -0.03 (Si($\underline{C}H_3$)$_2$). ^{29}**Si NMR** (79 MHz, $CDCl_3$ δ, ppm): -.2.13, -2.23, -2.78. **GC-MS** (EI, m/z): 595(M^+, 4) 551(15), 509(24), 422(17), 393(8), 311(16), 295(47), 245(72), 221(23), 193(60), 129(52), 117(31), 73(100), 59(64). **FT-IR** (cm^{-1}): 695, 787, 830, 960, 1055, 1252, 1363, 1600, 2860, 2931, 2961, 3056. **Elemental Anal.** for $C_{37}H_{50}OSi_3$ (%): calcd.: C, 74.68; H, 8.47; found: C, 74.52; H, 8.38. Yellowish oil. Isolated yield: 69%.

*1-((E)-2,2-Dimethyldodec-3-en-5-yn-4-yl)-3-((E)-1,2-diphenylvinyl)-1,1,3,3-tetramethyldisiloxane (**4n**)*

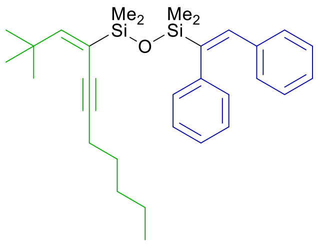

Chemical Formula: $C_{31}H_{44}OSi_2$
Molecular Weight: 488,86

¹H NMR (400 MHz, $CDCl_3$, δ, ppm): 7.36 – 7.26 (m, 2H), 7.23 – 7.19 (m, 1H), 7.11 – 7.08 (m, 3H), 7.07 – 7.04 (m, 2H), 7.02 – 6.96 (m, 2H), 6.93 (s, 1H, PhC=CHPh), 6.08 (s, 1H, C=CHCH₂), 2.36 (t, $J_{H\text{-}H}$ = 7.0 Hz, 2H, C=CHCH₂), 1.58 – 1.48 (m, 2H), 1.42 – 1.27 (m, 5H), 1.20 (s, 9H, C(CH₃)₃), 0.89 (m, 4H), 0.21 (s, 6H, Si(CH₃)₂)), 0.20 (s, 6H, Si(CH₃)₂)). **¹³C NMR** (101 MHz, $CDCl_3$, δ, ppm): 158.79, 146.36, 142.16, 137.95, 137.44, 129.74, 128.70, 128.01, 127.87, 127.23, 125.83, 122.06, 101.42 (C≡C), 79.63, 35.51, 31.54, 29.80, 29.05, 28.83, 22.76, 20.24, 14.22, 0.23 (Si(CH₃)₂), -0.14 (Si(CH₃)₂). **²⁹Si NMR** (79 MHz, $CDCl_3$ δ, ppm): -2.65, -3.17. **GC-MS** (EI, m/z): 502(M⁺, 1), 488(1), 445(11), 311(28), 295(8), 233(16), 193(27), 133(100), 73(20). **FT-IR** (cm^{-1}): 692, 784, 829, 958, 1054, 1251, 1360, 1599, 2859, 2929, 2955, 3055. **Elemental Anal.** for $C_{32}H_{46}OSi_2$ (%): calcd.: C, 76.43; H, 9.22; found: C, 76.54; H, 9.31. Yellowish oil. Isolated yield: 72%.

*1,1,3,3-Tetramethyl-1,3-di((E)-oct-4-en-4-yl)disiloxane (**5a**)*

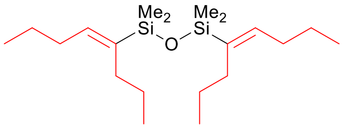

Chemical Formula: $C_{20}H_{42}OSi_2$
Molecular Weight: 354,73

¹H NMR (400 MHz, $CDCl_3$, δ, ppm): 5.81 (t, $J_{H\text{-}H}$ = 6.9 Hz, 2H, C=CHCH₂), 2.19 – 2.01 (m, 8H), 1.52 – 1.25 (m, 8H), 0.97 – 0.85 (m, 12H), 0.12 (s, 12H, Si(CH₃)₂). **¹³C NMR** (101 MHz, $CDCl_3$, δ, ppm): 141.25 (C=C), 140.89 (C=C), 31.50, 30.51, 23.50, 22.84, 14.64, 14.08, 0.95 (Si(CH₃)₂). **²⁹Si NMR** (79 MHz, $CDCl_3$ δ, ppm): -2.70. **GC-MS** (EI, m/z): 354(M⁺, 2), 339(4), 242(26), 227(16), 201(7), 173(14), 133(100), 119(33), 108(20), 73(13). **FT-IR** (cm^{-1}): 779, 830, 1039, 1252, 1457, 1612, 2872, 2931, 2957. **Elemental Anal.** for $C_{20}H_{42}OSi_2$ (%): calcd.: C, 67.72; H, 11.93; found: C, 67.83; H, 12.01. Colorless oil. Isolated yield: 93%. Despite previous study, the title compound was isolated and characterized for the first time.[12]

*1,3-Bis((E)-1,2-diphenylvinyl)-1,1,3,3-tetramethyldisiloxane (**5b**)*

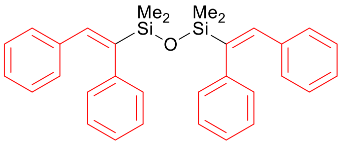

Chemical Formula: $C_{32}H_{34}OSi_2$
Molecular Weight: 490,79

¹H NMR (300 MHz, $CDCl_3$, δ, ppm): 7.41 – 6.90 (m, 22H, Ph, C=CHPh), 0.23 (s, 12H, Si(CH₃)₂). **¹³C NMR** (101 MHz, $CDCl_3$, δ, ppm): δ 146.31, 142.06, 138.09, 137.32, 129.77, 128.77, 128.04, 127.86, 127.31, 125.91, 0.29 (Si(CH₃)₂). **²⁹Si NMR** (79 MHz, $CDCl_3$ δ, ppm): -2.80. **GC-MS** (EI, m/z): 490(M⁺, 15), 311(100), 295(28), 233(31), 221(24), 193(62), 179(16), 133(87), 73(35). **FT-IR** (cm^{-1}): 693, 784, 831, 958, 1057, 1250, 1361, 1600,

2955, 3055. Melting point: 91 °C. White solid. Isolated yield: 88%. The analytical data are in agreement with the literature and were extended with ^{29}Si NMR, MS and FT-IR analyzes.[13]
Crystal structure determined for the first time, data presented in part: **7. Single crystal X-ray diffraction data**.

1,3-Bis((E)-1,2-bis(4-bromophenyl)vinyl)-1,1,3,3-tetramethyldisiloxane (**5c**)

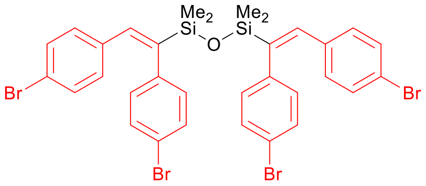

Chemical Formula: $C_{32}H_{30}Br_4OSi_2$
Molecular Weight: 806,38

^{1}H NMR (400 MHz, $CDCl_3$, δ, ppm): 7.43 (d, J_{H-H} = 8.4 Hz, 4H, Ph), 7.31 – 7.25 (m, 4H, Ph), 6.89 (d, J_{H-H} = 8.4 Hz, 4H, Ph), 6.85 – 6.78 (m, 4H, Ph), 0.20 (s, 12H, Si(CH_3)$_2$). **^{13}C NMR** (101 MHz, $CDCl_3$, δ, ppm): 145.73, 140.45, 137.55, 135.68, 132.10, 131.40, 131.17, 129.52, 121.66, 120.23, 0.27 (Si(CH_3)$_2$). **^{29}Si NMR** (79 MHz, $CDCl_3$ δ, ppm): -2.42. **ESI-HRMS** (m/z) ($[M+Na]^+$, (%)): 828.8435. **FT-IR** (cm^{-1}): 693, 784, 829, 958, 1057, 1250, 1361, 1599, 2955, 3056. **Elemental Anal.** for $C_{32}H_{30}Br_4OSi_2$ (%): calcd.: C, 47.66; H, 3.75; found: C, 47.61; H, 3.81. Colorless liquid. Isolated yield: 58%.

1,1,3,3-Tetramethyl-1,3-di((E)-styryl)disiloxane (**5d**)

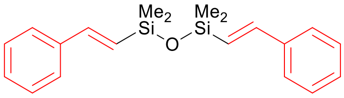

Chemical Formula: $C_{20}H_{26}OSi_2$
Molecular Weight: 338,60

^{1}H NMR (300 MHz, $CDCl_3$, δ, ppm): 7.56 – 7.46 (m, 4H, Ph), 7.45 – 7.28 (m, 6H, Ph), 7.05 (d, J_{H-H} = 19.2 Hz, 1H, CH=CH), 6.54 (d, J_{H-H} = 19.2 Hz, 1H, CH=CH), 0.35 (s, 12H, Si(CH_3)$_2$).**^{13}C NMR** (101 MHz, $CDCl_3$, δ, ppm): 144.46 (CH=CH), 138.29, 128.71, 128.65, 128.30, 126.69, 1.01 (Si(CH_3)$_2$). **^{29}Si NMR** (79 MHz, $CDCl_3$ δ, ppm): - 2.05 (OSi(CH_3)$_2$). **GC-MS** (EI, m/z): 338(M^+, 17), 246(64), 220(100), 193(85), 180(21), 145(27), 133(41), 113(26), 102(12), 89(11), 74(20), 59(19). **FT-IR** (cm^{-1}): 687, 781, 843, 990, 1027, 1250, 1446, 1494, 1604, 2956, 3025. Yellowish oil. Isolated yield 88%. The analytical data are in agreement with the literature.[14]

1,3-Bis((E)-4-bromostyryl)-1,1,3,3-tetramethyldisiloxane (**5e**)

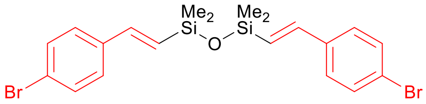

Chemical Formula: $C_{20}H_{24}Br_2OSi_2$
Molecular Weight: 496,39

^{1}H NMR (400 MHz, $CDCl_3$, δ, ppm): 7.47 (d, J_{H-H} = 8.5 Hz, 4H, Ph), 7.31 (d, J_{H-H} = 8.4 Hz, 4H, Ph), 6.91 (d, J_{H-H} = 19.2 Hz, 2H, CH=CH), 6.46 (d, J_{H-H} = 19.2 Hz, 2H, CH=CH), 0.30 (s, 12H, Si(CH_3)$_2$). **^{13}C NMR** (101 MHz, $CDCl_3$, δ, ppm): 143.17 (CH=CH), 137.14, 131.77, 129.65, 128.16, 122.21, 0.95 (Si(CH_3)$_2$). **^{29}Si NMR** (79 MHz, CDC_3 δ, ppm): - 1.99 (OSi(CH_3)$_2$). **GC-MS** (EI, m/z): 417($(M-77)^+$, 4), 415($(M-79)^+$, 5), 327(4), 325(5), 297(15), 299(14), 271(10), 273(10), 207(9), 133(100), 117(19), 73(25). **FT-IR** (cm^{-1}): 493, 779, 844, 986,

1050, 1250, 1396, 1484, 1604, 2958, 2989. Melting point: 65 °C. White solid. Isolated yield 73%. The analytical data are in agreement with the literature and were extended with ^{29}Si NMR.[14]
Crystal structure determined for the first time, data presented in part: **7. Single crystal X-ray diffraction data**.

*1,3-Bis((E)-4-fluorostyryl)-1,1,3,3-tetramethyldisiloxane (**5f**)*

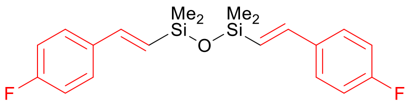

Chemical Formula: $C_{20}H_{24}F_2OSi_2$
Molecular Weight: 374,58

^{1}H NMR (300 MHz, $CDCl_3$, δ, ppm): 7.45 – 7.36 (m, 4H, Ph), 7.02 (m, 4H, Ph), 6.93 (d, $J_{H\text{-}H}$ = 19.2 Hz, 1H, CH=CH), 6.37 (d, $J_{H\text{-}H}$ = 19.2 Hz, 1H, CH=CH), 0.28 (s, 12H, Si(CH_3)$_2$). **^{13}C NMR** (101 MHz, $CDCl_3$, δ, ppm): 162.87 (d, $J^1_{C\text{-}F}$ = 248 Hz), 143.19, 134.52, 134.49, 128.37 (d, $J^4_{C\text{-}F}$ = 2.3 Hz), 128.22 (d, $J^3_{C\text{-}F}$ = 8.1 Hz), 115.55 (d, $J^2_{C\text{-}F}$ = 21.5 Hz), 0.98 Si(CH_3)$_2$. **^{29}Si NMR** (79 MHz, $CDCl_3$ δ, ppm): -2.07 (OSi(CH_3)$_2$). **MS** (EI, m/z): 374(M^+, 12), 265(53), 237(100), 211(64), 197(19), 163(17), 133(25), 117(28), 91(14), 73(44), 59(34). **FT-IR** (cm^{-1}):. 796, 985, 1034, 1155,1225, 1252, 1410, 1505, 1601, 2957. Yellowish oil. Isolated yield: 89%. The analytical data are in agreement with the literature and were extended with ^{29}Si NMR.[15]

*1,1,3,3-Tetramethyl-1,3-bis((E)-4-(trifluoromethyl)styryl)disiloxane (**5g**)*

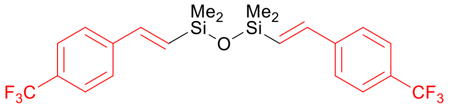

Chemical Formula: $C_{22}H_{24}F_6OSi_2$
Molecular Weight: 474,59

^{1}H NMR (400 MHz, $CDCl_3$, δ, ppm): 7.59 (d, $J_{H\text{-}H}$ = 8.3 Hz, 4H), 7.52 (d, $J_{H\text{-}H}$ = 8.3 Hz, 4H), 7.01 (d, $J_{H\text{-}H}$ = 19.2 Hz, 2H, CH=CH), 6.59 (d, $J_{H\text{-}H}$ = 19.2 Hz, 2H, CH=CH), 0.33 (s, 12H, Si(CH_3)$_2$). **^{13}C NMR** (101 MHz, $CDCl_3$, δ, ppm): 143.00, 141.58, 131.99, 130.10 (q, $^2J_{C\text{-}F}$ = 32.4 Hz), 126.82, 125.65 (q, $^3J_{C\text{-}F}$ = 3.6 Hz), 123.00, 120.30, 0.87 (Si(CH_3)$_2$). **^{29}Si NMR** (79 MHz, $CDCl_3$ δ, ppm): - 1.90 (OSi(CH_3)$_2$). **GC-MS** (EI, m/z): 474(M^+, 1), 459(3), 323(12), 304(62), 281(10), 259(5), 235(5), 215(6), 167(13), 155(25), 151(100), 127(19), 117(13), 73(22), 59(18). **FT-IR** (cm^{-1}): 791, 1014, 1065, 1258, 1323, 1412, 2962. **Elemental Anal.** for $C_{22}H_{24}F_6OSi_2$ (%): calcd.: C, 55.68; H, 5.10; found: C, 55.49; H, 5.01. Melting point: 51 °C. White solid. Isolated yield: 85%.
Crystal structure determined for the first time, data presented in part: **7. Single crystal X-ray diffraction data**.

*1,3-Bis((E)-4-methoxystyryl)-1,1,3,3-tetramethyldisiloxane (**5h**)*

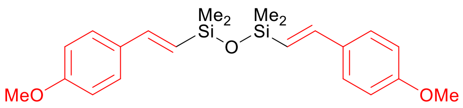

Chemical Formula: $C_{22}H_{30}O_3Si_2$
Molecular Weight: 398,65

^{1}H NMR (300 MHz, $CDCl_3$, δ, ppm): 7.42 (d, $J_{H\text{-}H}$ = 8.7 Hz, 4H, Ph), 6.98 (d, $J_{H\text{-}H}$ = 19.2 Hz, 2H, CH=CH), 6.90 (d, $J_{H\text{-}H}$ = 8.7 Hz, 4H, Ph), 6.35 (d, $J_{H\text{-}H}$ = 19.2 Hz, 2H, CH=CH), 3.83 (s, 6H, OCH_3), 0.32 (s, 12H, Si(CH_3)$_2$). **^{13}C NMR** (101 MHz, $CDCl_3$, δ, ppm): 159.83, 143.84, 136.36, 131.31, 127.94, 127.53, 126.05, 114.03, 111.72, 55.45 (OCH_3), 1.06 (Si(CH_3)$_2$). **^{29}Si NMR** (79 MHz, $CDCl_3$ δ, ppm): -2.16 (OSi(CH_3)$_2$). **GC-MS** (EI, m/z): 398(M^+, 30), 383(8), 277(33), 275(32), 264(15), 249(100), 223(96), 193(21), 176(17), 160(23),

133(47), 121(30), 91(18), 73(37), 59(20). **FT-IR** (cm^{-1}): 690, 787, 830, 958, 1057, 1251, 1361, 1600, 2986. Colorless oil. Isolated yield: 85%. The analytical data are in agreement with the literature and were extended with ^{29}Si NMR.[14]

*1,3-Bis((E)-3,3-dimethylbut-1-en-1-yl)-1,1,3,3-tetramethyldisiloxane (**5i**)*

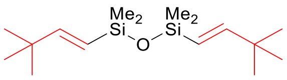

Chemical Formula: $C_{16}H_{34}OSi_2$
Molecular Weight: 298,62

^{1}H NMR (300 MHz, $CDCl_3$, δ, ppm): 6.09 (d, $J_{H\text{-}H}$ = 19.0 Hz, 2H, CH=CH), 5.50 (d, $J_{H\text{-}H}$ = 19.0 Hz, 2H, CH=CH), 1.00 (s, 18H, $C(CH_3)_3$), 0.12 (s, 12H, $Si(CH_3)_2$). **^{13}C NMR** (101 MHz, $CDCl_3$, δ, ppm): 158.28 (CH=CH), 122.76 (CH=CH), 35.01, 29.17, 1.04 ($Si(CH_3)_2$). **^{29}Si NMR** (79 MHz, $CDCl_3$ δ, ppm): - 2.44 ($OSi(CH_3)_2$). **GC-MS** (EI, m/z): 298(M^+, 1), 283(9), 241(19), 201(13), 157(15), 133(100), 117(15), 73(30). **FT-IR** (cm^{-1}): 760, 796, 846, 1039, 1251, 1613, 2866, 2902, 2957. Colorless oil. Isolated yield 79%. The analytical data are in agreement with the literature and were extended with ^{29}Si NMR.[14]

*(5E,10E)-2,2,4,4,7,7,9,9,12,12,14,14-Dodecamethyl-3,8,13-trioxa-2,7,9,14-tetrasilapentadeca-5,10-diene (**5j**)*

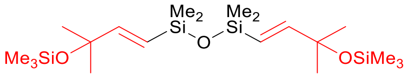

Chemical Formula: $C_{20}H_{46}O_3Si_4$
Molecular Weight: 446,93

^{1}H NMR (400 MHz, $CDCl_3$, δ, ppm): 6.17 (d, $J_{H\text{-}H}$ = 18.9 Hz, 1H, CH=CH), 5.70 (d, $J_{H\text{-}H}$ = 18.9 Hz, 1H, CH=CH), 1.29 (s, 6H, $C(CH_3)_2$), 0.14 (s, 6H, $Si(CH_3)_2$), 0.11 (s, 9H, $Si(CH_3)_3$). **^{13}C NMR** (101 MHz, $CDCl_3$, δ, ppm): 154.84 (C=C), 124.33 (C=C), 74.75, 30.11, 2.76, 0.95. **^{29}Si NMR** (79 MHz, $CDCl_3$ δ, ppm): 9.70 ($OSi(CH_3)_3$), - 2.44 ($OSi(CH_3)_2$). **GC-MS** (EI, m/z): 446(M^+, 1), 289(2), 221(59), 207(13), 147(50), 131(100), 73(85). **FT-IR** (cm^{-1}): 752, 807, 835, 1034, 1148, 1249, 1619, 2959. **Elemental Anal.** for $C_{20}H_{46}O_3Si_4$ (%): calcd.: C, 53.75; H, 10.37; found: C, 53.88; H, 10.41. Yellowish oil. Characterized from the crude reaction mixture after the filtration through the syringe filter. Yield: 95%.

*1,3-Bis((E)-2-(triisopropylsilyl)-1-vinyl)-1,1,3,3-tetramethyldisiloxane (**5k**)*

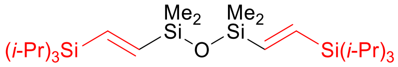

Chemical Formula: $C_{26}H_{58}OSi_4$
Molecular Weight: 499,09

^{1}H NMR (300 MHz, $CDCl_3$, δ, ppm): 6.65 (d, $J_{H\text{-}H}$ = 23.0 Hz, 2H, CH=CH), 6.57 (d, $J_{H\text{-}H}$ = 23.0 Hz, 2H, CH=CH), 1.06 (s, 42H, $Si(CH(CH_3)_2)_3$), 0.15 (s, 12H, $Si(CH_3)_2$). **^{13}C NMR** (101 MHz, $CDCl_3$, δ, ppm): 153.14 (CH=CH), 145.74 (CH=CH), 18.78, 10.87, 0.62 ($OSi(CH_3)_2$. **^{29}Si NMR** (79 MHz, $CDCl_3$ δ, ppm): -1.72, -5.25. **GC-MS** (EI, m/z): 499(M^+, 1), 455(16), 413(9), 371(6), 311(15), 283(17), 273(21), 241(7), 199(7), 171(6), 157(100), 133(20), 129(56), 115(55), 101(23), 73(74), 59(68). **FT-IR** (cm^{-1}): 654, 794, 839, 1014, 1046, 1251, 1462, 2864, 2941. **Elemental Anal.** for $C_{26}H_{58}OSi_4$ (%): calcd.: C, 62.57; H, 11.71; found: C, 62.70; H, 11.80. Colorless oil. Isolated yield: 95%.

1-Methyl-4-(1-propyl-1-penten-1-yl)benzene (**7a**)

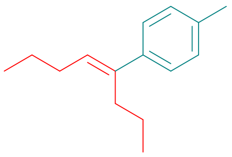

[1]**H NMR** (300 MHz, $CDCl_3$, δ, ppm): 7.26 (d, $J_{H\text{-}H}$ = 8.2 Hz, 2H, Ph), 7.12 (d, $J_{H\text{-}H}$ = 7.8 Hz, 2H, Ph), 5.66 (t, $J_{H\text{-}H}$ = 7.2 Hz, 1H, C<u>H</u>=C), 2.54 – 2.40 (m, 2H), 2.35 (s, 3H), 2.25 – 2.08 (m, 2H), 1.53 – 1.43 (m, 2H), 1.43 – 1.32 (m, 2H), 0.98 (t, $J_{H\text{-}H}$ = 7.4 Hz, 3H), 0.90 (t, $J_{H\text{-}H}$ = 7.3 Hz, 3H). [13]**C NMR** (101 MHz, $CDCl_3$, δ, ppm): 140.78, 139.97, 136.08, 128.96, 128.61, 126.36, 31.85, 30.79, 23.26, 21.98, 21.16, 14.12, 14.09. **GC-MS** (EI, m/z): 202(M+ , 32), 187(101), 173 (14), 159 (54), 132(24), 131(100), 129(21), 128(19), 117(15), 116(11), 115(24), 105(30), 91(24). Colorless oil. Isolated yield: 92%. The analytical data are in agreement with the literature.[16]

5. NMR spectra

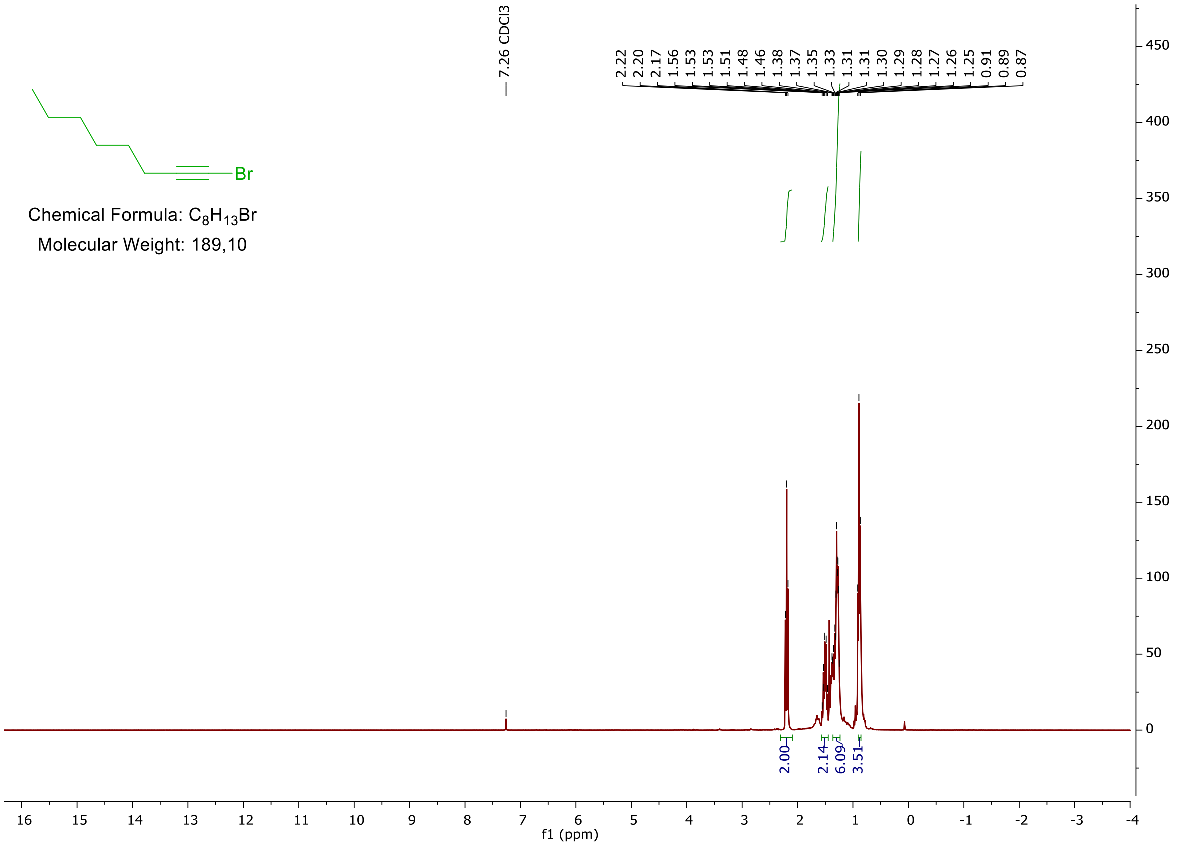

Figure S1. ^{1}H NMR spectrum of *1-bromooct-1-yne.*

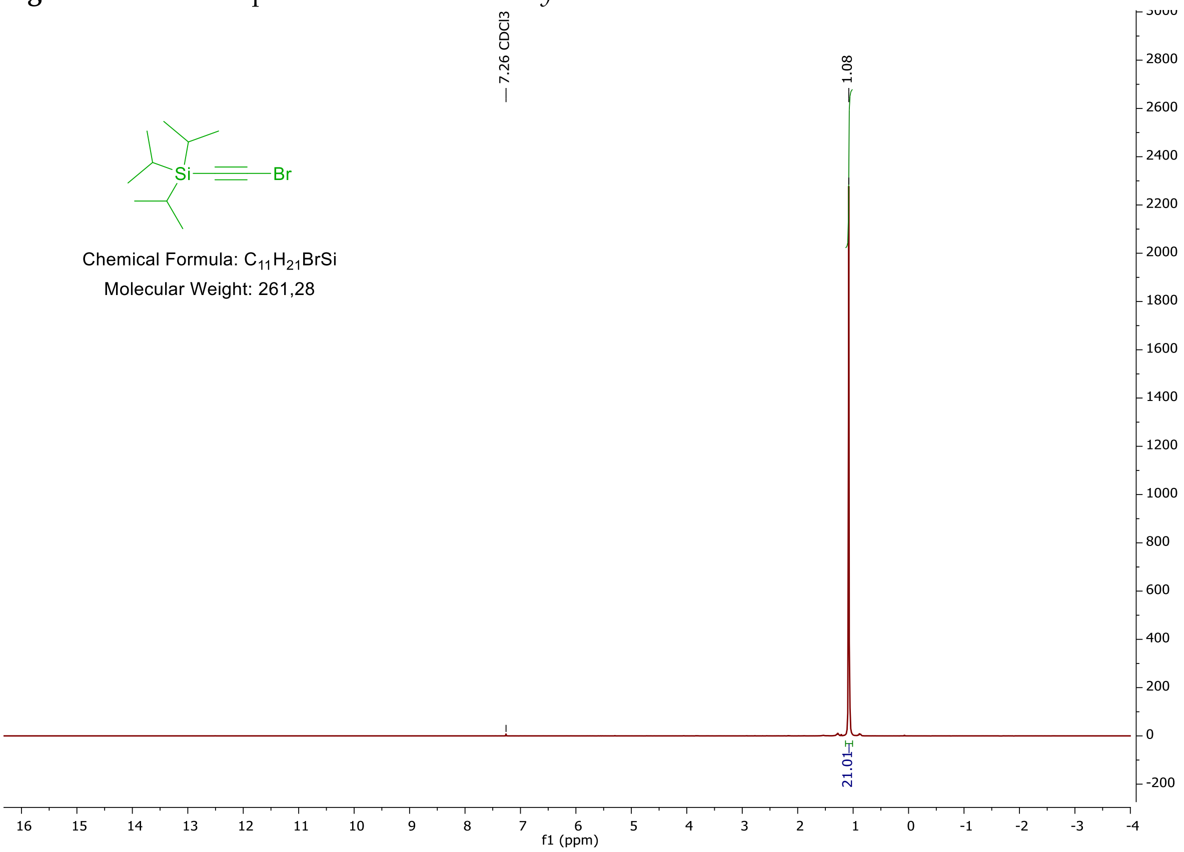

Figure S2. ^{1}H NMR spectrum of *(bromoethynyl)triisopropylsilane.*

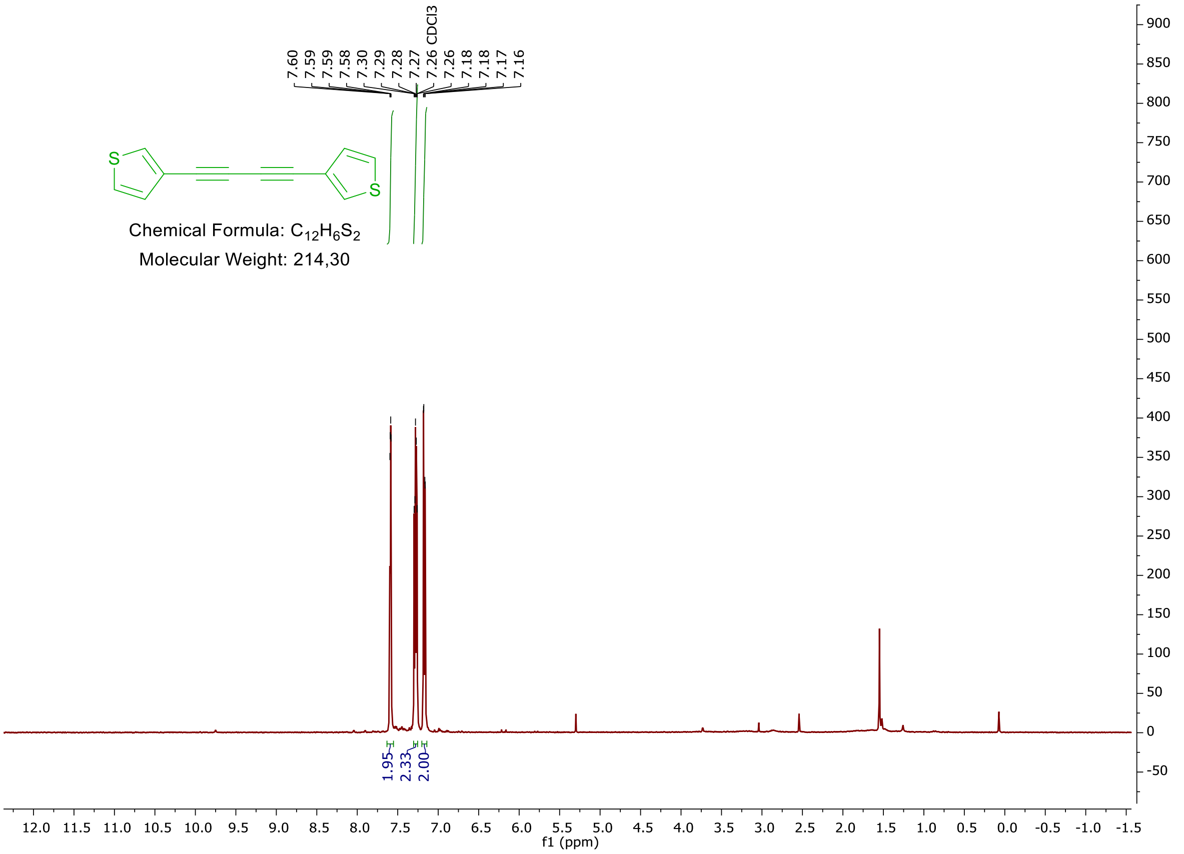

Figure S3. ^{1}H NMR spectrum of **2k**.

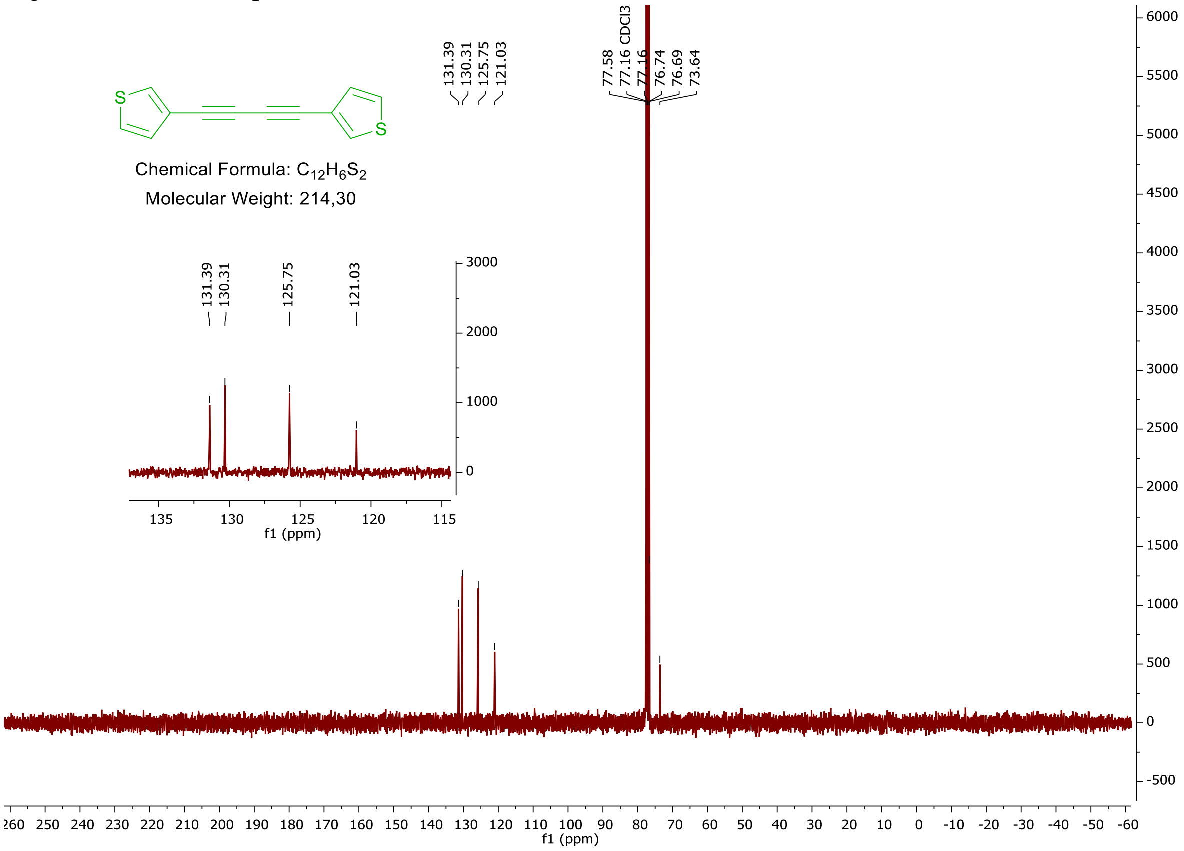

Figure S4. ^{13}C NMR spectrum of **2k**.

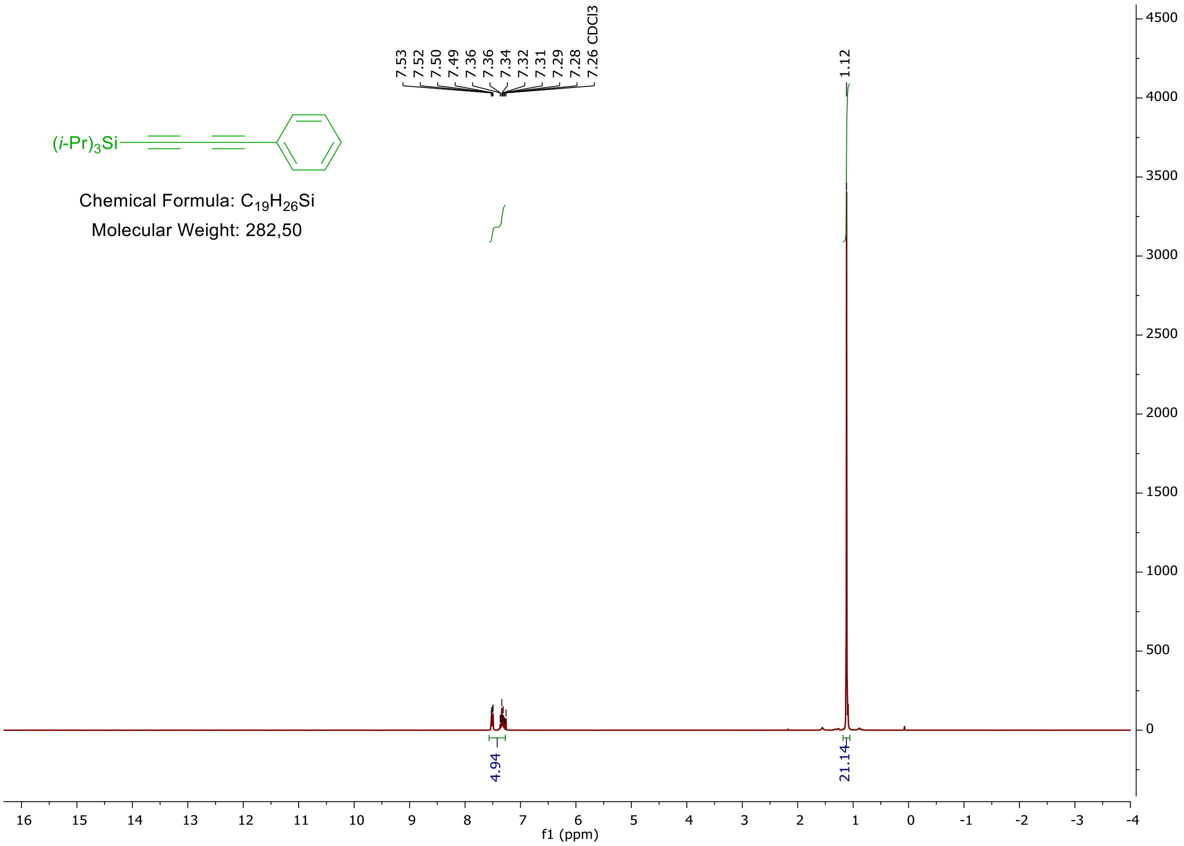

Figure S5. ^{1}H NMR spectrum of **2m**.

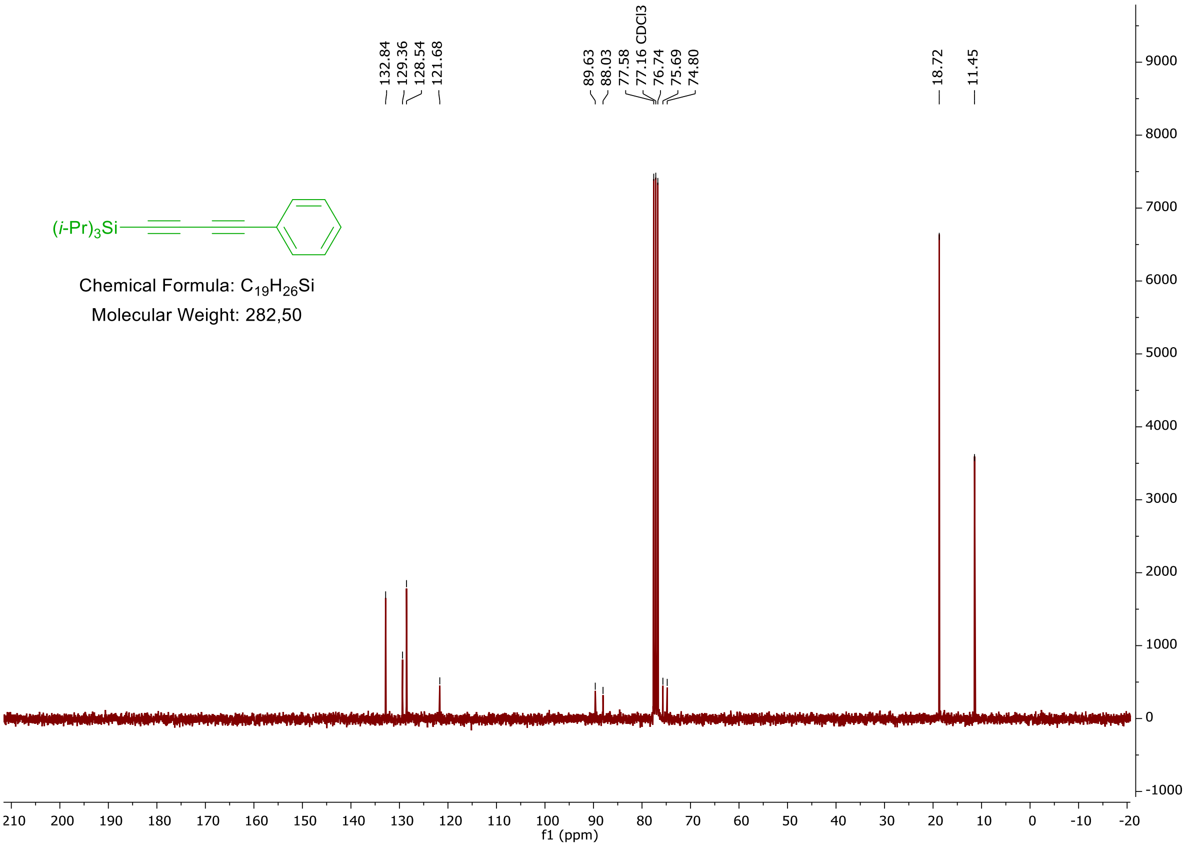

Figure S6. ^{13}C NMR spectrum of **2m**.

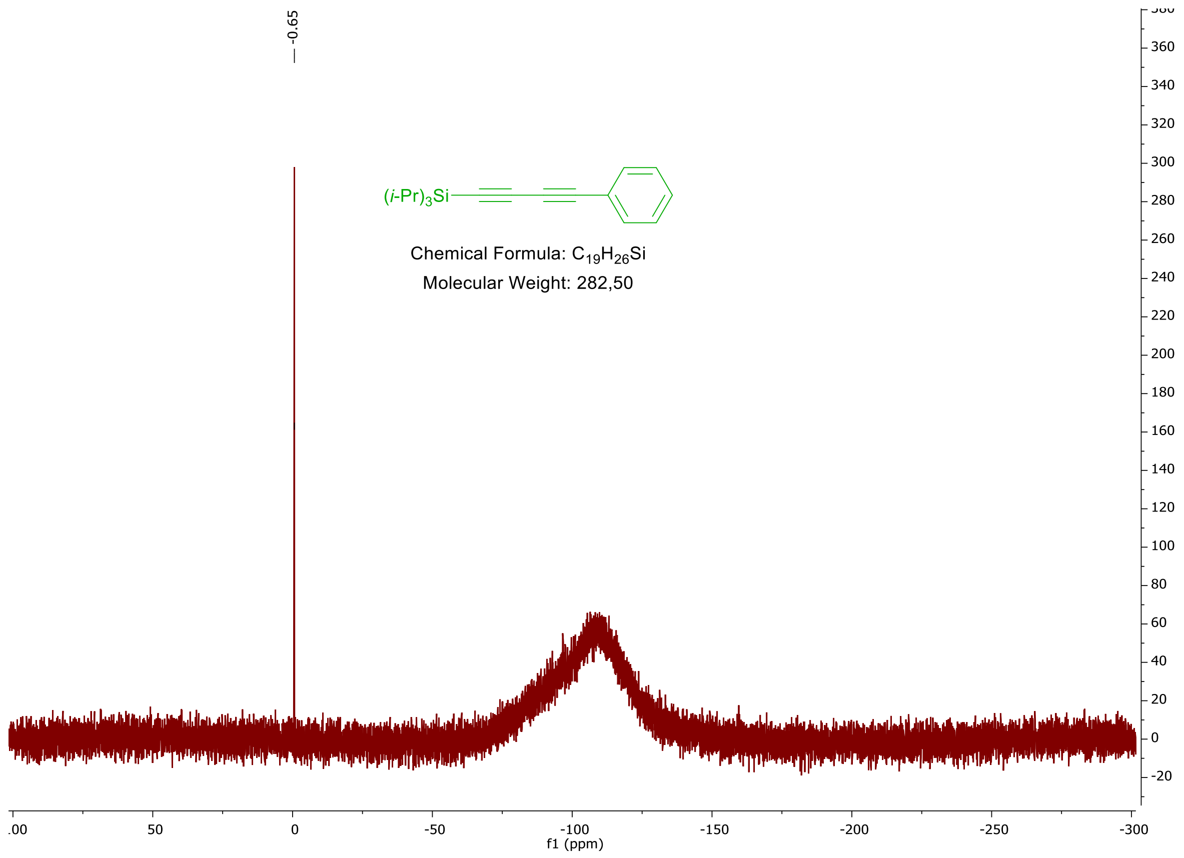

Figure S7. ^{29}Si NMR spectrum of **2m**.

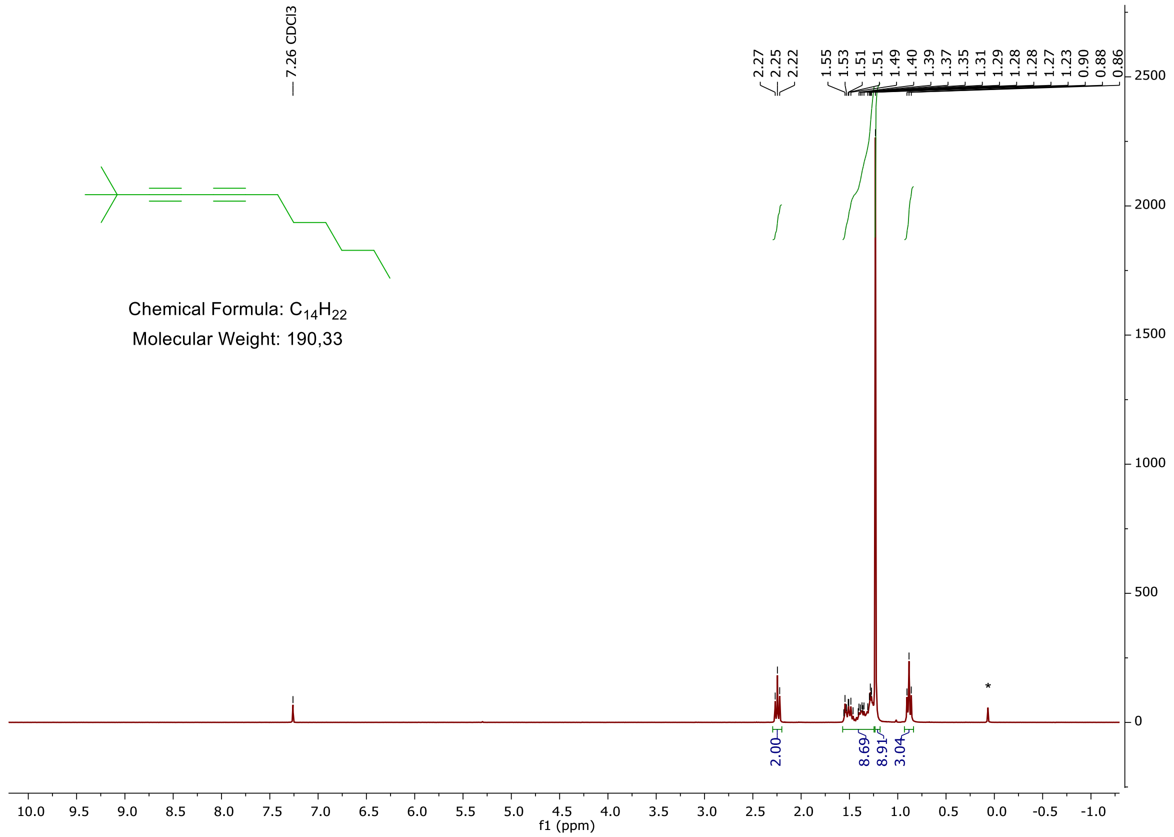

Figure S8. ^{1}H NMR spectrum of **2n**. *Grease.

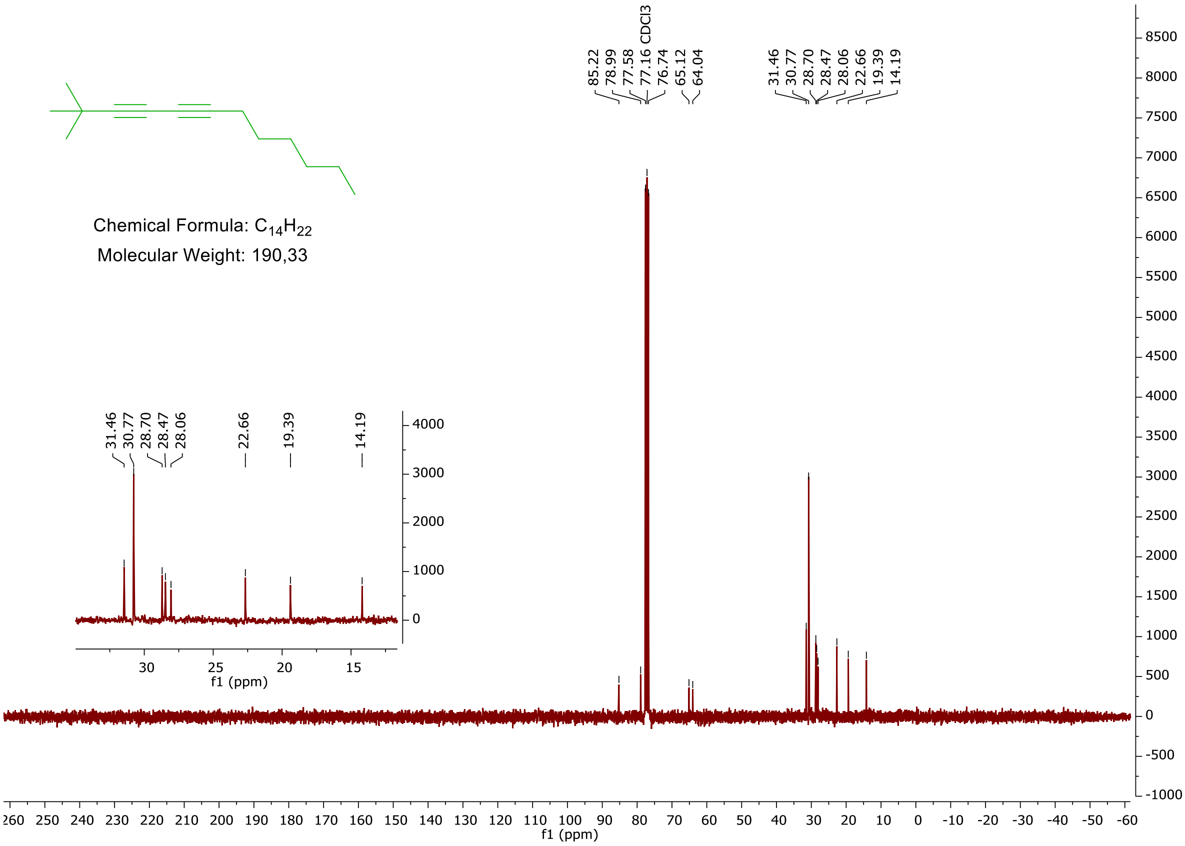

Figure S9. ^{13}C NMR spectrum of **2n**.

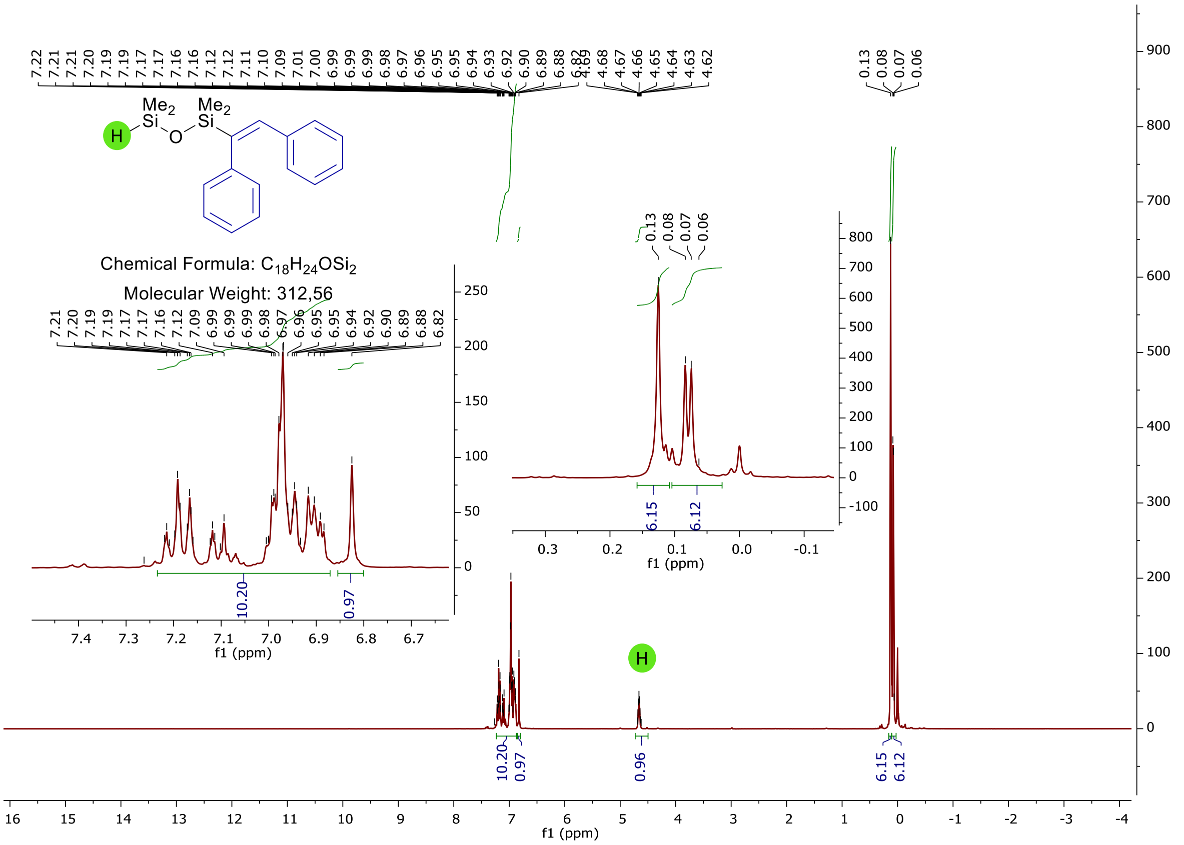

Figure S10. ^{1}H NMR spectrum of **3a**.

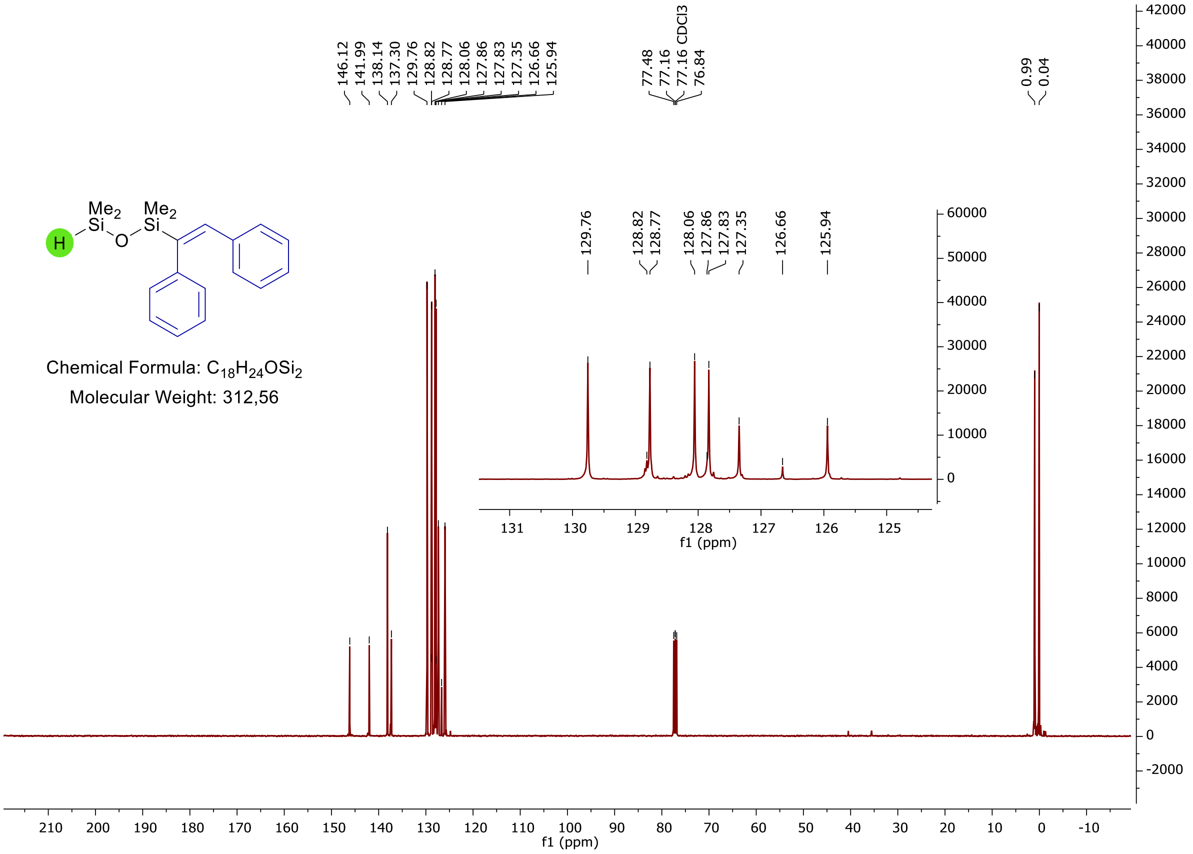

Figure S11. ^{13}C NMR spectrum of **3a**.

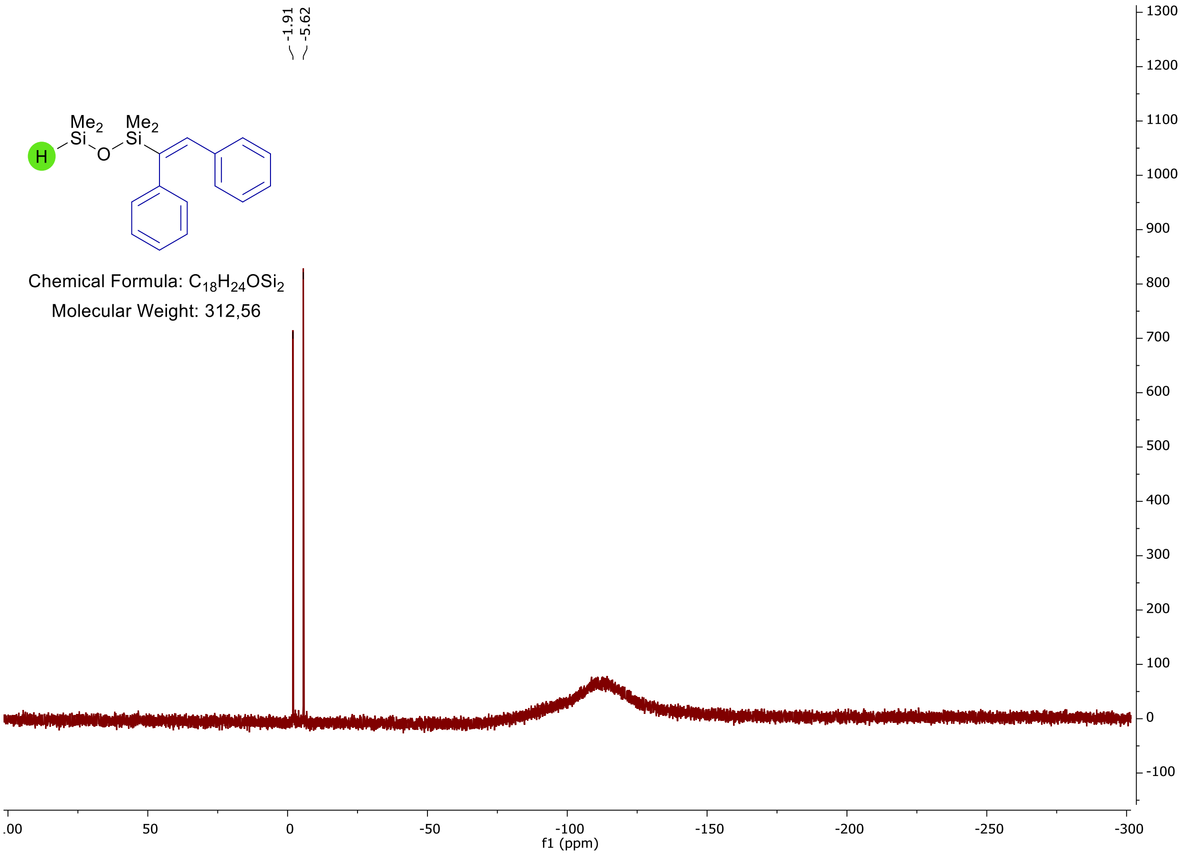

Figure S12. ^{29}Si NMR spectrum of **3a**.

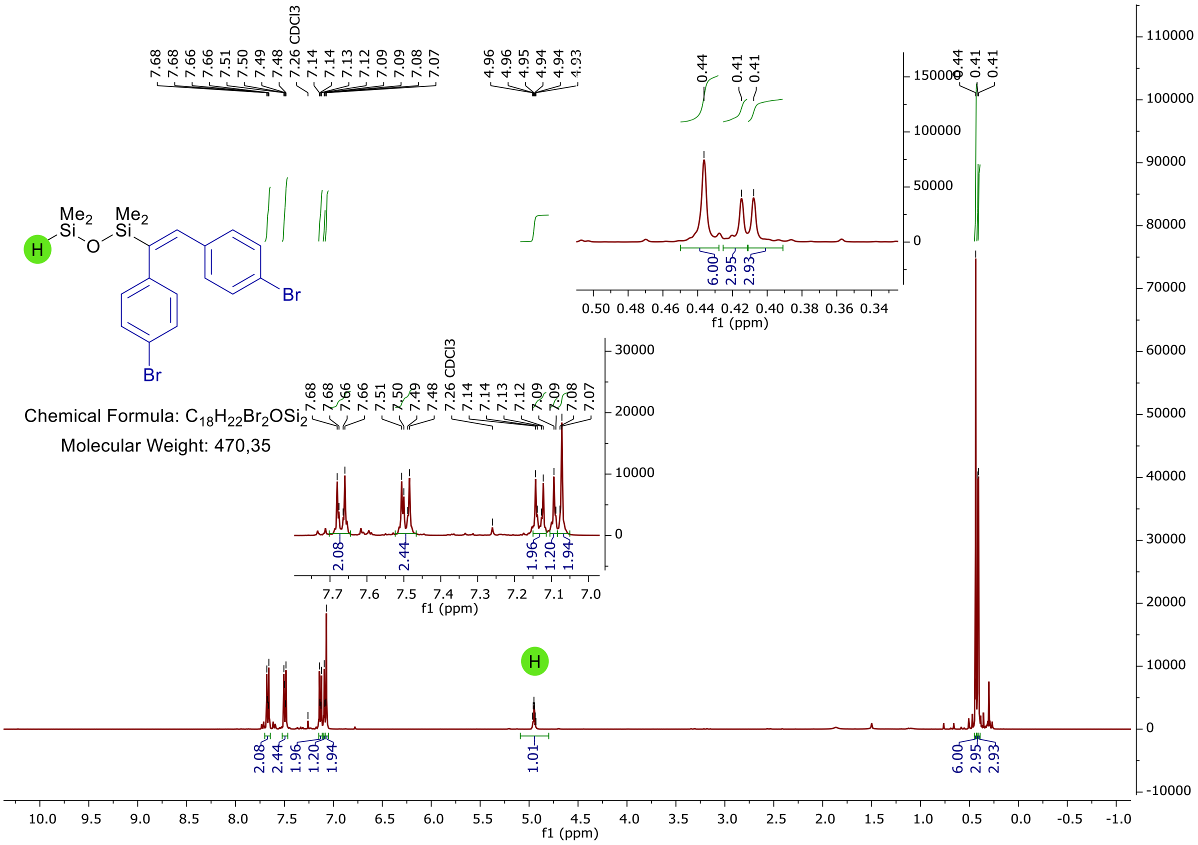

Figure S13. ^{1}H NMR spectrum of **3b**

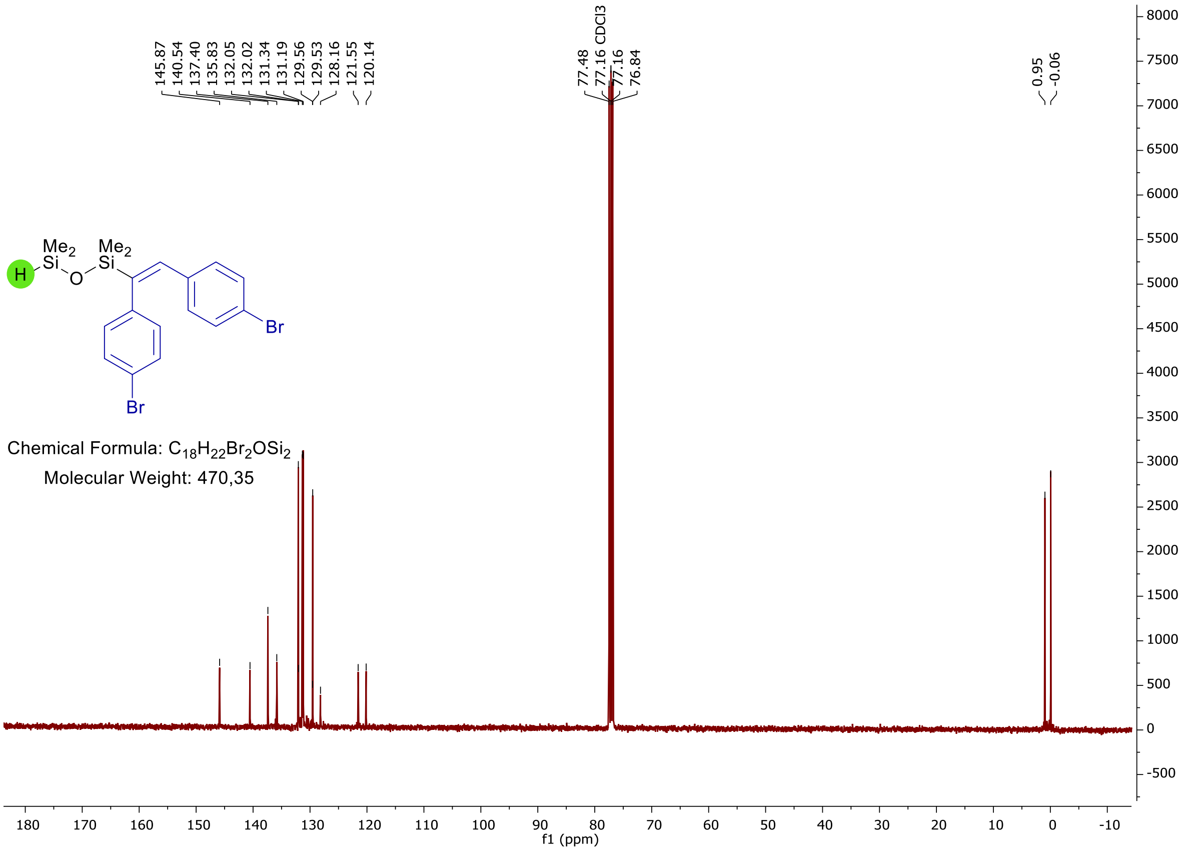

Figure S14. ^{13}CNMR spectrum of **3b.**

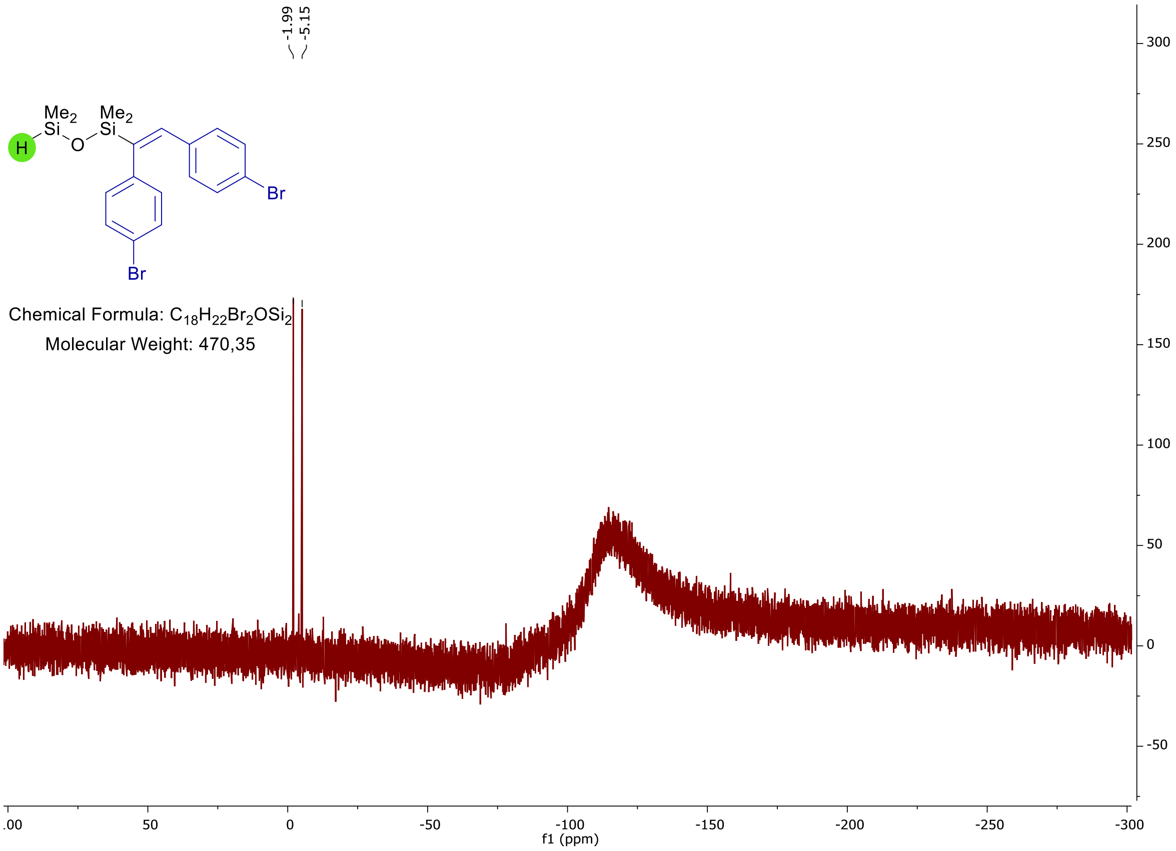

Figure S15. ^{29}Si NMR spectrum of **3b**.

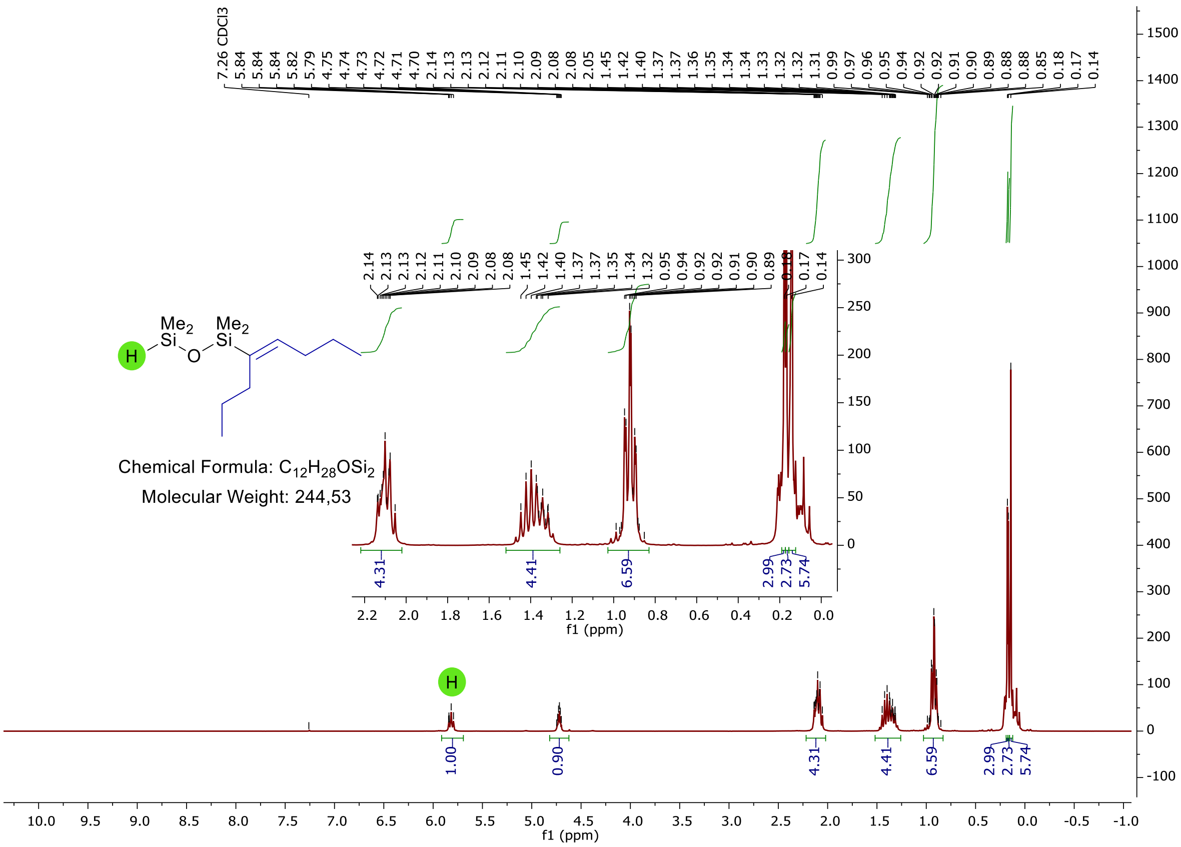

Figure S16. ^{1}H NMR spectrum of **3c**.

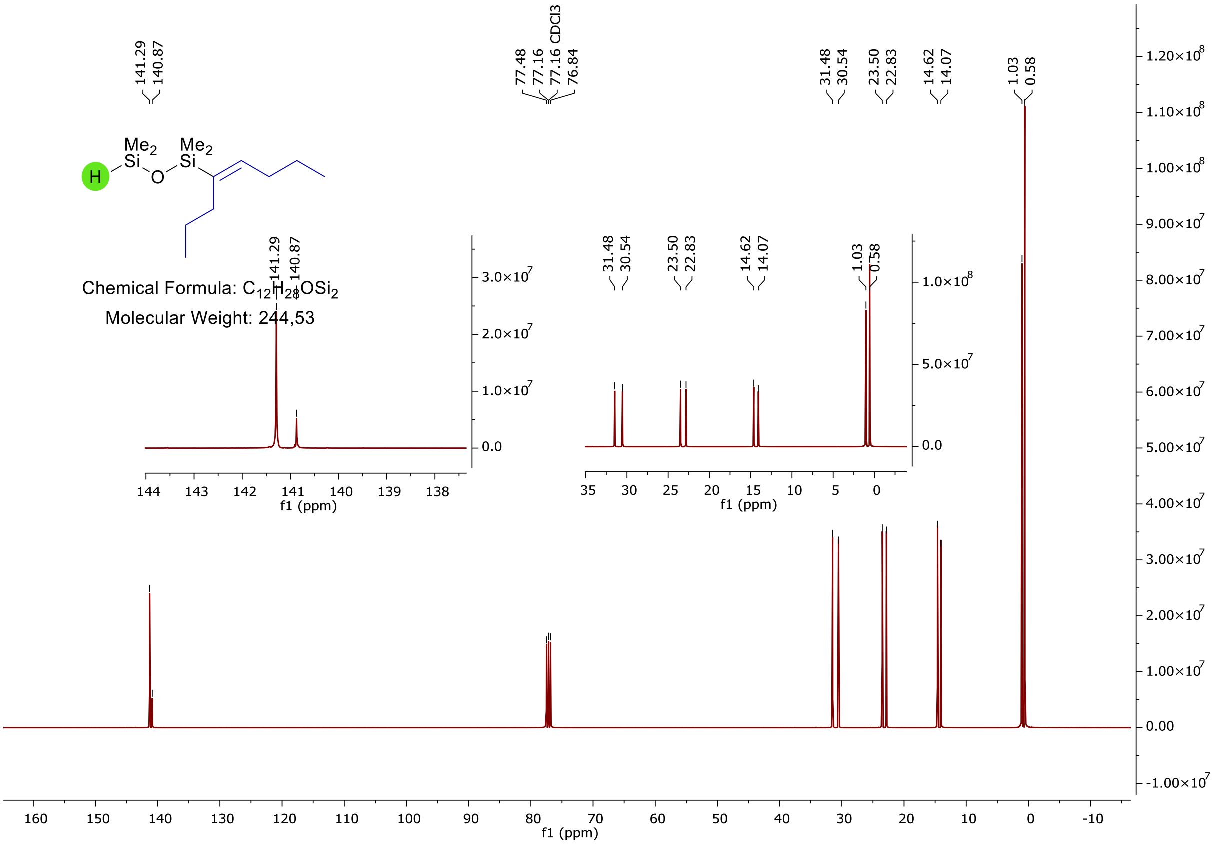

Figure S17. ^{13}C NMR spectrum of **3c**.

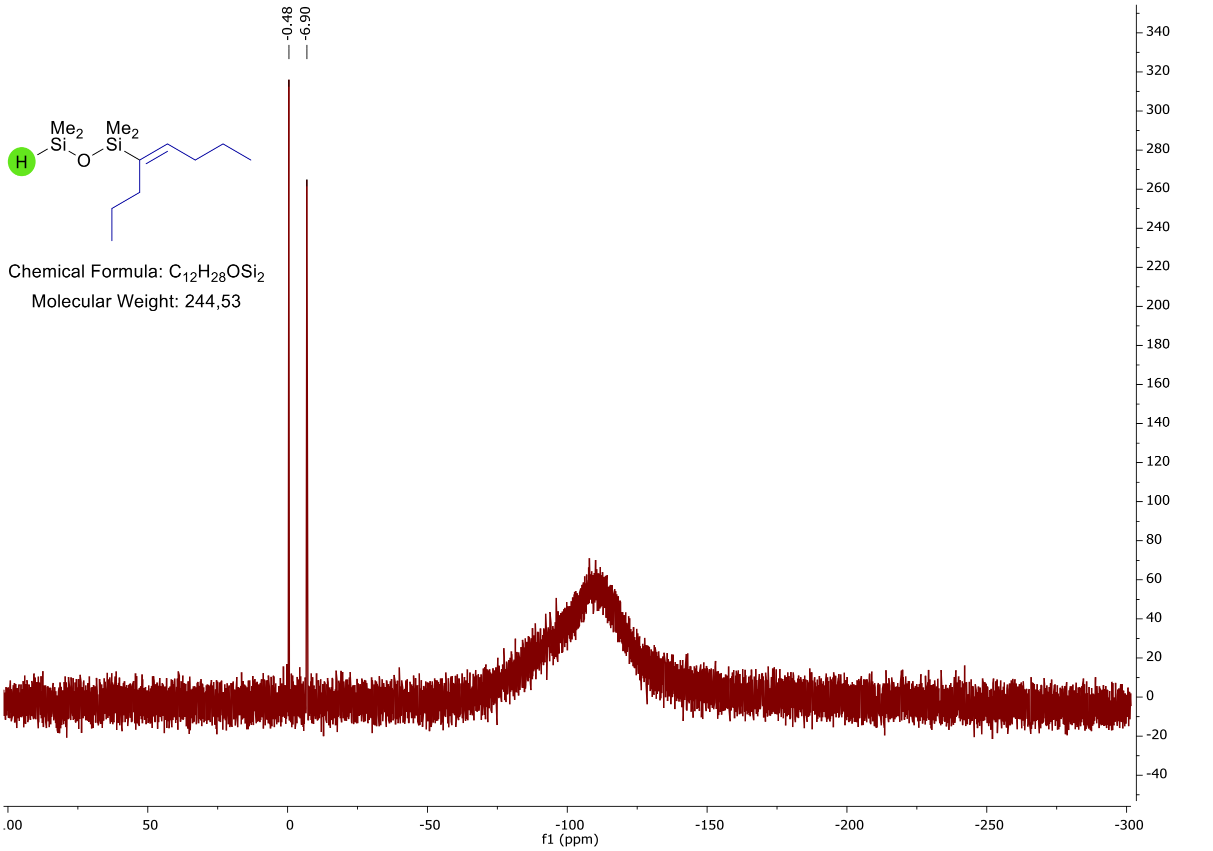

Figure S18. ^{29}Si NMR spectrum of **3c**.

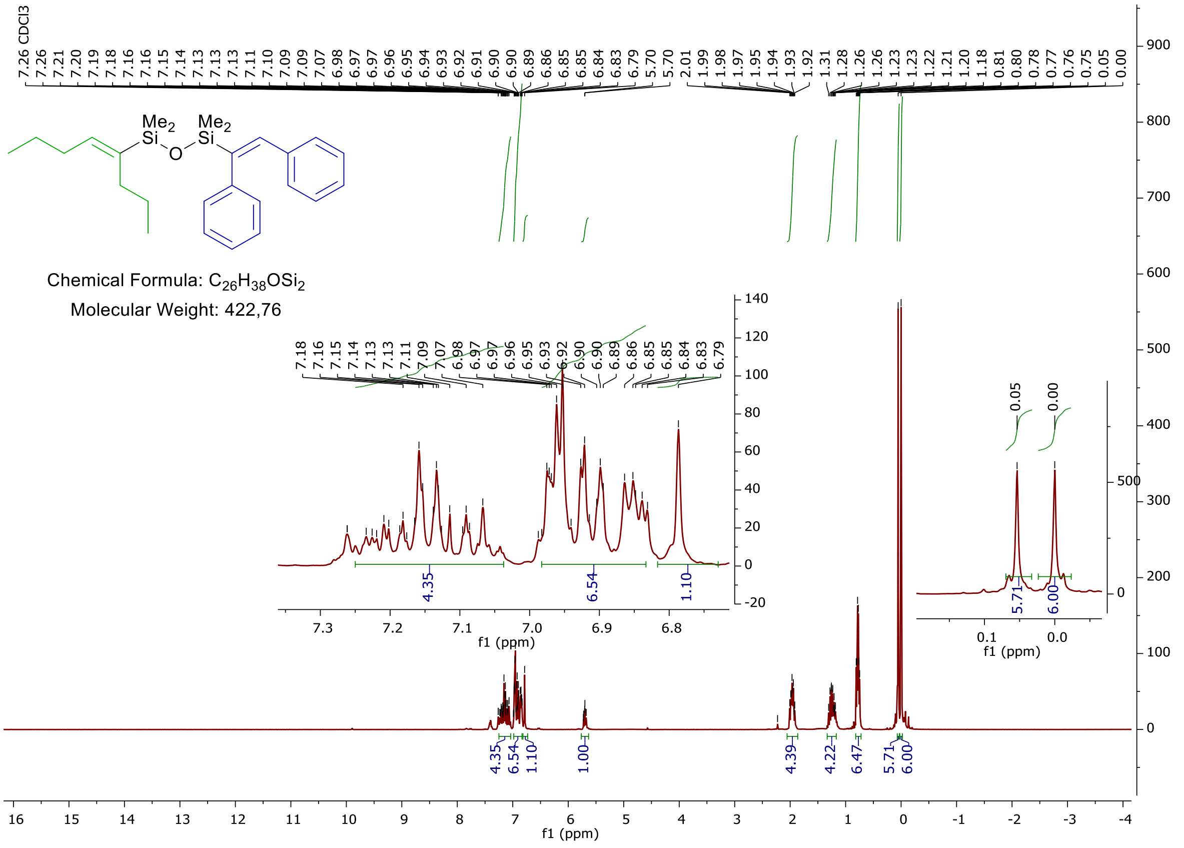

Figure S19. ^{1}H NMR spectrum of **4a**.

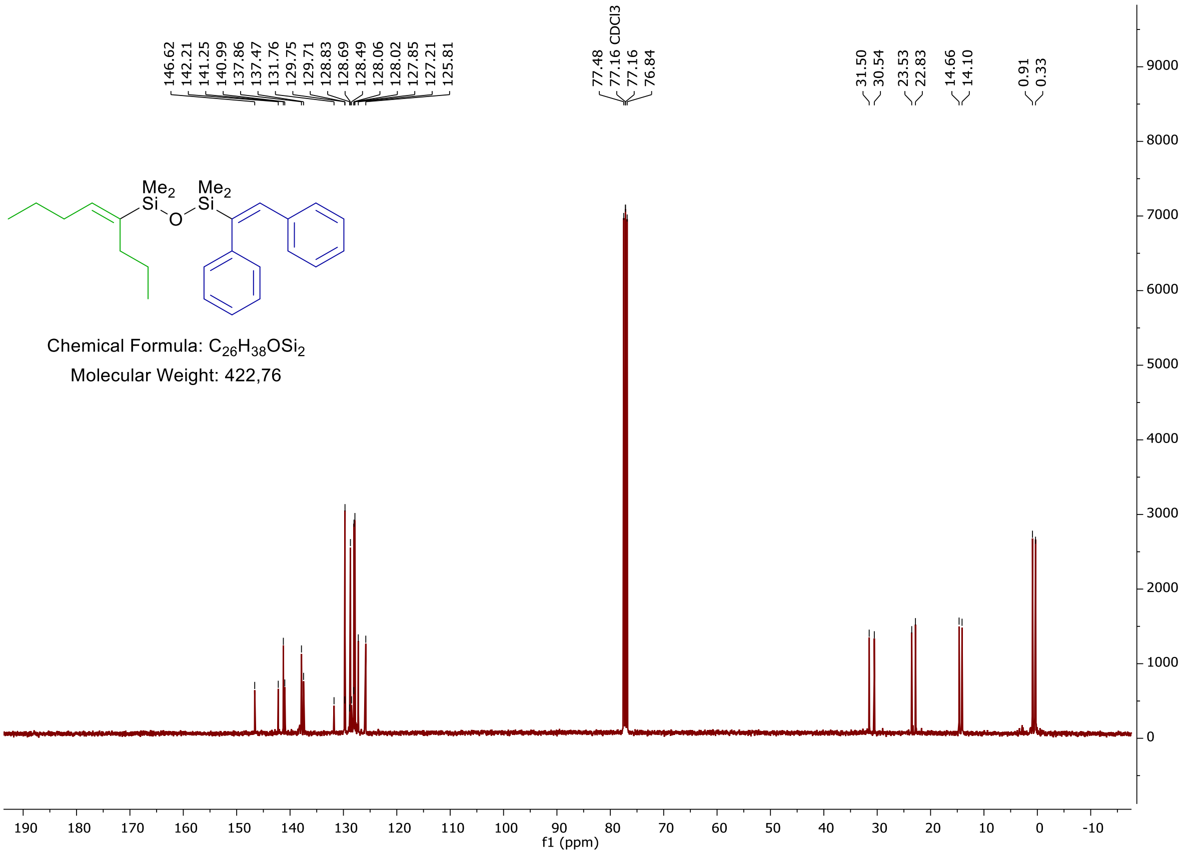

Figure S20. ^{13}C NMR spectrum of **4a**.

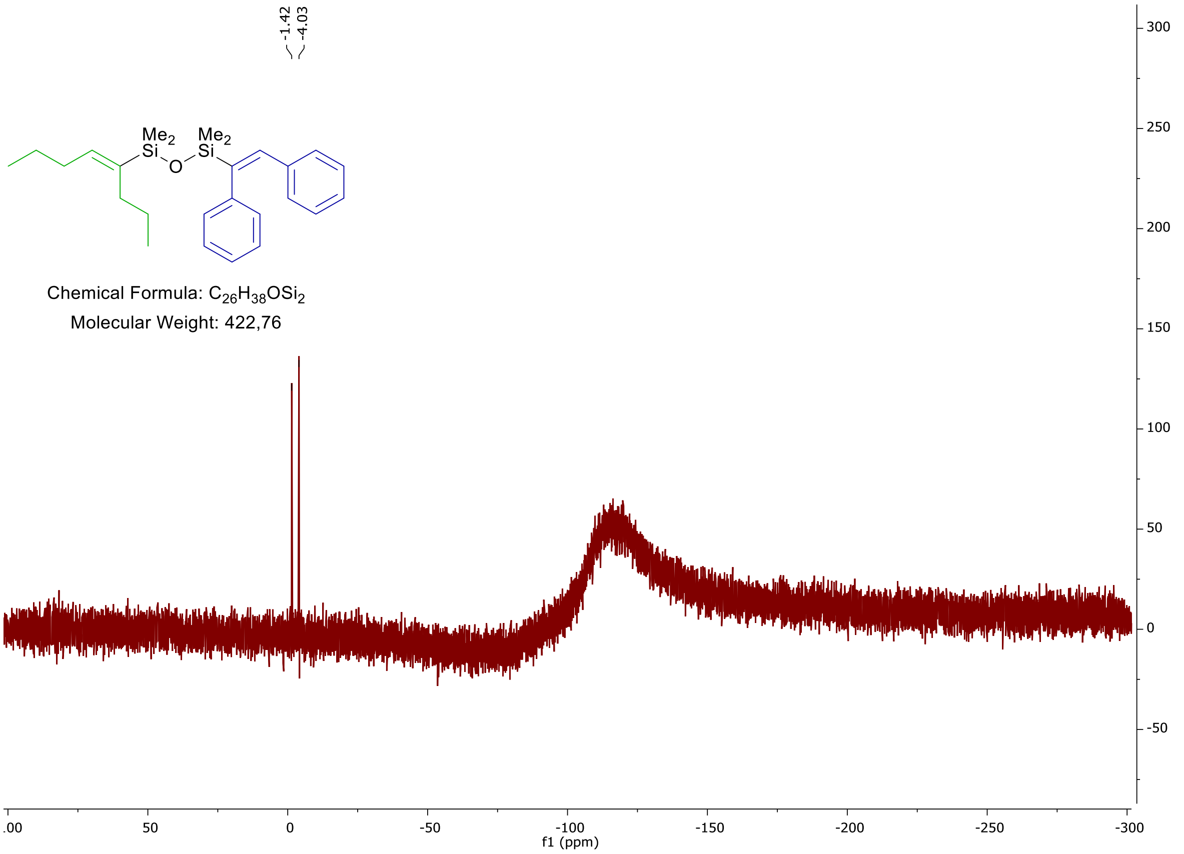

Figure S21. ^{29}Si NMR spectrum of **4a**.

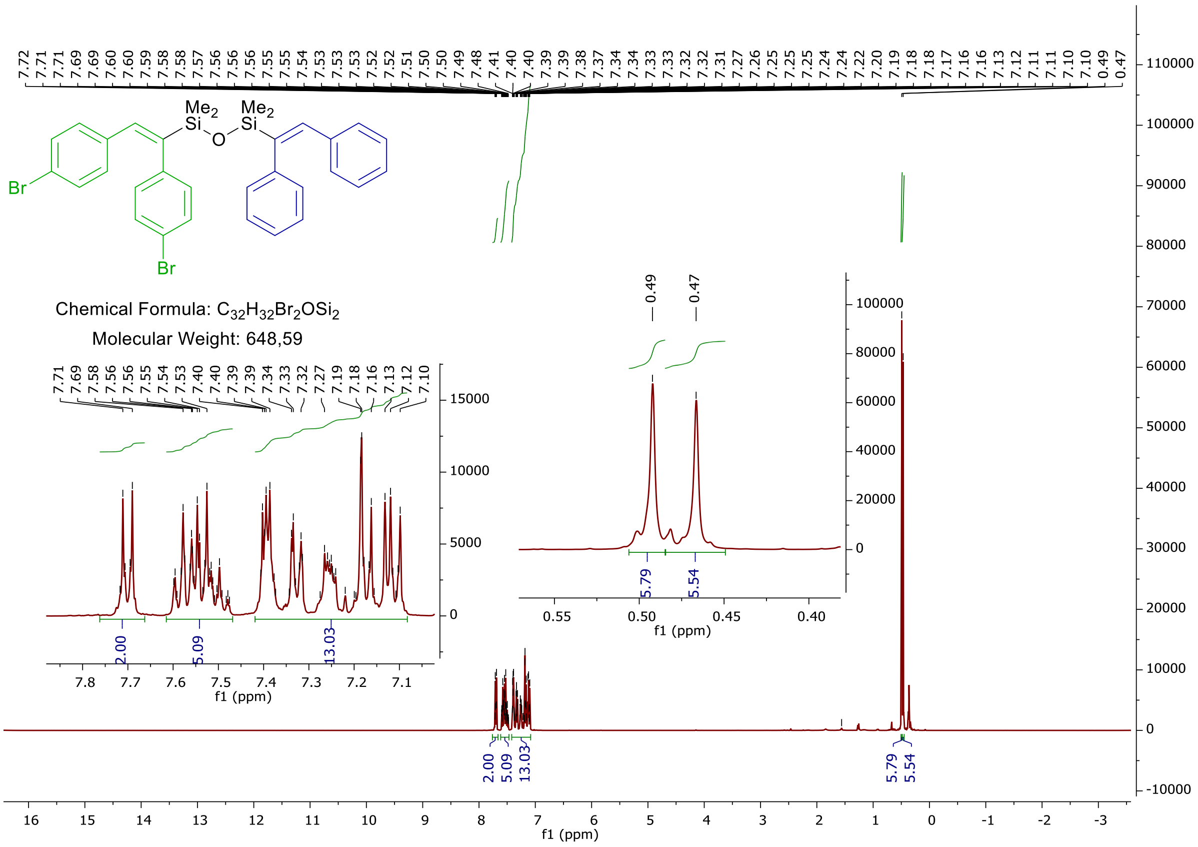

Figure S22. ^{1}H NMR spectrum of **4b**.

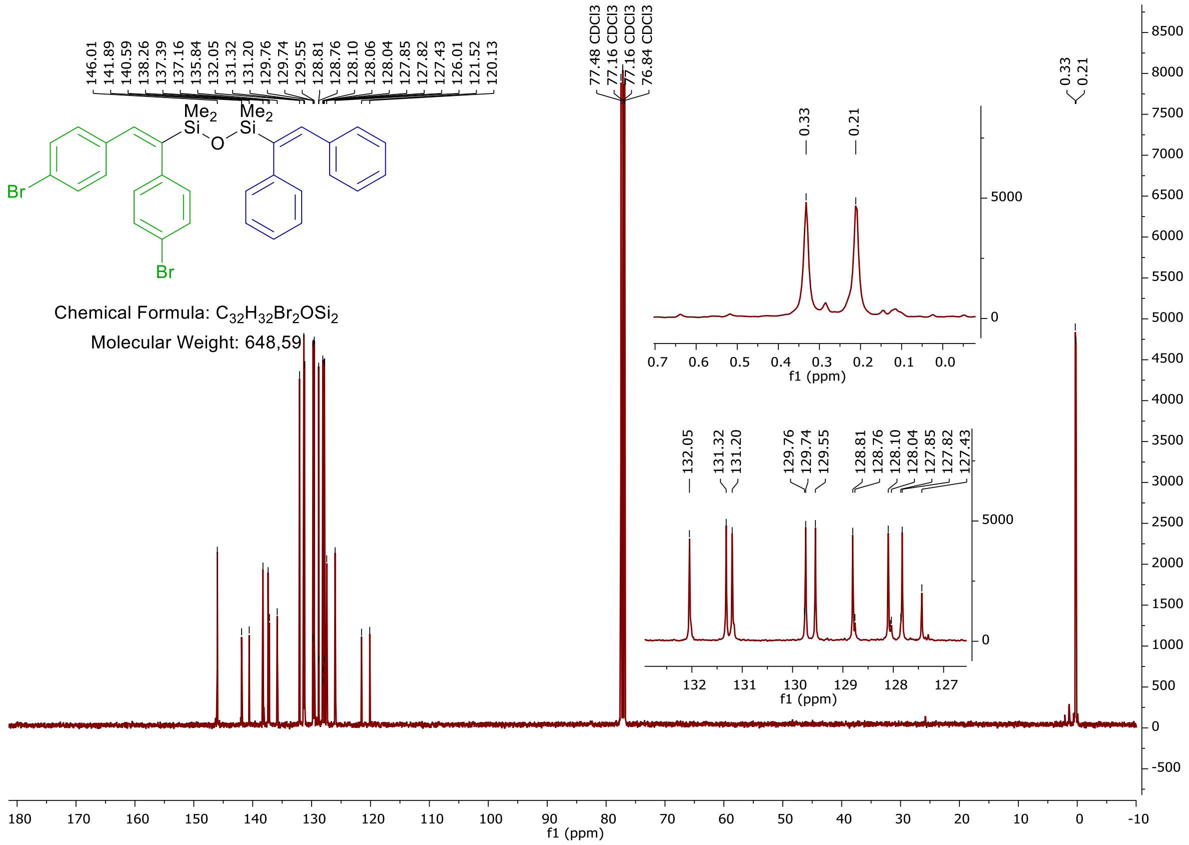

Figure S23. ^{13}C NMR spectrum of **4b**.

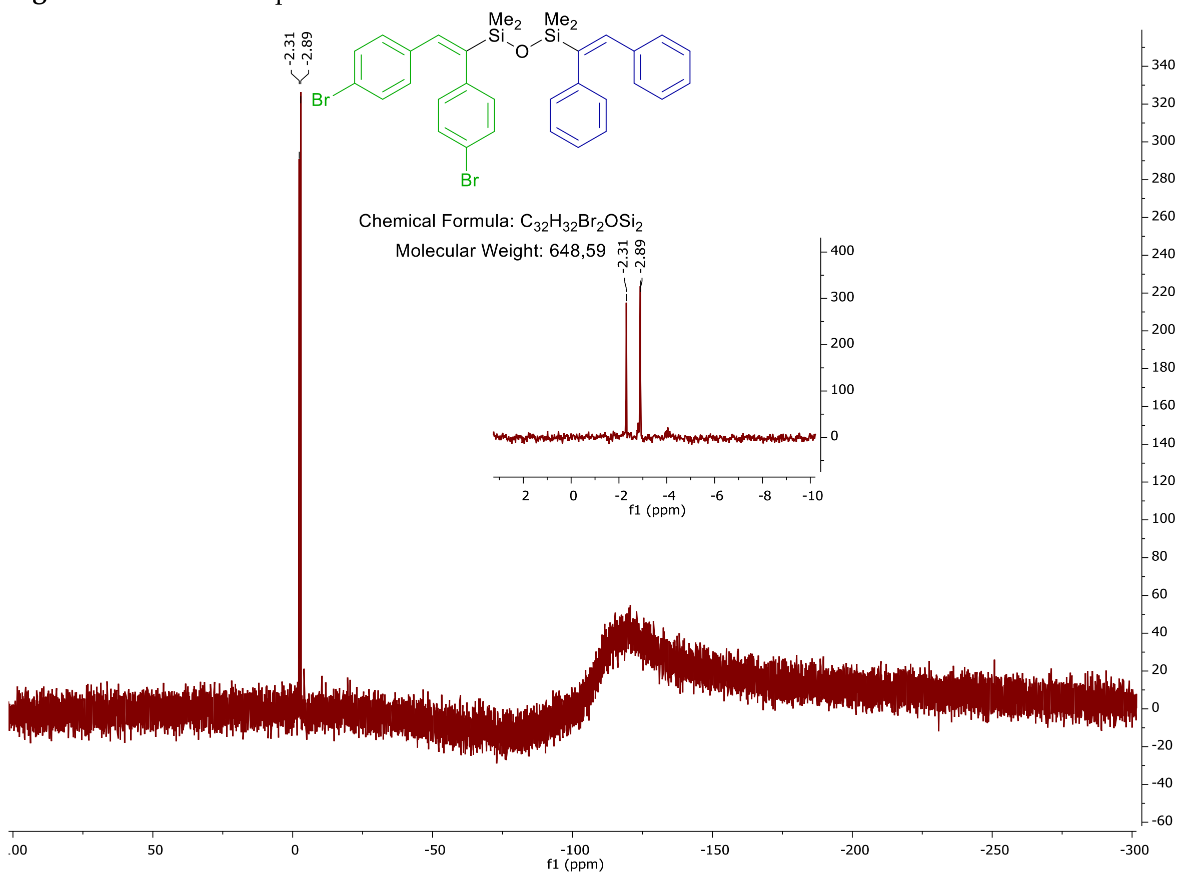

Figure S24. ^{29}Si NMR spectrum of **4b**.

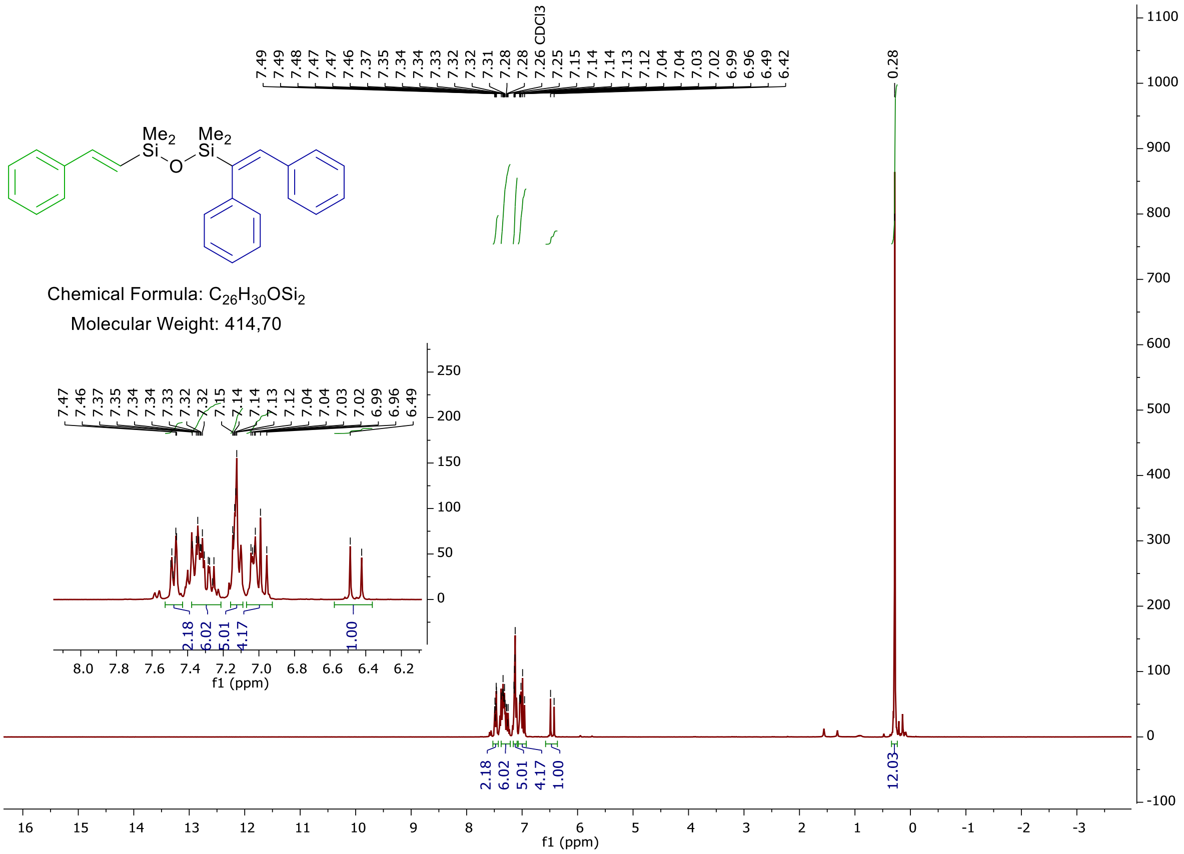

Figure S25. ^{1}H NMR spectrum of **4c.**

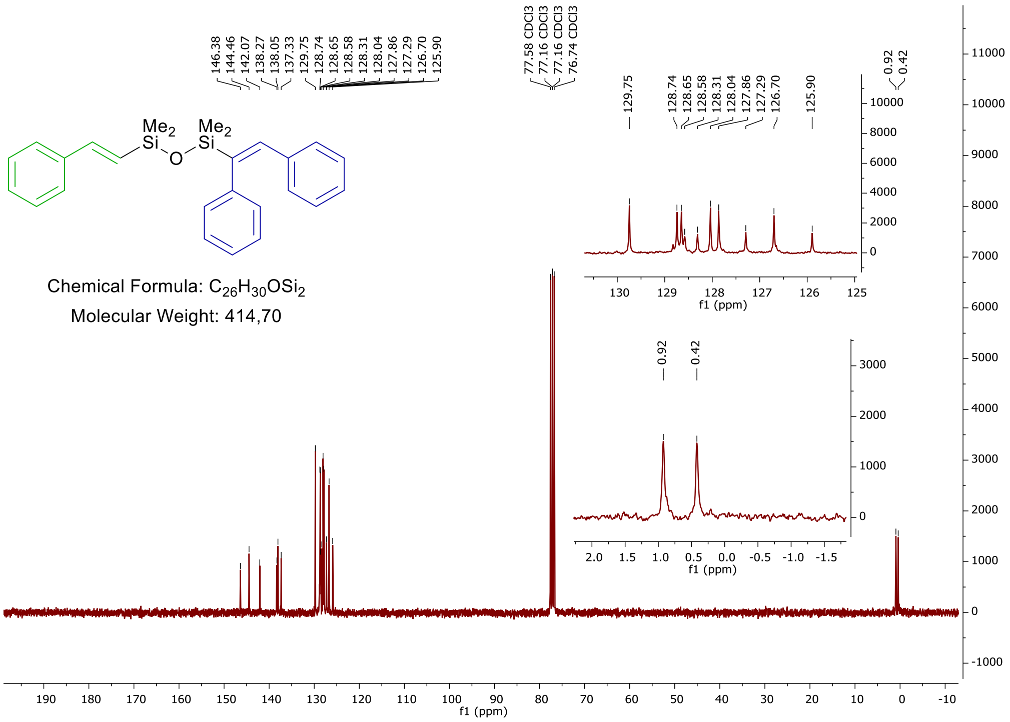

Figure S26. ^{13}C NMR spectrum of **4c.**

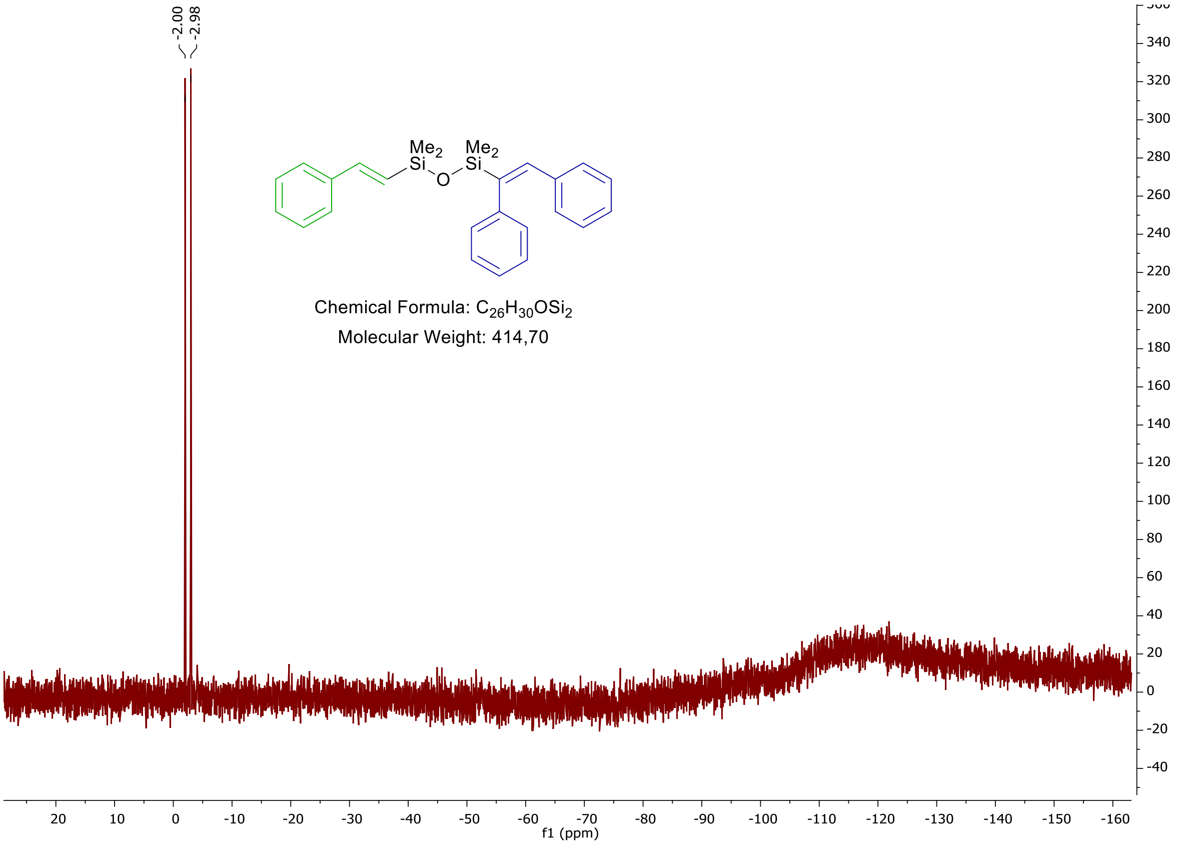

Figure S27. ^{29}Si NMR spectrum of **4c**.

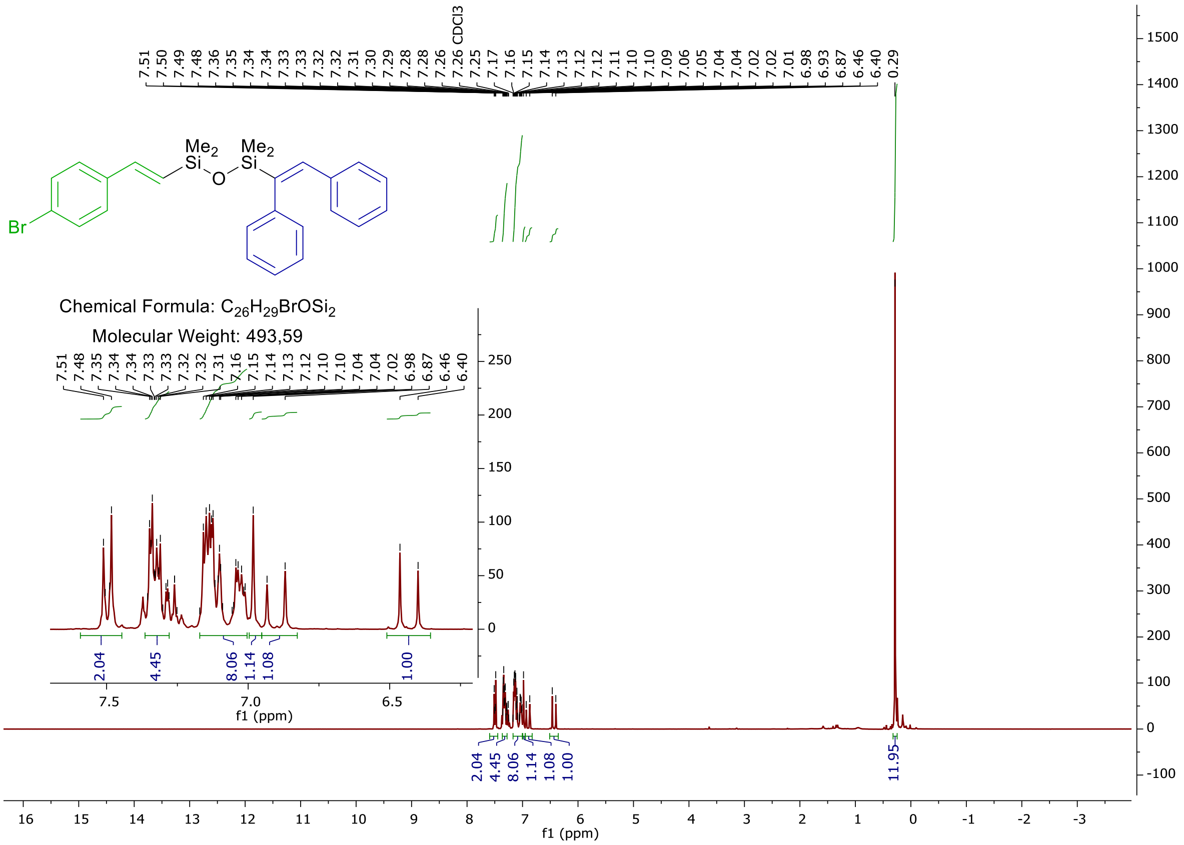

Figure S28. ^{1}H NMR spectrum of **4d**.

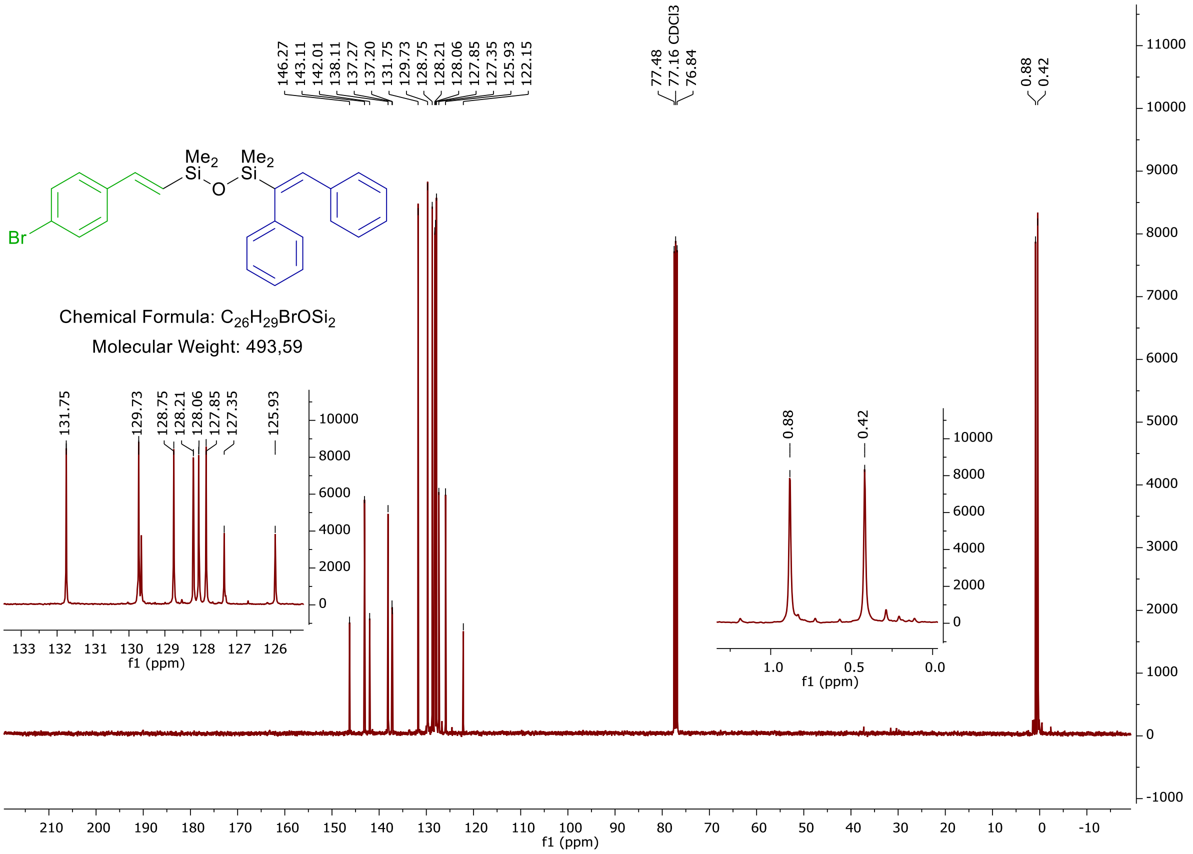

Figure S29. ^{13}C NMR spectrum of **4d**.

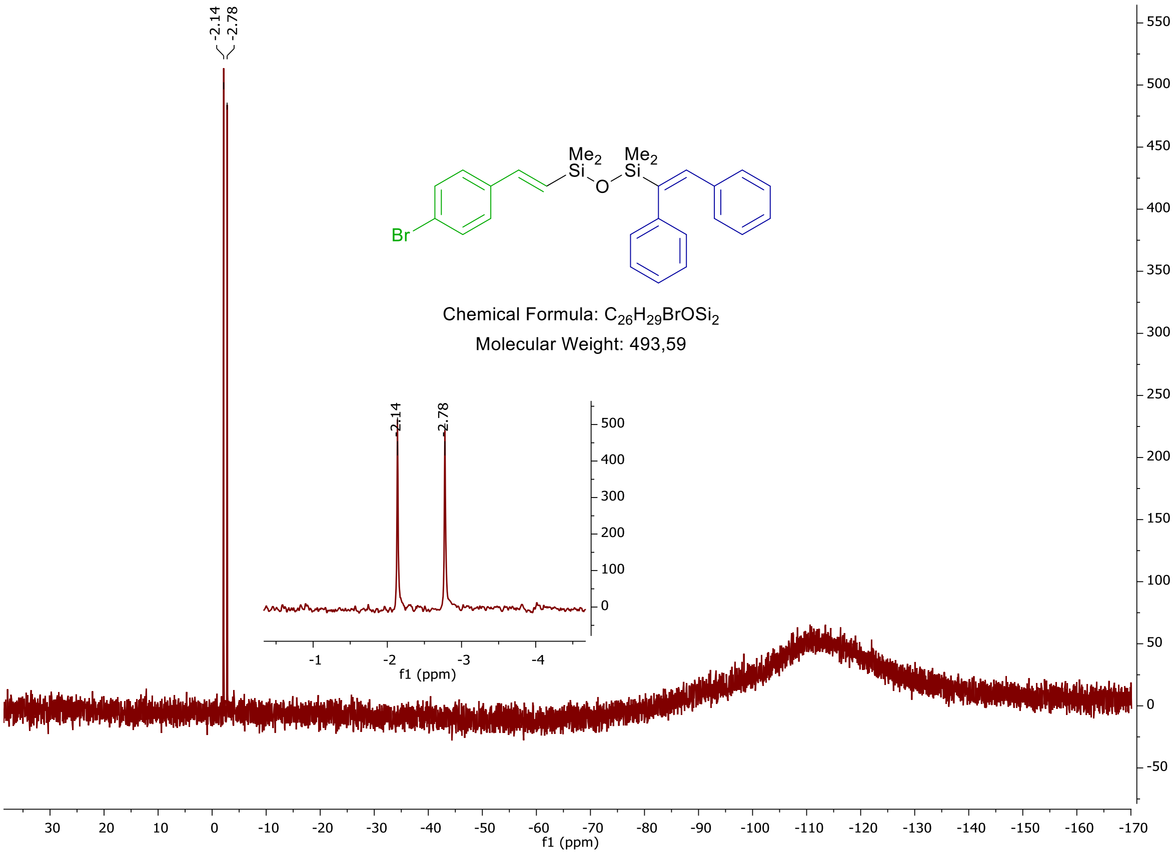

Figure S30. ^{29}Si NMR spectrum of **4d**.

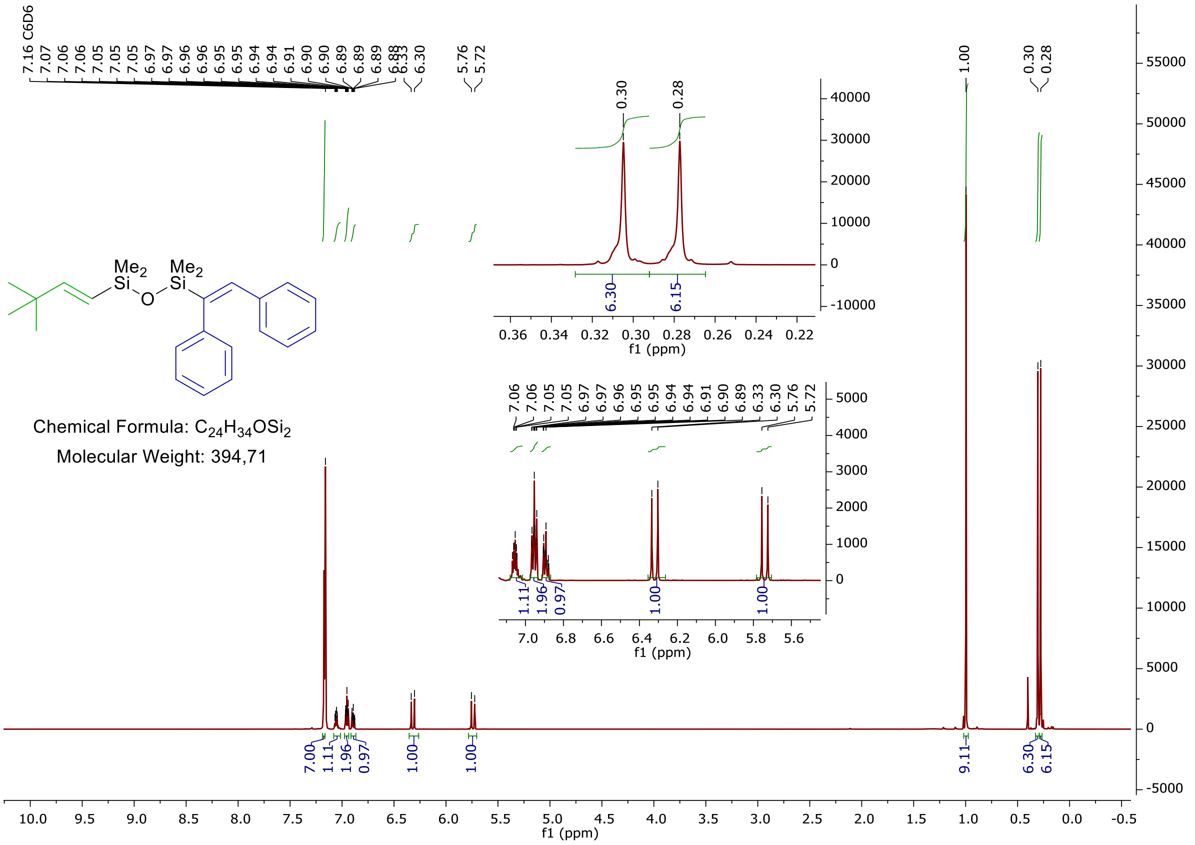

Figure S31. ^{1}H NMR spectrum of **4e**.

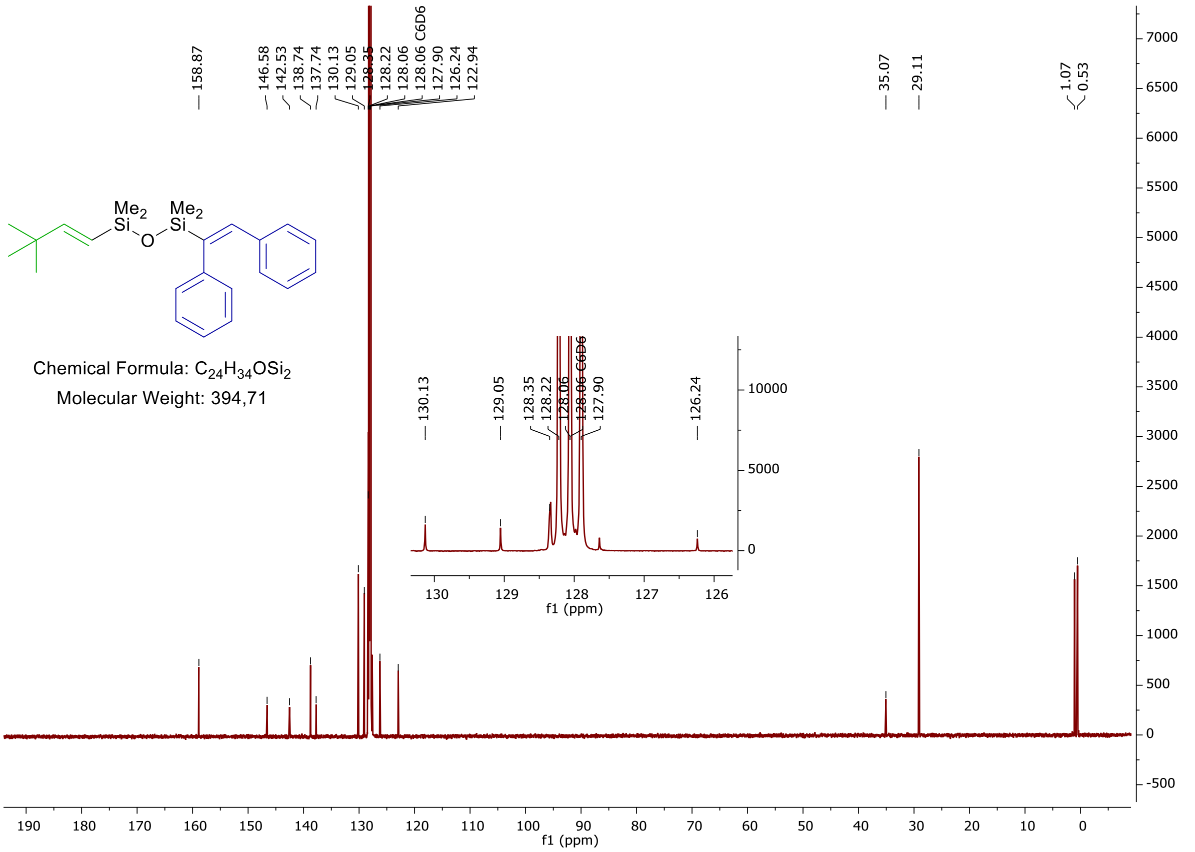

Figure S32. ^{13}C NMR spectrum of **4e**.

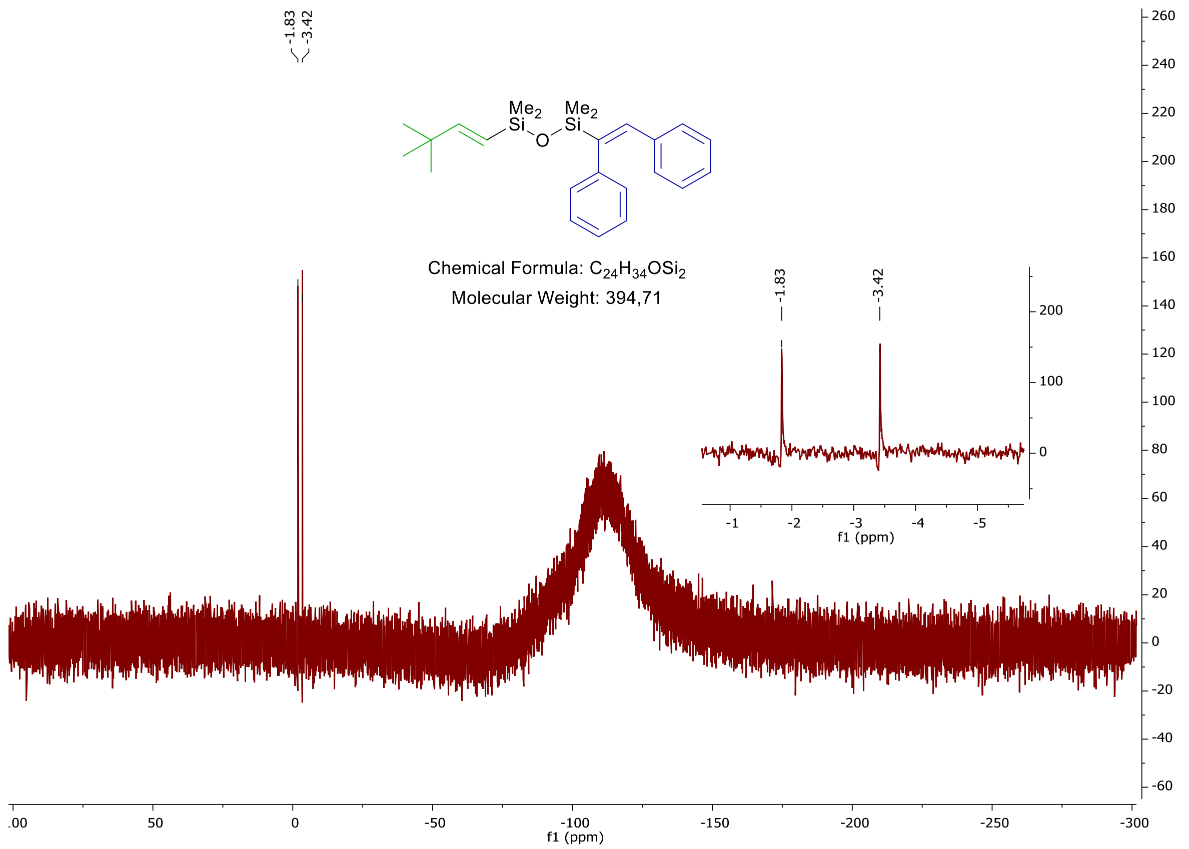

Figure S33. ^{29}Si NMR spectrum of **4e**.

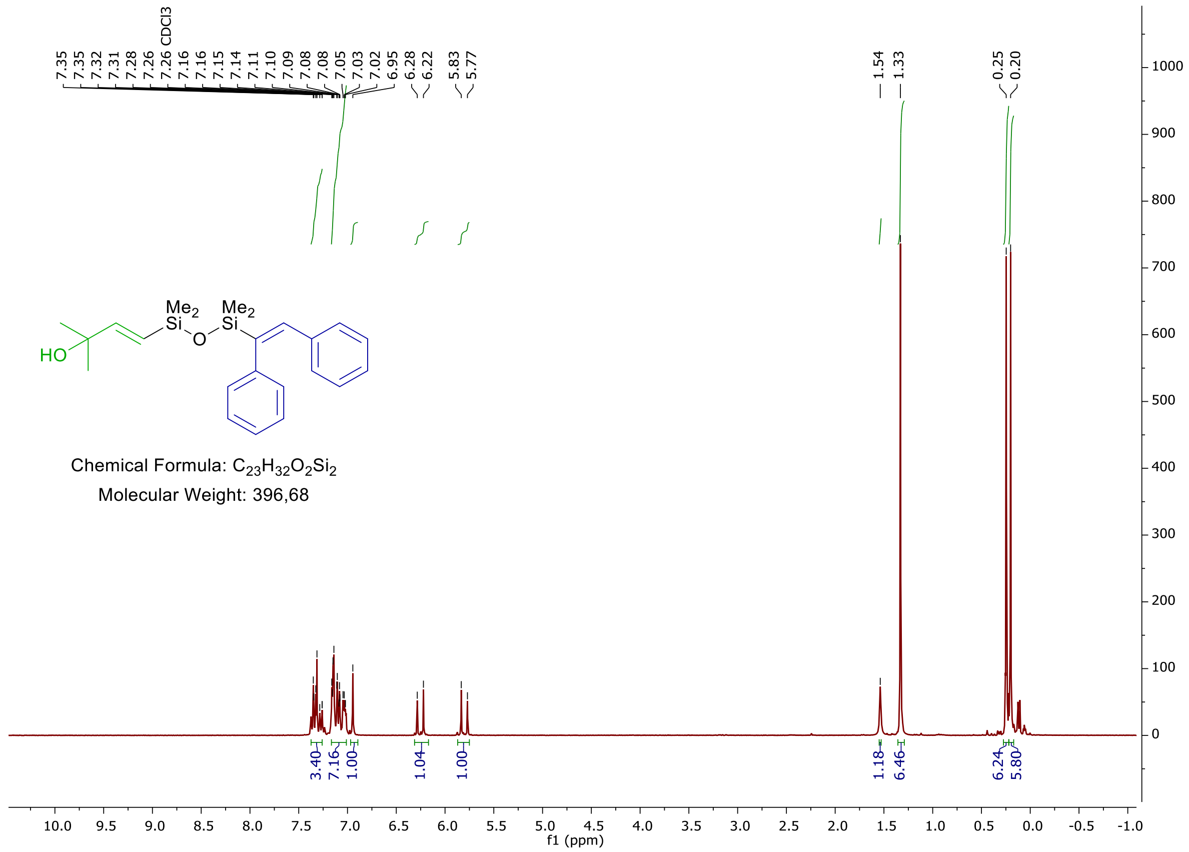

Figure S34. ^{1}H NMR spectrum of **4g**.

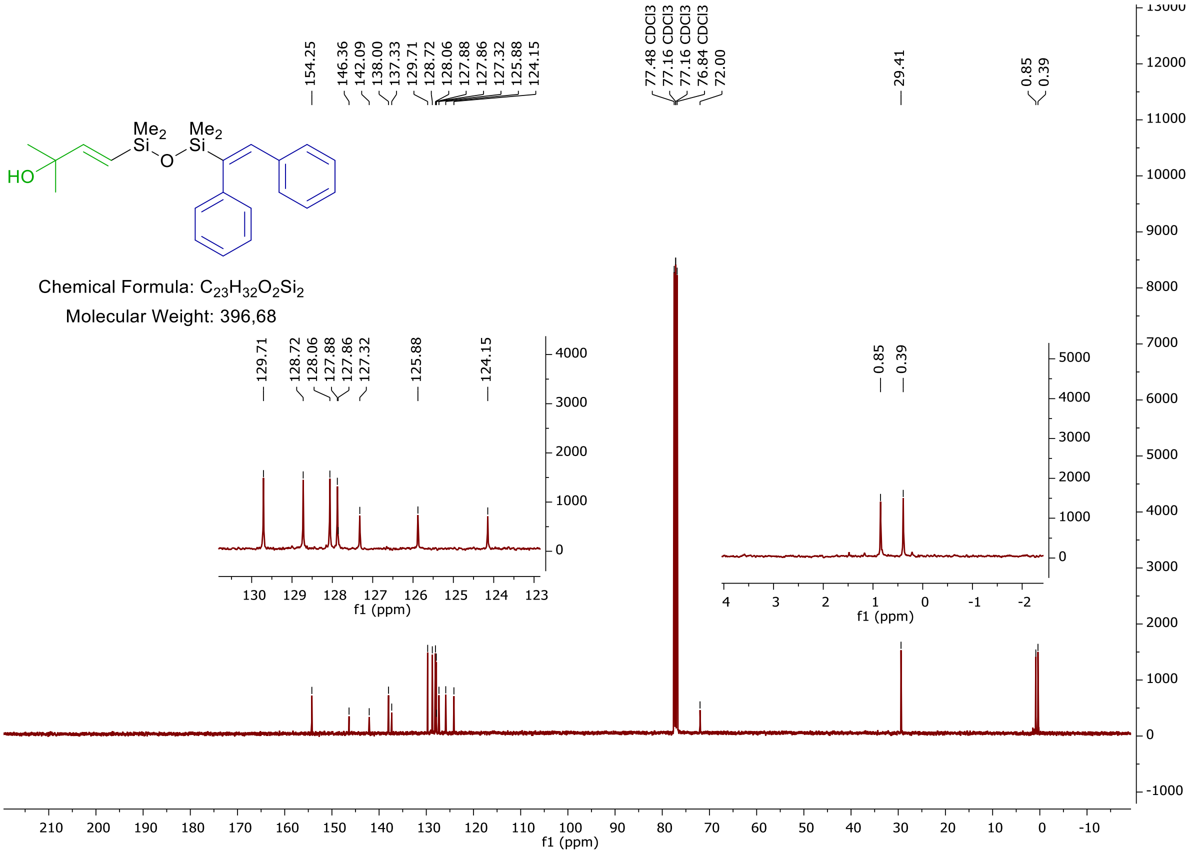

Figure S35. ^{13}C NMR spectrum of **4g**.

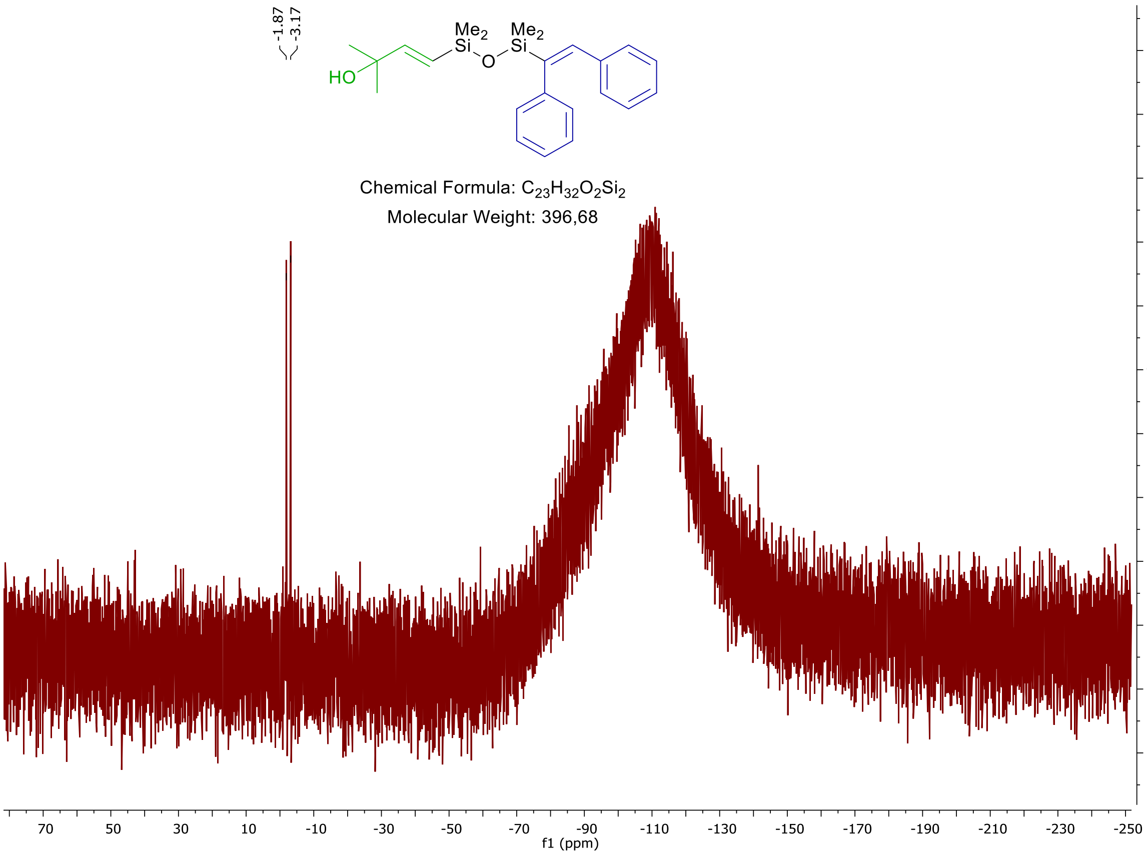

Figure S36. ^{29}Si NMR spectrum of **4g**.

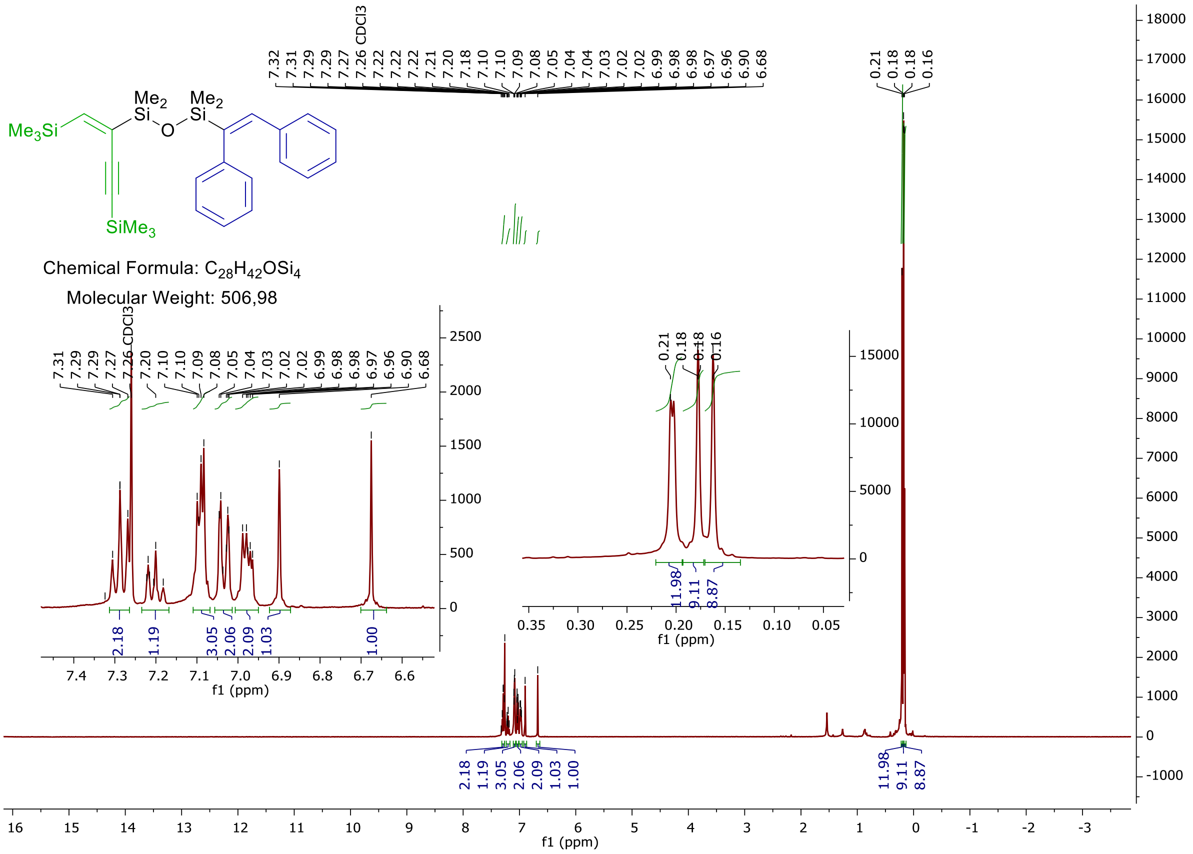

Figure S37. ^{1}H NMR spectrum of **4h.**

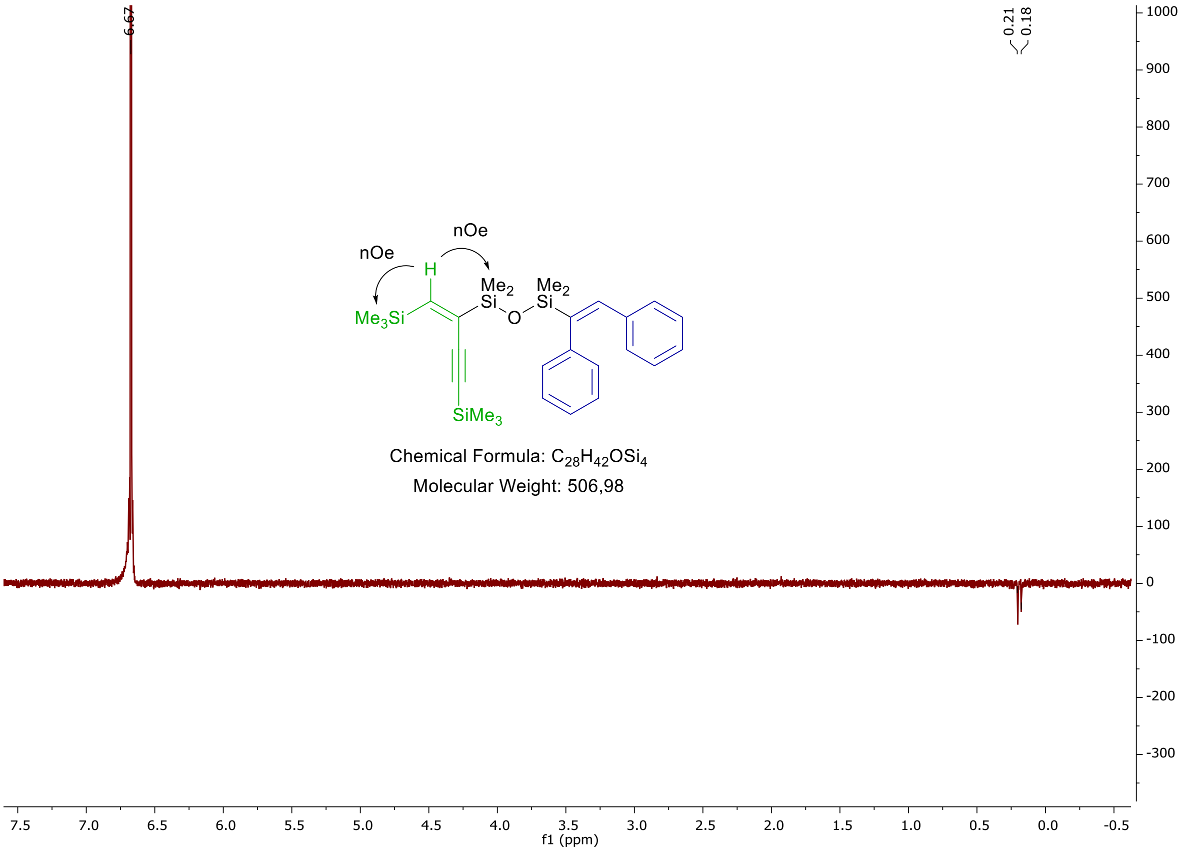

Figure S38. 1D Selective Gradient NOESY of **4h**. Freq.: 6.68 ppm.

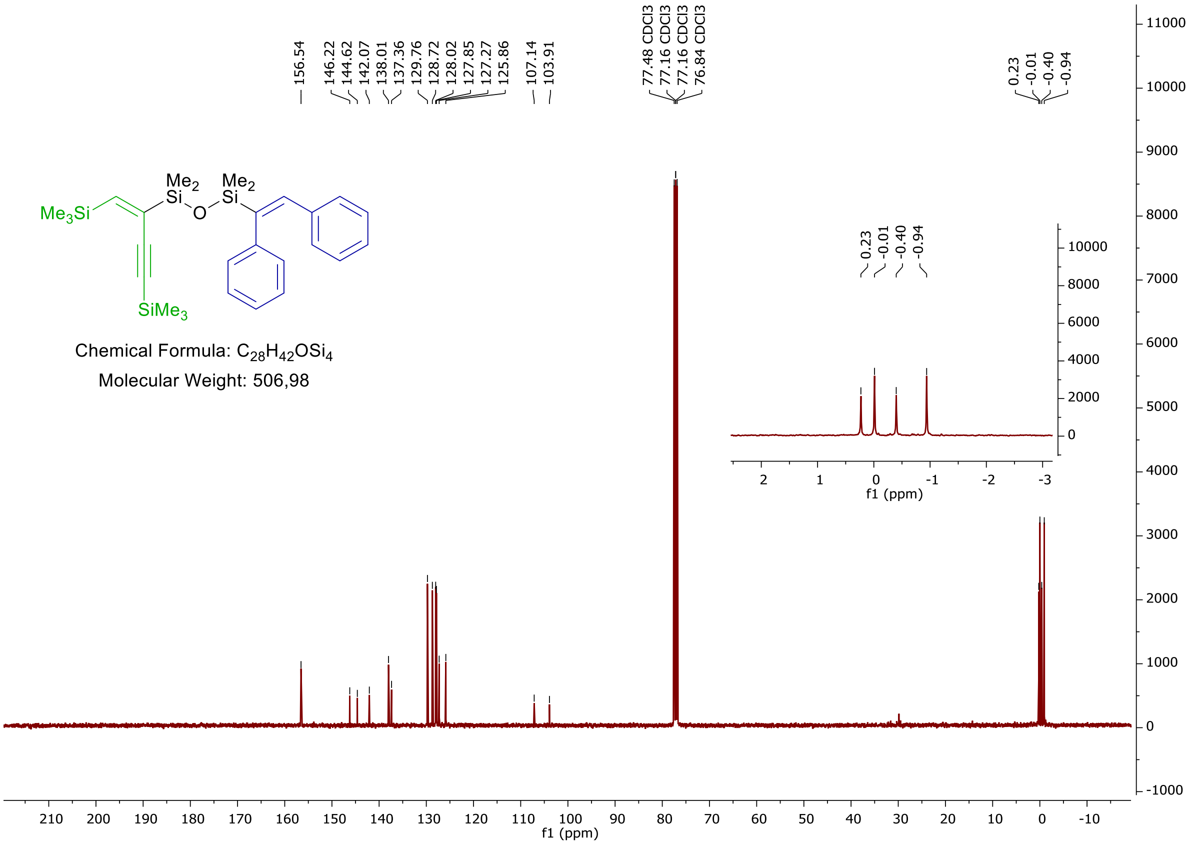

Figure S39. ^{13}C NMR spectrum of **4h.**

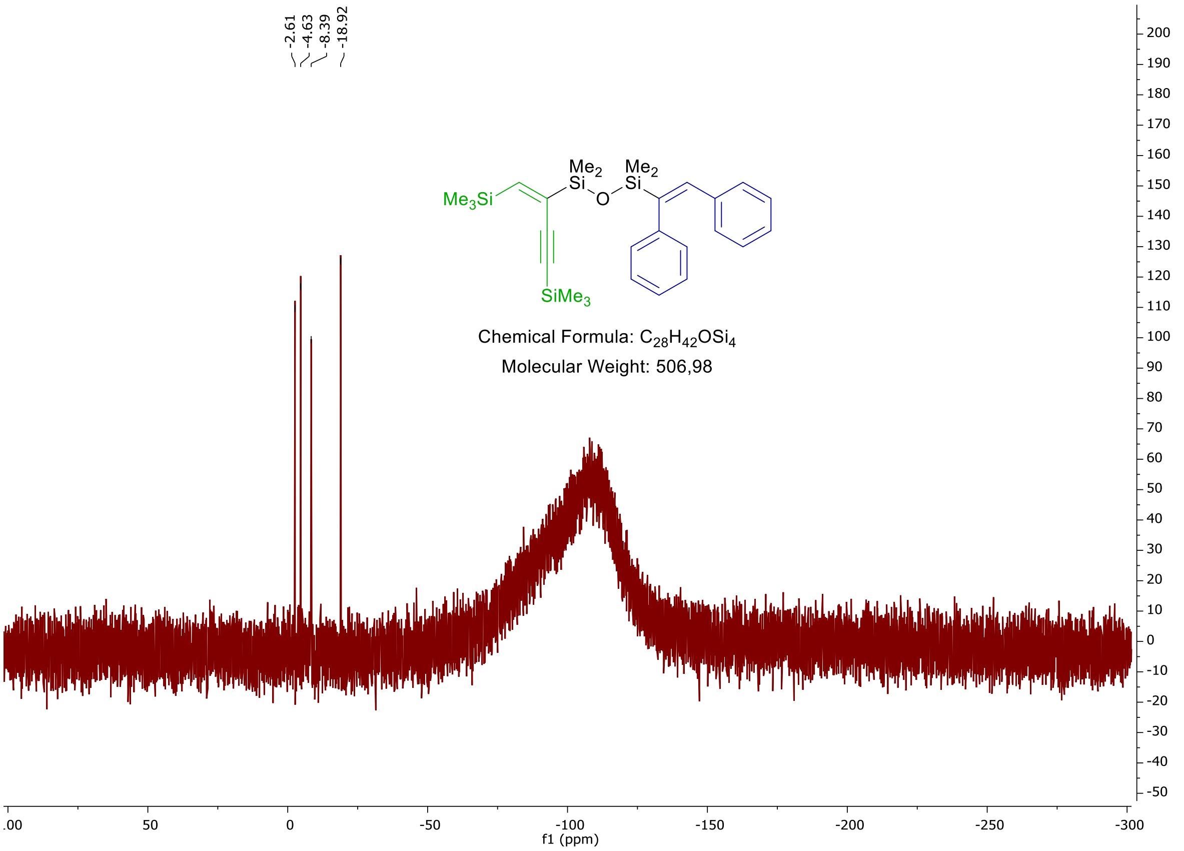

Figure S40. ^{29}Si NMR spectrum **4h.**

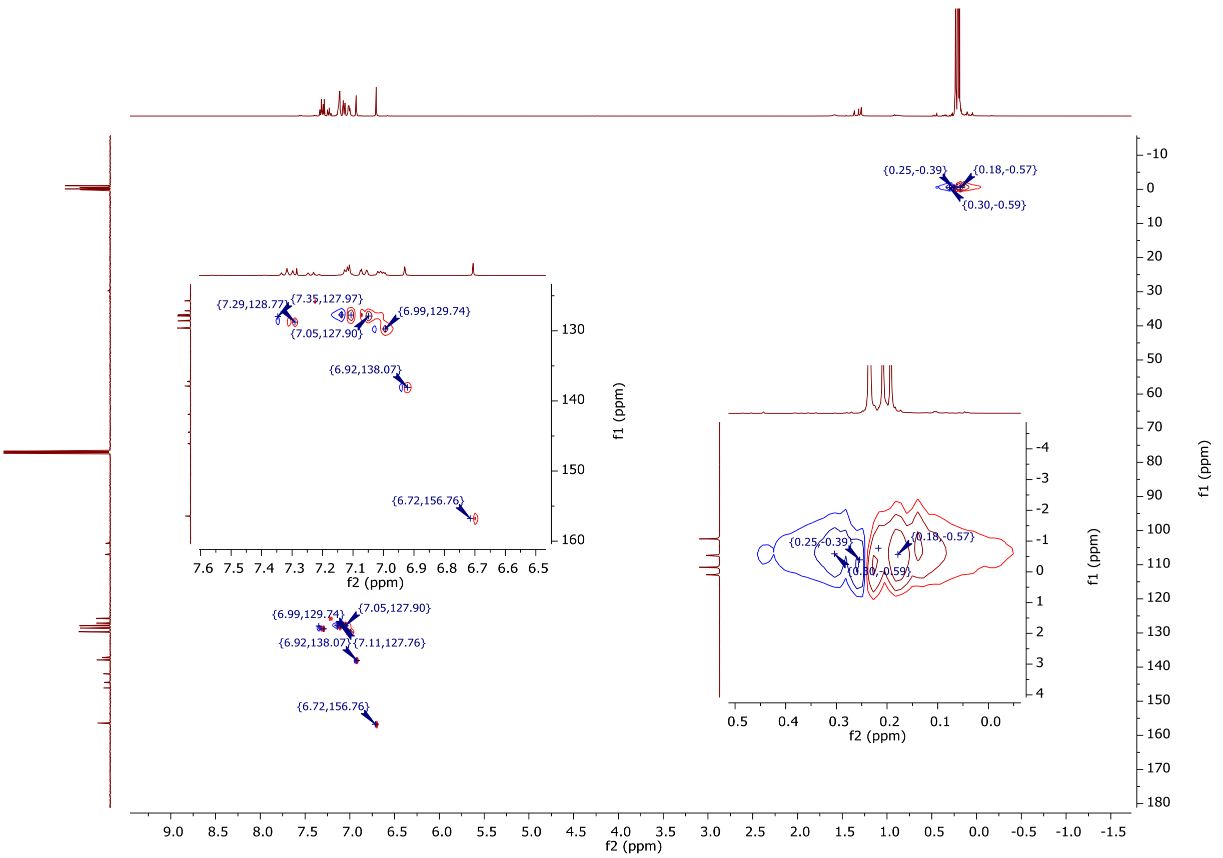

Figure S41. 2D HSQC of **4h.**

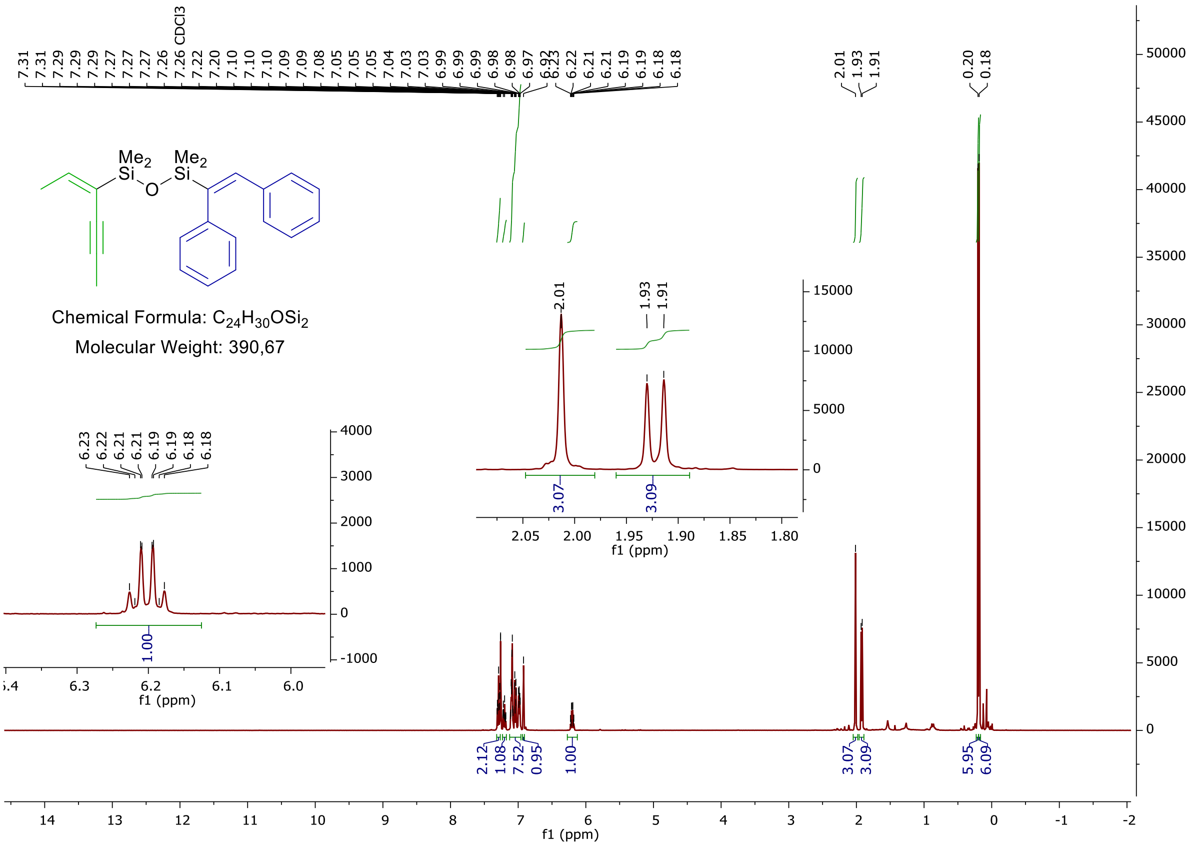

Figure S42. ^{1}H NMR spectrum of **4i.**

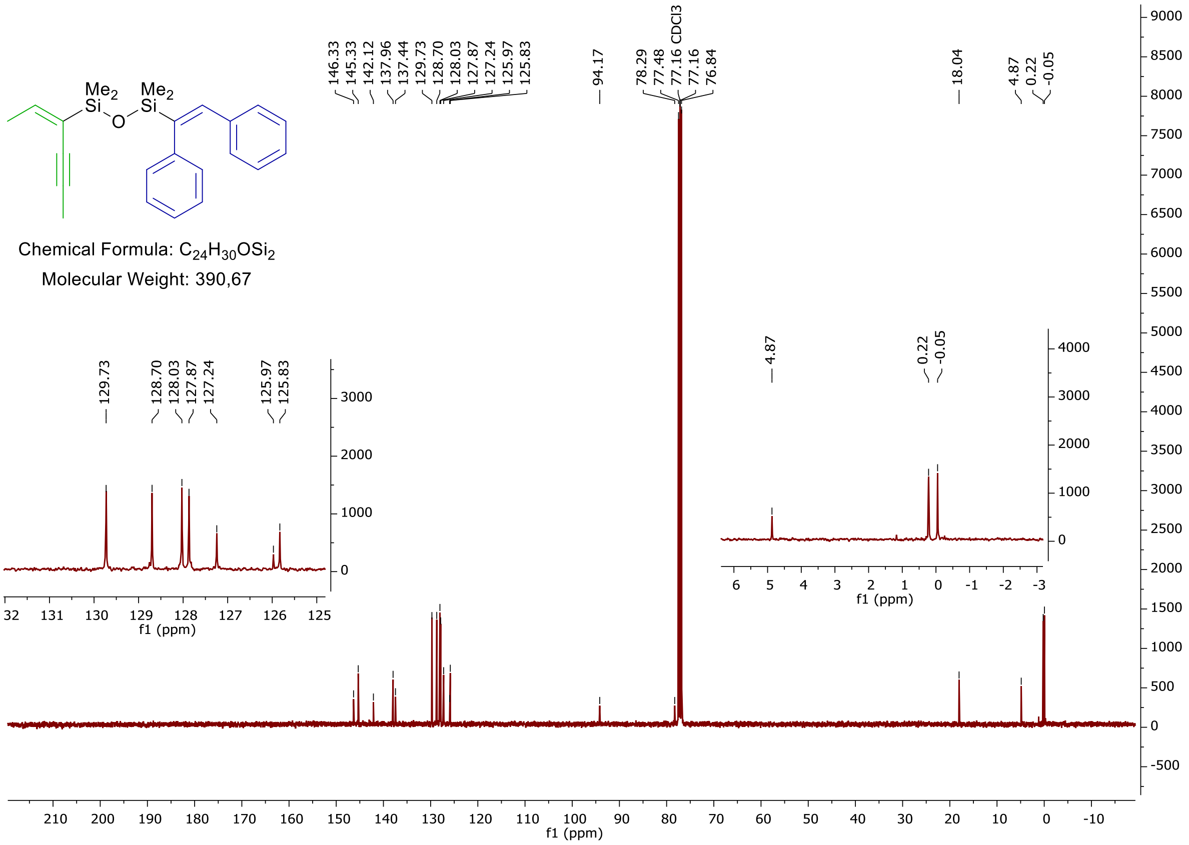

Figure S43. ^{13}C NMR spectrum of **4i**.

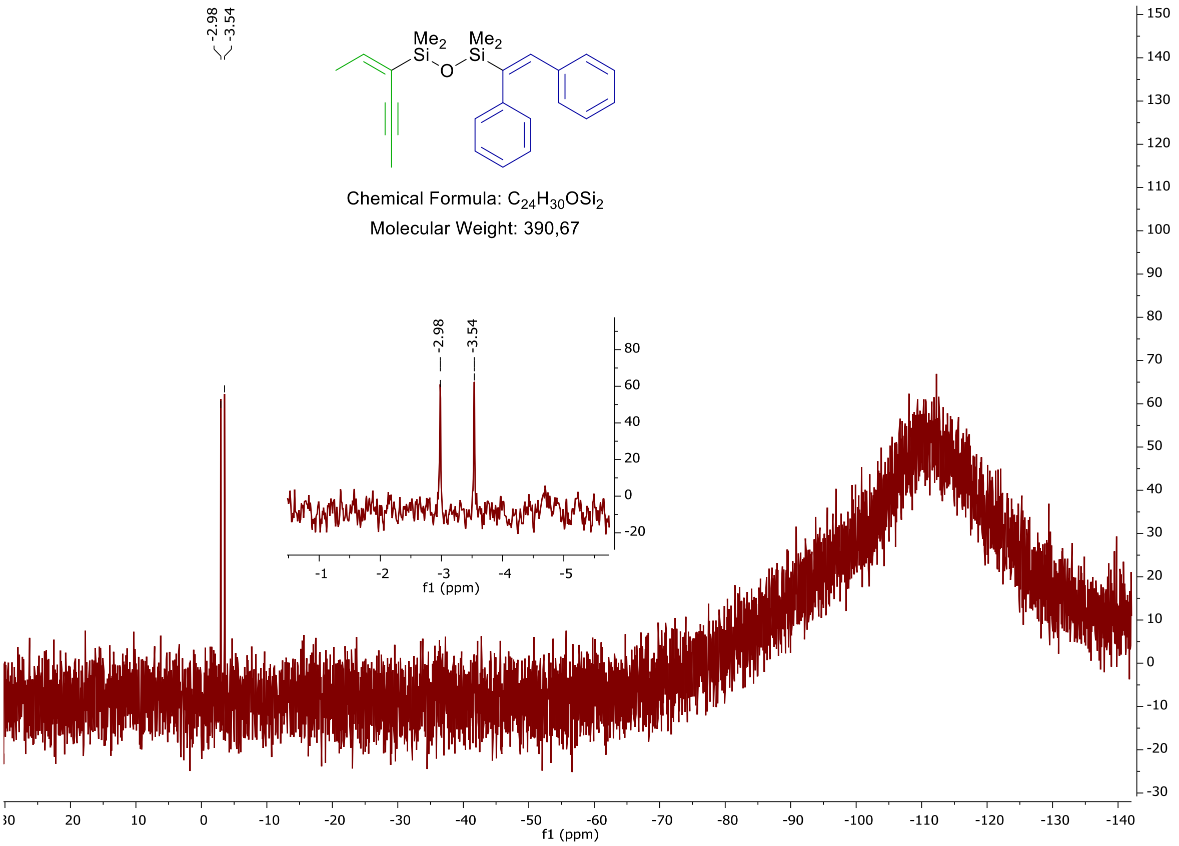

Figure S44. ^{29}Si NMR spectrum **4i**.

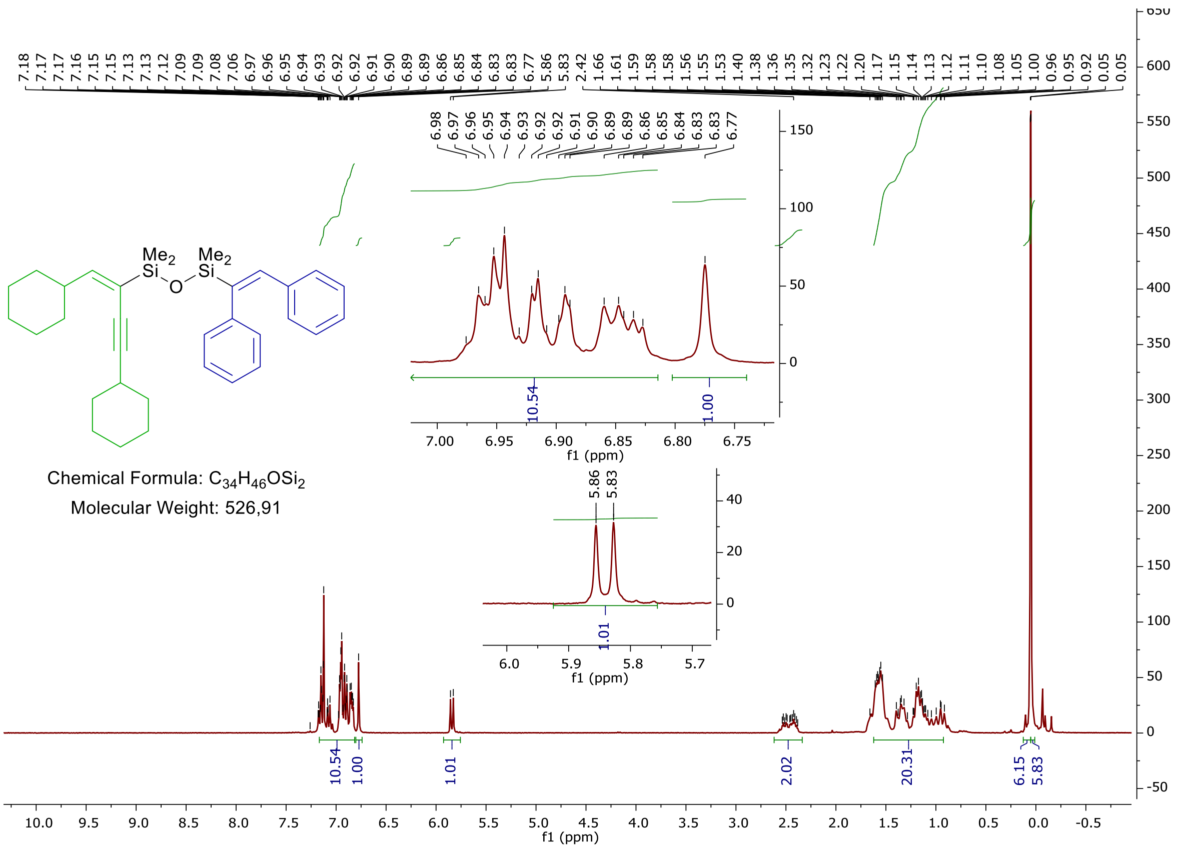

Figure S45. ^{1}H NMR spectrum of **4j**.

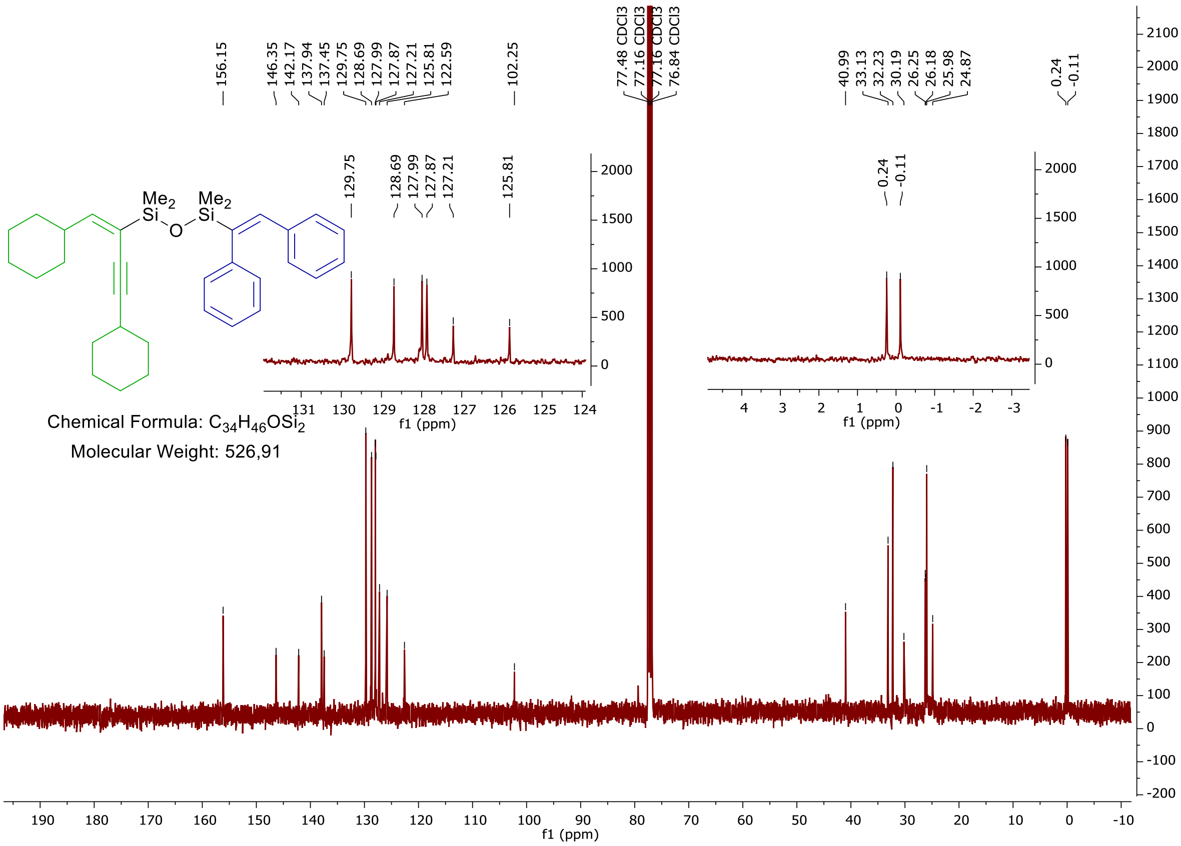

Figure S46. ^{13}C NMR spectrum of **4j**.

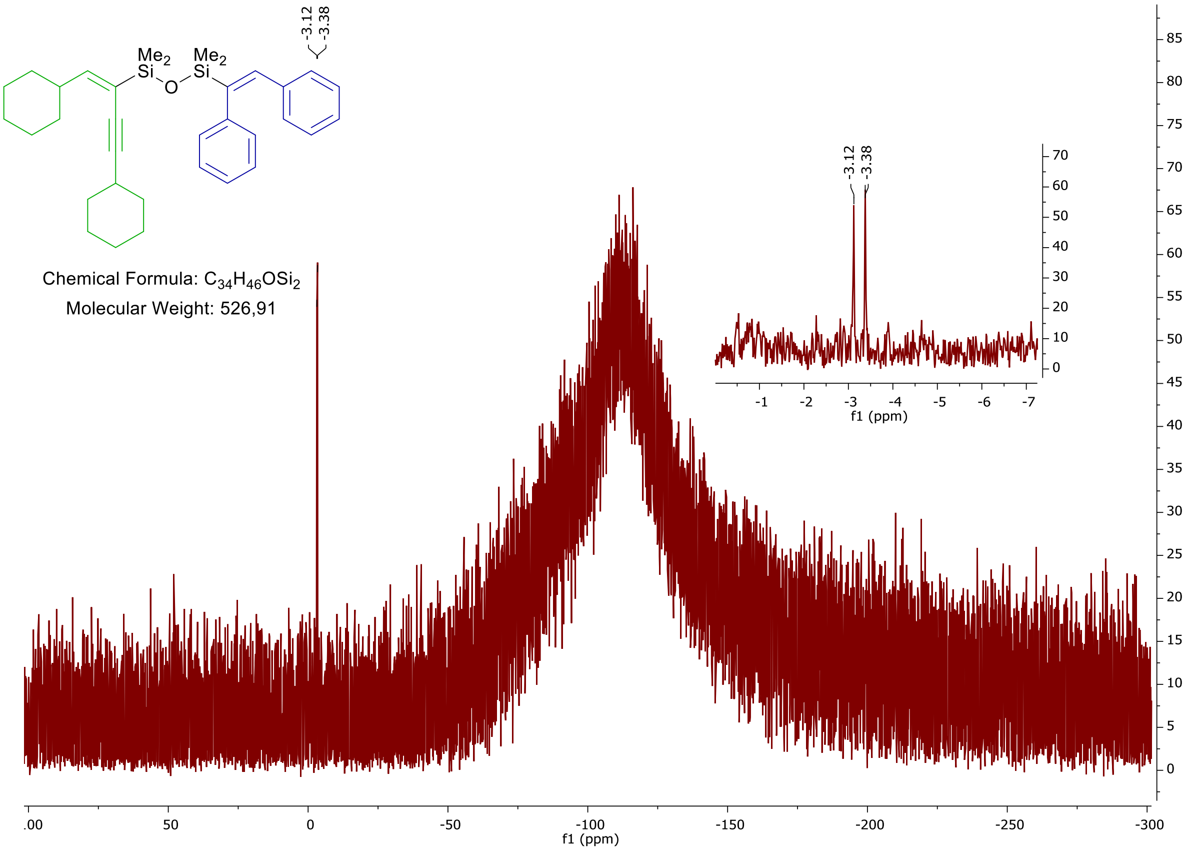

Figure S47. ^{29}Si NMR spectrum of **4j**.

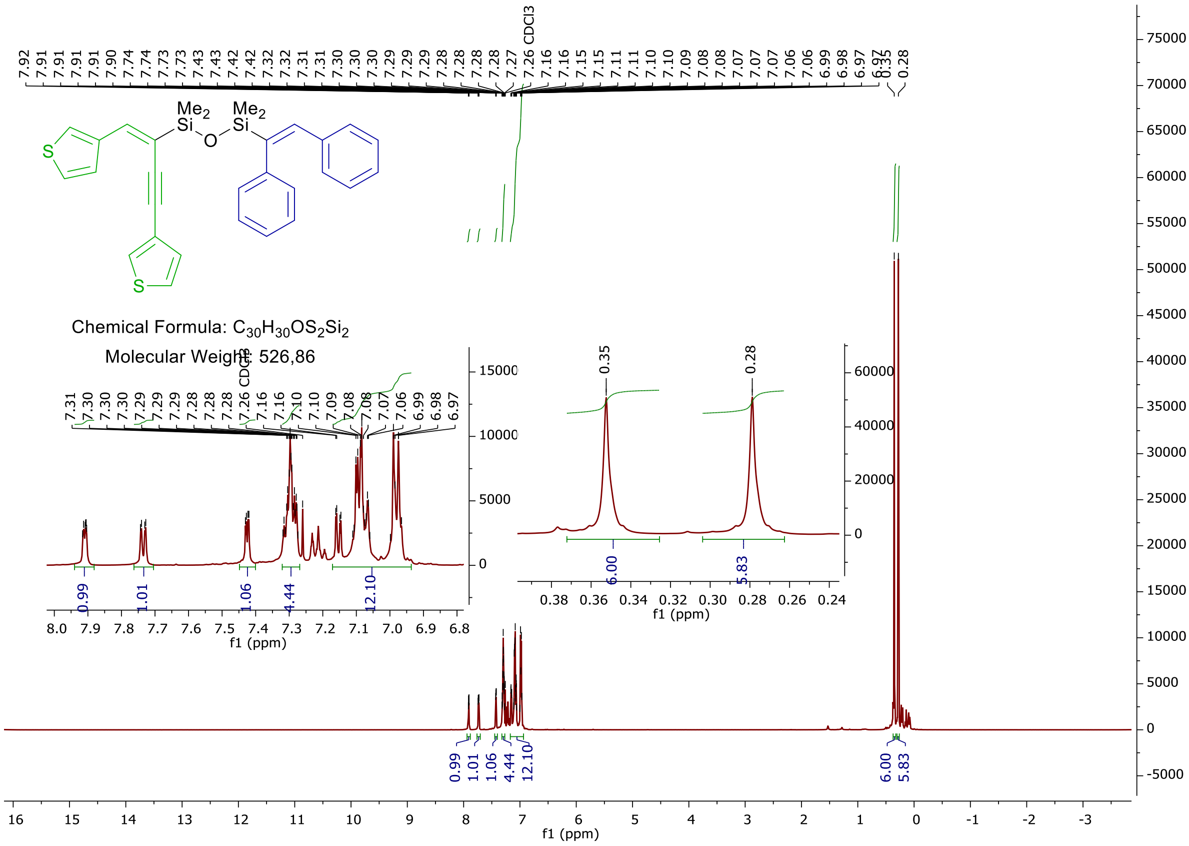

Figure S48. ^{1}H NMR spectrum of **4k.**

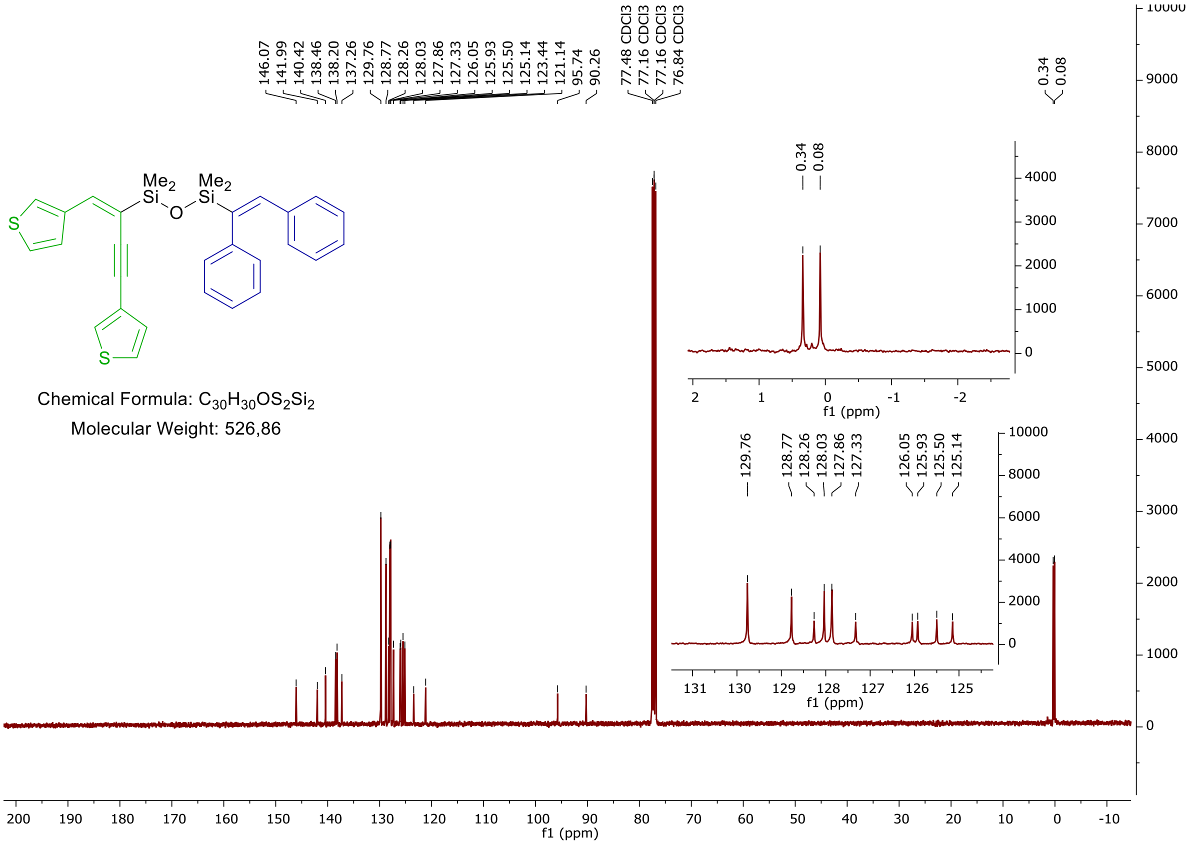

Figure S49. ^{13}C NMR spectrum of **4k.**

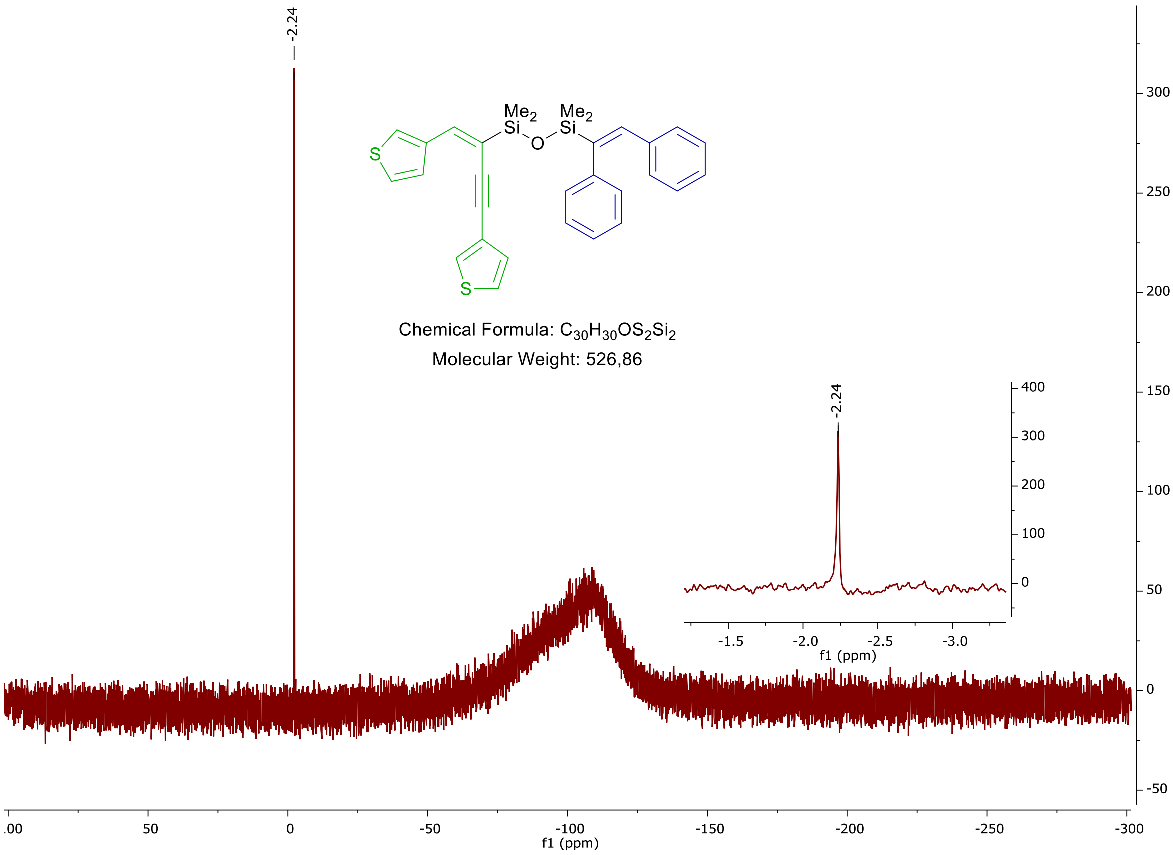

Figure S50. ^{29}Si NMR spectrum of **4k.**

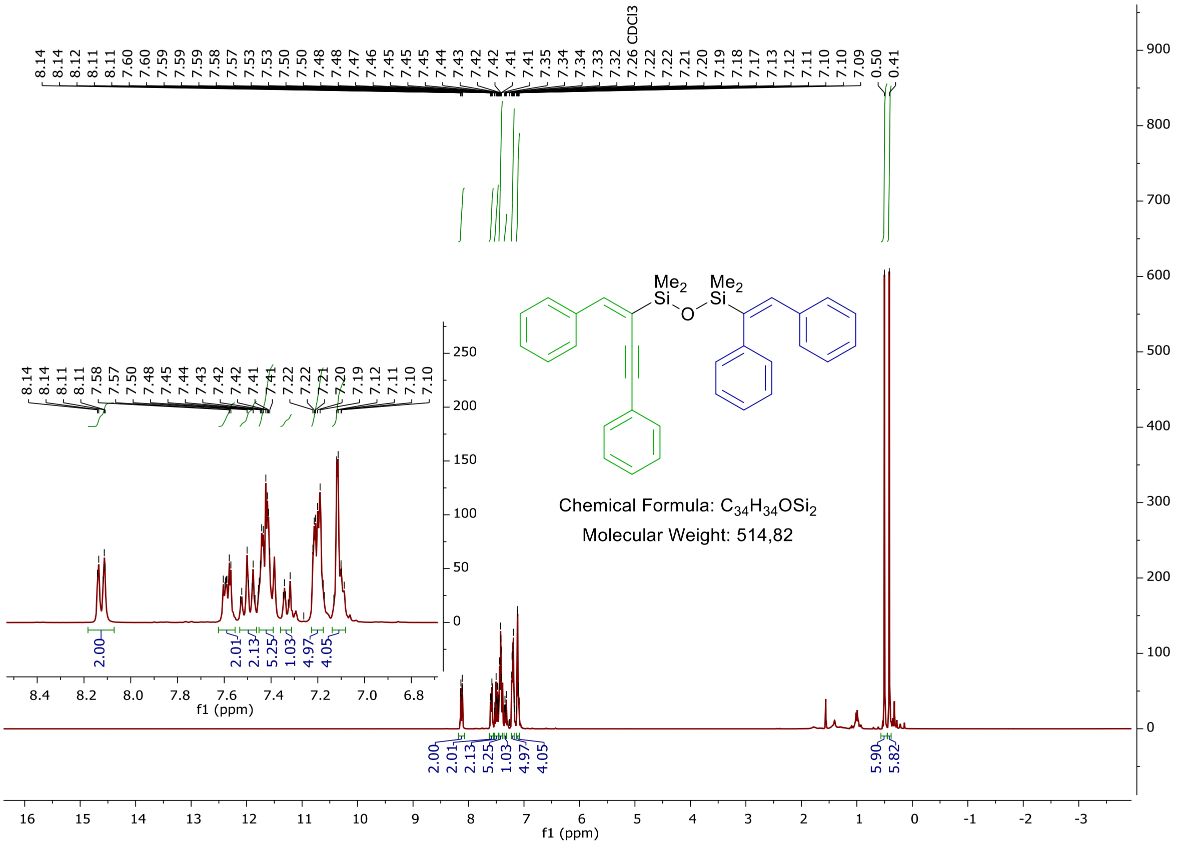

Figure S51. ^{1}H NMR spectrum of **4l**.

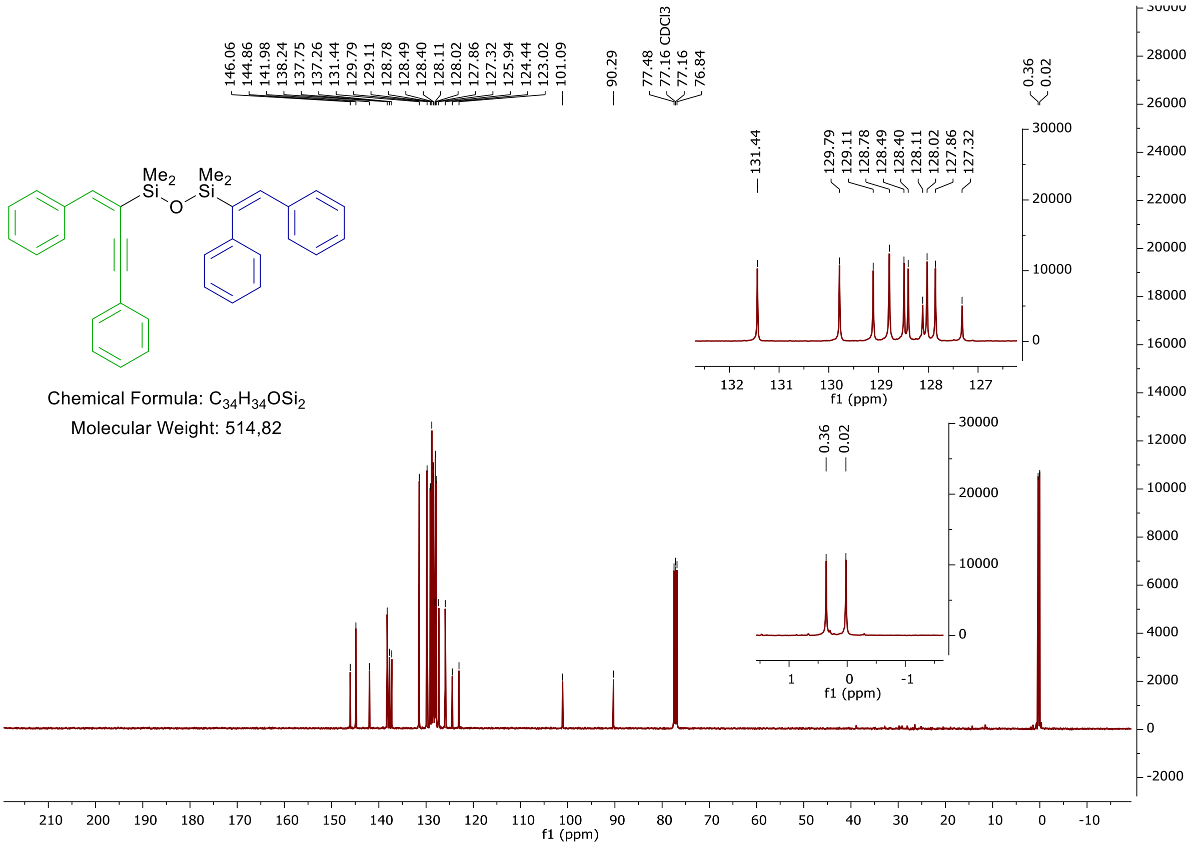

Figure S52. ^{13}C NMR spectrum of **4l**.

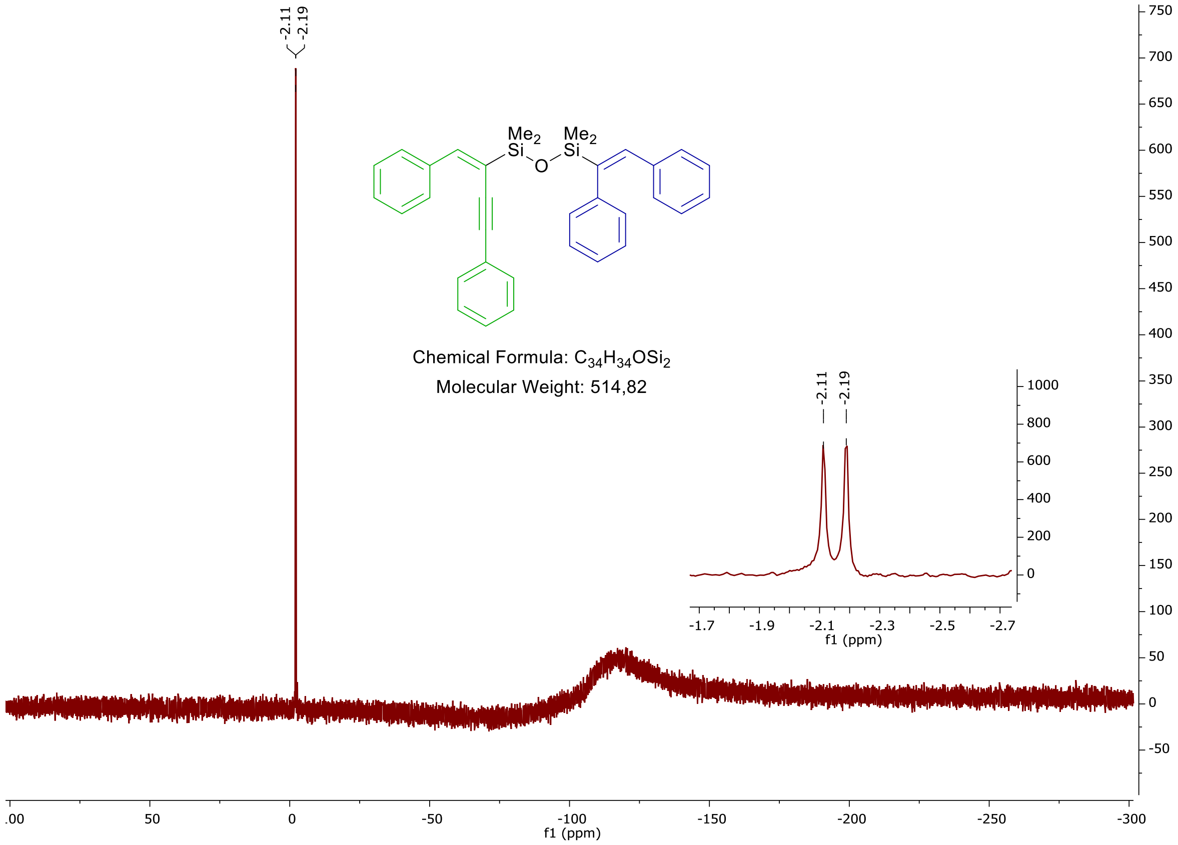

Figure S53. ^{29}Si NMR spectrum of **4l**.

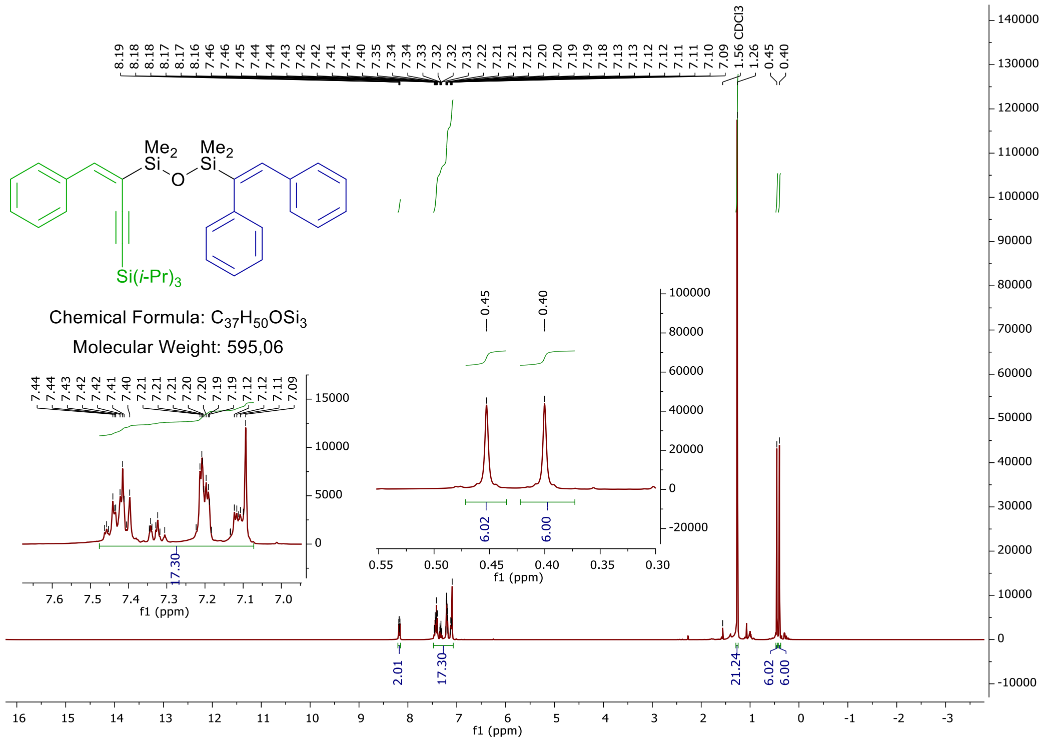

Figure S54. ^{1}H NMR spectrum of **4m**.

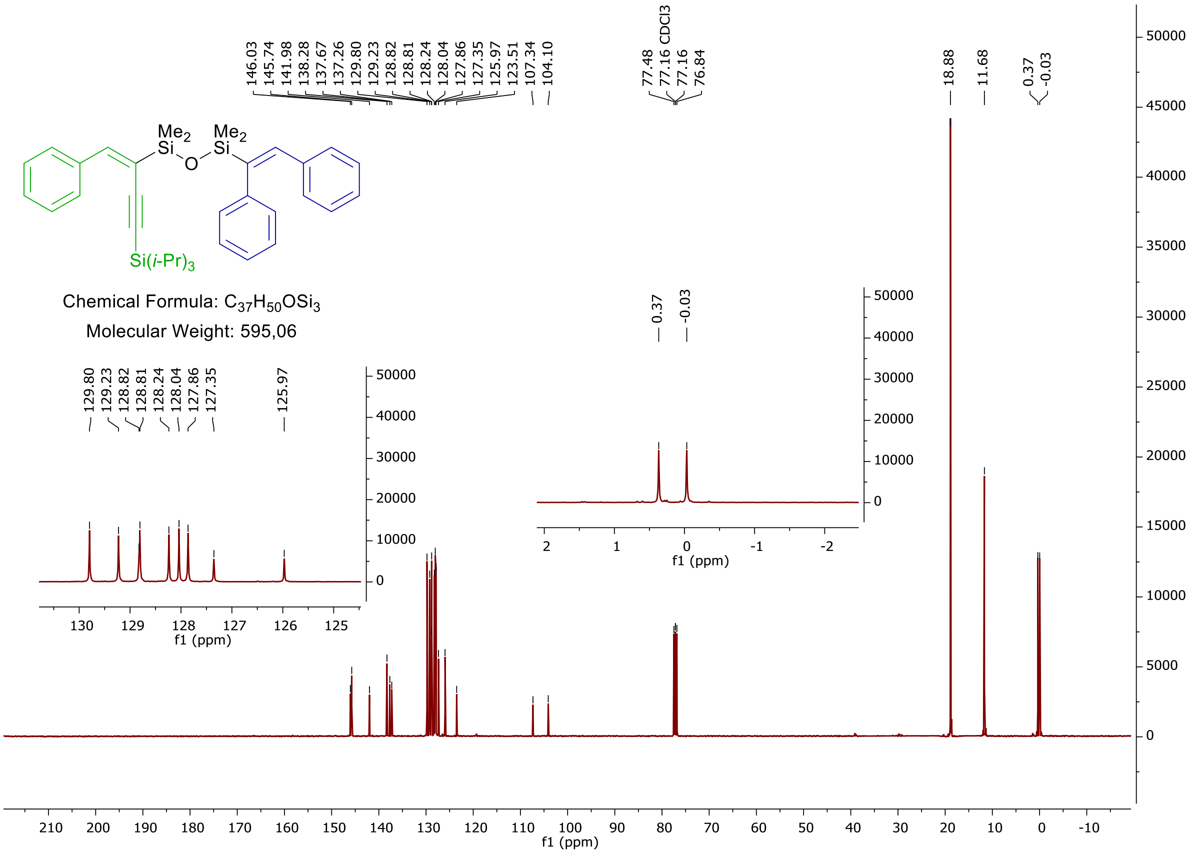

Figure S55. ^{13}C NMR spectrum of **4m.**

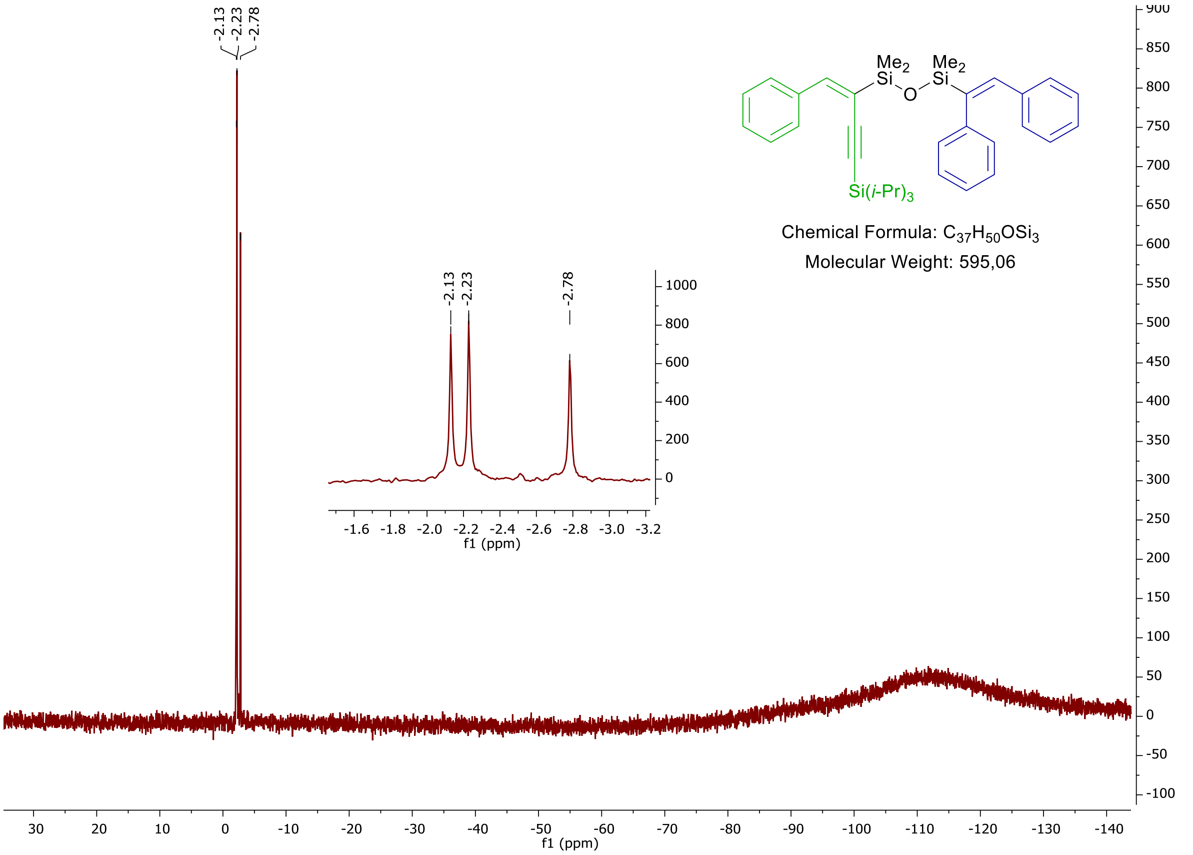

Figure S56. ^{29}Si NMR spectrum of **4m.**

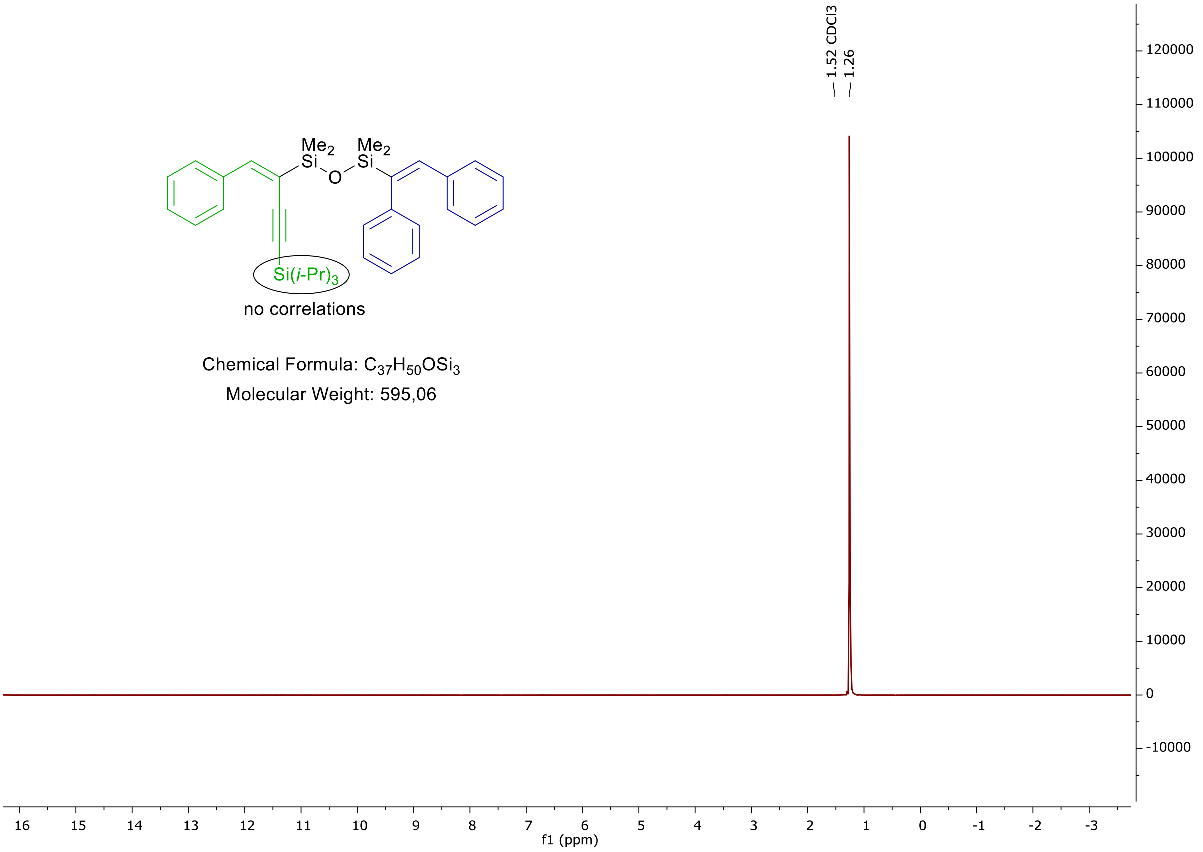

Figure S57. 1D Selective Gradient NOESY of **4m**. Freq.: 1.26 ppm.

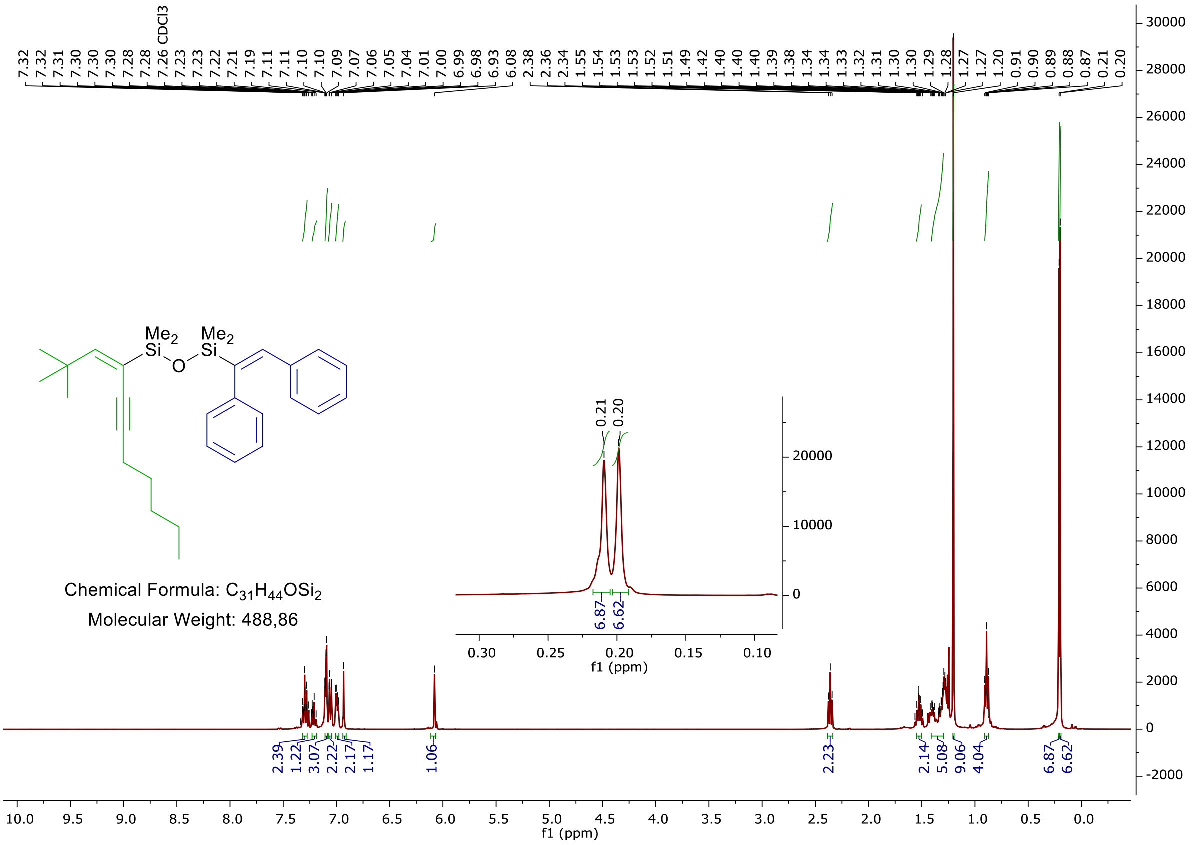

Figure S58. ^{1}H NMR spectrum of **4n.**

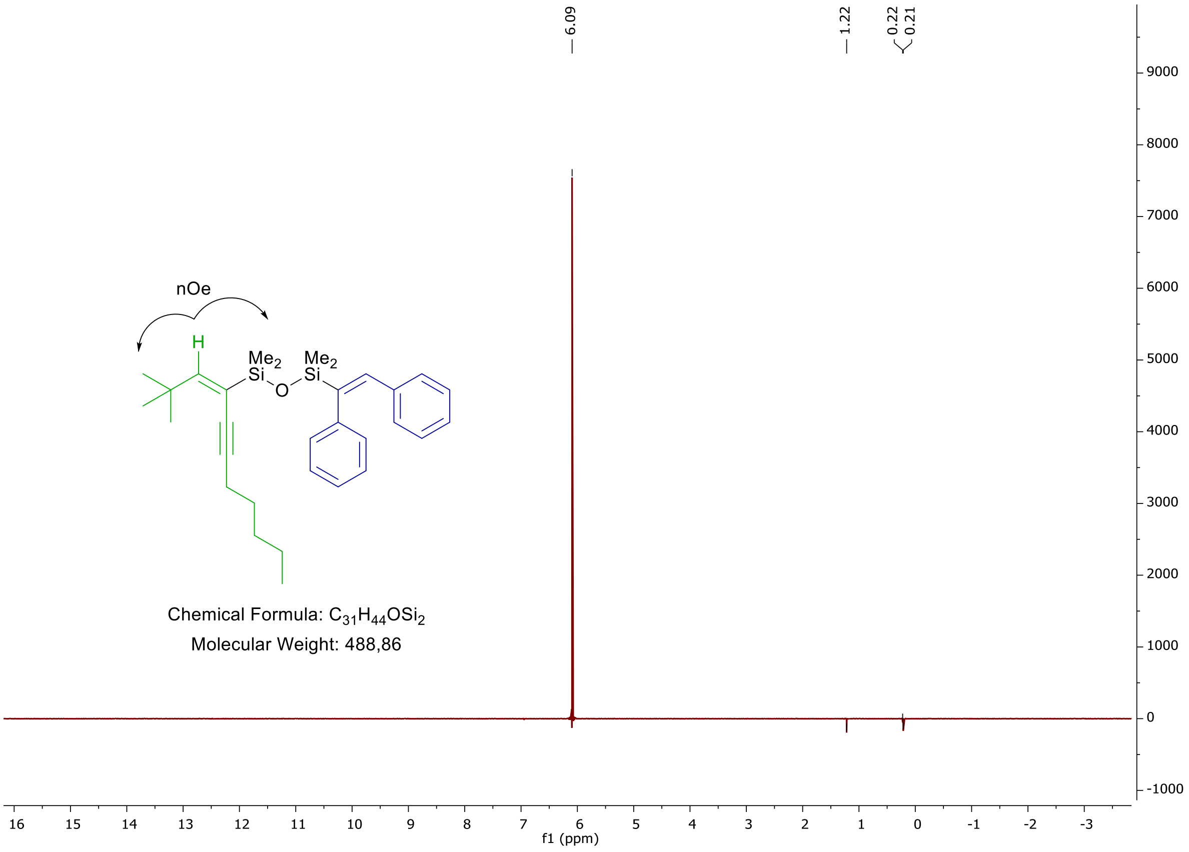

Figure S59. 1D Selective Gradient NOESY of **4n**. Freq.: 6.09 ppm.

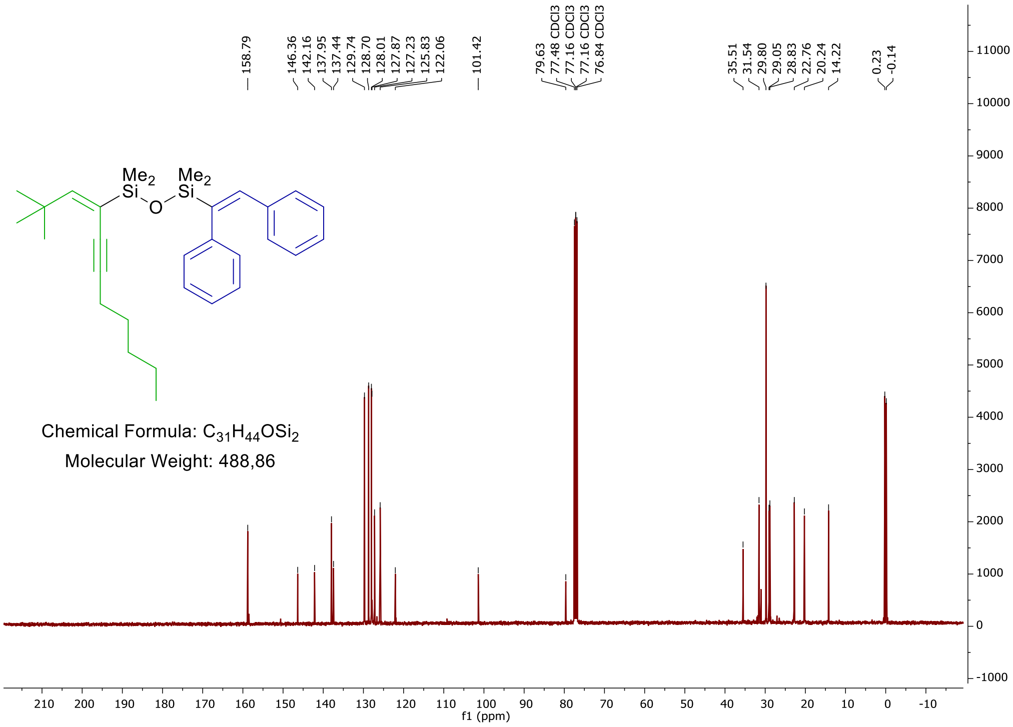

Figure S60. ^{13}C NMR spectrum of **4n**.

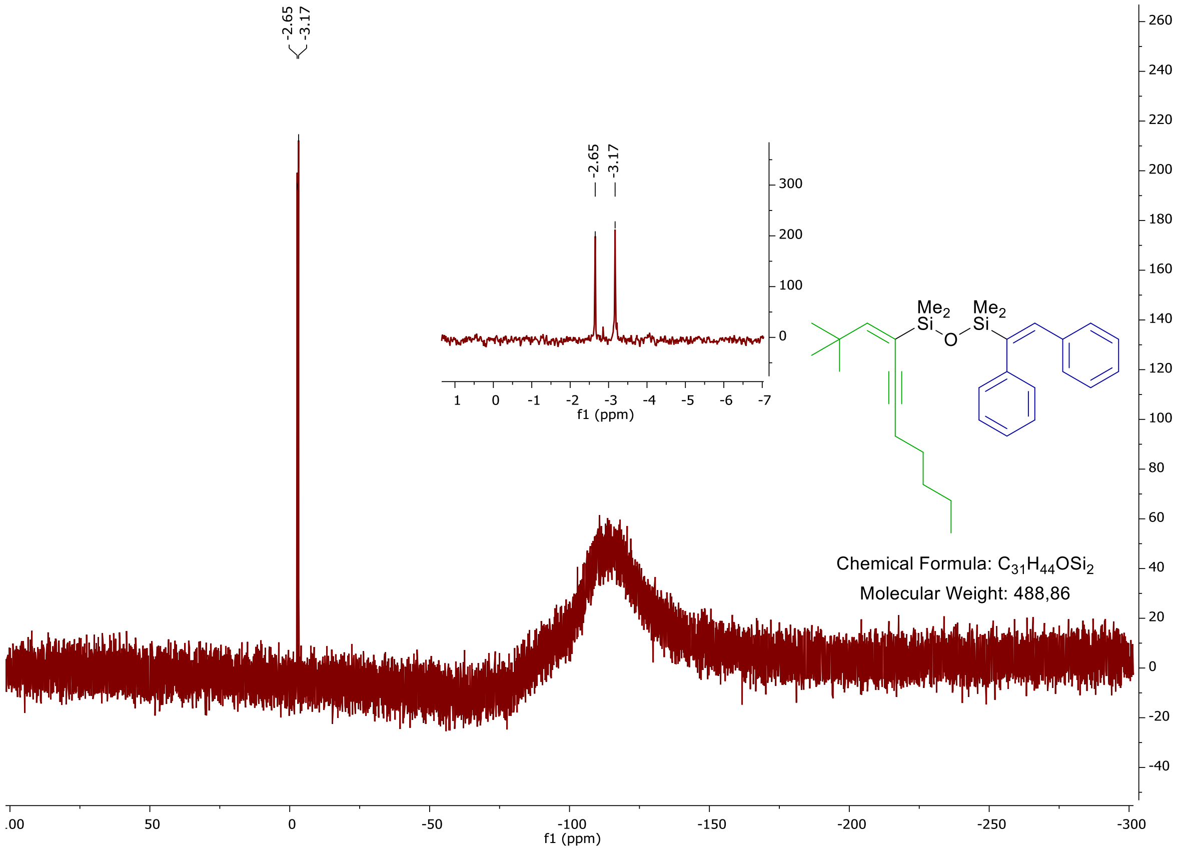

Figure S61. ^{29}Si NMR spectrum of **4n**.

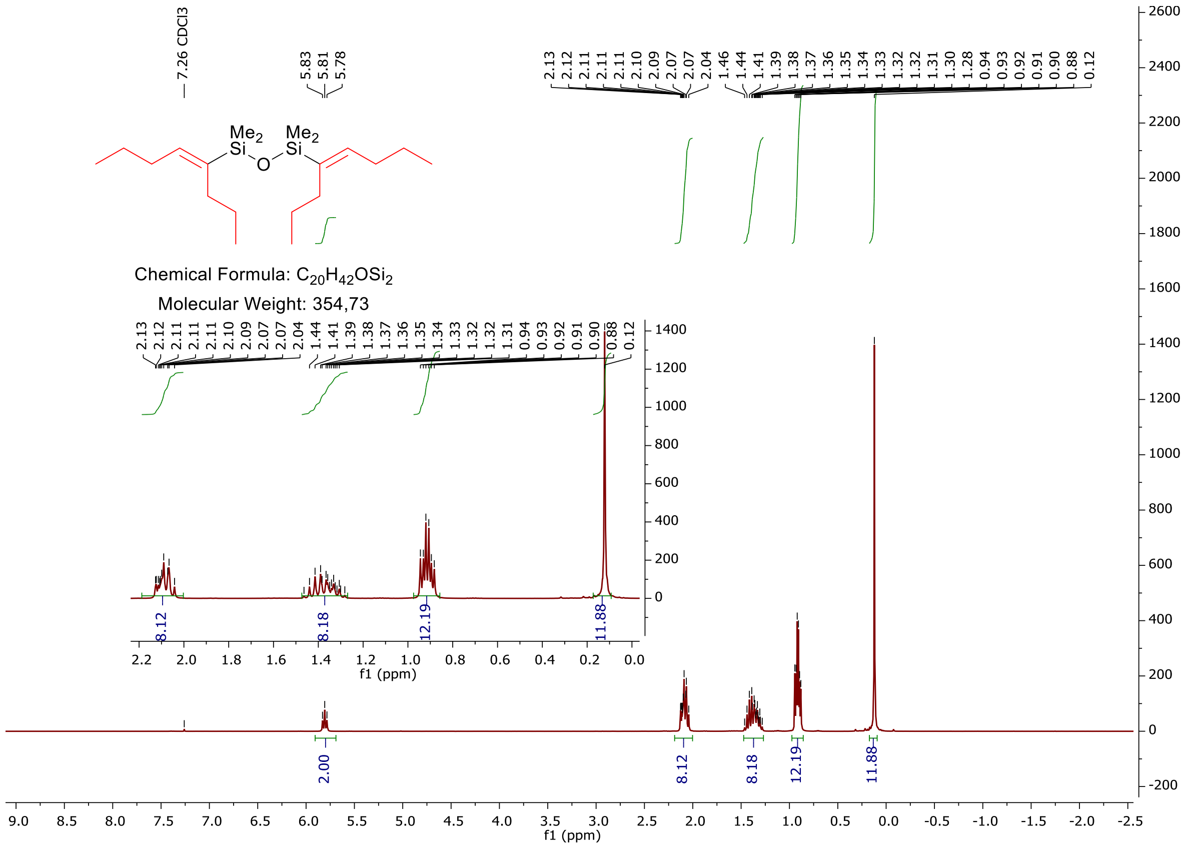

Figure S62. ^{1}H NMR spectrum of **5a**.

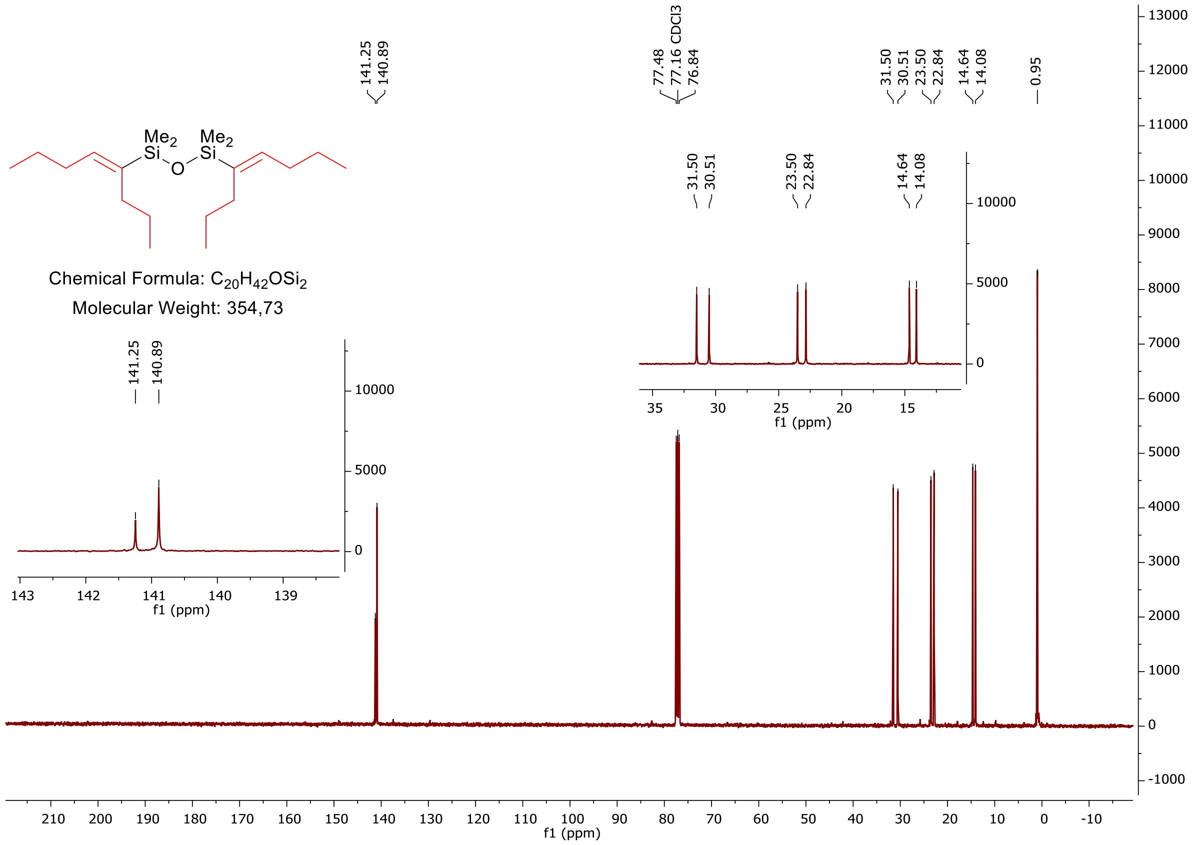

Figure S63. ^{13}C NMR spectrum of **5a**.

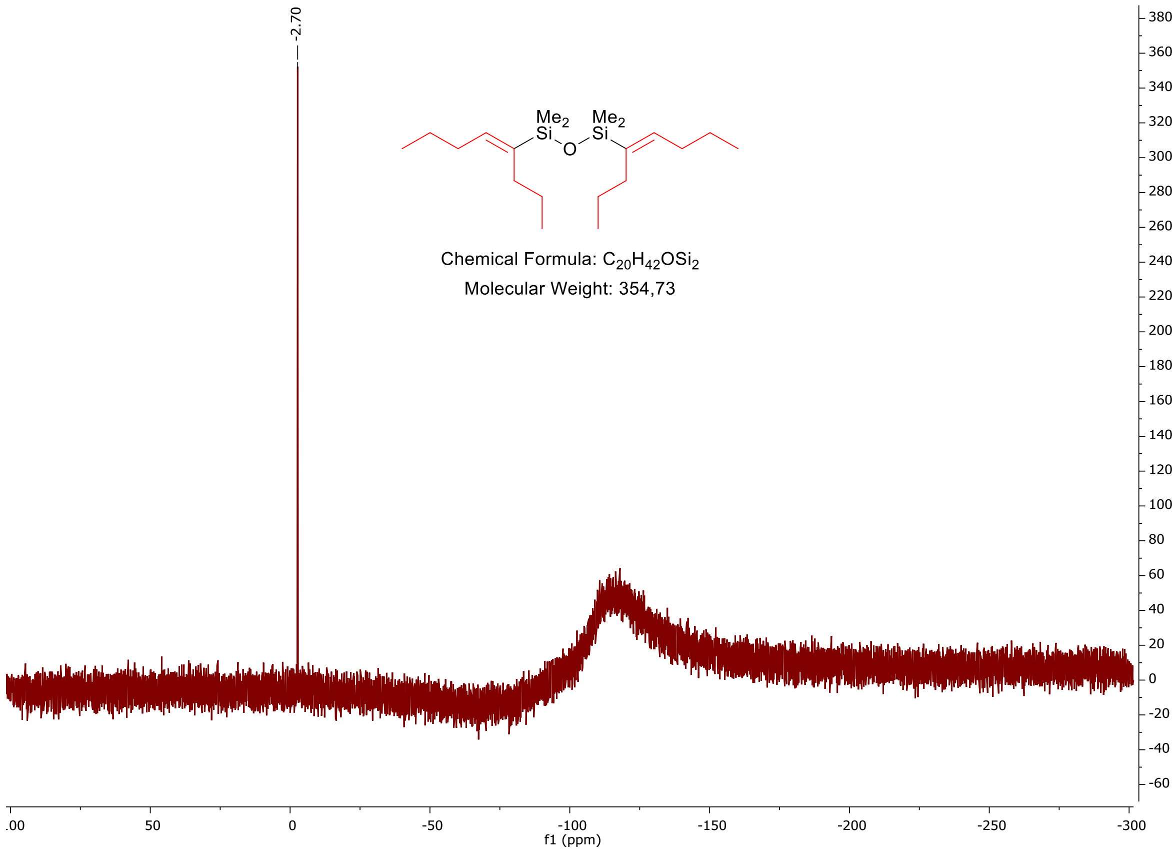

Figure S64. ^{29}Si NMR spectrum of **5a**.

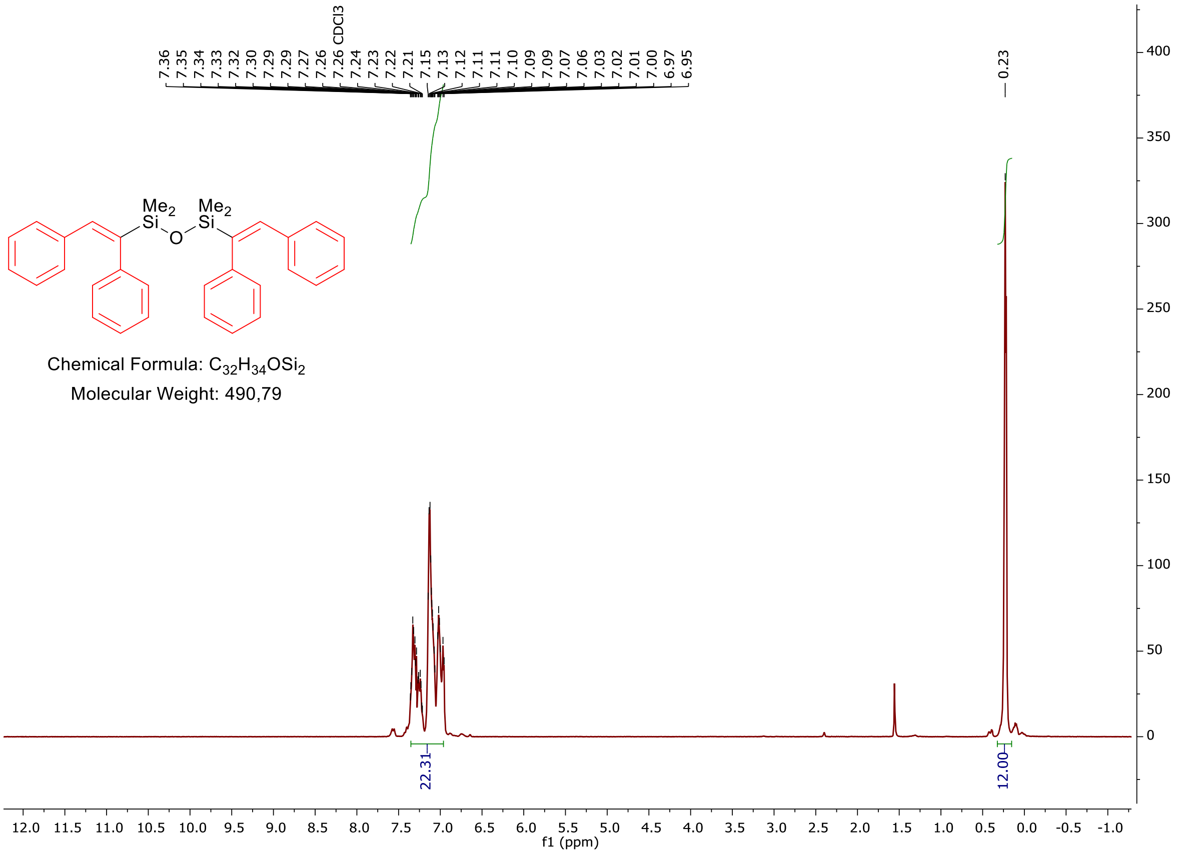

Figure S65. ^{1}H NMR spectrum of **5b**.

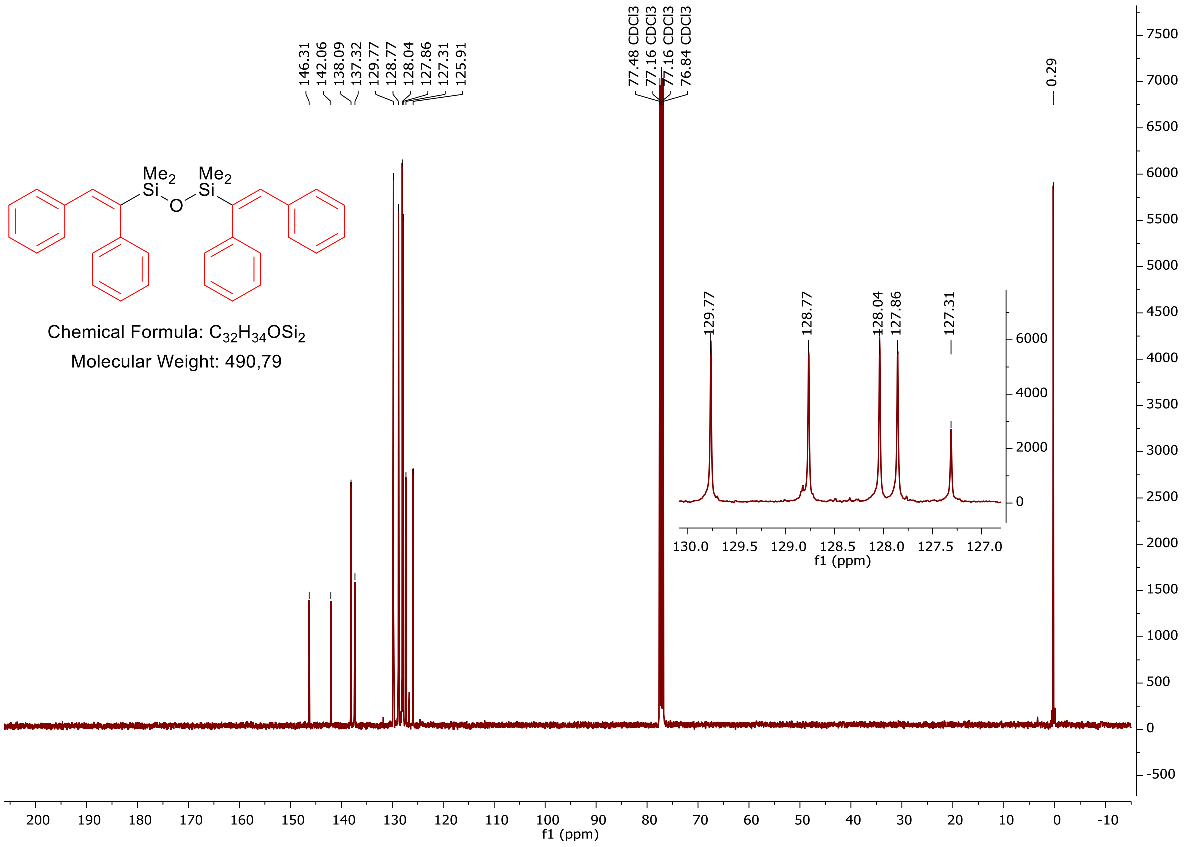

Figure S66. ^{13}C NMR spectrum of **5b**.

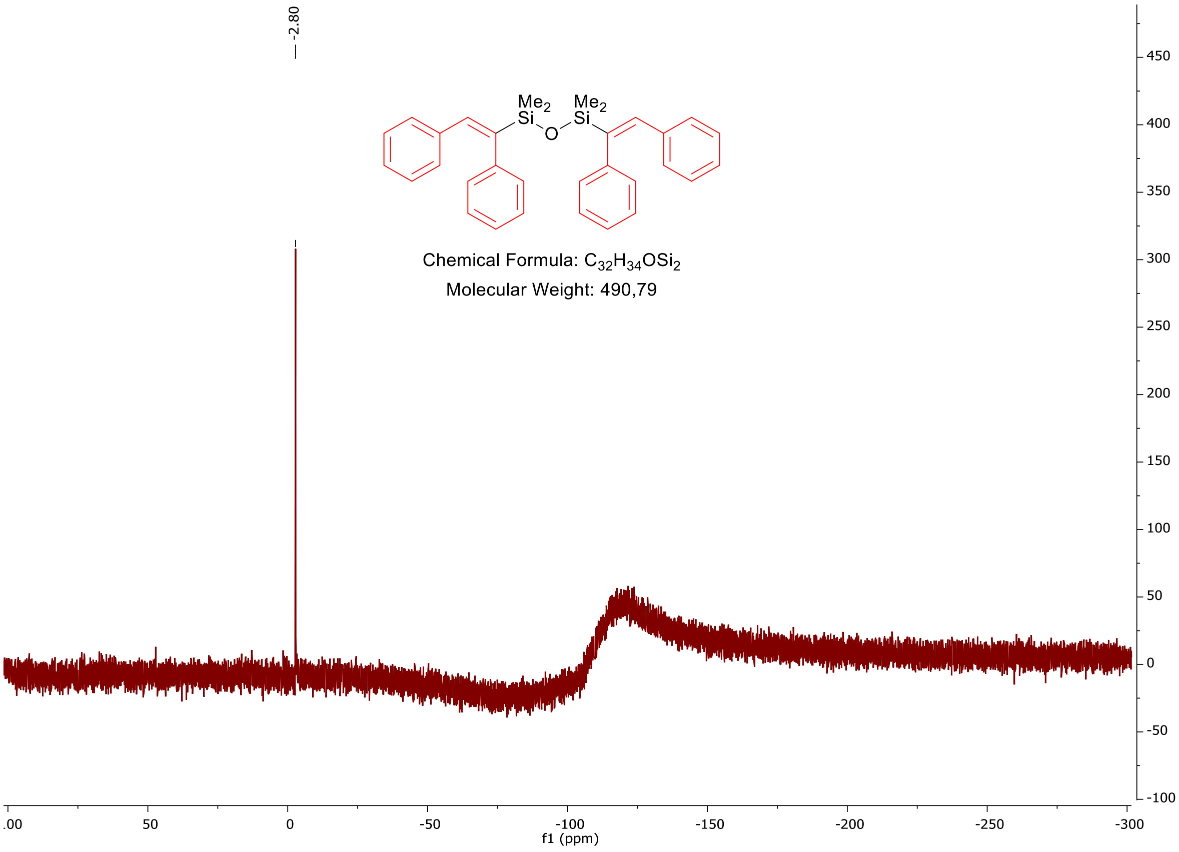

Figure S67. ^{29}Si NMR spectrum of **5b**.

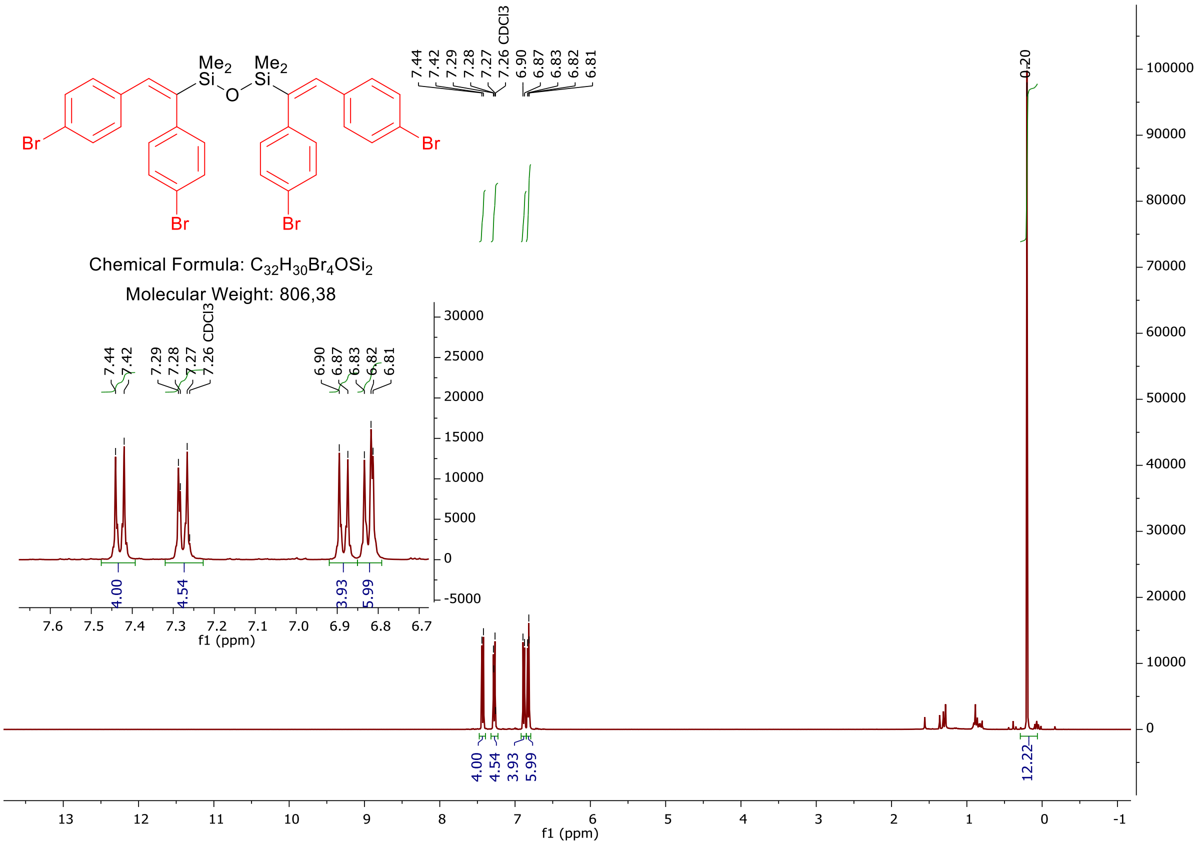

Figure S68. ^{1}H NMR spectrum of **5c**.

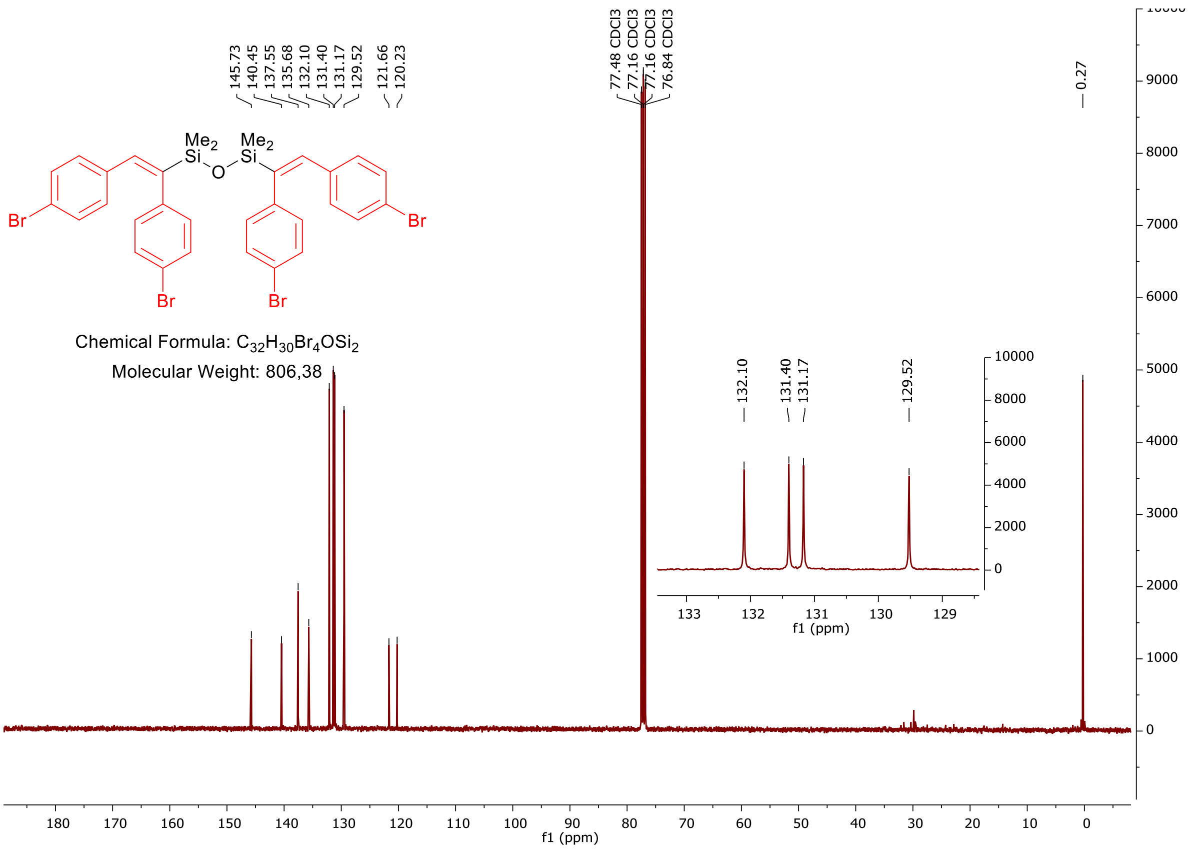

Figure S69. ^{13}C NMR spectrum of **5c.**

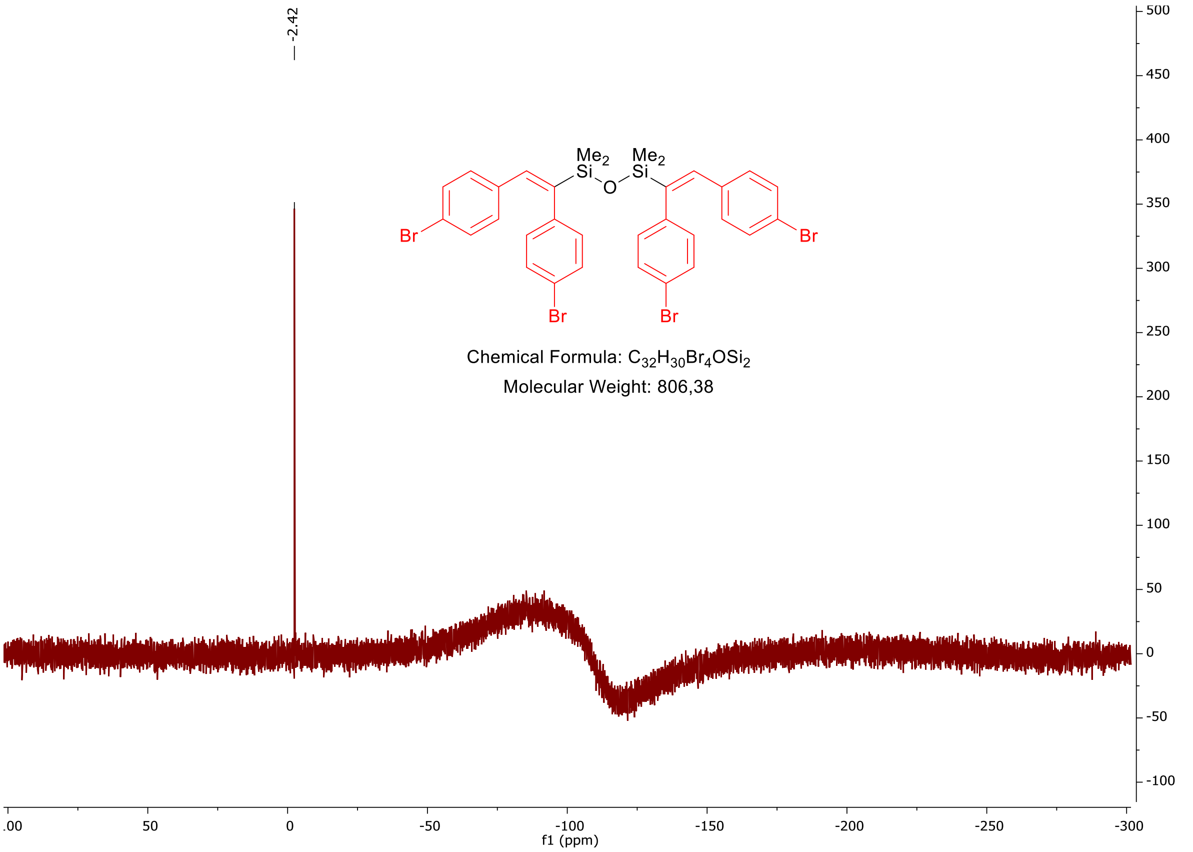

Figure S70. ^{29}Si NMR spectrum of **5c.**

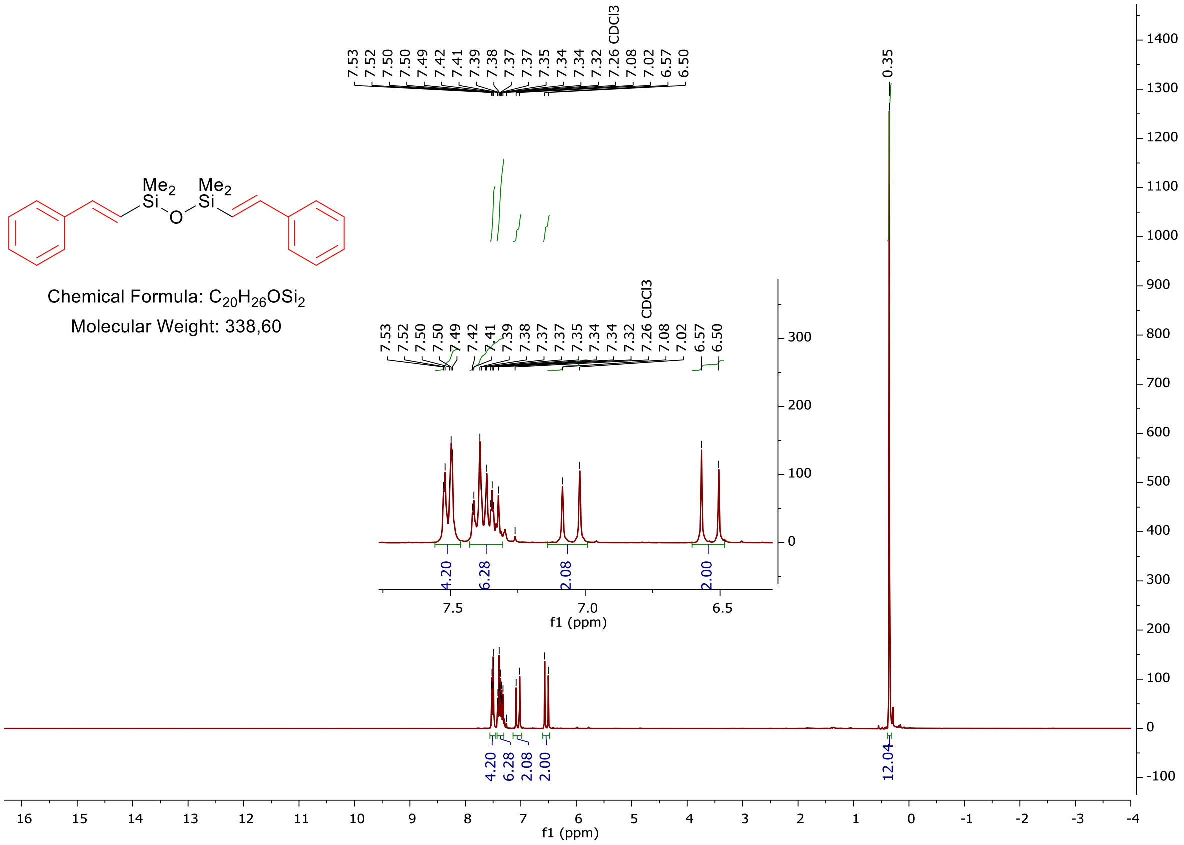

Figure S71. ^{1}H NMR spectrum of **5d**.

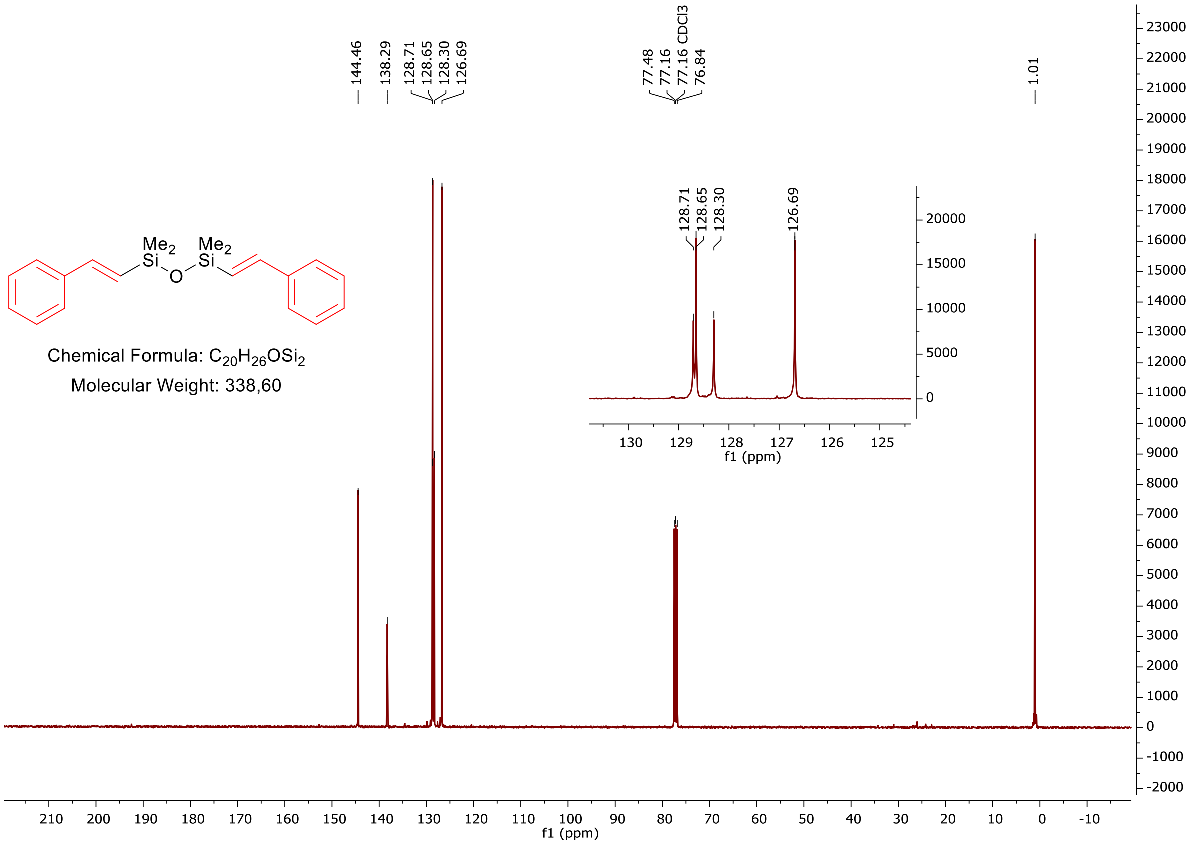

Figure S72. ^{13}C NMR spectrum of **5d**.

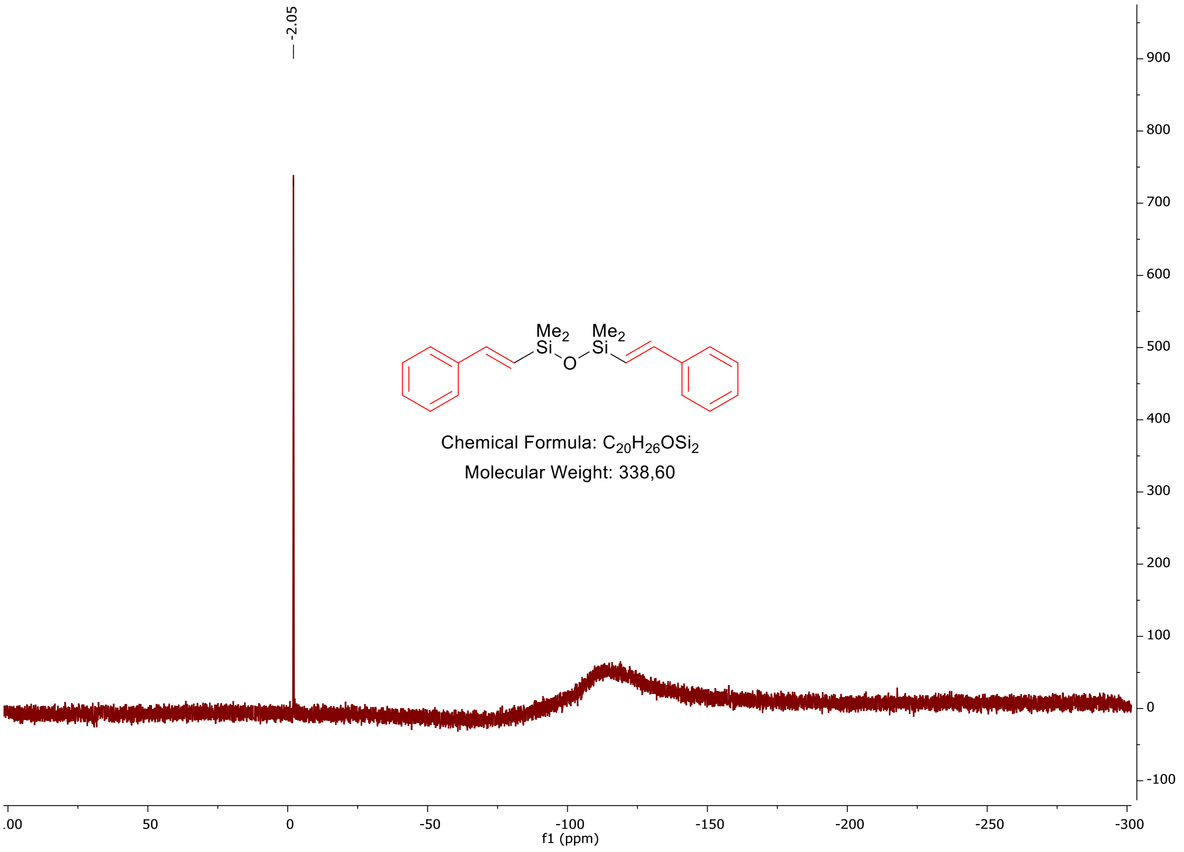

Figure S73. ^{29}Si NMR spectrum of **5d**.

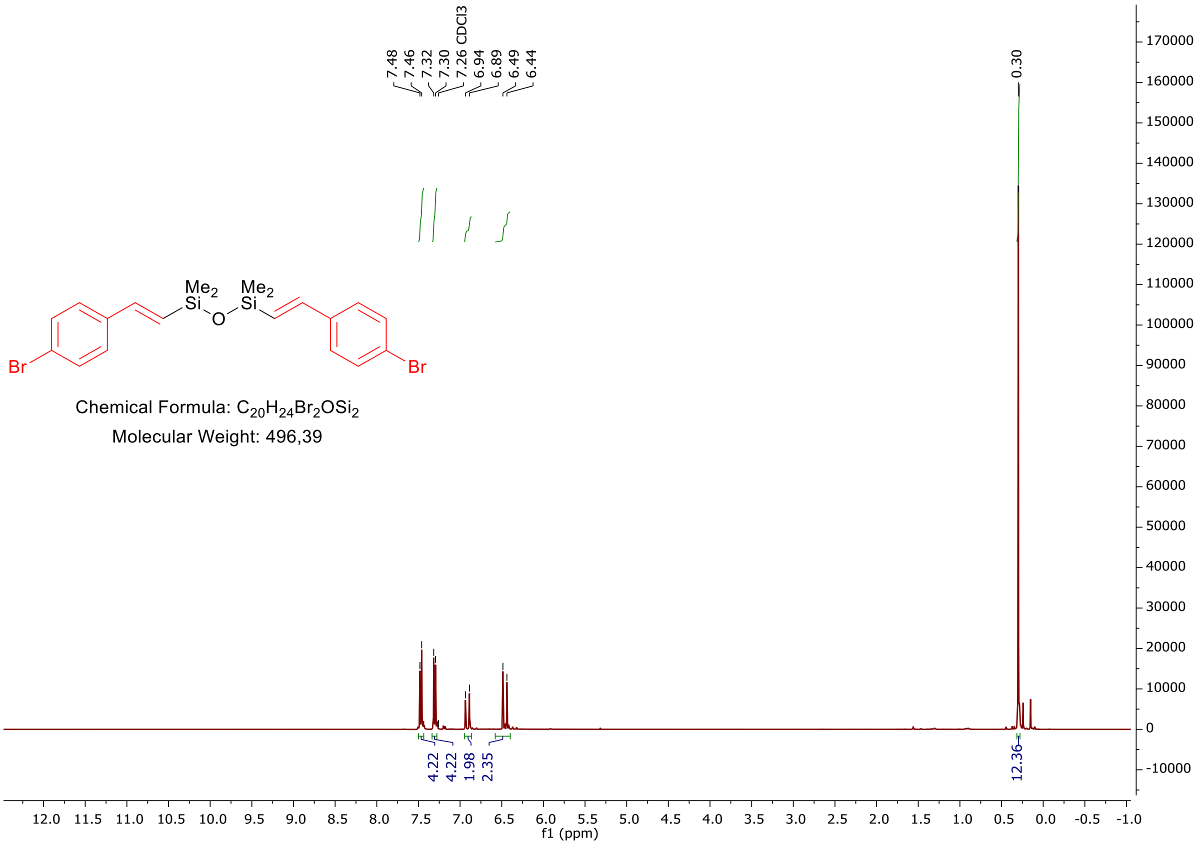

Figure S74. ^{1}H NMR spectrum of **5e**.

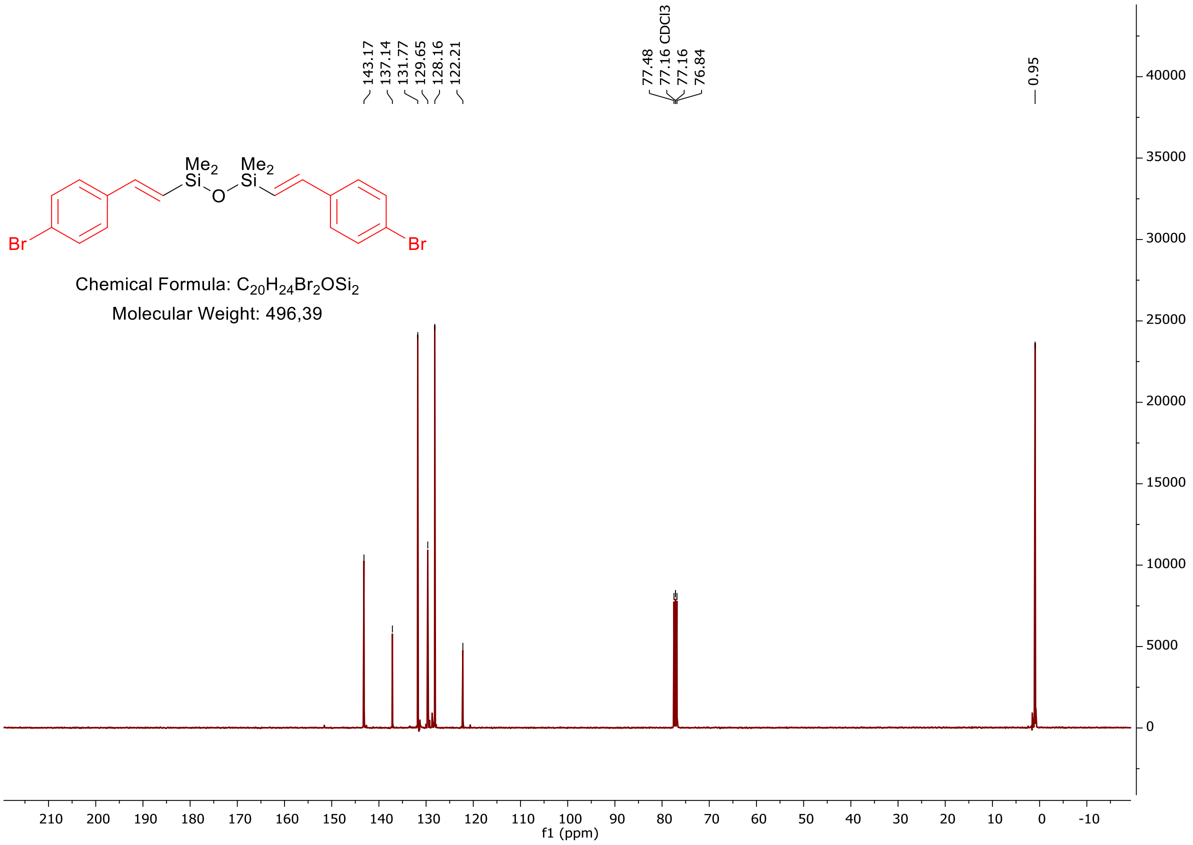

Figure S75. ^{13}C NMR spectrum of **5e**.

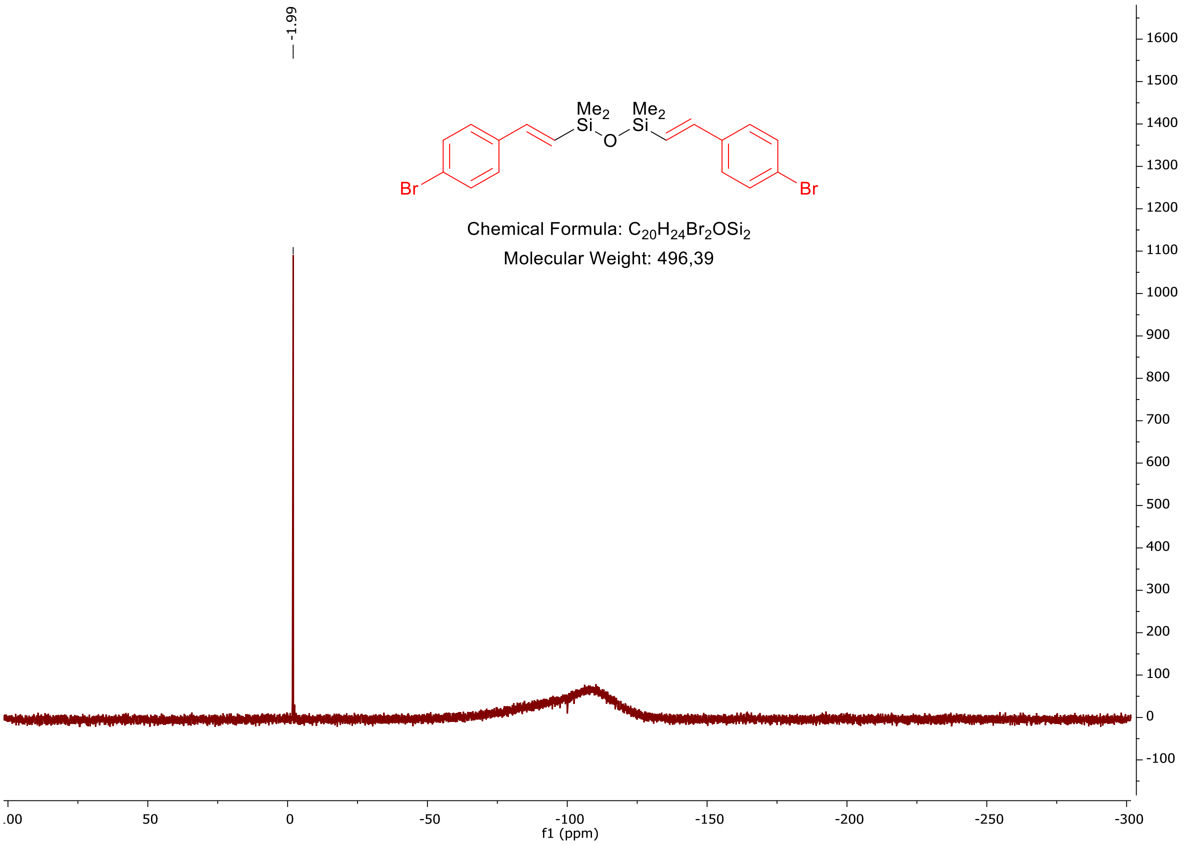

Figure S76. ^{29}Si NMR spectrum of **5e**.

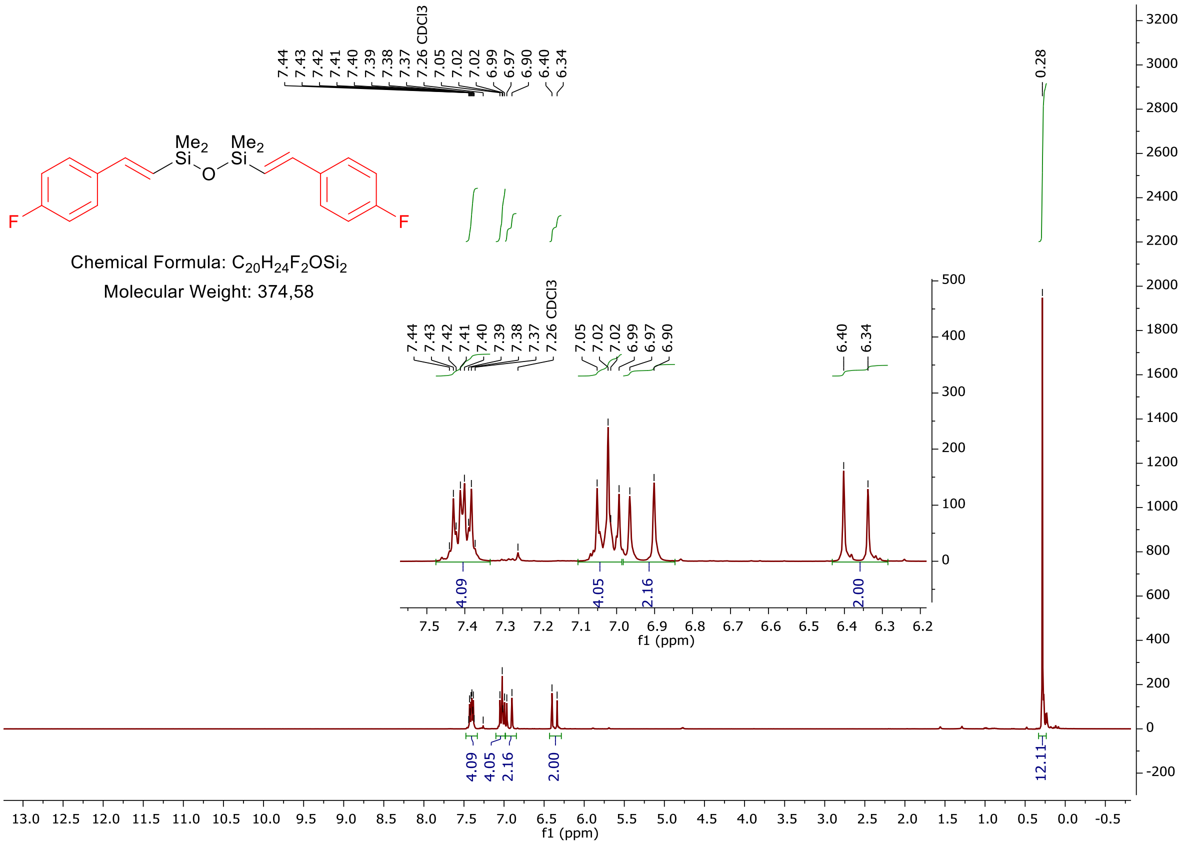

Figure S77. ^{1}H NMR spectrum of **5f**.

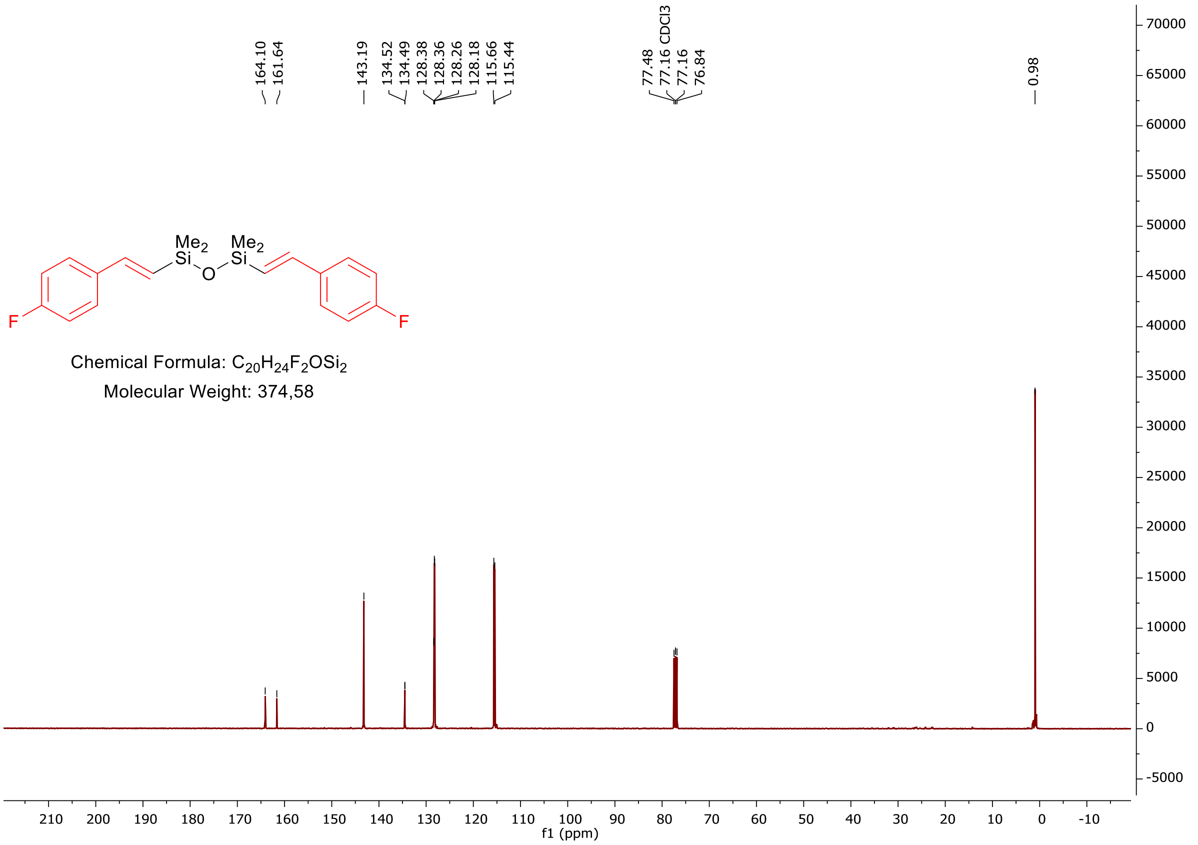

Figure S78. ^{13}C NMR spectrum of **5f**.

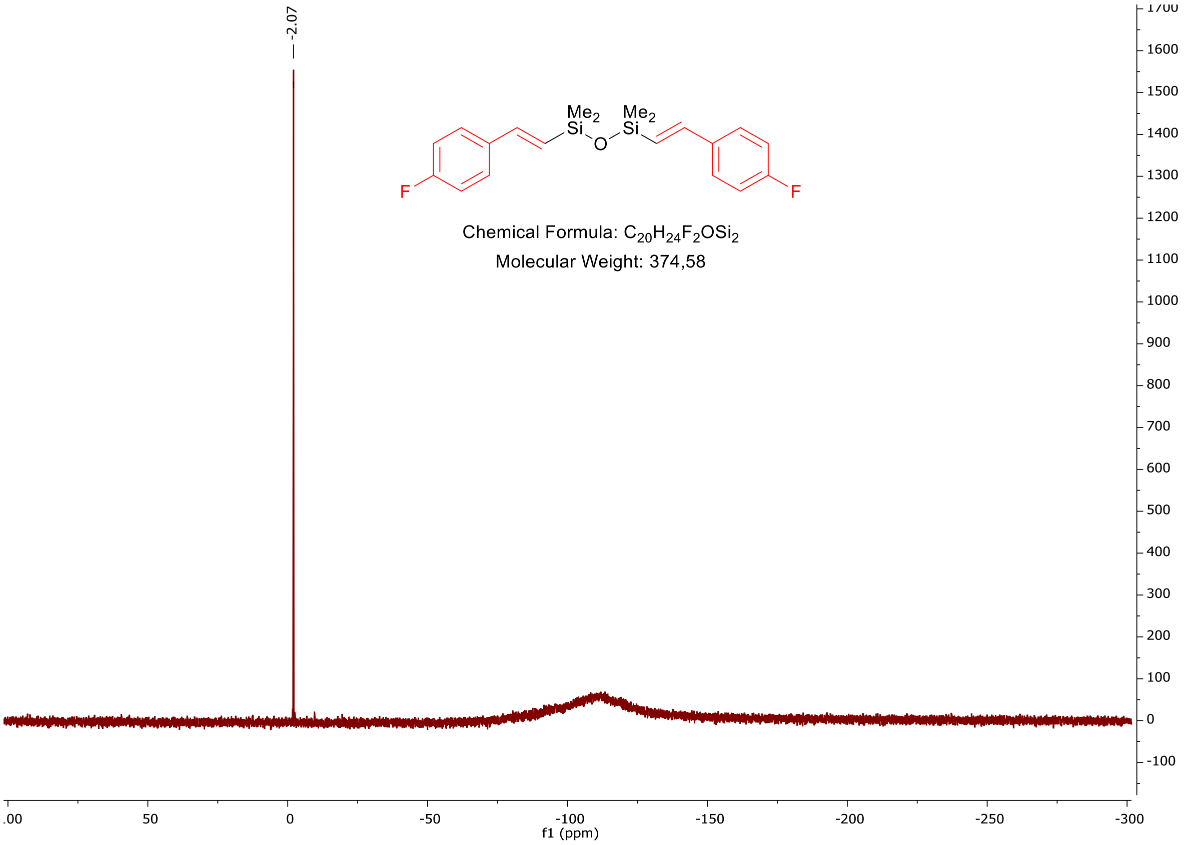

Figure S79. ^{29}Si NMR spectrum of **5f.**

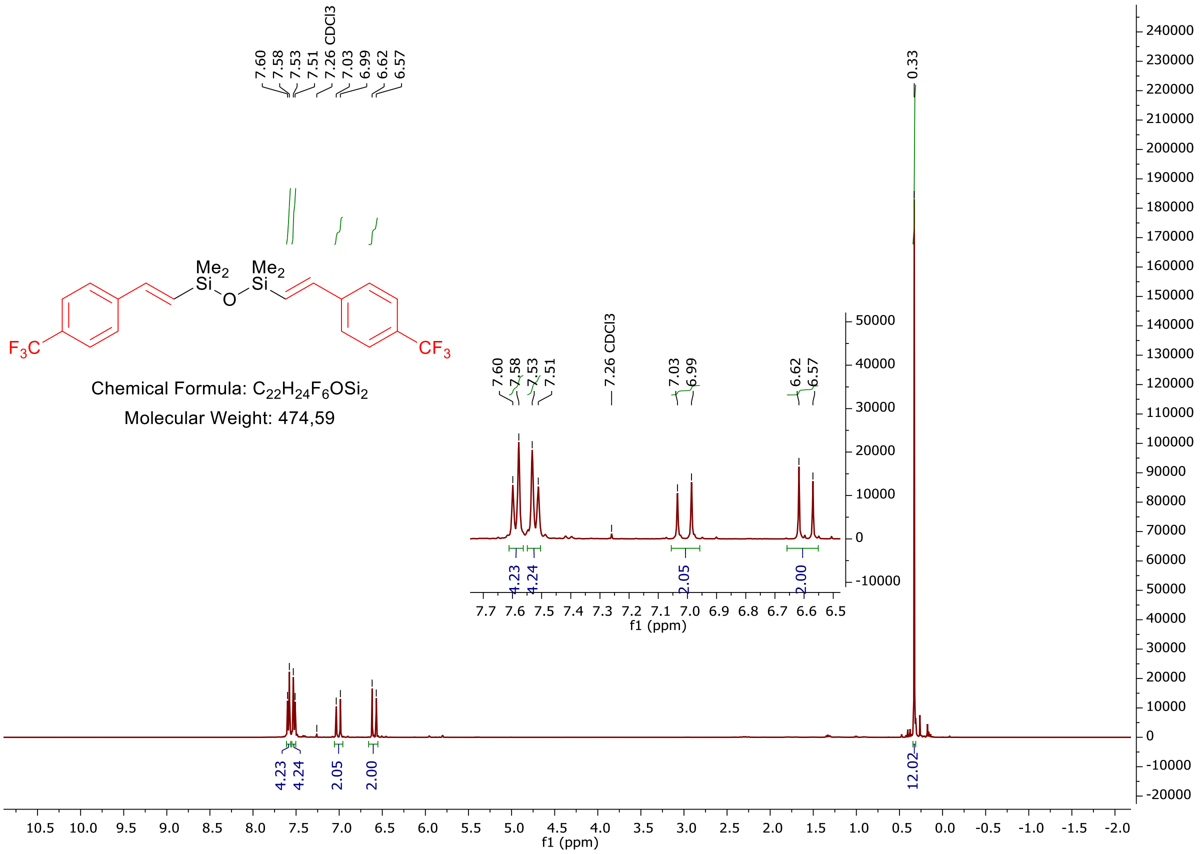

Figure S80. ^{1}H NMR spectrum of **5g.**

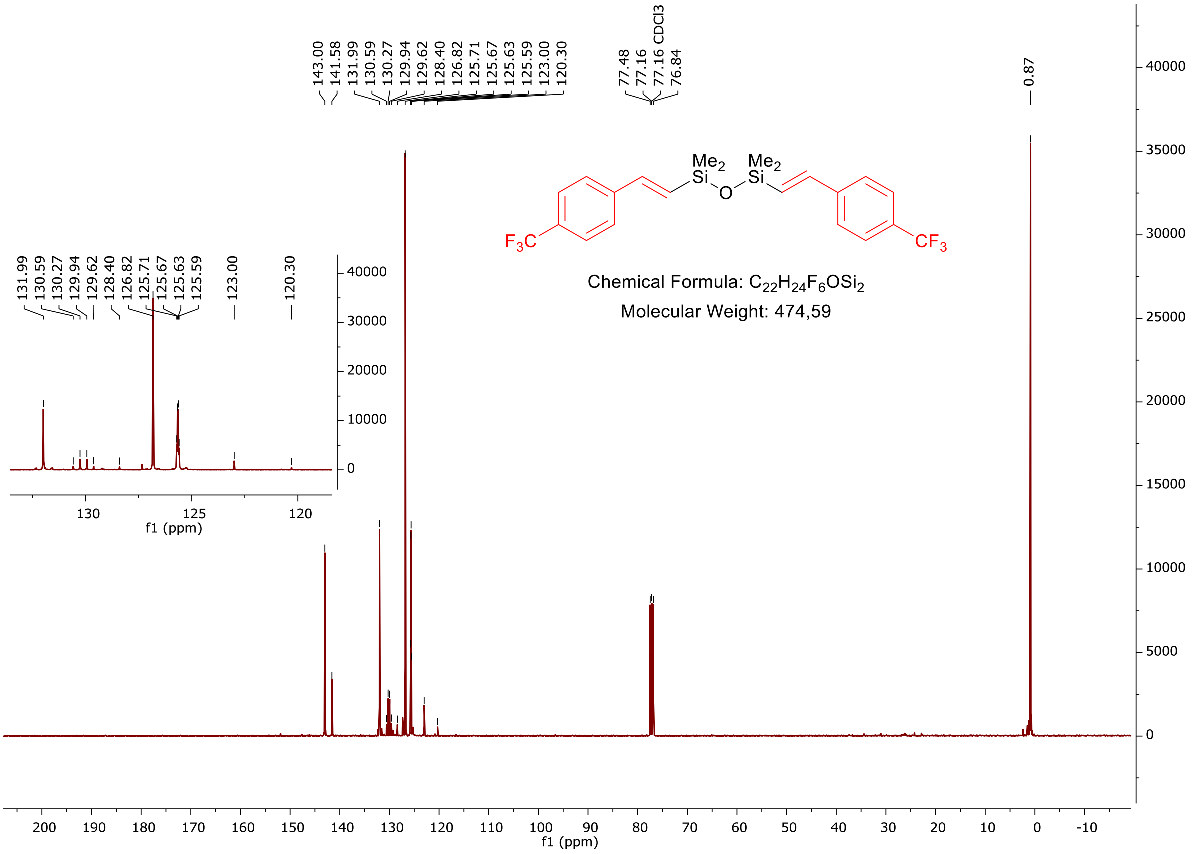

Figure S81. ^{13}C NMR spectrum of **5g.**

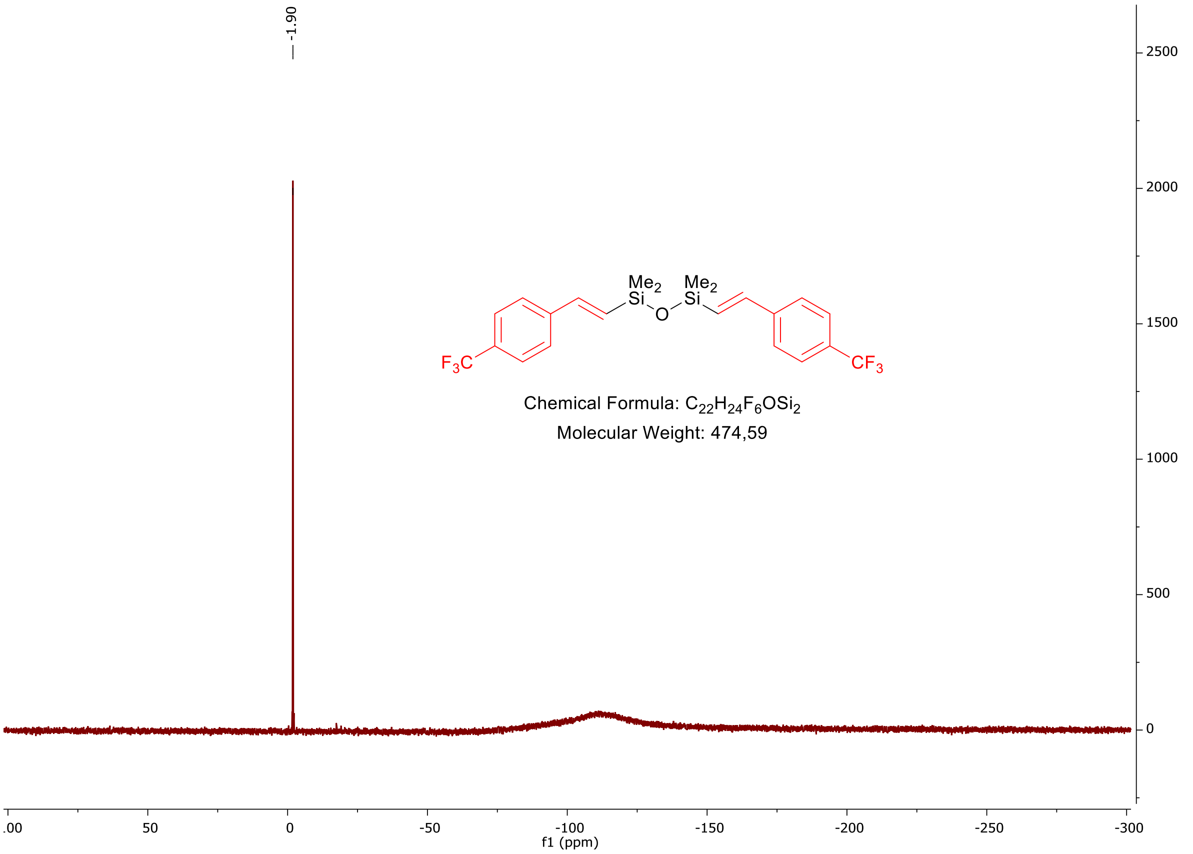

Figure S82. ^{29}Si NMR spectrum of **5g.**

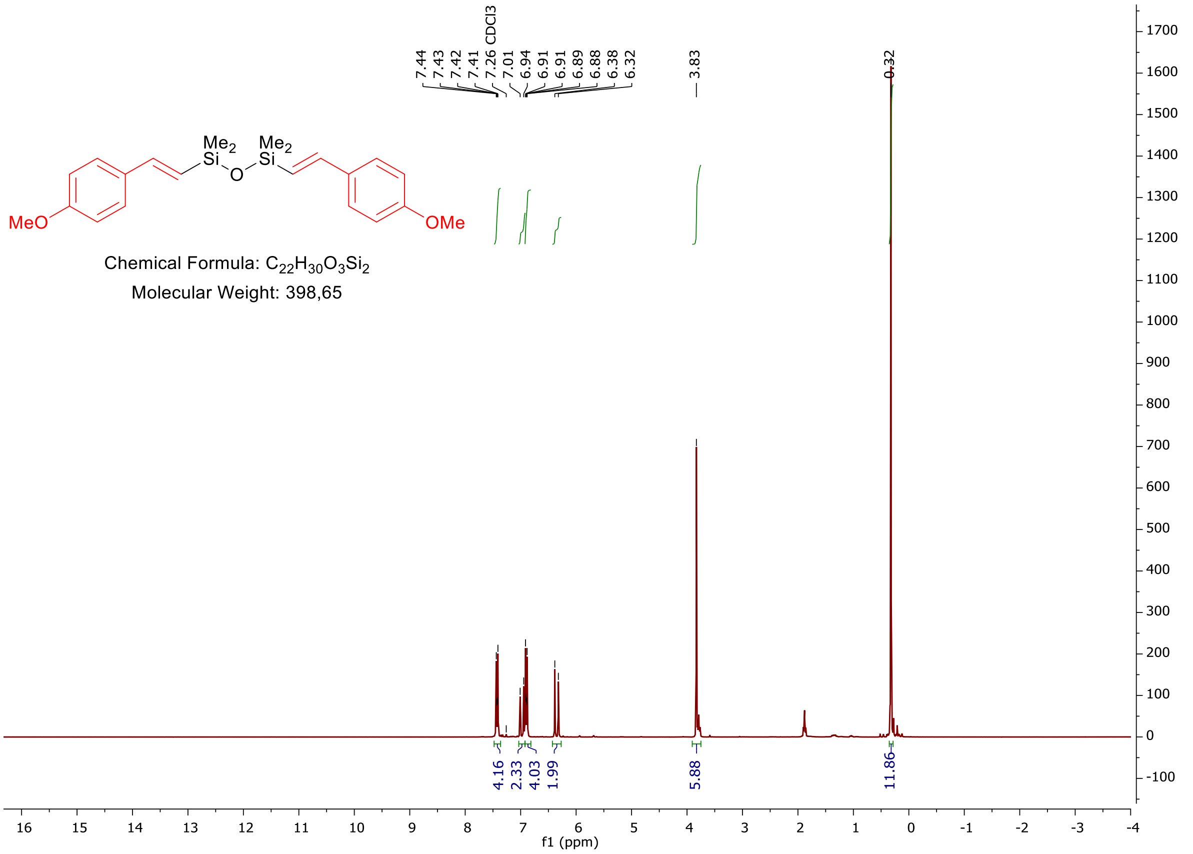

Figure S83. ^{1}H NMR spectrum of **5h.**

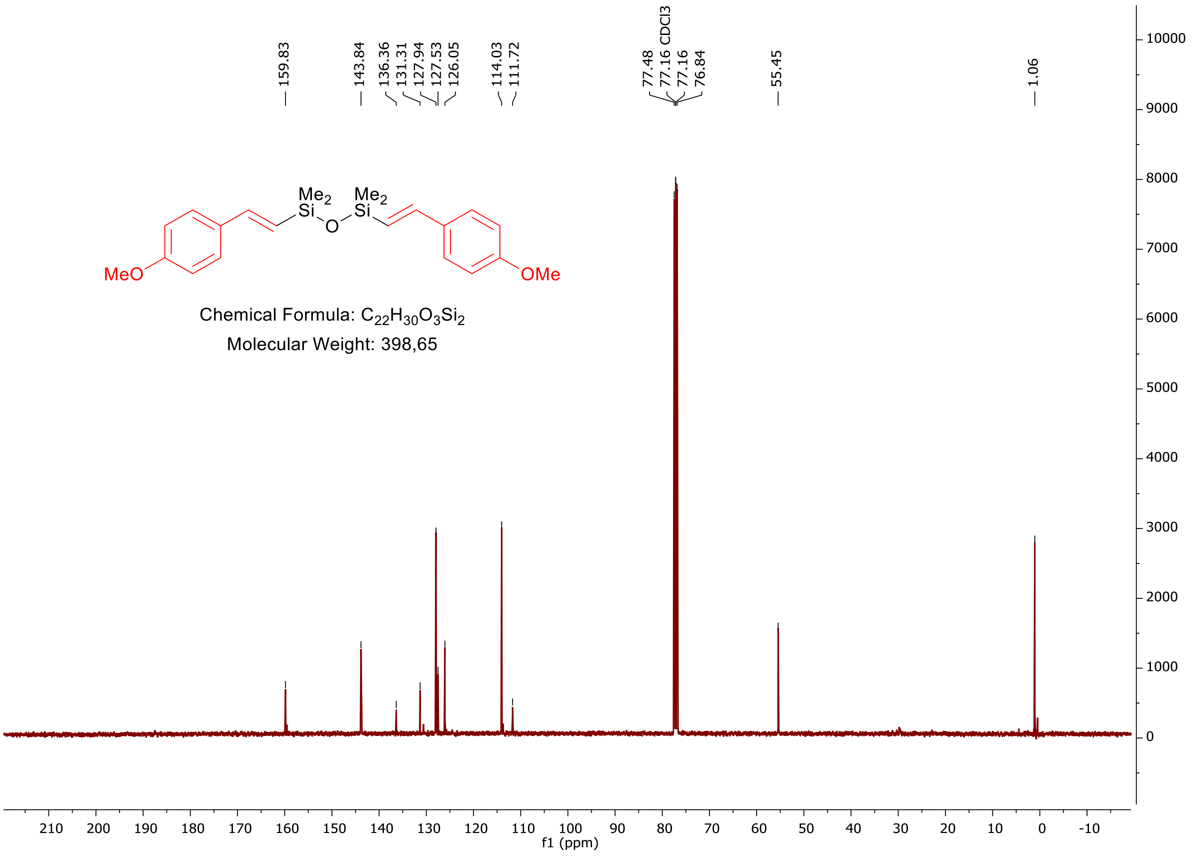

Figure S84. ^{13}C NMR spectrum of **5h.**

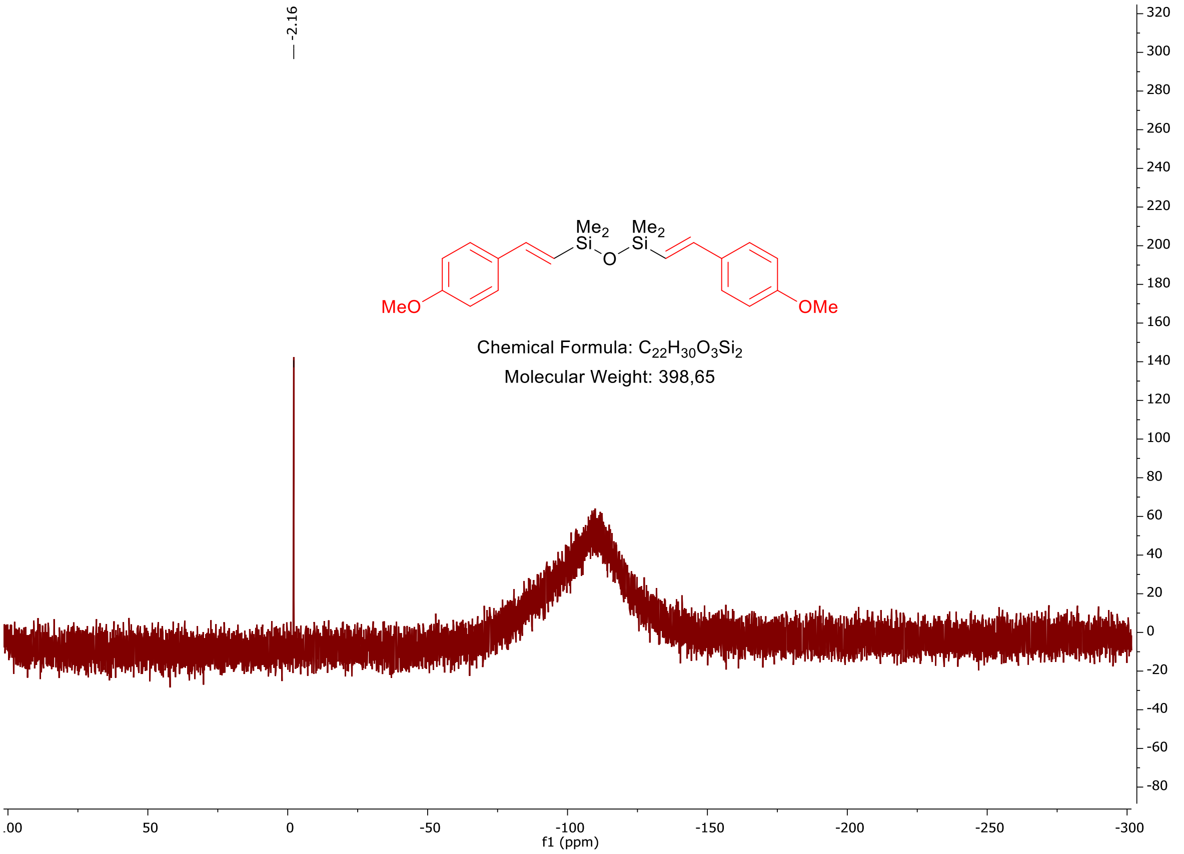

Figure S85. ^{29}Si NMR spectrum of **5h.**

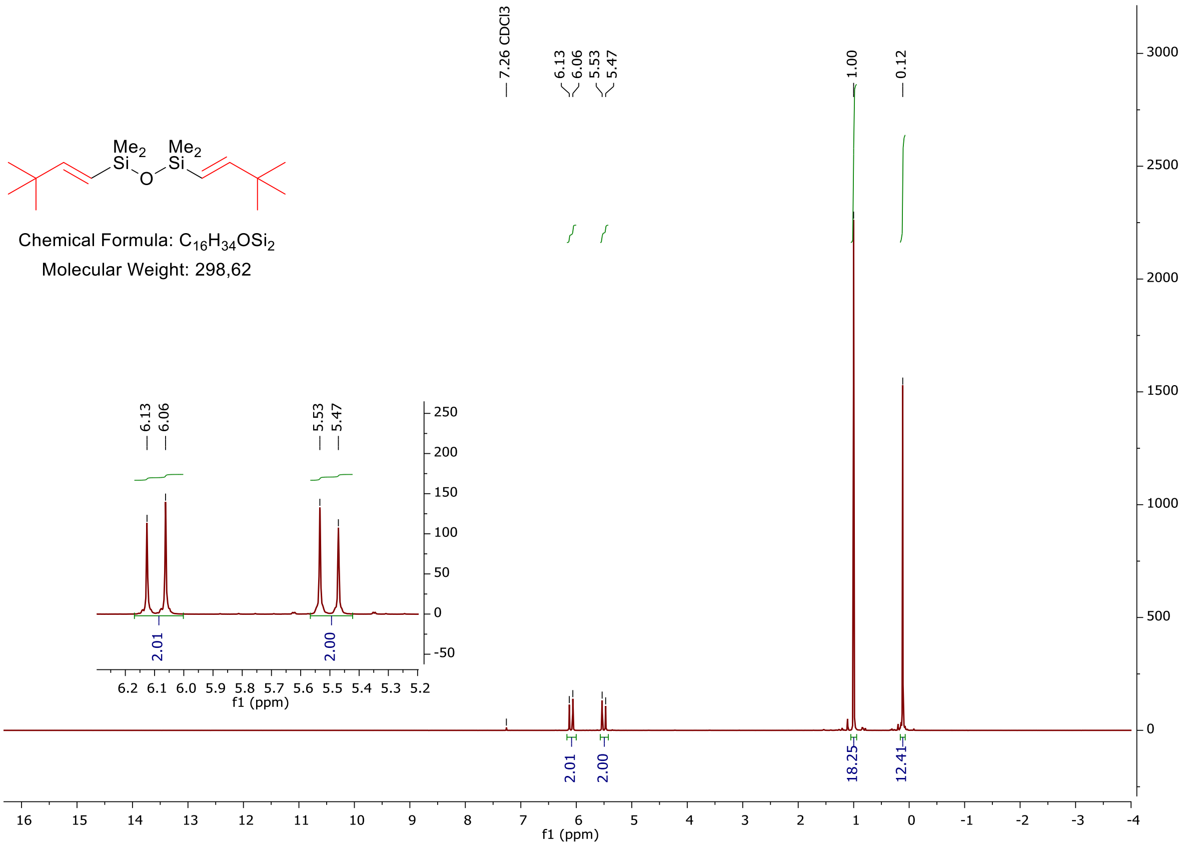

Figure S86. ^{1}H NMR spectrum of **5i.**

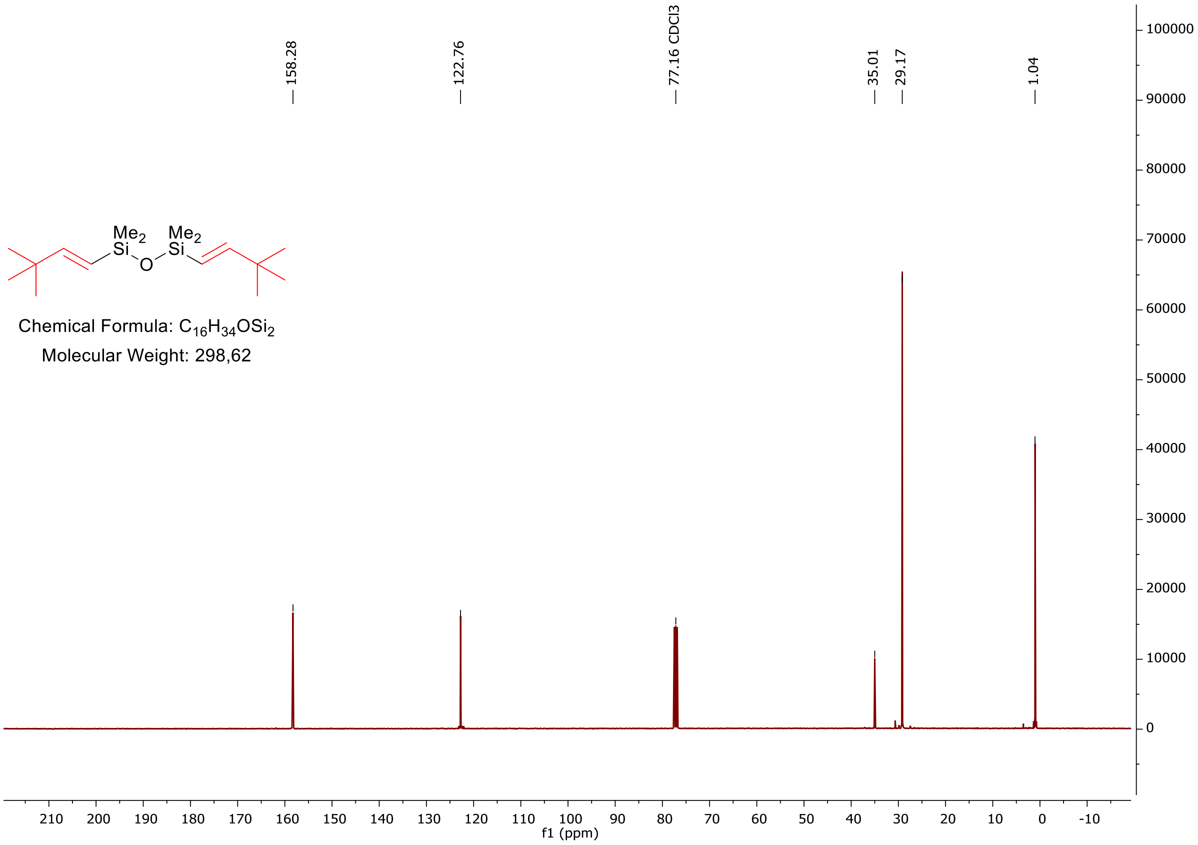

Figure S87. ^{13}C NMR spectrum of **5i.**

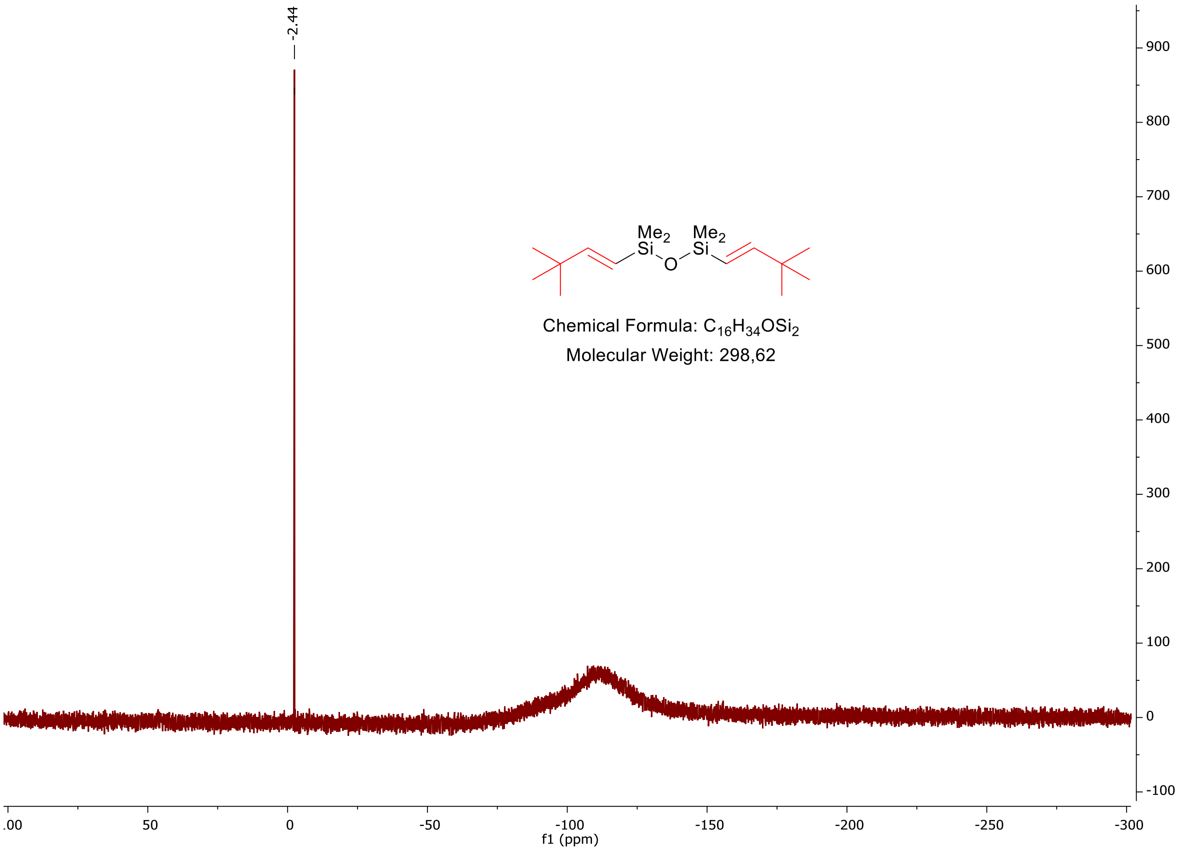

Figure S88. ^{29}Si NMR spectrum of **5i.**

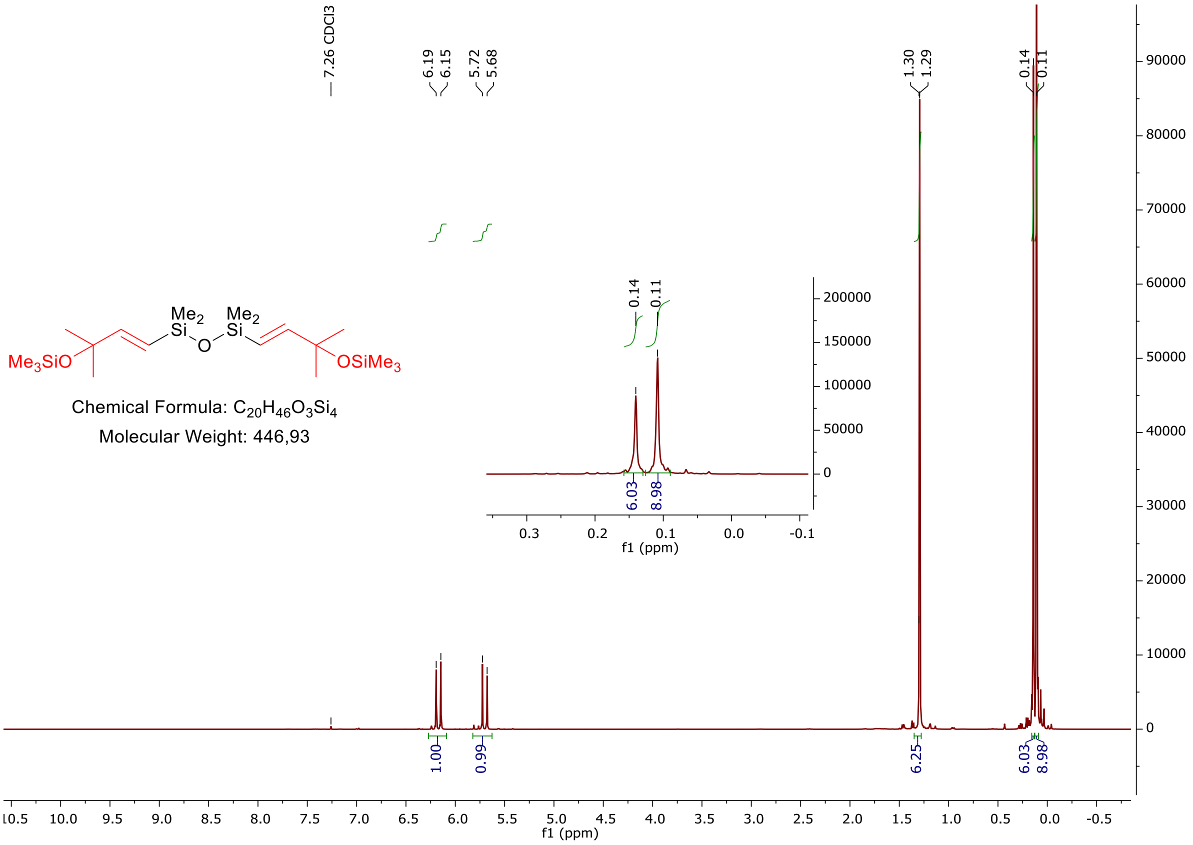

Figure S89. ^{1}H NMR spectrum of **5j**.

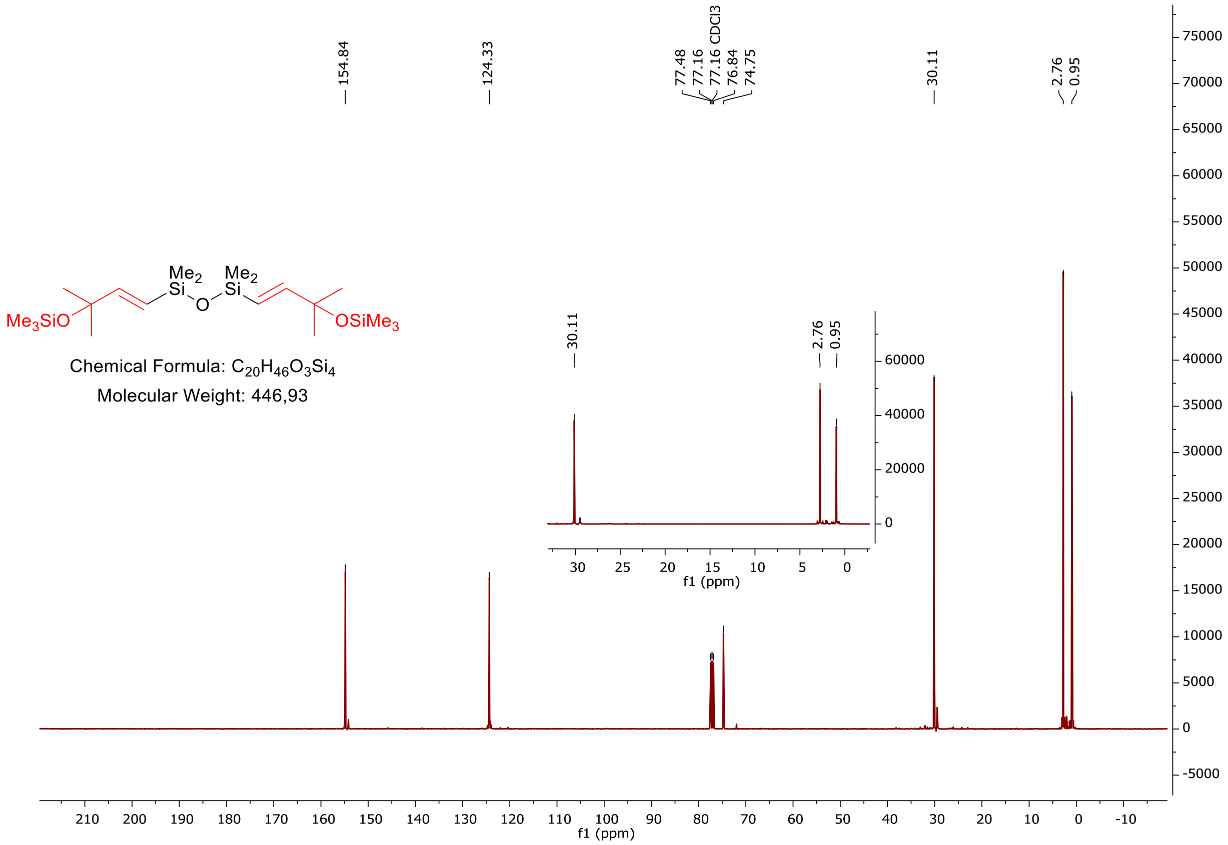

Figure S90. ^{13}C NMR spectrum of **5j**.

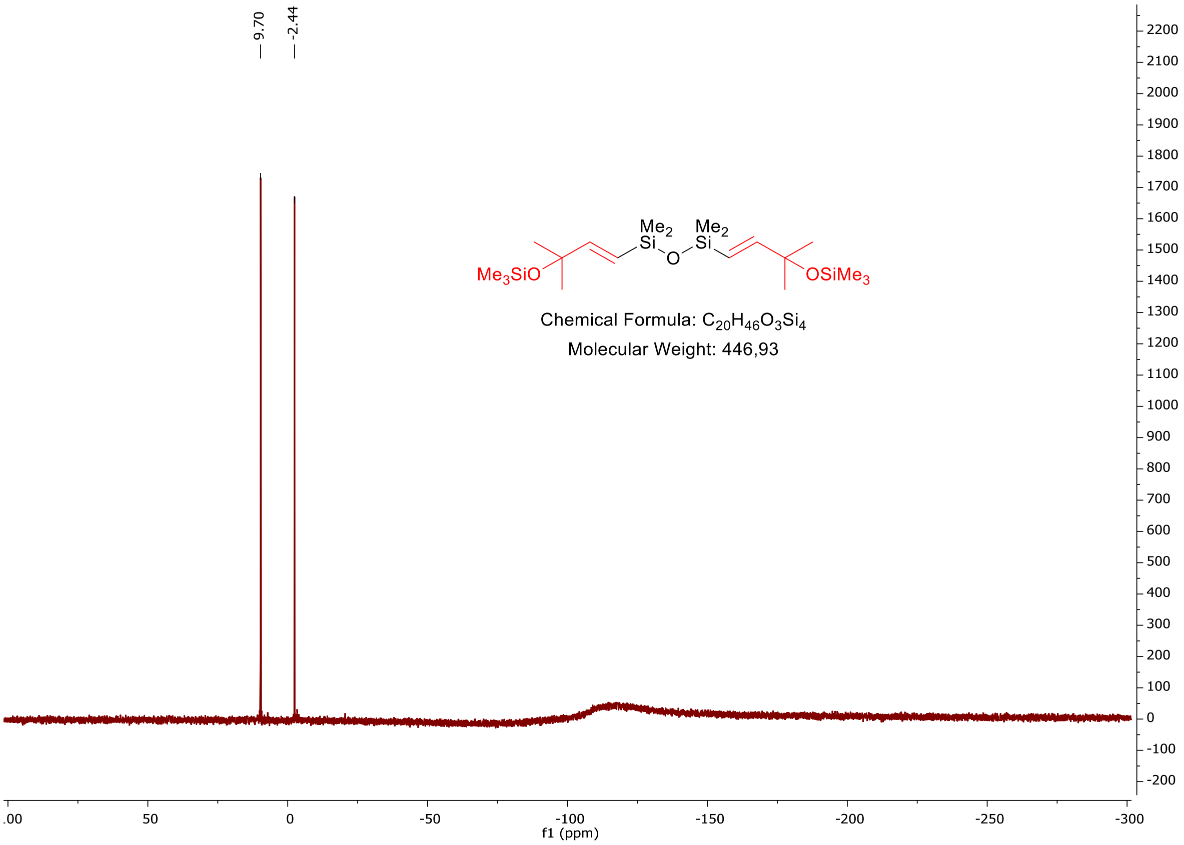

Figure S91. ^{29}Si NMR spectrum of **5j**.

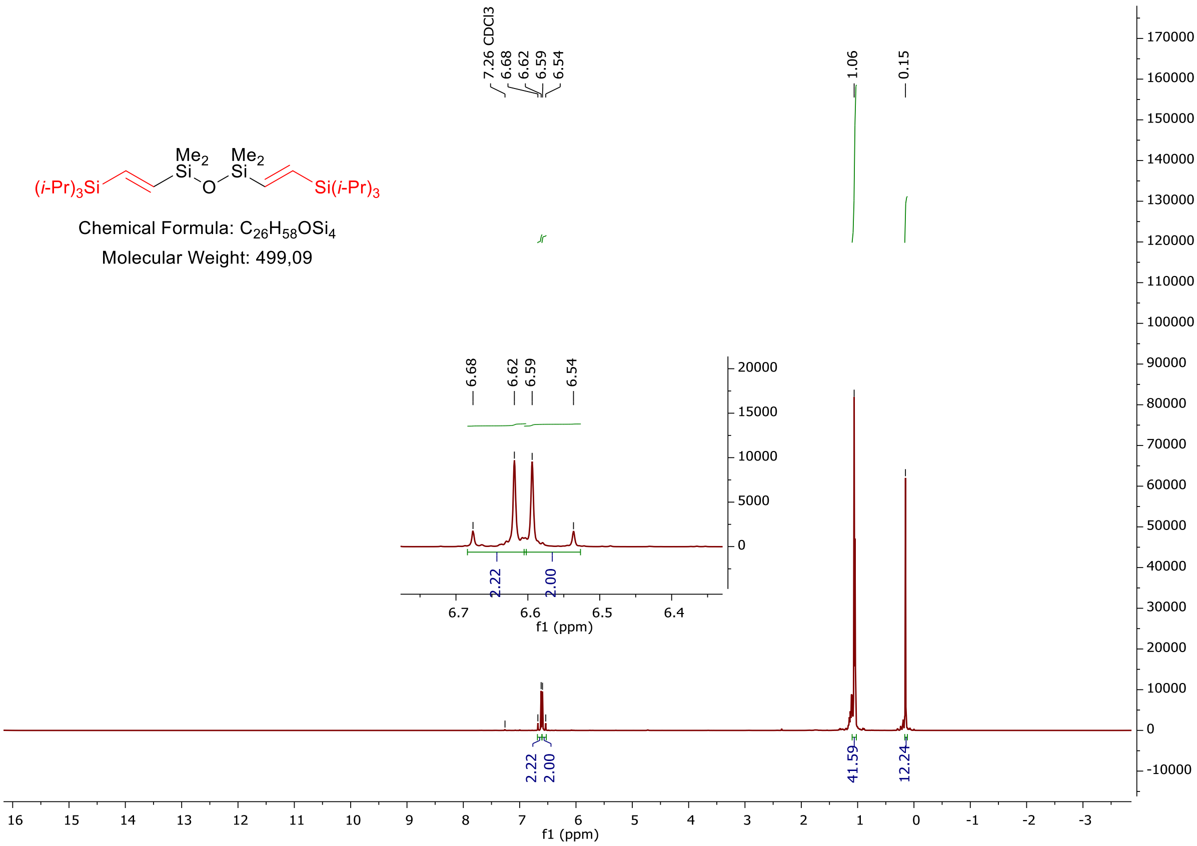

Figure S92. ^{1}H NMR spectrum of **5k**.

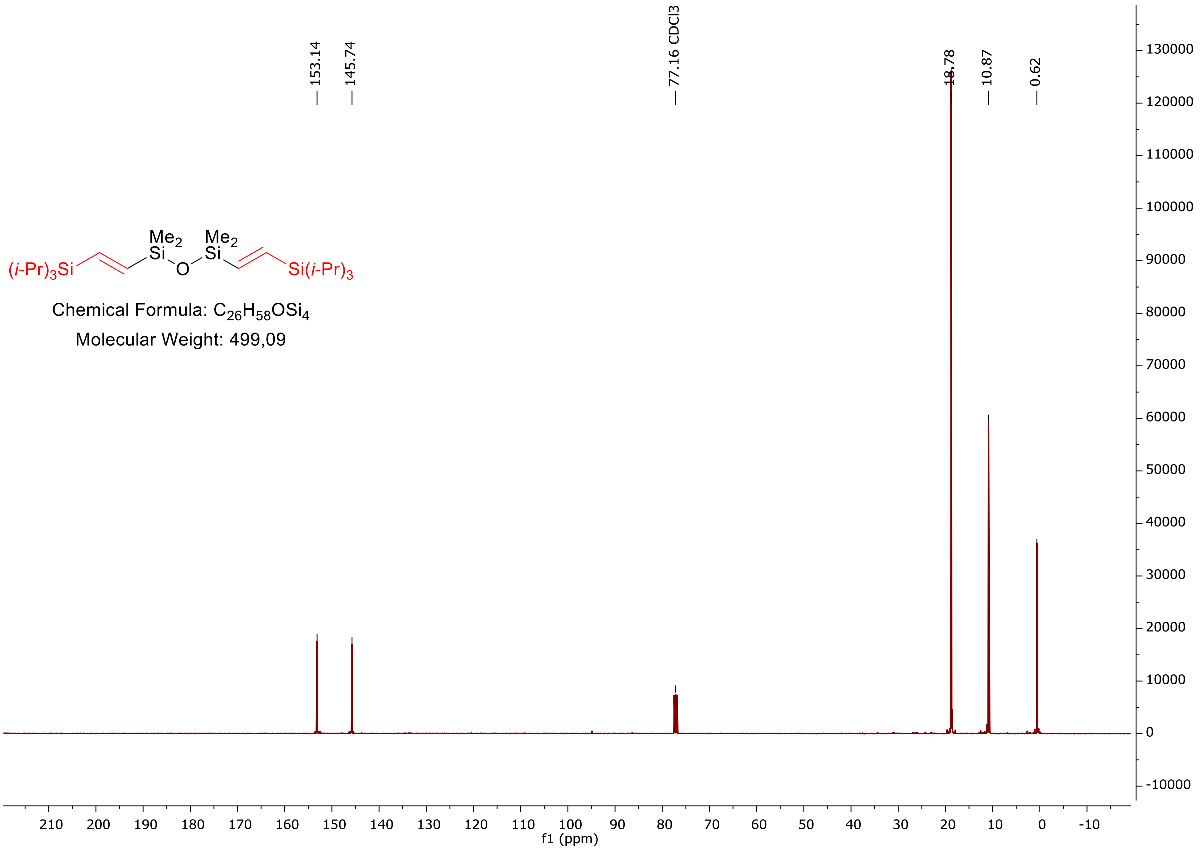

Figure S93. ^{13}C NMR spectrum of **5k.**

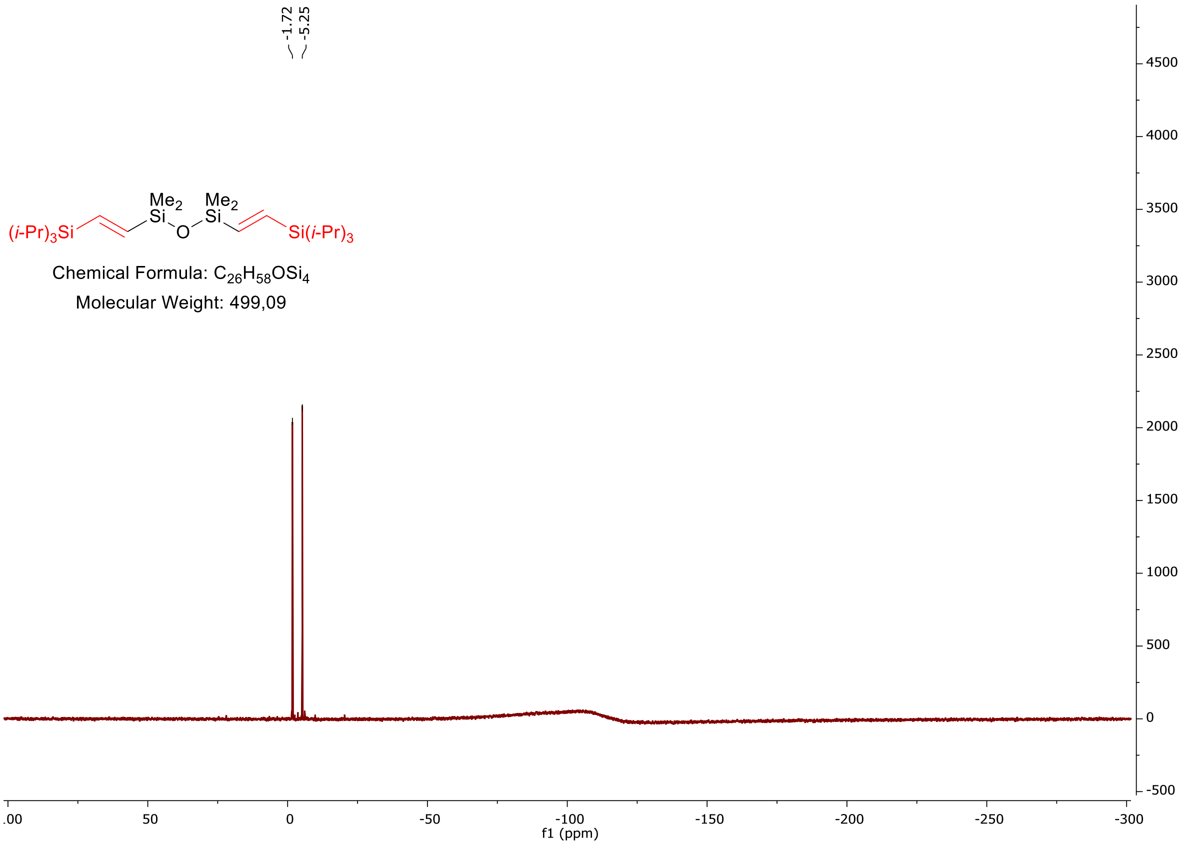

Figure S94. ^{29}Si NMR spectrum of **5k.**

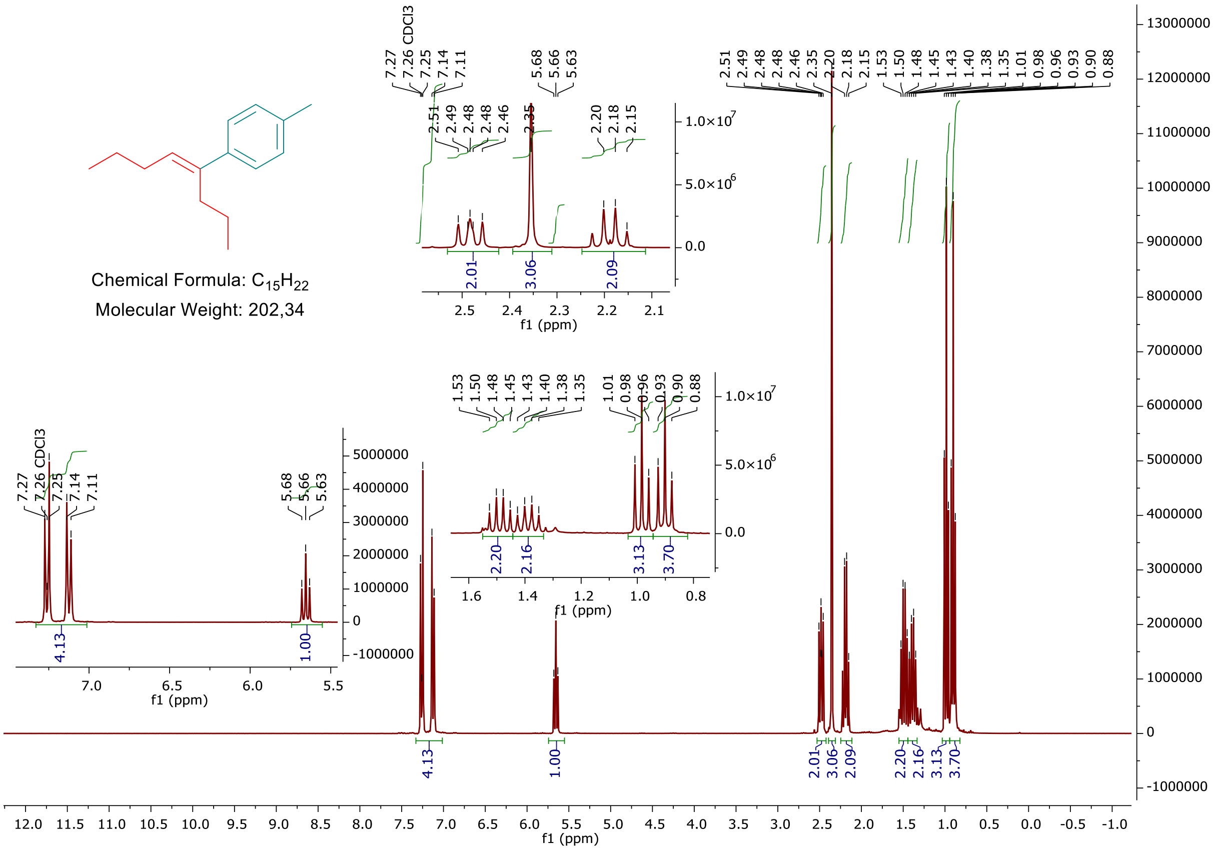

Figure S95. ^{1}H NMR spectrum of **7a**.

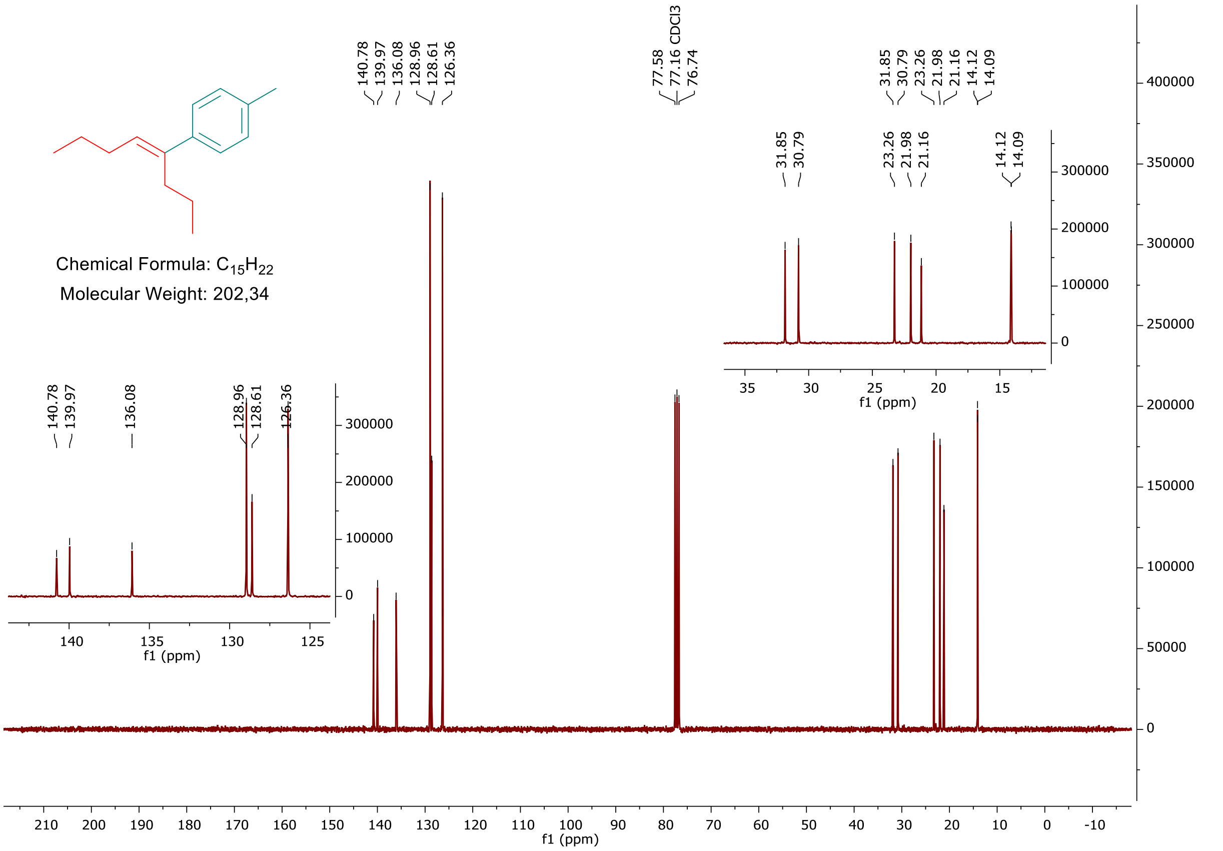

Figure S96. ^{13}C NMR spectrum of **7a**.

6. ESI-HRMS spectra

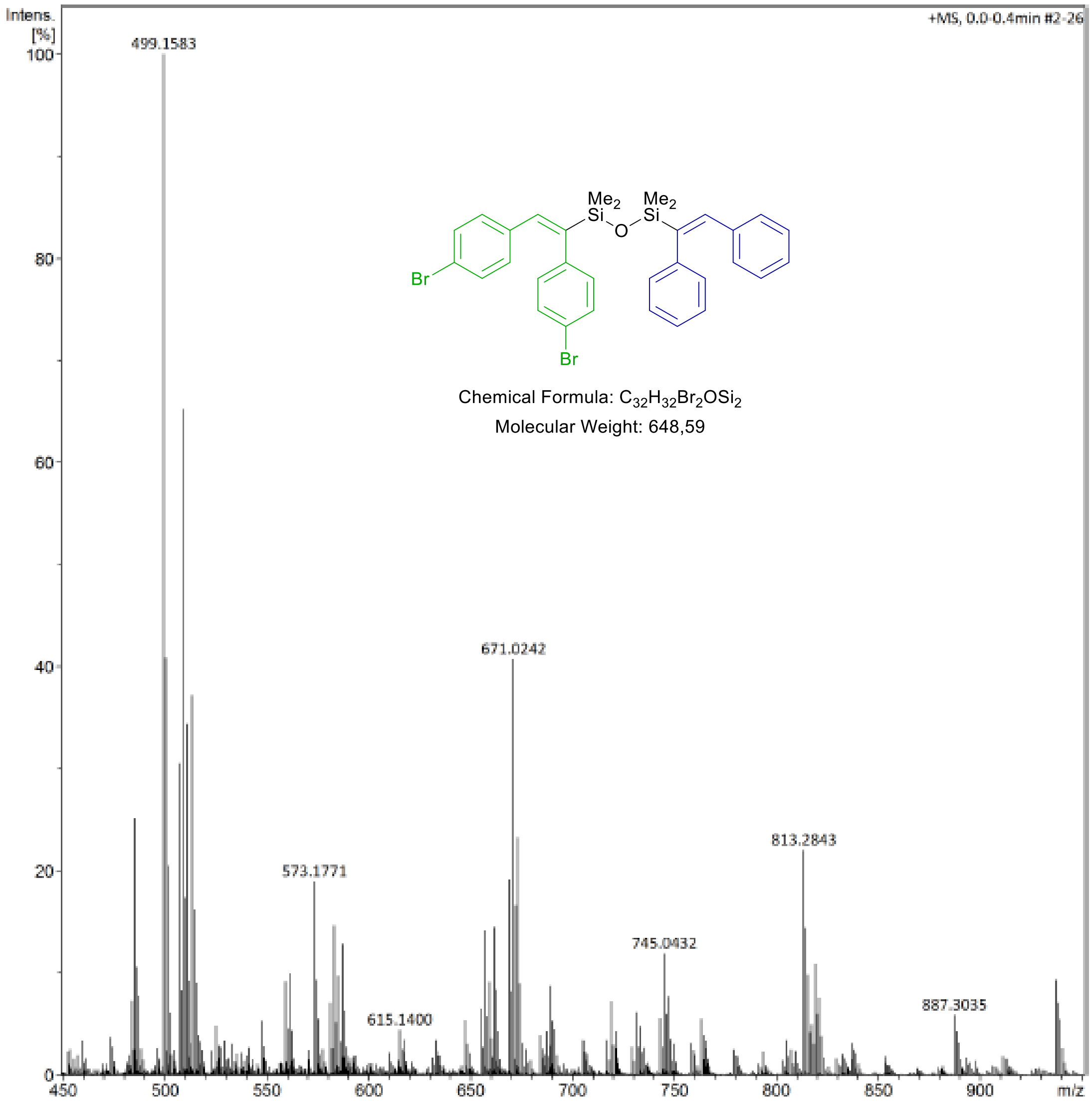

Figure S97. ESI-HRMS spectrum of **4b.**

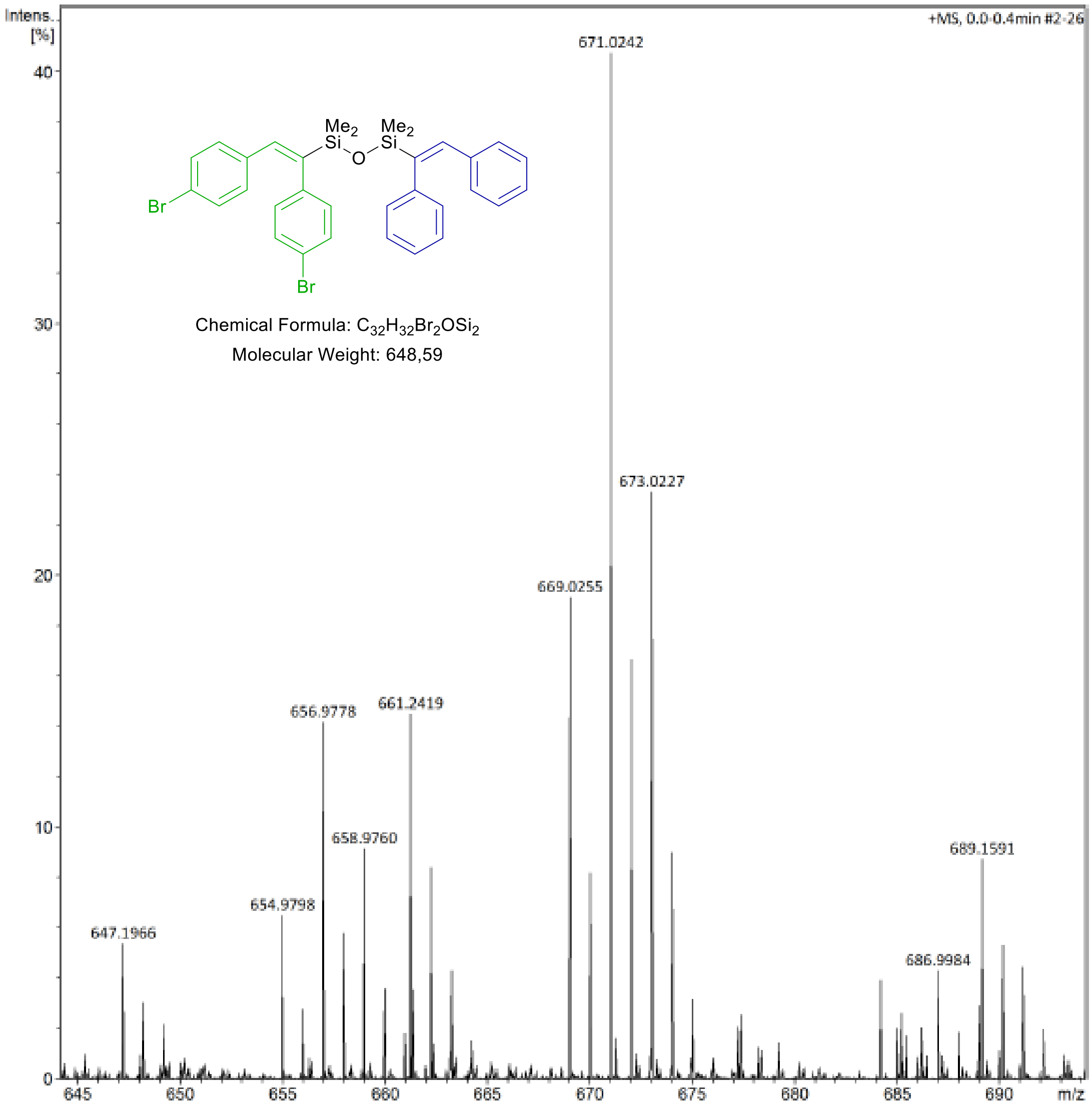

Figure S98. ESI-HRMS spectrum of **4b.**

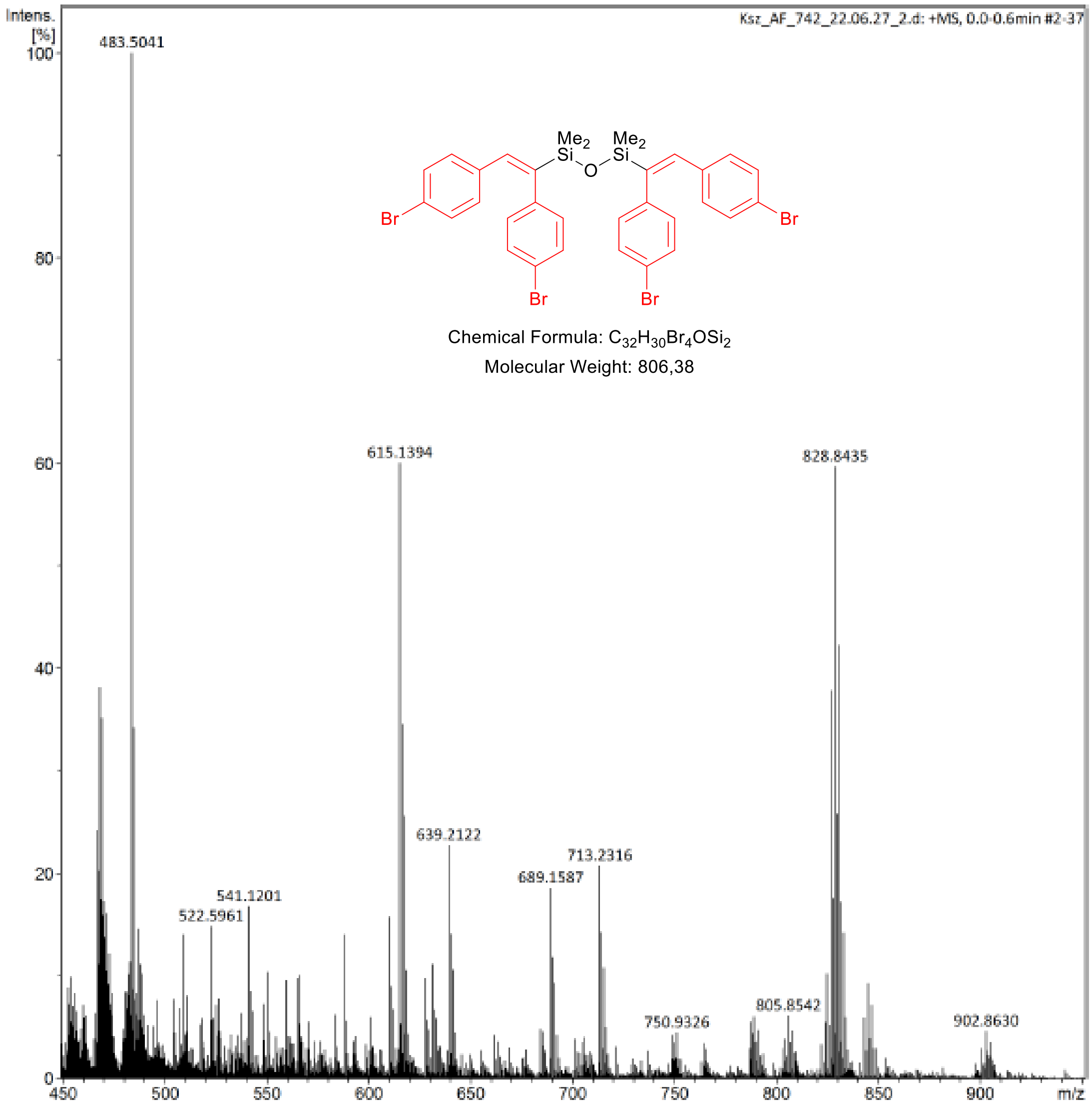

Figure S99. ESI-HRMS spectrum of **5c**.

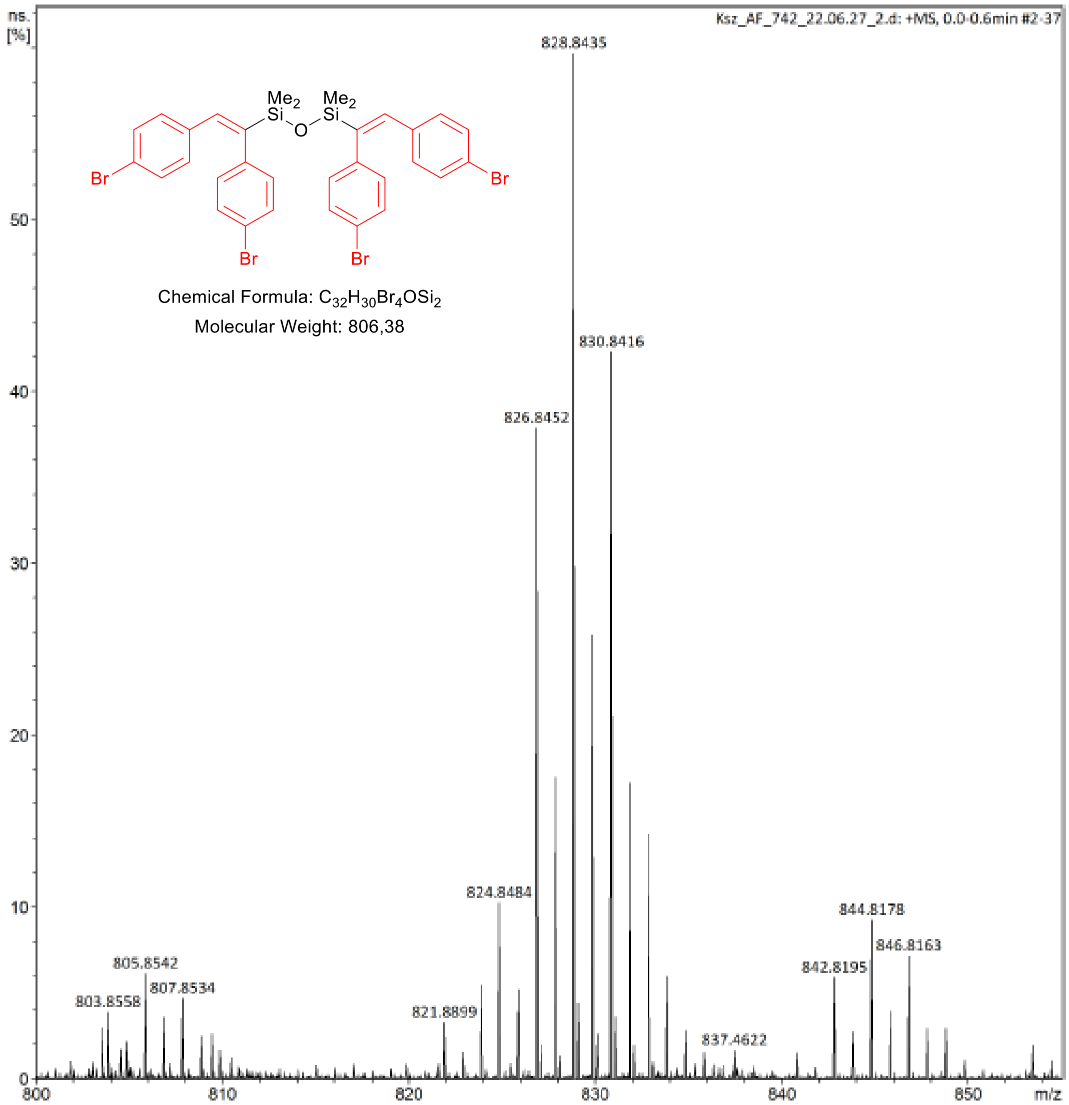

Figure S100. ESI-HRMS spectrum of **5c**.

7. Single crystal X-ray diffraction data

CCDC 2222532-2222534 contains the supplementary crystallographic data for this paper. These data can be obtained free of charge via www.ccdc.cam.ac.uk/data_request/cif, or by emailing data_request@ccdc.cam.ac.uk, or by contacting The Cambridge Crystallographic Data Centre, 12 Union Road, Cambridge CB2 1EZ, UK; fax: +44 1223 336033.

The molecule of compound **5g** in the crystal is disordered and the phenyl ring can take one of two alternative positions, as shown in Figure S101. The refined occupancy factors for these fragments are: 0.58 and 0.42. In addition, disorder of the second CF_3 group, rotating around the C-C bond, is observed (refined occupancy factors are 0.72 : 0.28).

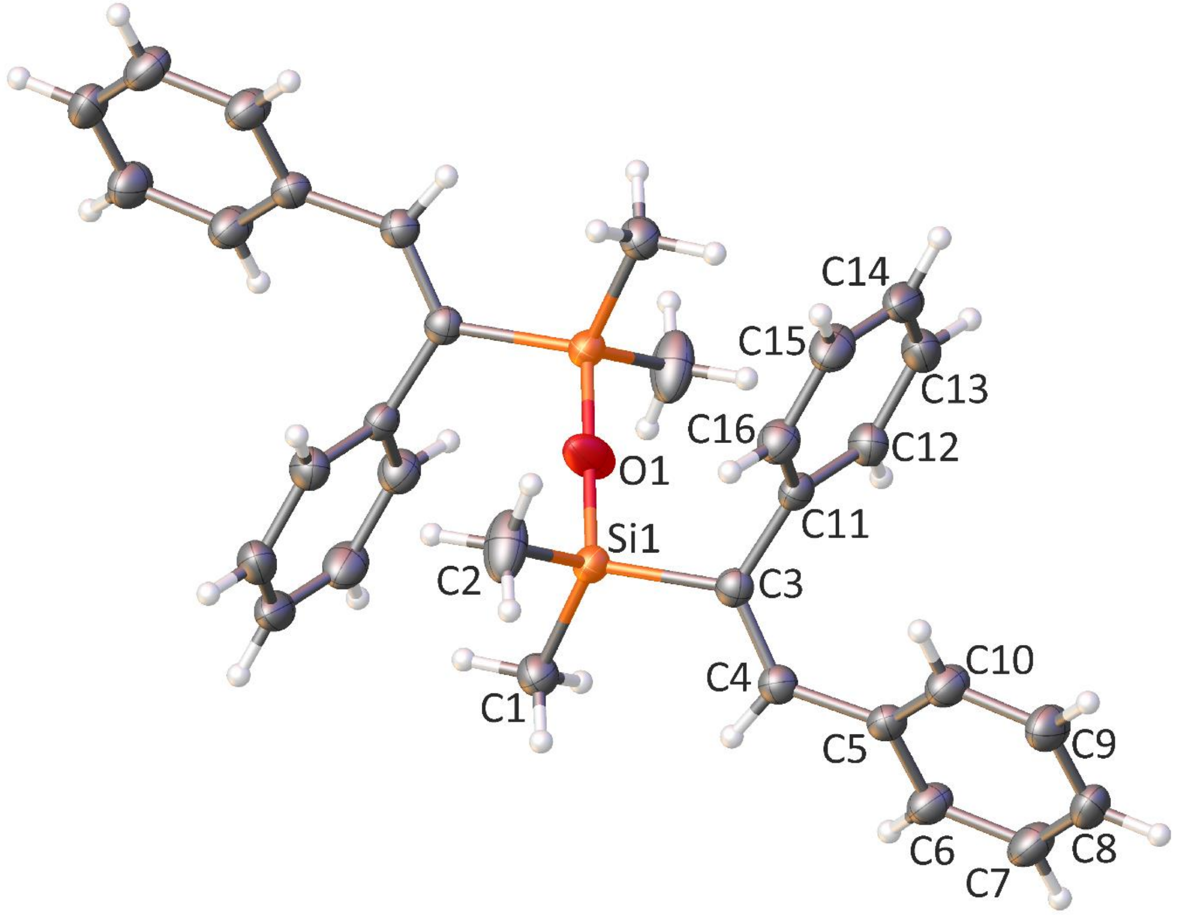

Figure S101. Molecular structure of compound **5b** (numbering scheme for asymmetric part of the molecule only). Displacement ellipsoids shown at the 50% probability level.

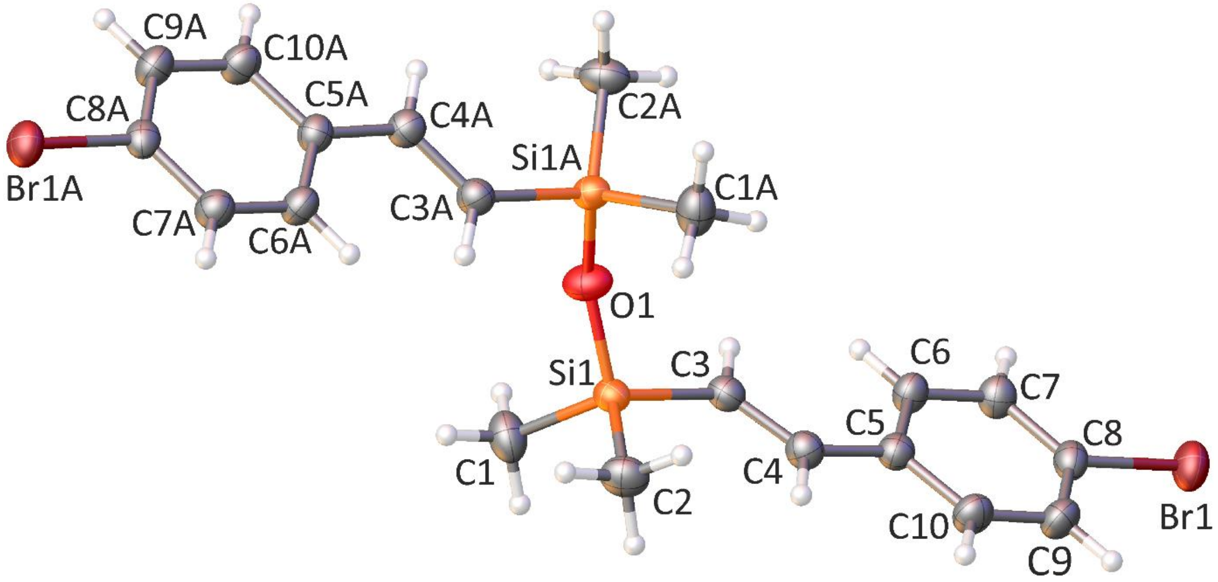

Figure S102. Molecular structure of compound **5e** and atoms numbering scheme. Displacement ellipsoids shown at the 50% probability level.

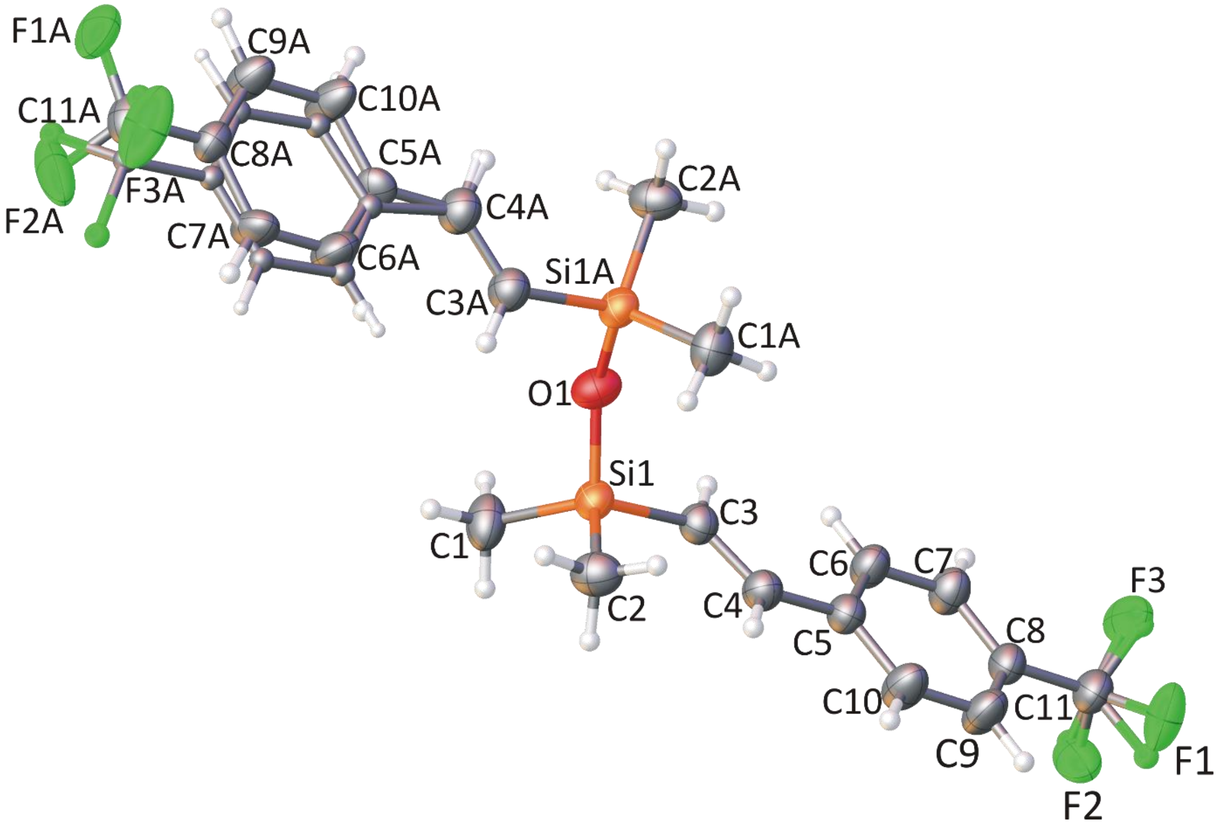

Figure S103. Molecular structure of compound **5e** and atoms numbering scheme (atomic numbering shown for part with the higher occupancy factors only). Displacement ellipsoids shown at the 50% probability level.

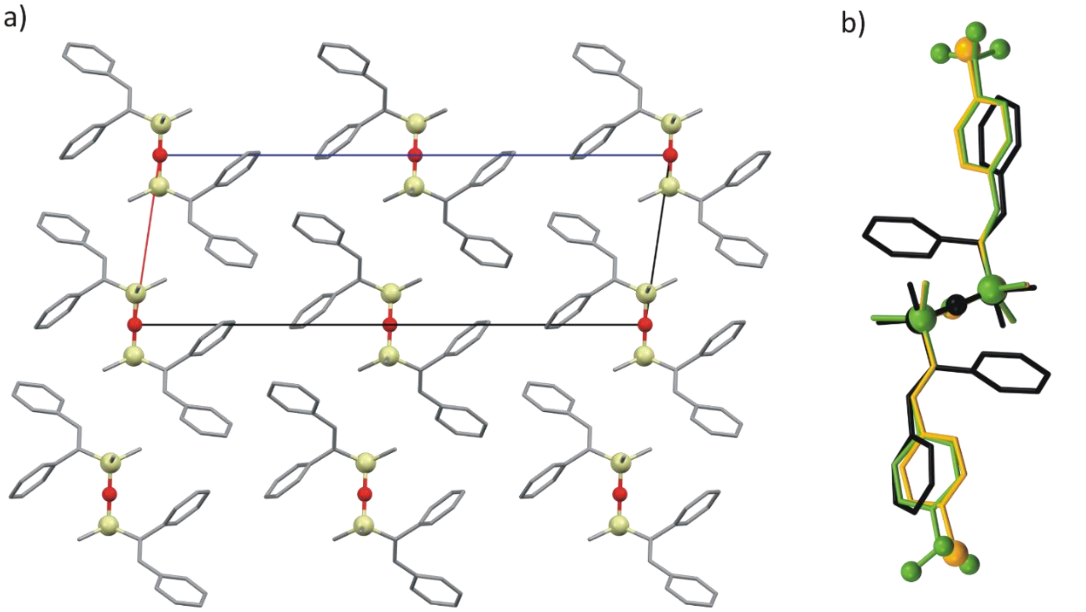

Figure S104. a) Molecular packing in crystal of compound **5b** and b) comparison of molecular conformations of compounds **5b** (black) **5e** (yellow) and **5g** (green). Hydrogen atoms are omitted for clarity. Non-H and non-C atoms are shown as balls.

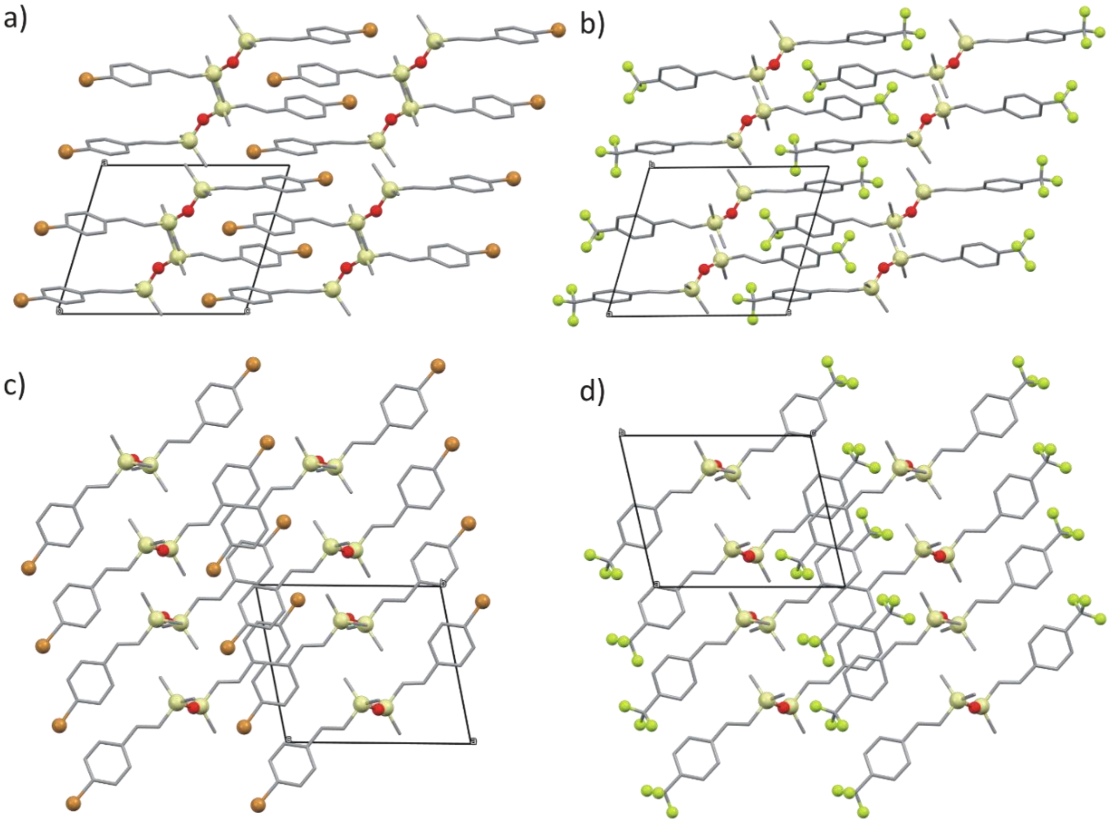

Figure S105. Molecular packing in isostructural crystals of compounds **5e**: a) view along b-axis, c) view along a-axis and **5e** b) view along a-axis, d) view along b-axis. Hydrogen atoms are omitted for clarity, non-H and non-C atoms are shown as balls.

Table S1. Selected crystal data and structure refinement details.

	5b	***5e***	***5g***
CCDC number	2222532	2222533	2222534
Chemical formula	$C_{32}H_{34}OSi_2$	$C_{20}H_{24}Br_2OSi_2$	$C_{22}H_{24}F_6OSi_2$
Mr	490.77	496.39	474.59
Crystal system, space group	Monoclinic, $P2_1/c$	Triclinic, $P\bar{1}$	Triclinic, $P\bar{1}$
Temperature (K)	130	130	130
a, *b*, *c* (Å)	8.6697 (1),<br>6.3113 (1),<br>25.8379 (2)	10.0717 (4),<br>10.3279 (4),<br>12.0737 (6)	10.0937 (7),<br>10.8704 (7),<br>12.5174 (12)
α, β, γ (°)	98.460 (1)	73.130 (4),<br>69.239 (4),<br>71.280 (4)	68.804 (7),<br>71.379 (7),<br>70.024 (6)
V (Å^3)	1398.39 (3)	1089.92 (9)	1173.06 (17)
Z	2	2	2
D_x (Mg m^{-3})	1.166	1.512	1.345
Radiation type	Cu *K*α	Cu *K*α	Cu *K*α
μ (mm^{-1})	1.31	5.81	1.92
Crystal size (mm)	0.45 × 0.12 × 0.04	0.3 × 0.2 × 0.05	0.3 × 0.1 × 0.05
No. of measured, independent and observed [$I > 2\sigma(I)$] reflections	19584, 2895, 2813	17215, 4495, 4202	20270, 4859, 4059
R_{int}	0.021	0.024	0.031
Range of *h*, *k*, *l*	*h* = -10→10,<br>*k* = -7→7,<br>*l* = -32→32	*h* = -12→12,<br>*k* = -12→12,<br>*l* = -13→15	*h* = -12→12,<br>*k* = -13→13,<br>*l* = -15→14
$R[F^2 > 2\sigma(F^2)]$, $wR(F^2)$, *S*	0.033, 0.088, 1.05	0.025, 0.072, 1.07	0.040, 0.116, 1.06
No. of parameters	162	231	404
$\Delta\rangle_{max}$, $\Delta\rangle_{min}$ (e Å^{-3})	0.26, -0.25	0.34, -0.48	0.38, -0.27

Table S2. Selected geometrical parameters (Å, °) in molecules of **5b**, **5e**, **5g**.

	**5b**	**5e**	**5g**
Si1—O1	1.6061 (3)	1.6329 (12)	1.6357 (13)
Si1—C3	1.8795 (12)	1.8549 (17)	1.8579 (16)
C3—C4	1.3422 (17)	1.332 (2)	1.327 (2)
C5—C4	1.4770 (16)	1.469 (2)	1.478 (2)
Si1A—O1		1.6324 (13)	1.6342 (13)
Si1A—C3A		1.8531 (17)	1.8616 (17)
C3A—C4A		1.326 (2)	1.323 (3)
C5A—C4A		1.474 (2)	1.395 (13)
Si1—O1—Si1^{i}	180.0		
Si1—O1—Si1A		151.29 (9)	147.68 (9)
Si1—C3—C4—C5	-179.33 (10)	179.38 (13)	179.24 (13)
Si1A—C3A—C4A—C5A		-179.43 (13)	-179.7 (8)
			-177.7 (9)

(Symmetry code(s): (i) -*x*, -*y*+1, -*z*+1.)

8. Literature

(1) **CrysAlis PRO 1.171.42.73a, Rigaku Oxford Diffraction, 2022.**

(2) Sheldrick, G. M. SHELXT–Integrated space-group and crystal-structure determination. *Acta Crystallographica Section A: Foundations and Advances* **2015**, *71*, 3.

(3) Sheldrick, G. M. Crystal structure refinement with SHELXL. *Acta Crystallographica Section C: Structural Chemistry* **2015**, *71*, 3.

(4) Dolomanov, O. V.; Bourhis, L. J.; Gildea, R. J.; Howard, J. A.; Puschmann, H. OLEX2: a complete structure solution, refinement and analysis program. *Journal of applied crystallography* **2009**, *42*, 339.

(5) Macrae, C. F.; Sovago, I.; Cottrell, S. J.; Galek, P. T.; McCabe, P.; Pidcock, E.; Platings, M.; Shields, G. P.; Stevens, J. S.; Towler, M. Mercury 4.0: From visualization to analysis, design and prediction. *Journal of applied crystallography* **2020**, *53*, 226.

(6) Yamagishi, M.; Nishigai, K.; Hata, T.; Urabe, H. Nucleophilic Addition of Sulfonamides to Bromoacetylenes: Facile Preparation of Pyrroles. *Organic Letters* **2011**, *13*, 4873.

(7) Marino, J. P.; Nguyen, H. N. Bulky trialkylsilyl acetylenes in the Cadiot− Chodkiewicz cross-coupling reaction. *The Journal of Organic Chemistry* **2002**, *67*, 6841.

(8) Ji, X.; Nie, J.; Peng, X.; Hu, J.; Xu, X.; Huang, Y.; Li, Y.; Jiang, H. Palladium-Catalyzed Cross Haloalkynylation of Haloalkynes. *Organic letters* **2022**, *24*, 3384.

(9) Lutter, F. H.; Grokenberger, L.; Spieß, P.; Hammann, J. M.; Karaghiosoff, K.; Knochel, P. Cobalt-Catalyzed Cross-Coupling of Functionalized Alkylzinc Reagents with (Hetero)Aryl Halides. *Angewandte Chemie International Edition* **2020**, *59*, 5546.

(10) Liu, J.; Yang, J.; Baumann, W.; Jackstell, R.; Beller, M. Stereoselective Synthesis of Highly Substituted Conjugated Dienes via Pd-Catalyzed Carbonylation of 1,3-Diynes. *Angewandte Chemie International Edition* **2019**, *58*, 10683.

(11) Schörgenhumer, J.; Waser, M. Transition metal-free coupling of terminal alkynes and hypervalent iodine-based alkyne-transfer reagents to access unsymmetrical 1,3-diynes. *Organic & Biomolecular Chemistry* **2018**, *16*, 7561.

(12) Tanaka, M.; Uchimaru, Y.; Lautenschlager, H. J. Platinum-complex-catalyzed dehydrogenative double silylation of acetylenes, dienes, and olefins with bis(hydrosilanes). *Organometallics* **1991**, *10*, 16.

(13) Cano, R.; Yus, M.; Ramón, D. J. Impregnated Platinum on Magnetite as an Efficient, Fast, and Recyclable Catalyst for the Hydrosilylation of Alkynes. *ACS Catalysis* **2012**, *2*, 1070.

(14) Zhou, H.; Wang, Y.-B. Copper(I)-Catalyzed Highly Regio- and Stereoselective Hydrosilylation of Terminal Alkynes with Boryldisiloxane. *ChemCatChem* **2014**, *6*, 2512.

(15) Martin, S. E.; Watson, D. A. Preparation of vinyl silyl ethers and disiloxanes via the silyl-Heck reaction of silyl ditriflates. *Journal of the American Chemical Society* **2013**, *135*, 13330.

(16) Hayashi, T.; Inoue, K.; Taniguchi, N.; Ogasawara, M. Rhodium-Catalyzed Hydroarylation of Alkynes with Arylboronic Acids: 1,4-Shift of Rhodium from 2-Aryl-1-alkenylrhodium to 2-Alkenylarylrhodium Intermediate. *Journal of the American Chemical Society* **2001**, *123*, 9918.
